# Reassessing ecdysteroidogenic cells from the cell membrane receptors’ perspective

**DOI:** 10.1038/srep20229

**Published:** 2016-02-05

**Authors:** Alexandros Alexandratos, Panagiotis Moulos, Ioannis Nellas, Konstantinos Mavridis, Skarlatos G. Dedos

**Affiliations:** 1 Department of Biology, National and Kapodistrian University of Athens, Athens 157 84, Greece; 2 HybridStat Predictive Analytics, Aiolou 19, 10551, Athens, Greece; 3 Institute of Molecular Biology and Genetics, Biomedical Sciences Research Center ‘Alexander Fleming’, Fleming 34, 16672, Vari, Greece

## Abstract

Ecdysteroids secreted by the prothoracic gland (PG) cells of insects control the developmental timing of their immature life stages. These cells have been historically considered as carrying out a single function in insects, namely the biochemical conversion of cholesterol to ecdysteroids and their secretion. A growing body of evidence shows that PG cells receive multiple cues during insect development so we tested the hypothesis that they carry out more than just one function in insects. We characterised the molecular nature and developmental profiles of cell membrane receptors in PG cells of *Bombyx mori* during the final larval stage and determined what receptors decode nutritional, developmental and physiological signals. Through iterative approaches we identified a complex repertoire of cell membrane receptors that are expressed in intricate patterns and activate previously unidentified signal transduction cascades in PG cells. The expression patterns of some of these receptors explain precisely the mechanisms that are known to control ecdysteroidogenesis. However, the presence of receptors for the notch, hedgehog and wingless signalling pathways and the expression of innate immunity-related receptors such as phagocytosis receptors, receptors for microbial ligands and Toll-like receptors call for a re-evaluation of the role these cells play in insects.

Cells decode information about their extracellular environment and integrate cues they receive into timely and appropriate physiological and developmental responses that serve a specific purpose. This cannot be more elegantly illustrated than in cells that play a particular and crucial role in development such as the prothoracic gland (PG) cells of insects. The PGs have been historically considered as the tissue responsible for synthesis and secretion of ecdysteroids that control and coordinate the development of immature insect stages^1^,^2^. This is the only demonstrated function of the PG cells and virtually every research that has been conducted on these cells has been guided by this principle. In Lepidoptera, in particular, the PG is an anatomically distinct tissue composed of a single type of cells^3^. Once fulfilling their documented role in immature insect stages, PGs undergo apoptosis during the transition from the pupal to adult stage or early in the adult stage when enough ecdysteroids have been produced to accomplish the final moult^1^. This programmed cell death of PG cells occurs also in insects that possess a ring gland, where the PG is part of a composite, multi-tissue organ^1^,^4^.

A growing body of evidence shows that the PGs receive a multiplicity of signals from other insect tissues and respond by secreting ecdysteroids through integration of a very broad array of second messengers and signalling modules^1^,^4^,^5^. The regulatory mechanisms of ecdysteroids synthesis and secretion are quite complex and become even more perplexing as additional ligands for receptors are identified that stimulate or inhibit ecdysteroids secretion^6^,^7^,^8^. The documented multiplicity of cell membrane receptors that shape the steroidogenic response of these cells has been growing at a rapidly accelerated pace^1^,^4^ that calls for a total re-evaluation of the array of extracellular stimuli that these cells receive simply to carry out the task of synthesising and secreting ecdysteroids. Are PGs carrying out just a single function during insect development?

The most affirmative way to answer this question is to identify the cell membrane receptors that these cells use to decode and transduce information from the extracellular environment, so in this study we carried out a systematic analysis of the cell membrane receptors that are involved in signal transduction and are expressed by the PG cells during the final larval stage of the model insect, *Bombyx mori*. We used bioinformatic analysis to identify known or candidate genes for G protein-coupled receptors (GPCRs), receptor tyrosine kinases (RTKs), receptor serine/threonine kinases (RTSKs), receptor tyrosine phosphatases (RTPs), receptor type guanylate cyclases (RTGCs), integrins, innate immunity-related receptors as well as other cell membrane receptors. Upon generating a list of 369 genes that code for cell membrane receptors involved in signal transduction in this insect, we combined proteomics, transcriptomics and quantitative PCR data analysis to identify which of these receptors are expressed by PG cells and thus provide evidence for other functions that are carried out by PGs in insects. Our results show that PG cells express 104 genes encoding for various types of cell membrane receptors during the last larval instar and the beginning of the pupal stage, before these cells undergo apoptosis. Most of these genes exhibit temporal expression profiles that are inconsistent with the ecdysteroidogenic activity of these cells, whereas others have temporal expression profiles consistent with the already known mode of action of the coding protein as a positive or negative regulator of ecdysteroidogenesis^4^. The combined expression profiles, the puzzling array of different receptor proteins we identified and the expression of specific immunity-related receptors call into question the scientific principle that PGs function in immature insect stages only as the provider of ecdysteroids. In many respects, such as its function at immature life stages, its endocrine properties, its apoptosis once ecdysteroids are produced and the presence of immunity-related receptors, the prothoracic gland shows homoplacious characteristics with the thymus gland of jawed vertebrates^9^, an organ of critical importance for the immature life stages that becomes atrophic in adult stages.

## Results

### Ecdysteroidogenic capacity of prothoracic gland cells

**Figure 1:**
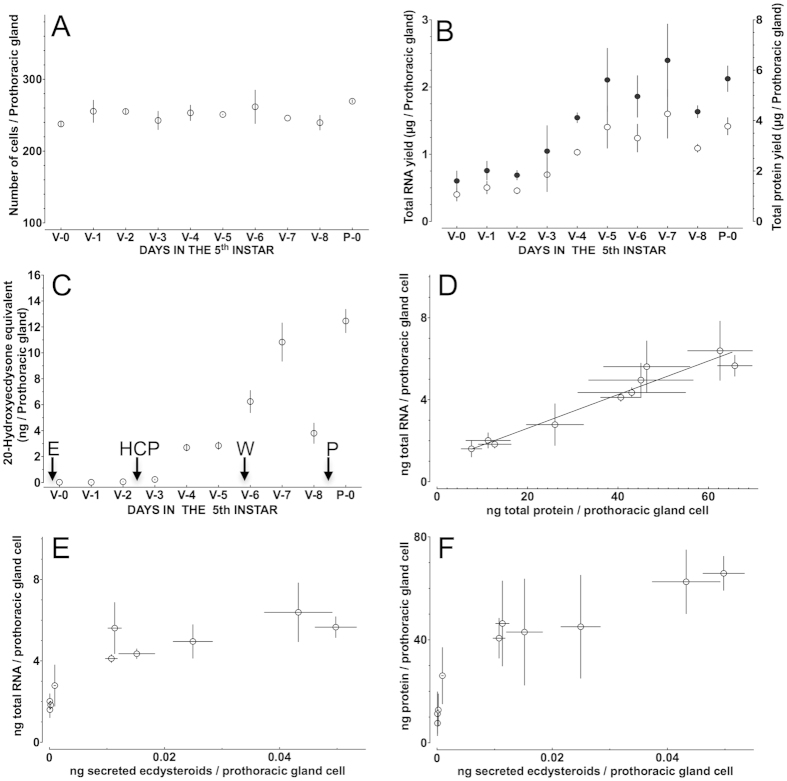
Biochemical units can be expressed at the single cell level in prothoracic glands. (**A**) Fluctuation in cell number of PGs during the final larval instar and the 1^st^ day of the pupal stage (n = 7) (One way ANOVA: p = 0.56). (**B**) Total RNA yields (left axis, open circles) and total protein content (right axis, filled circles) during the final larval instar and the 1^st^ day of the pupal stage (n = 3–7). (**C**) Ecdysteroids secretion by the prothoracic glands at the same developmental period (n = 7). Arrows indicate the time of ecdysis (E) to the final larval stage, the time of head critical period (HCP; see text for details), the time of feeding cessation and onset of wandering (W) behaviour and the time of metamorphosis to pupa (P). (**D**) Correlation between RNA yield and protein content in a prothoracic gland cell at the same developmental period. (**E**) Absence of a linear relationship between ecdysteroids secretion and total RNA content in PG cells expressed at a single cell level. (**F**) Absence of a linear relationship between ecdysteroids secretion and total protein content in PG cells expressed at a single cell level.

The number of cells that comprise the PG remains constant (mean ± SEM: 250.3 ± 3.30) throughout the 5^th^ instar and the 1^st^ day of the pupal stage of *B. mori* (Fig. 1A). However, the amount of protein and the total RNA yield per PG is gradually increasing with peaks occurring on V-7 and P-0 (Fig. 1B). Similarly, ecdysteroids secretion shows peaks on V-7 and P-0 (Fig. 1C).

The presence of a constant number of cells in the PGs, allowed us to normalise and express units at a single cell level, and thereby, while the correlation between the amount of protein and RNA yield per prothoracic gland cell is close to 1 (*r*^*2*^ = 0.94, Fig. 1D), there was no linear correlation between ecdysteroids secretion and RNA yield (*r*^*2*^ = 0.70, Fig. 1E) and ecdysteroids secretion and protein content (*r*^*2*^ = 0.81, Fig. 1F), an indication that specific signals regulate ecdysteroidogenesis beyond day 3 (V-3) in this insect (Fig. 1E,F).

### Comprehensive annotation of *B. mori* cell membrane receptors

Using bioinformatic analysis we mapped a total of 369 cell membrane receptor expressing genes (Table 1 and Supplementary Table S1) to chromosome and scaffold locations on *B. mori* genome. For genes already identified in *B. mori* we used the existing nomenclature and where *D. melanogaster* homologues existed for unidentified *B. mori* genes we named the genes after their closest homologue of *D. melanogaster*. Finally, when no homologue was identified in insect species we used the closest homologue as named in the signalling receptome webpage^10^ (Supplementary Table S1). NCBI reference sequence and Uniprot accessions, where available, are also provided for all genes used in this study in Supplementary Table S1.

As shown in Table 1, we identified a total of 119 GPCRs^11^, with 86 classified as Rhodopsin-like (Class A)^12^, 16 classified as Secretin-like (Class B)^13^, 9 classified as Metabotropic-glutamate-like, 5 belonging to the Frizzled/Smoothened group and 3 unclassified GPCRs. In one case (i.e. *Bombyx mori* prostanoid receptor-like), we used cDNA from PGs to amplify, clone and sequence the entire ORF of the putative gene (GenBank accession No. KT449429), before verifying its expression in PG cells. This total number of 119 GPCR genes is comparable to those described for *A. gambiae* (121)^12^ but smaller than the number of GPCRs identified in *D. melanogaster* (162)^12^. We mapped 64 odorant receptors^14^ to their chromosome or scaffold locations (Supplementary Table S1) and identified and mapped 71 gustatory receptors^15^ to their chromosome or scaffold locations (Supplementary Table S1). Using previously published data on dipteran species^16^, we identified 20 RTK genes (Supplementary Table S1), 5 RTSK genes (Supplementary Table S1) and 5 RTP genes (Supplementary Table S1) that are present in *B. mori* genome. Our search also identified 9 RTGC genes^16^ (Supplementary Table S1) and 11 integrins^17^ (Supplementary Table S1). We screened the lists of innate immunity-related genes^18^ for receptor proteins that possess transmembrane domains and identified 5 peptidoglycan recognition proteins (PGRPs), 13 Toll-like receptors, 2 homologues of *Nimrod*, 1 homologue of *Draper* and 1 homologue of *Domeless*^18^ (Supplementary Table S1). We expanded our search for other receptors that activate signal transduction pathways and identified 6 *B. mori* homologues of proteins that are members of the low-density lipoprotein receptor (LDLR) family^19^ (Supplementary Table S1). Members of this family, such as Arrow and the single transmembrane low-density lipoprotein receptor-related protein 5 or 6 (LRP5/6) are known to be involved in Wnt/Wingless signalling^19^. We also identified genes that code for proteins involved in Wnt signalling pathway such as the gene encoding for transmembrane protein 198 (*TMP198*)^20^, *furrowed*^21^ and 4 members of the Wnt receptor *Frizzled*^19^. The Hedgehog signalling pathway^22^ has been identified as a key regulator of ecdysteroidogenesis in the *D. melanogaster* ring gland^5^. We identified 5 genes that code for proteins with high sequence similarity to *Patched*, the Hedgehog receptor, and 1 gene that codes for the *B. mori* homologue of *Smoothened*^23^ (Supplementary Table S1). We also identified *Dispatched*^24^ and *Interference hedgehog*^25^ which have also been identified as components of the Hedgehog signalling pathway in *D. melanogaster*. We identified 3 members of the Notch signalling pathway, the *B. mori* homologue of *Serrate, Notch* and *Notch3*^26^ and *Uniflatable*, a gene that codes for a protein that participates in Notch signal transduction^27^, 2 homologues of the Tumor Necrosis Factor Receptor (*TNFR*)^28^, 2 homologues of Netrin receptors and the *B. mori* homologue of Frazzled^29^, the homologues of *D. melanogaster Fat* and *Daschous* involved in the Hippo signalling pathway and the *B. mori* homologue of Nephrin^30^, among others (Supplementary Table S1). Detailed descriptions for each of these genes as well as several others together with their chromosome location are provided in Supplementary Table S1.

### Proteome analysis of PG cells

**Figure 2:**
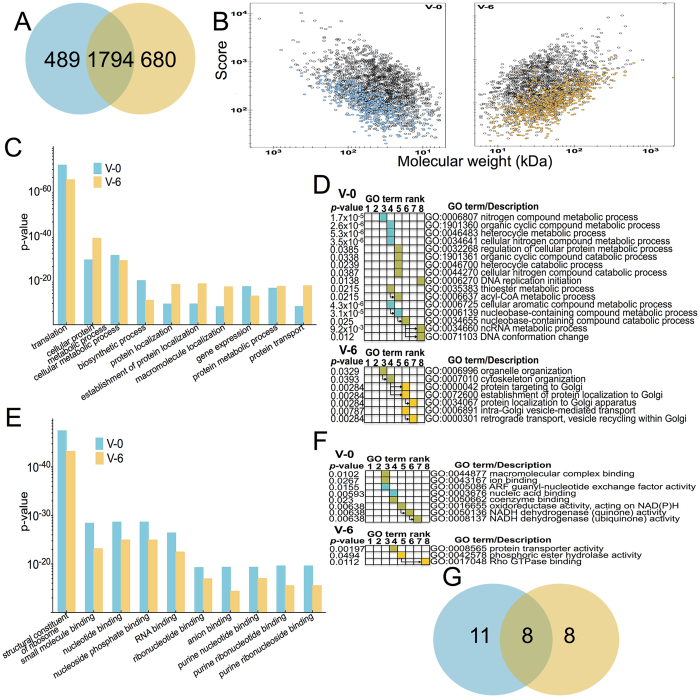
Proteomic analysis of prothoracic glands from day 0 (V-0, blue) and day 6 (V-6, yellow) of the 5^th^ instar. (**A**) Venn diagram of the common and unique proteins identified on these two days of development. (**B**) Volcano plots of the proteins identified on V-0 and V-6. Identified proteins are plotted based on their molecular weight (x-axis) versus their peptide ion scores during mass spectrometry analysis (y-axis). (**C**) g:Profiler results showing the 10 most significantly represented biological processes (GO terms) of the identified proteins. (**D**) Gene enrichment analysis generated by g:Profiler showing the unique and statistically significant biological processes on V-0 and V-6. Blue squares indicate GO terms enriched by the uniquely identified proteins on V-0. Yellow squares indicate GO terms enriched by the uniquely identified protein on V-6. Green squares indicate GO terms enriched by the proteins identified on both days. Arrows indicate the immediate hierarchical relationship of each term. (**E**) g:Profiler results showing the 10 most significantly represented molecular functions (GO terms) of the identified proteins. (**F**) Gene enrichment analysis generated by g:Profiler showing the unique and statistically significant molecular functions on V-0 and V-6. Blue squares indicate GO terms enriched by the uniquely identified proteins on V-0. Yellow squares indicate GO terms enriched by the uniquely identified protein on V-6. Green squares indicate GO terms enriched by the proteins identified on both days. Arrows indicate the immediate hierarchical relationship of each term. (**G**) Venn diagram of the common and unique receptor proteins identified on V-0 and V-6.

For proteomic analysis we used samples from day 0 and day 6 of the 5^th^ instar. The choice for these days of development was guided by previous research that showed the absence of response of PG cells to extracellular stimuli but the presence of several receptors on day 0^31^ and the involvement of several signalling pathways on ecdysteroidogenesis on day 6 of the 5^th^ instar^32^,^33^. We carried out a *de novo* analysis on the initial 3184 protein hits of the proteomic dataset from PG cells from V-0 and V-6 (Fig. 2). This analysis returned 221 (6.94%) hits as duplicate entries and false annotations and a total of 2963 proteins hits with 489 and 680 of those uniquely identified on V-0 and V-6 samples, respectively (Fig. 2A and Supplementary Table 2A,B). In both days, the identified proteins had similar average molecular weights (59.3 kDa (V-0) and 60.5 kDa (V-6)) and similar average ion scores of 394.5 ± 11.7 on V-0 and 410 ± 12.1 on V-6 (Fig. 2B). We employed Gene Ontology (GO) term enrichment analysis by g:Profiler^34^ to identify clusters of biological processes that were highly represented (p-value) on these two days and we identified translation as the most significantly represented biological process in both days (Fig. 2C). By focusing on the uniquely identified proteins on V-0 and V-6 and assessing how these proteins influence the GO term enrichment clustering, we observed that the uniquely identified proteins on V-0 influence the higher ranking biological processes while the uniquely identified proteins on V-6 enrich the lower ranking biological processes (Fig. 2D). More specifically, the uniquely identified proteins on V-0 are mostly involved in transcription or translation while the uniquely identified proteins on V-6 are mostly involved in protein targeting and localization to the Golgi apparatus of PG cells (Fig. 2D). GO term enrichment analysis of molecular functions of the identified proteins showed that ribosomal proteins represent the most statistically significant cluster (Fig. 2E). In a similar pattern as with biological processes, the uniquely identified proteins enriched the higher ranking molecular functions on V-0 while the uniquely identified proteins on V-6 enrich lower and highly specialised molecular functions in PG cells (Fig. 2F). Among the 2963 proteins, we identified 27 receptors (Fig. 2G and Supplementary Table S1 for detailed description), a low (0.009%) but expectable percentage given that transmembrane proteins are notoriously difficult to identify in proteomic analysis. For example, we did not identify Torso, the prothoracicotropic hormone receptor^35^, or the myosuppressin receptor^36^, both known to be expressed on day 6 (see Supplementary Table S2A,B). To understand whether the low yield was caused by the total protein extraction process of our samples, we analysed the raw proteomic dataset of a previous study in which the authors have investigated the proteomic profile of PGs from V-4 of the 5^th^ instar^37^. Using BLASTP searches of their dataset^37^ we detected 7 proteins that match our dataset of 369 cell membrane receptors.

### Transcriptome analysis of PG cells

**Figure 3:**
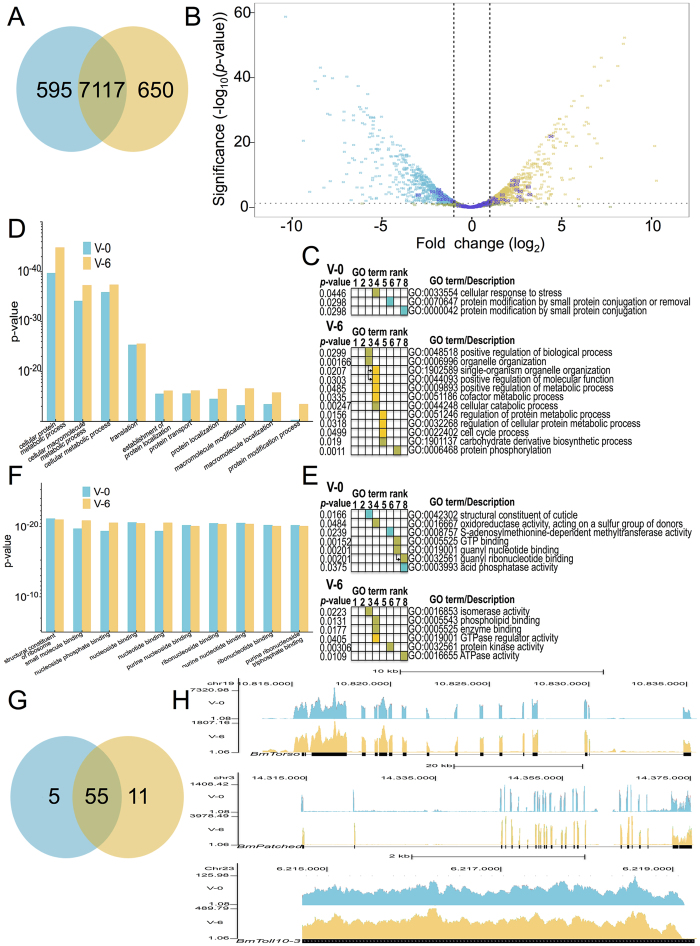
Transcriptome analysis of prothoracic glands from day 0 (V-0, blue) and day 6 (V-6, yellow) of the 5^th^ instar. (**A**) Venn diagram of the common and unique genes identified on these two days of development. (**B**) Volcano plot of the expressed genes identified on V-0 and V-6. (**C**) g:Profiler results showing the 10 most significantly represented biological processes (GO terms) of the identified genes. (**D**) Gene enrichment analysis generated by g:Profiler showing the unique and statistically significant biological processes on V-0 and V-6. Blue squares indicate GO terms enriched by the uniquely identified gene products on V-0. Yellow squares indicate GO terms enriched by the uniquely identified gene products on V-6. Green squares indicate GO terms enriched by the gene products identified on both days. Arrows indicate the immediate hierarchical relationship of each term. (**E)** g:Profiler results showing the 10 most significantly represented molecular functions (GO terms) of the identified genes. (**F**) Gene enrichment analysis generated by g:Profiler of the unique and statistically significant molecular functions on V-0 and V-6. Blue squares indicate GO terms enriched by the uniquely identified gene products on V-0. Yellow squares indicate GO terms enriched by the uniquely identified gene products on V-6. Green squares indicate GO terms enriched by the gene products identified on both days. (**G**) Venn diagram of the common and unique receptor proteins identified on V-0 and V-6. (**H**) Mapping of transcriptome reads to 3 exemplar receptor genes (*BmTorso* upper panel, *BmPatched* middle panel and *BmToll10-3* lower panel) on day 0 and day 6 of the 5^th^ instar.

To obtain a global view of gene activity in PGs at single-nucleotide resolution, we performed high-throughput RNA-Seq experiments using Illumina sequencing technology on poly (A)–enriched RNAs of samples from V-0 and V-6 of the 5^th^ instar. After removing low quality reads, a total of 3.15 × 10^8^ reads from V-0 and 3.26 × 10^8^ reads from V-6 were obtained from each day. Of these reads, 87.57 ± 0.05% and 86.74 ± 0.91% represented mapped reads from V-0 and V-6 respectively, while 82.54 ± 0.21% and 77.57 ± 4.67% represented uniquely mapped reads from the respective days. The total length of the mapped reads was about 56 gigabases (Gb), representing about a 130-fold coverage of the *B. mori* genome and more than 3.1 × 10^3^-fold coverage of the annotated transcriptome^38^. Transcript analysis of PG cells was carried out using the PANDORA method^39^ and setting the threshold of gene expression to the median reads per gene model (rpgm) value (≈ 0.0519 normalised reads per gene model length). Of the predicted 14623 protein-coding *B. mori* genes that were built by merging different gene datasets using GLEAN in the SilkDB^40^, 8362 were found to be expressed on V-0 and/or V-6 which covered 57.18% of all the predicted genes (Fig. 3A). Volcano plots of the expressed genes revealed that expression of receptor genes was mostly upregulated on V-6 (Fig. 3B). In the same way as with our proteomic analysis, we employed Gene Ontology (GO) term enrichment analysis by g:Profiler^34^ to identify clusters of biological processes that were highly represented (p-value) on these two days and we identified ribosomal proteins to be the most significantly represented genes in both days (Fig. 2C) while genes whose products are involved in protein trafficking and localization were predominantly expressed on V-6 (Fig. 3C). By focusing on the uniquely represented biological processes on V-0 and V-6, we observed that there is a substantial enrichment of unique biological processes identified on V-6 compared with V-0 (Fig. 3D). GO term enrichment analysis of molecular functions of the expressed genes on both days showed that genes expressing ribosomal proteins represent the most statistically significant cluster but other clusters were equally enriched (Fig. 2E). One interesting aspect revealed by the GO term enrichment analysis of the molecular functions of expressed genes was the preponderance of secreted cuticle proteins on V-0 compared to V-6 (Fig. 2F).

Among the 8362 genes, that had an rpgm value higher than the median (>0.0519) in our V-0 or V-6 samples and thereby considered as being expressed by the PG cells, we identified 71 cell membrane receptors (Fig. 3G and Supplementary Table S1 for detailed description), a 0.0085% of the expressed genes that is comparable to the percentage identified by our proteomic approach. To resolve whether this yield was caused by the 3.1 × 10^3^-fold coverage of the annotated transcriptome, we analysed the raw transcriptome dataset of a previous study in which the authors investigated the trascriptome profile of prothoracic glands and brain-corpora cardiac-corpora allata complexes from V-6 of the 5^th^ instar^6^ (Supplementary Table S4). By taking the median rpgm value (≈ 0.047 normalised reads per gene model length) (Supplementary Table S4) and comparing those datasets with our datasets, we detected the expression by the PG cells of 62 receptor genes (55 of them identified also in our dataset) while 69 receptor genes were identified as expressed in the brain-corpora cardiac-corpora allata complexes (Supplementary Table S4).

### Quantitative PCR analysis of cell membrane receptors in PG cells

**Figure 4:**
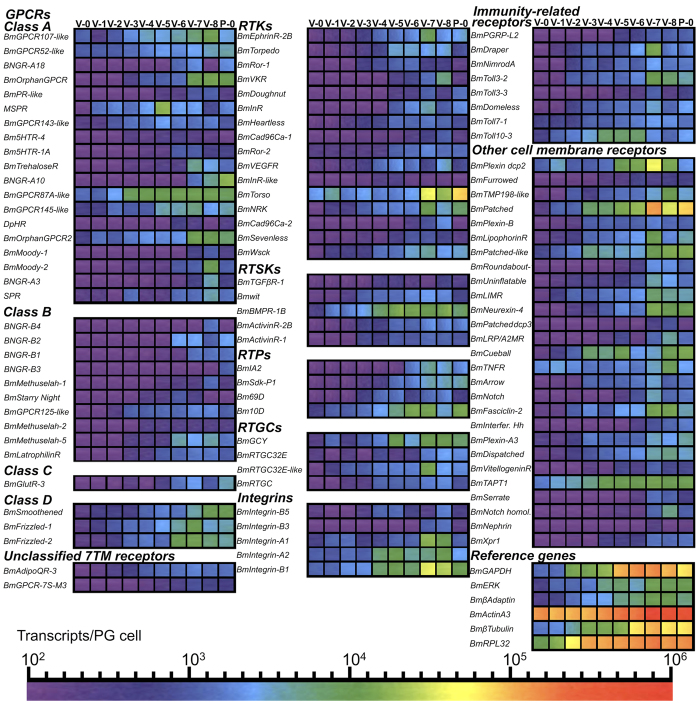
Expression profile heat maps of 104 receptor genes and the reference genes during the final larval stage and the first day of the pupal stage. Abbreviations: V-0 to V-8: Days of the 5^th^ Instar; P-0: First day of the pupal stage; Bm: *Bombyx mori*; GPCR: G protein-coupled receptor; BNGR: Bombyx neuropeptide G protein-coupled receptor; PR: prostanoid receptor; MSPR: Myosuppressin receptor; 5HTR: 5-hydroxytryptamine receptor; TrehaloseR: Trehalose receptor; DpHR: Diapause hormone receptor; SPR: Sex peptide receptor; GlutR-3: Glutamate receptor 3; AdipoQR-3: Adiponectin receptor 3; GPCR-7S-M3: G protein-coupled receptor-7 superfamily member 3; EphrinR-2B: Ephrin receptor type-2B; Ror: Receptor tyrosine kinase like orphan receptor; VKR: Venus kinase receptor; InR: Insulin receptor; Cad96Ca: Cadherin 96Ca; VEGFR1: Vascular endothelial growth factor receptor 1; NRK: Neurospecific receptor kinase; Wsck: Cell-wall integrity and stress-response component kinase; BmTGFBR-1: Transforming growth factor beta receptor 1; wit: Wishful thinking; BMPR1B: Bone morphogenetic protein receptor type-1B; ActivinR-2B: Activin Type 2B receptor; IA2: Receptor tyrosine phosphatase islet antigen 512; Sdk-P1: Sidekick protein 1; 69D: Receptor tyrosine phosphatase 69D; 10D: Receptor tyrosine phosphatase 10D; GCY: Guanylate cyclase; RTGC32E: Receptor type guanylate cyclase 32E; RTGC: Receptor type guanylate cyclase; PGRP-L2: Peptidoglycan recognition protein long 2; Plexin dcp2: Plexin domain-containing protein 2; TMP198: Transmembrane protein 198; LIMR: Lipocalin-1 interacting membrane receptor; Patched dcp3: Patched domain-containing protein 3; LRP/A2MR: Low-density lipoprotein receptor-related protein/Alpha-2 macroglobulin receptor; TNFR: Tumor necrosis factor receptor; I. hedgehog: Interference hedgehog; TAPT1: Transmembrane anterior posterior transformation protein 1; Xpr1: Xenotropic and polytropic murine leukemia virus receptor 1; GAPDH: Glyceraldehyde-3-phosphate dehydrogenase; ERK: Extracellular regulated MAP kinase, RPL32: Ribosomal Protein L32 (also known as RPL49).

**Figure 5:**
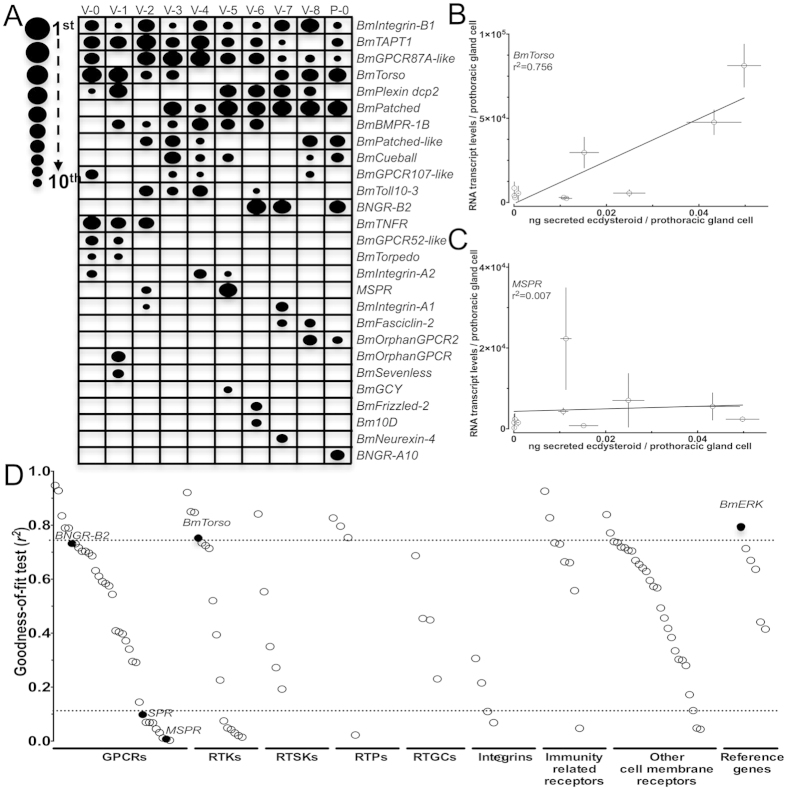
Evaluation of the most abundantly expressed receptor genes and correlation of the identified receptors with ecdysteroids secretion by the PGs. (**A**) Ranking maps of the 10 most abundantly expressed receptor genes on each day of the 5th instar and the first day of the pupal stage. (**B)** Correlation plot between transcript levels of *BmTorso* and ecdysteroids secretion by the PGs. (**C**) Correlation plot between transcript levels of *MSPR* and ecdysteroids secretion by the PGs. (**D**) Ranking of all the expressed receptors genes by the correlation (*r*2) of their transcript levels versus ecdysteroids secretion by the PGs.

To resolve the temporal expression profiles of the 369 receptor genes we identified by our bioinformatic analysis and rule out any possibility that receptors not expressed on day 0 or day 6 may be expressed on other days of the 5^th^ instar, we carried out quantitative PCR (qPCR) analysis of each of these genes on each day of the 5^th^ instar and the 1^st^ day of the pupal stage (Fig. 4, Supplementary Fig. 2 and Supplementary Table S7). Such analysis was also necessitated by the fact that both our proteomic and transcriptomic approach revealed some differences in the cell membrane receptors that are expressed in these two days (Supplementary Table S1). For our qPCR analysis we first determined the expression of reference genes that have been frequently used in other studies such as RPL32 (also known as RPL49), actin A3, GAPDH or β-tubulin^41^, extracellular regulated MAP kinase^20^, which is known to regulate ecdysteroidogenesis in PGs^1^ and β-adaptin a component of clathrin coated vesicles^42^. Our results showed that transcripts of such genes have widely varying patterns of expression throughout the developmental period of interest and they were therefore unsuitable for relative qPCR assays^41^ (Fig. 4). Remaining, therefore, sceptical about the use of relative quantification^41^ as a means of quantifying gene expression over a broad developmental period, we used an absolute quantification approach by calculating the expression levels of genes from a standard curve generated with *BmTorso*, the prothoracicotropic hormone receptor, which is known to be exclusively expressed in the prothoracic glands^35^. Such standard curve (C_*q*_ = −3.225*(log_10_template copies) + 35.25) (Supplementary Fig. 1) enabled us to express C*q* values of the cDNAs as transcript levels per cell since each PG contains a constant number of cells in the developmental period of interest. This approach yielded a total of 104 receptors that are expressed in PGs during this developmental stage (Fig. 4) with 35 of them showing detectable expression levels on days other than those used in our transcriptome analysis. The most abundantly expressed receptor genes were the *B. mori* homologue of the Hedgehog receptor *Patched*, a plexin domain-containing protein (*BmPlexin dcp2*) and *BmTorso* (Fig. 4 and Supplementary Figure 2). To provide biological relevance to this heat map (Fig. 4), we ranked the 10 most abundantly expressed receptor genes for each day of the developmental stage of interest and generated a ranking map (Fig. 5A) for each of the 27 receptor genes we identified as the highest expressing ones. Such ranking corroborated previous results on the expression of *BNGR-B2* at the end of 5^th^ instar^6^, on the critical role of *BmTorso* as a major regulator of the ecdysteroids peak at the end of the 5^th^ instar^35^ and on the critical role of myosuppressin receptor (*MSPR*) as a negative regulator of ecdysteroidogenesis^36^. Indeed, when transcript levels of *BmTorso* were plotted against ecdysteroids secretion by the PGs, a rather highly positive correlation was identified (Fig. 5B), while a similar analysis with *MSPR* showed no correlation at all (Fig. 5C). Because these 2 genes, a positive and a negative regulator of ecdysteroidogenesis respectively^35^,^36^, can be considered as the vertices of a broad spectrum within which several other genes can be identified as regulators of ecdysteroidogenesis, we plotted the transcript levels of each of the 104 genes we identified against ecdysteroids secretion (Fig. 1C) for each day of the developmental stage of interest and we visualised these data as a goodness-of-fit test (Fig. 5D and Supplementary Table S6). This test showed that 17 receptors had a highly positive correlation with ecdysteroids secretion while 22 receptors had a highly negative correlation (Fig. 5D and Supplementary Table S6). Although such analysis may just represent the ecdysteroids-mediated induction of expression of these receptors, the presence of all known positive regulators of ecdysteroidogenesis (*BNGR-B2, BmTorso* and *BmERK*) at the upper limit of the graph and the presence of all known negative regulators of ecdysteroidogenesis (*MSPR* and *SPR*) at the lower limit of the graph (Fig. 5D) suggests that such ranking may convey factual information about the role of these receptors. The majority of the 104 genes showed no correlation of their transcript levels with the ecdysteroidogenic activity of PGs, an indication that their levels are not mediated by ecdysteroids but rather by other cues.

## Discussion

The mechanisms underlying ecdysteroidogenesis in insects have been the subject of extensive research for decades. Through these decades of research our understanding of the biochemical and developmentally coordinated pattern of ecdysteroids synthesis and secretion has gone through substantial advances both at the level of the proteins that are responsible for converting cholesterol to ecdysteroids^2^ and at the level of receptors, signalling molecules and second messengers that participate in the finely-tuned synthesis of ecdysteroids^1^,^4^. Still, there exist less than a dozen known ligands and cell membrane receptors that are known to participate and shape the ecdysteroidogenic activity of these cells and although the list of known receptors is growing^5^,^6^,^7^, whether all these receptors participate in a combinatorial fashion and regulate exclusively the ecdysteroidogenic activity of these cells has not been determined yet.

To identify the molecular nature and variety of receptors that through their activation shape ecdysteroidogenesis in immature insect stages, we selected *B. mori* larvae of the final larval stage as our insect model. The size of prothoracic glands, the constant number of their cells during the final larval stage (Fig. 1A), and their structural integrity makes them a perfect sample for proteome and transcriptome analysis.

We identified 35 of the 119 GPCR genes in the completed *B. mori* genome database to be expressed by the PG cells during the developmental stage we investigated (Fig. 4). We confirmed the expression of all the GPCR genes (Supplementary Table S1 and Fig. 4) that were previously identified to be expressed in the prothoracic glands by other research groups^6^,^8^,^31^,^36^,^43^. By ranking the transcript abundance of each of the receptor genes (Fig. 5A), we found 9 GPCRs to be amongst the 10 most abundant receptor gene transcripts during the 5^th^ instar and early in the pupal stage. All but two of these genes (i.e. *BNGR-B2* and *MSPR*; Fig. 5A) are orphan receptors.

We mapped on *B. mori* chromosomes and scaffolds 64 odorant receptors^14^ and increased through bioinformatic analysis the number of gustatory receptor genes in the *B. mori* genome to 71^15^ but none of these receptors is expressed by the prothoracic gland cells (Supplementary Table S1). We identified a large repertoire of RTKs, RTSKs and RTPs (Fig. 4) expressed by the PG cells and some of these genes have been previously identified in the *D. melanogaster* ring gland^7^. Among them, we find the *B. mori* homologue of *D. melanogaster* gene *Torpedo* and *Sevenless* to be enriched (Table 5A) in transcript abundance early in the 5^th^ instar, while the prothoracicotropic hormone receptor *Torso* and the *B. mori* homologue of *D. melanogaster* gene *Thickveins* (*BmBMPR-1B*) exhibit opposing levels of transcript abundance (Fig. 5A). Receptor-type guanylate cyclases (RTGCs) have been identified as the receptors for the insect eclosion hormone^44^ but there has been no report on their expression in prothoracic gland cells. We find 4 of the 9 RTGCs identified in the *B. mori* genome to be expressed by the PG cells although their pattern of expression does not follow the ecdysteroidogenic activity of these cells (Fig. 4 and 5D). We identified receptors for several other signalling pathways such as the Wnt/Wingless signalling pathway, the Hedgehog signalling pathway^5^,^22^ with the *B. mori* homologue of *Patched*, the Hedgehog receptor, identified as the most abundantly expressed receptor gene after day 3, the day after the Head Critical Period (HCP) when neck ligation can prevent subsequent metamorphosis^45^. It is therefore relevant to our results (Fig. 5A), that Hedgehog has been identified as a metabolic hormone that inhibits ecdysteroids production by the ring gland and delays maturation in *D. melanogaster*^5^, acting as one among many limiting inputs that shape ecdysteroidogenesis and coordinate the development of insects.

Prothoracic gland cells do not divide and become very large^3^ before they initiate apoptosis. One of the signalling pathways that suppresses organ size and cell proliferation and promotes apoptosis is the Hippo signalling pathway^46^. The two receptors that activate the core Hippo signalling pathway in *D. melanogaster* are called Fat and Dachsous and they exert negative control on cell proliferation in a cell autonomous manner^46^. We were unable to identify the expression of *B. mori* homologues of *Fat* and *Dachsous* in PG cells (Supplementary Table S1) although we identified downstream components of this pathway both in our proteome data (Supplementary Table 2A,B) and our transcriptome data (NCBI Short Read Archive No. SRP062258). The regulatory mechanisms of the Hippo signalling pathway in PG cells deserves further investigation and perhaps these cells are ideal tools to understand how in the absence of these specific receptors they receive signals from their neighbouring cells or other tissues which tell them to continue grow or initiate apoptosis.

The presence of 8 innate immunity-related receptors in PG cells brings forth the intriguing possibility that the PGs have more than a single function in the insect body. We identified 4 Toll-like receptors to be expressed in PG cells (Fig. 4), out of the 13 Toll receptor genes present in the *B. mori* genome^18^. The most abundantly expressed among these receptors was *BmToll10-3* (Fig. 4). The levels of *BmToll10-3* ranked among the highest in PGs until day 6 (Fig. 5), the day these animals stop feeding, and rapidly declined thereafter (Fig. 4). Among the 22 immunity-related receptors we screened by qPCR, we identified one cytokine receptor, the *B. mori* homologue of *Domeless* as well as one microbial ligand receptor (*PGRP-L2*) and two phagocytosis receptors, the *B. mori* homologues of *Draper* and *NimrodA* (Fig. 4) to be expressed by the PG cells. The presence of such a specialised set of innate immunity-related receptors suggests that PG cells are involved in particular aspects of the defence response of insects. Both our transcriptomic (NCBI Short Read Archive No. SRP062258) and proteomic data (Supplementary Table 2A,B) showed that PG cells express several genes that code for secreted proteins, among them serpins^47^ and the *B. mori* homologue of *Spätzle*^18^ that participate in innate immunity responses^18^.

Pathogens such as baculoviruses^48^ or fungi^49^ can biochemically alter the ecdysteroids titre in insects while parasitism is just another example where changes in ecdysteroids titre of the host have been shown to be associated with the efficient survival and metamorphosis of the parasite in the insect body^50^. Intricate patterns of disruption or manipulation of host’s ecdysteroidogenesis by parasitoid wasps, their venomous secretions and co-inhabiting viruses has already been reported^1^,^50^, and in this context the PGs may be pivotal in decoding an ecdysteroidogenesis inhibiting signal from the parasitoid as the trigger to mount a generalised immune response against the invader. How extensive is the involvement of PGs in the innate immunity responses of insects certainly deserves a detailed examination and it would be particularly important to identify which of the functions of PG cells was the primordial one: Were (and still are) the PGs originally part of the immune system or part of the endocrine system? If the former is true, could it be that ecdysteroids were actually one component of the defence arsenal of primordial ecdysozoa, a form of defence by small molecule secretions that developed later into a morphogen responsible for the development of insects?

## Methods

### Animals

All experiments were conducted using *B. mori* larvae of the hybrid J106xDAIZO. Larvae were reared on fresh mulberry leaves under a 12:12-L:D photoperiod at 25 ± 1 °C and 60% relative humidity. Larvae were staged after every larval ecdysis, and the day of each ecdysis was designated as day 0. Since larvae mainly moult to the final (5^th^) instar during the scotophase, all larvae that ecdysed during the scotophase were segregated immediately after the onset of photophase. This time was designated as 0 h of the 5^th^ instar. In this particular hybrid, the 5^th^ instar period lasts about ~208 h. The onset of pupal commitment occurs after 60 h (day 3) and the onset of wandering behaviour occurs 144 h (day 6) after the final larval ecdysis. This hybrid has a short period of cocoon spinning that lasts ~38 h followed by a period of ~26 h before pupal metamorphosis. Each day of the 5^th^ (V) instar is designated with its numerical number (i.e.V-0, V-1 etc.) while the first day of the pupal stage is designated as P-0.

### *In vitro* PG assay and measurement of PG cells

Larvae were anaesthetised by submersion in water and PGs were dissected rapidly (~2 min/animal) from each larva in sterile saline (0.85% NaCl). The glands were pre-incubated in Grace’s medium (Invitrogen) for 15**–**30 minutes and then each gland was incubated for 2 h in 20 μl of Grace’s medium at 25 ± 1 °C and high humidity (90%) in a well of a 96-well micro plate (Greiner). Then, the amount of secreted ecdysteroids in the incubation medium was determined on an aliquot (2 μl) by enzyme immunoassay (ACE Enzyme Immunoassay; Cayman Chemical), using 20-Hydroxyecdysone (20E) EIA antiserum (Cayman Chemical). Calibration curves were generated using 20E (Cayman Chemical) and results were expressed as ng of 20E per gland.

The same isolation procedure as above was used to determine the number of PG cells throughout the 5^th^ instar and the first day of the pupal stage. In this case, pairs of PGs were briefly incubated separately and counts of PG cells were taken from each pair (n = 7) placed on 5 μl of 0.02% Trypan blue (Sigma) in phosphate buffered saline (pH = 7.4) under a cover slip. At this concentration, Trypan blue permeates slowly in PG cells and measurements can be accurately taken within 5 min for each gland.

### Bioinformatic screening of *B. mori* genome for cell membrane receptors

Candidate genes coding for GPCRs, RTKs, RTSKs, RTPs, RTGCs, Integrins, innate immunity-related receptors and other cell membrane receptors were identified using databases of *B. mori* genome annotations, other insect species as well as vertebrate species^12^,^51^, in several ways as queries to the *B. mori* genome. Initially, we employed reciprocal tBLASTΝ and the methodology used to identify GPCRs in *B. mori*^11^ and GPCRs, RTKs, RTSKs and RTGCs in other dipteran genomes^16^, but expanded our search to include odorant receptors^14^, gustatory receptors^15^, RTP homologues, *B. mori* integrins^17^, innate immunity-related receptors^18^ and other cell membrane receptors involved in signal transduction that mediate, the Wnt/Wingless signalling pathway, the Hedgehog signalling pathway^5^,^22^ the Notch signalling pathway^52^, the Roundabout signalling pathway^53^, the Hippo signalling pathway^46^ as well as other receptors involved in signal transduction^10^,^54^. Additional *B. mori* proteins were also retrieved using a combination of literature searches^11^,^14^,^15^,^18^,^31^,^55^ and available databases such as the NCBI RefSeq protein repository (http://www.ncbi.nlm.nih.gov/), the UniprotKB (http://www.uniprot.org/), the SilkDB database (http://silkworm.genomics.org.cn/) and tBLASTN queries against other available insect genomes to identify as many as possible cell membrane receptors. The KAIKOBLAST server (http://kaikoblast.dna.affrc.go.jp/) was used to search and annotate our lists of cell membrane receptors and tentative matches were aligned and checked using BLASTP, tBLASTN (http://blast.ddbj.nig.ac.jp/top-j.html) and the EST database in KAIKOBLAST for gene prediction errors. Protein sequences with *e*-values less than 0.1 were listed and assigned to classes and categories using the profile hidden Markov model on HMMER (http://www.ebi.ac.uk/Tools/hmmer/). The nucleotide and amino acid sequences and the position of the retrieved genes on chromosomes and scaffolds were determined on the KAIKOBLAST server and, when necessary, further manually curated based on previously published data. Additional domain analysis of the retrieved protein sequences was carried out by Pfam (http://www/sanger.ac.uk/Software/Pfam/), PROSITE (http://au.expasy.org/prosite/), SMART (http://smart.embl-heidelberg.de/) and InterPro (http://www.ebi.ac.uk/interpro/). Signal peptide and transmembrane domains were analysed by SignalP4.1 (http://www.cbs.dtu.dk/services/SignalP/) and TMHMM server v.2.0 (http://www.cbs.dtu.dk/services/TMHMM/).

### Proteomic analysis of *B. mori* PG cells

Prothoracic glands from day 0 and day 6 (onset of wandering stage) of the 5^th^ instar were used to analyse the protein expression profiles of *B. mori* PG cells. Larvae were anaesthetised by submersion in water and PGs were dissected rapidly (~2 min/animal) from each larva in sterile saline (0.85% NaCl). The glands were pre-incubated in Grace’s medium (Invitrogen) for 15**–**30 minutes and meticulously cleared of any associated tissue or debris. Then, glands were pooled and successively transferred to gradually diminishing volumes of Grace’s medium drops (n = 5) before being snap frozen in dry ice and stored at −80 ^o^C.

Each sample was dissolved in a buffer consisting of 6 M Urea, 2 M Thiourea, 4% (w/v) CHAPS, 5 mM Mg Acetate and 10 mM Tris (pH 8.5). Samples were vortexed and sonicated 4 times for 30 sec with 2 min breaks on ice between pulses. Samples were then centrifuged at 10000×*g* and the supernatant was collected for protein separation on a 1D gel. 40 μl of sample were mixed with 5× concentrated loading buffer with addition of fresh DDT (1%) before being heated for 5 minutes at 95 ^o^C, cooled down and loaded (50 μl) on a Mini-Protean TGX gel (4–15%) (Bio-Rad). The gel was then fixed for 30 minutes (45% methanol, 1% acetic acid) and stained with colloidal coomassie overnight.

Each lane was cut into 16 gel slices and each slice was transferred into a 96-well PCR plate. Gel bands were cut into 1 mm^2^ pieces, destained, reduced with DTT then alkylated with iodoacetamide and subjected to enzymatic digestion with trypsin overnight at 37 °C. After digestion, the supernatant was pipetted into a sample vial and loaded onto an auto-sampler for automated liquid chromatography-tandem mass spectrometry (LC-MS/MS) analysis.

All LC-MS/MS experiments were performed using a nanoAcquity UPLC (Waters Corp.) system and an LTQ Orbitrap Velos hybrid ion trap mass spectrometer (Thermo Scientific). Separation of peptides was performed by reverse-phase chromatography using a Waters reverse-phase nano column (BEH C18, 75 μm i.d. x 250 mm, 1.7 μm particle size) at flow rate of 300 nl/min. Peptides were initially loaded onto a pre-column (Waters UPLC Trap Symmetry C18, 180 μm i.d x 20 mm, 5 μm particle size) from the nanoAcquity sample manager with 0.1% formic acid for 3 minutes at a flow rate of 10 ml/min. After this period, the column valve was switched to allow the elution of peptides from the pre-column onto the analytical column. Solvent A was water + 0.1% formic acid and solvent B was acetonitrile + 0.1% formic acid. The linear gradient employed was 5–40% B in 60 minutes.

The LC eluant was sprayed into the mass spectrometer by means of a nanospray source (New Objective). All *m/z* values of eluting ions were measured in the Orbitrap Velos mass analyzer, set at a resolution of 30000. Data dependent scans (Top 20) were employed to automatically isolate and generate fragment ions by collision-induced dissociation in the linear ion trap, resulting in the generation of MS/MS spectra. Ions with charge states of 2 + and above were selected for fragmentation. Post-run, data was processed using Protein Discoverer (version 1.4., Thermo Scientific). Briefly, all MS/MS data were converted to mgf files and sixteen files (representing the entire gel lanes per sample) were combined and submitted to the Mascot search algorithm (v2.3.02, Matrix Science, London UK) and searched against the UniProt *B. mori* sequence database (14788 sequences). The mass spectrometry proteomics data have been deposited to the ProteomeXchange Consortium^56^ via the PRIDE partner repository with the dataset identifier PXD002771 and 10.6019/PXD002771. Our final analysis integrated previously reported proteomic data from PGs of day 4, 5^th^ instar *B. mori* larva^37^ in an attempt to identify additional proteins not identified by our method. Comparisons with that dataset^37^ were generated using the BLASTP algorithm on Metazome v3.0 (http://www.metazome.net/search.php?show=blast&method=Org_Bmori) and are reported on Supplementary Table S3.

### Transcriptome analysis of *B. mori* PG cells

Prothoracic glands from day 0 and day 6 (onset of wandering stage) of the 5^th^ instar were isolated as described above, meticulously cleared of any associated tissue or debris and total RNA was isolated immediately upon gland removal with TRIzol (Invitrogen) according to manufacturer’s instructions. The lllumina^®^ mRNA-Seq Sample Prep Kit was used to process the samples according to manufacturer’s instructions (1004898 Rev.D). Briefly, mRNA was isolated from total RNA using oligo-dT magnetic beads. After fragmentation of the mRNA, cDNA synthesis was performed and the resulting cDNA was ligated with the sequencing adapters and amplified by PCR. Quality and yield after sample preparation was measured with the Agilent 2100 Bioanalyzer (Agilent Technologies). The size of the resulting products was consistent with the expected size distribution with a broad peak between 200–500 bp on a DNA 1000 chip. A concentration of 17 pM of DNA was used for clustering and DNA sequencing on lllumina cBot and HiSeq2500 (HCS v2.2.58 software) according to manufacturer’s protocols.

### The *B. mori* reference genome

Τo construct a *B. mori* reference genome for subsequent use with short read alignment software, the part of the *B. mori* genome that is assembled in scaffolds and anchored to chromosomes was retrieved from the public data repository (http://sgp.dna.affrc.go.jp/pubdata/genomicsequences.html) of KAIKObase (http://sgp.dna.affrc.go.jp/KAIKObase). Then, *B. mori* genome contigs assembled to scaffolds but not anchored to chromosomes were also retrieved and from these scaffolds those that were less than 20 kb in length were excluded. These two parts of the *B. mori* genome were merged to a final FASTA file with chromosome and scaffold sequences. From this file, a Bowtie2 (http://bowtie-bio.sourceforge.net/bowtie2/index.shtml) index was constructed for subsequent use with TopHat2^57^ and Bowtie2 aligners. Comprehensive gene sets were retrieved again from KAIKObase (http://sgp.dna.affrc.go.jp/ComprehensiveGeneSet/) and a similar procedure as described above was followed to construct a gene file containing genes anchored to chromosomes and additional genes which were inferred in the scaffold sequences so as to construct a final *B. mori* gene file in GTF format to supply it to the TopHat2 aligner. From the final GTF file, BED files suitable for visualization in the University of California, Santa Cruz (UCSC) Genome Browser (https://genome.ucsc.edu/) were also constructed.

### Short read mapping

Image analysis, base calling, and quality check was performed with the lllumina data analysis pipeline RTA v1.18.64 and Bcl2fastq v1.8.4. An average of 13.26 Gb for day 0 (V-0) samples (n = 3) and 13.69 Gb for day 6 (V-6) samples (n = 3) were read and clusters passing lllumina filters^58^ were 90.6% while percentage of bases with Q-score ≥ 30 were 89.36%. The resulting FASTQ files containing pair-end 125 bp sequence reads were subjected to quality control using the FastQC package and mapped on the reference genome using TopHat2, with the standard parameters for reads obtained with Illumina platforms. Minor adjustments to the parameters were as follows: i) the *GTF* parameter was supplied with additional transcript annotation data for the *B. mori* genome as described in the previous section. ii) The *mate-inner-dist* and *mate-std-dev* parameters which are crucial for paired-end reads were estimated from the Bioanalyzer reports provided by the sequencing for each sample, iii) *read-gap-length* and *read-edit-dist* were set to 3. Upon completion of the first round of spliced alignment with TopHat2, the remaining unmapped reads were converted back to FASTQ using the *bedtools bamtofastq* command from the BEDTools (https://github.com/arq5x/bedtools2) suite to perform a second round of unspliced alignment with Bowtie2 in local, very sensitive mode to examine more carefully the unmapped reads. The Bowtie2 options used were *local, very-sensitive-local, maxins 1000, -dovetail*. For visualization purposes, the resulting BAM files were converted to the UCSC Genome Browser BED format, using the *bedtools bamtobed* command from BEDTools suite with the *-split* option in order to report RNA-Seq reads split by the TopHat2 algorithm as separate alignments. The BED files were then converted to BedGraph format (http://genome.ucsc.edu/goldenPath/help/bedgraph.html) using the *bedtools genomecov* command from the BEDTools suite with the *-bg* option and then to bigWig format (http://genome.ucsc.edu/goldenPath/help/bigWig.html) using the *bedGraphToBigWig* program supplied by UCSC. The bigWig tracks were visualised in a custom UCSC Genome Browser^59^ track hub hosting the *B. mori* reference genome (http://epigenomics.fleming.gr/tracks/hs_trackhubs/ekpa_dedos_2/hub.txt) and the RNA-Seq samples that were normalised to a total wiggle signal of 10^10^. Our final analysis integrated previously reported transcriptome data from PG and brain samples from day 6, 5^th^ instar *B. mori* larva^6^ in an attempt to corroborate our transcriptome analysis. Analysis of that data was carried out as described above and it is reported in Supplementary Table S4. The raw reads of our transcriptome data have been deposited into the NCBI Short Read Archive (SRA, http://www.ncbi.nlm.nih.gov/sra/) under accession number SRP062258.

### Total RNA isolation and cDNA synthesis from prothoracic glands

Prothoracic gland isolation was carried out as described above, glands were meticulously cleared of any associated tissue or debris and total RNA was isolated immediately with TRIzol (Invitrogen) according to manufacturer’s instructions (n = 7 for each day of the investigated developmental stage). Integrity of total RNA from each sample was determined using gel electrophoresis and RNA quality was determined by measuring the absorbance at 260 and 280 nm (A260/280 of all samples > 1.9). First strand cDNA was synthesise from 2 μg total RNA with 200 U Superscript^®^III reverse transcriptase (Invitrogen) in 20 μl reaction volumes using an oligo(dT)_20_ primer (Invitrogen) according to manufacturer’s instructions. The resulting cDNA was diluted with nuclease-free water (Invitrogen) before use in quantitative PCR.

### Quantitative PCR analysis of *B. mori* genes

Quantitative real-time PCR was carried out with the SYBR^®^ Green dye in 96-well PCR micro plates (Applied Biosystems) on a 7500 Real-Time PCR System (Applied Biosystems). Fluorescence emission of the products and subsequent calculations were carried out with the Sequence Detection System software v2.0.6 (Applied Biosystems). The reaction mixture (10 μl total volume per well) included 20 ng cDNA, 0.8 μl nuclease-free water (Invitrogen), 5 μl Kapa SYBR^®^ Fast Universal 2X qPCR Master Mix (Kapa Biosystems), 0.2 μl of 50 × Rox Low passive reference dye (Kapa Biosystems) and primers at a final concentration of 200 nmol/l. Reactions (n = 3–7) to amplify 230–270 bp amplicons were performed under the following conditions: 95 °C for 3 min as an initial step followed by 40 cycles of 95 °C for 15 s and 60 °C for 60 s. After amplification, dissociation curves were produced (60 °C–95 °C at a heating rate of 0.1 °C/sec and acquiring fluorescence data every 0.3 °C) to discriminate the main reaction products from other nonspecific ones or primerdimers and PCR products were subjected to electrophoresis on 2% w/v agarose gels to corroborate the presence of a unique amplicon (see Supplementary Table S7 for detailed description). Each qPCR run always included a no-cDNA template control and reverse transcription negative controls. For each of the replicates (n = 7) genomic DNA as a qPCR template was also analysed. All the aforementioned negative controls gave no detectable quantification cycle (*C*_*q*_) value, proving the lack of any contamination or nonspecific signal. Absolute quantification analysis via a standard curve approach was utilised to calculate the transcript levels of 339 of the 369 genes (see legend of Supplementary Table S1). Serial dilutions (4 to 4 × 10^6^ copies/reaction) of known concentration of *B. mori Torso* (*BmTorso*) in plasmid *pBRAcPA*^35^ (a generous gift from Dr. Michael O’Connor, University of Minnesota, USA), linearised with *NheI*, was used in each qPCR run to construct the standard curve(s). Concentration of the standard solutions of the plasmid was determined by spectrophotometry and was converted to copy numbers per microliter using the following formula: Copy number/μl = 6.023 × 10^23^ copies/mol × DNA concentration (g/μl)/molecular weight (g/mol). Standard curves were constructed by plotting the threshold cycle (C*q*; ΔRn = 0.25) values against the initial copy number of *BmTorso* containing plasmid. Copy numbers of transcripts in the samples were calculated by interpolating the C*q* value of the sample within the generated standard curve(s). We conducted validation experiments to test the requirements for applying the above methodology. We estimated reaction efficiency (*E*) with the formula, *E*% = [−1 + 10^(−1/slope)^] × 100 using dilution series of sample cDNA, incorporating several orders of magnitude (100–0.1 ng) and *C*_*q*_ values were plotted against log_10_[cDNA quantity]. The primers were designed using an online tool (http://primer3plus.com/cgi-bin/dev/primer3plus.cgi) with custom settings and the primers used for qPCR are listed in Supplementary Table S5.

### Statistical analysis

For the derivation of differentially expressed gene lists, the Bioconductor package^60^ metaseqR was used with the PANDORA^39^ method which improves overall accuracy and also the trade-off between true positives and false hits. The resulting RNA-Seq BAM files were analysed with the Bioconductor package metaseqR. Briefly, the raw BAM files, one for each RNA-Seq sample, were summarised to an exon read counts table, using the Bioconductor package GenomicRanges (http://bioconductor.org/packages/release/bioc/html/GenomicRanges.html) and the *B. mori* genes derived as described above. The gene read counts table was normalised using the Bioconductor package DESeq after removing genes that had zero counts over all the RNA-Seq samples. The output of the normalization algorithm was a table with normalised counts, which was used for differential expression analysis with statistical algorithms developed specifically for count data. Prior to the statistical testing procedure, the gene read counts were filtered for possible artifacts that could affect the subsequent statistical testing procedures. Genes presenting any of the following were excluded from further analysis: i) genes whose average reads per 100 bp was less than the 25^th^ quantile of the total normalised distribution of average reads per 100 bp, ii) genes with read counts below the median read counts of the total normalised count distribution. The resulting gene counts table was subjected to differential expression analysis for the contrasts day 6 (V-6) versus day 0 (V-0), or brain versus prothoracic gland^6^, using PANDORA^39^ (Supplementary Tables 1 and 4). For Gene Ontology (GO) analysis we used term description and hierarchical ranking provided by the Gene Ontology Consortium (http://amigo2.berkeleybop.org/amigo/landing). GO term enrichment analysis was carried out using g:Profiler^34^ with *B. mori* as the organism and default settings.

## Additional Information

**How to cite this article**: Alexandratos, A. *et al.* Reassessing ecdysteroidogenic cells from the cell membrane receptors’ perspective. *Sci. Rep.* **6**, 20229; doi: 10.1038/srep20229 (2016).

## Supplementary Material

Supplementary Information

Supplementary Table S1

Supplementary Table S2A

Supplementary Table S2B

Supplementary Table S3

Supplementary Table S4

Supplementary Table S5

Supplementary Table S6

Supplementary Table S7

## Figures and Tables

**Table 1:** *B. mori* cell membrane receptors.

G protein-coupled receptors (GPCRs)	
Class A: Rhodopsin-like	
Biogenic amine	20
Glycoprotein hormone/LGRs	1
Peptide	44
(Rhod)opsin	6
Purine	1
Orphan	14
Class B: Secretin-like	
Peptide	5
Adhesion GPCR	3
Methuselah-like	5
Latrophilin	1
Orphan	2
Class C: Metabotropic glutamate-like	
Metabotropic glutamate	3
GABA_B_	3
Orphan	3
Class D: Atypical GPCRs	
Frizzled	4
Smoothened	1
Unclassified Seven-transmembrane (7TM) Receptors	3
Chemosensory Receptors	
Odorant receptors	64
Gustatory receptors	71
Receptor Tyrosine Kinases (RTKs)	20
Receptor Serine/Threonine Kinases (RTSKs)	5
Receptor Tyrosine Phosphatases (RTPs)	5
Receptor Type Guanylate Kinases (RTGCs)	9
Integrins	11
Immunity related receptors	22
Other cell membrane receptors	43

The number of receptors predicted in each category, class and family is shown. Classification is according to^12^,^16^,^51^.
