# Supplementary material for: Reassessing ecdysteroidogenic cells from the cell membrane receptors’ perspective
Source: Sci Rep. 2016 Feb 5;6:20229. doi: 10.1038/srep20229 (PMC4742824; doi:10.1038/srep20229)
Supplement: Supplementary Information [file srep20229-s1.pdf]

Reassessing ecdysteroidogenic cells from the cell membrane receptors' perspective

Alexandros Alexandratos, Panagiotis Moulos, Ioannis Nellas, Konstantinos Mavridis, Skarlatos G. Dedos

Supplementary Figure Legends

Supplementary Figure 1: Quantitative PCR standard curve generated using as template known concentrations of the open reading frame of *B. mori Torso* (*BmTorso*) in plasmid *pBRAcPA* [35].

Supplementary Figure 2: Full list of figures showing transcript levels per prothoracic gland cell during the 5th instar and the first day of the pupal stage of the 104 receptor genes and reference genes identified by qPCR. These data were used to generate the heat maps shown in Figure 4.

Supplementary Table Legends

Supplementary Table S1: Index of the 369 genes that encode cell membrane receptors in *B. mori*. The genes are grouped according to the various classes and types of receptors and listed according to their chromosomal location. The index is annotated with our results from proteomic, transcriptomic and qPCR data analysis. *Reads outside the exons boundaries, ** Due to sequence similarity, specific primers could not be designed/transcript incompatible with qPCR analysis, ***Pseudogene

Supplementary Table S2:

A: Index of proteins identified in prothoracic glands on V-0 of the 5th instar after *de novo* analysis of our original data (dataset identifier PXD002771 and 10.6019/PXD002771).

B: Index of proteins identified in prothoracic glands on V-6 of the 5th instar after *de novo* analysis of our original data (dataset identifier PXD002771 and 10.6019/PXD002771).

Supplementary Table S3: Analysis of previously reported proteome data from PGs [37] and comparison index of the receptors identified by our proteomic analysis (Supplementary Tables 2A and 2B) with those identified by the previously reported proteome dataset [37].

Supplementary Table S4: Analysis of previously reported transcriptome data from prothoracic glands and brain-corpora cardiac-corpora allata [6] using the PANDORA method [39] and omparison index between the two datasets and the 369 genes that encode cell membrane receptors in *B. mori*. The genes are grouped according to the various classes and types of receptors and listed according to their chromosomal location. The index is annotated with brief description of our results from proteomic, transcriptomic and qPCR data analysis (see Supplementary Table S1 for full description) and the analysis of the previously reported dataset with the PANDORA method [39]. *Reads outside the exons boundaries, ** Due to sequence similarity, specific primers could not be designed/transcript incompatible with qPCR analysis, ***Pseudogene

Supplementary Table S5: List of qPCR primers used in this study.

Supplementary Table S6: Index of correlation results (Goodness-of-fit test) between transcript levels of the 104 receptor genes and reference genes, identified by qPCR (see text and Figure 5D for details), and ecdysteroids secretion by the PGs during the 5th instar and the first day of the pupal stage.

Supplementary Table S7: Checklist of compliance with MIQE guidelines [58].

Reassessing ecdysteroidogenic cells from the cell membrane receptors' perspective

Alexandros Alexandratos, Panagiotis Moulos, Ioannis Nellas, Konstantinos Mavridis, Skarlatos G. Dedos

Supplementary Figure 1:

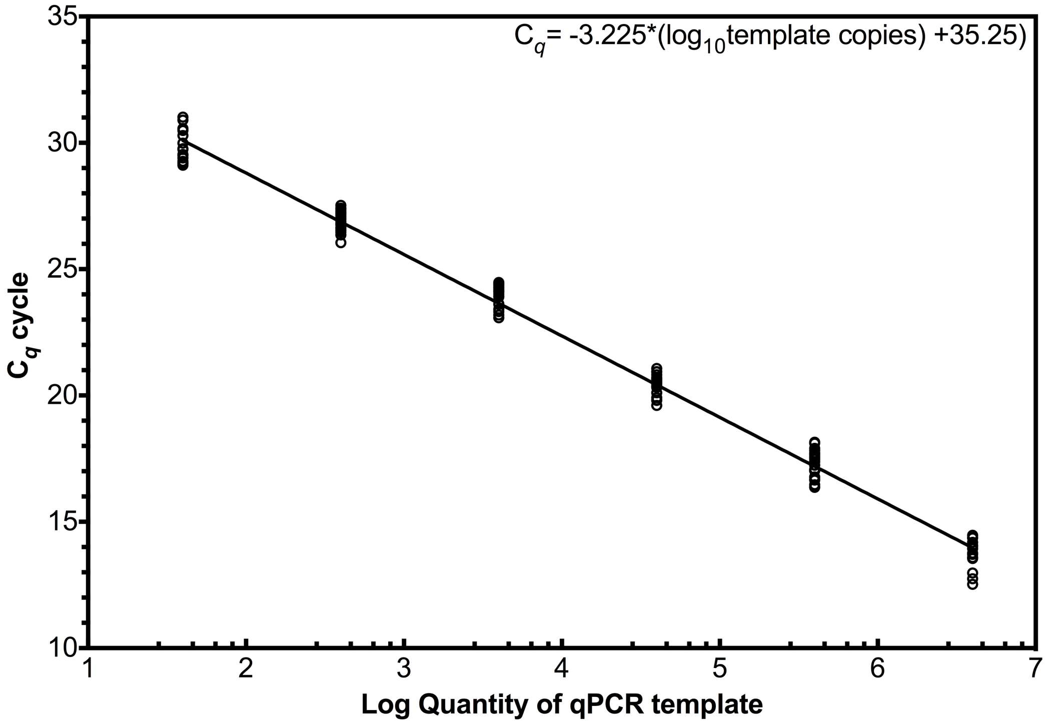

Reassessing ecdysteroidogenic cells from the cell membrane receptors' perspective

Alexandros Alexandratos, Panagiotis Moulos, Ioannis Nellas, Konstantinos Mavridis, Skarlatos G. Dedos

Supplementary Figure 2:

Class A

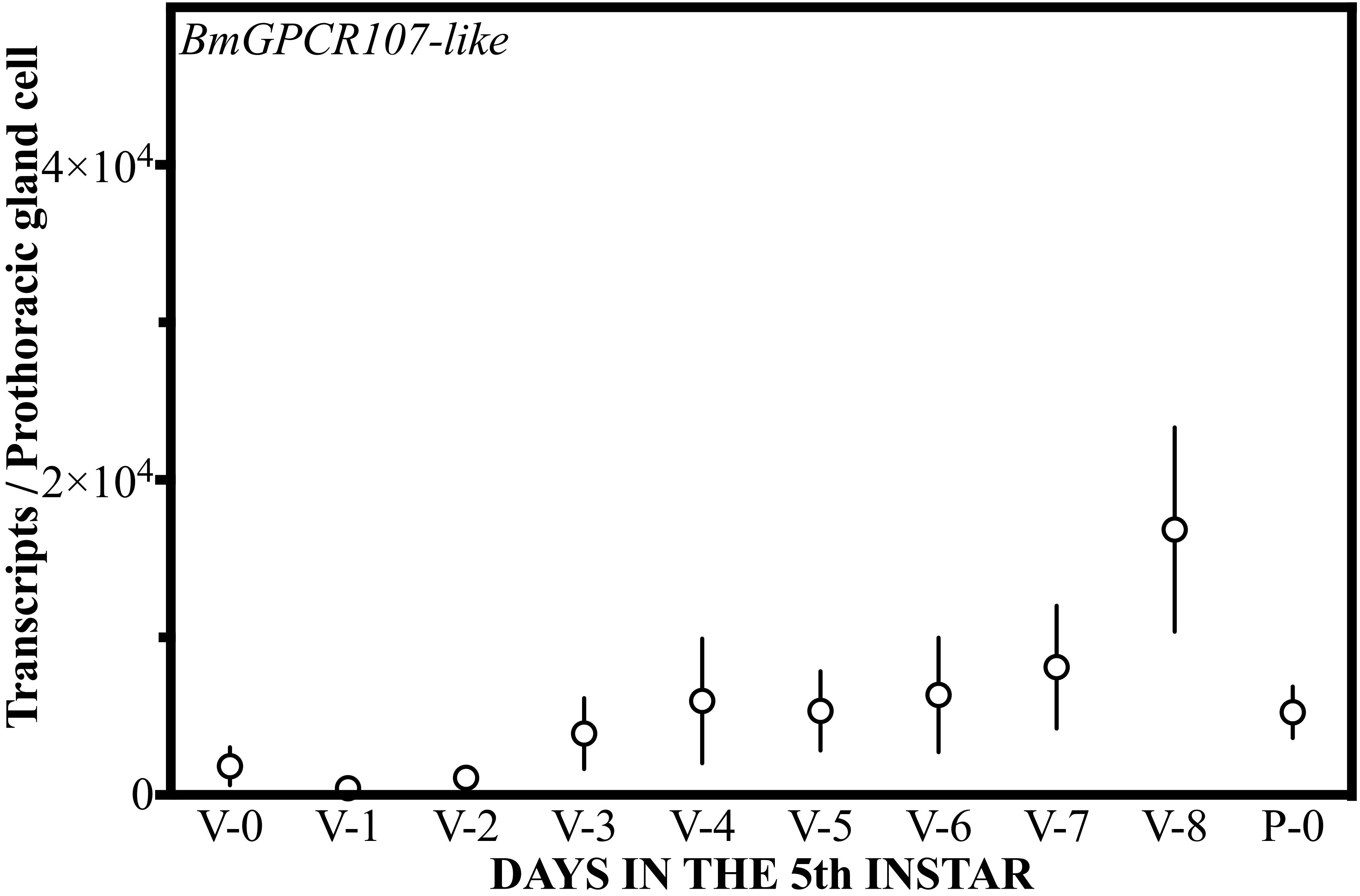

BmGPCR107-like
Transcripts / Prothoracic gland cell
4×10⁴
2×10⁴
0
V-0
V-1
V-2
V-3
V-4
V-5
V-6
V-7
V-8
P-0
DAYS IN THE 5th INSTAR

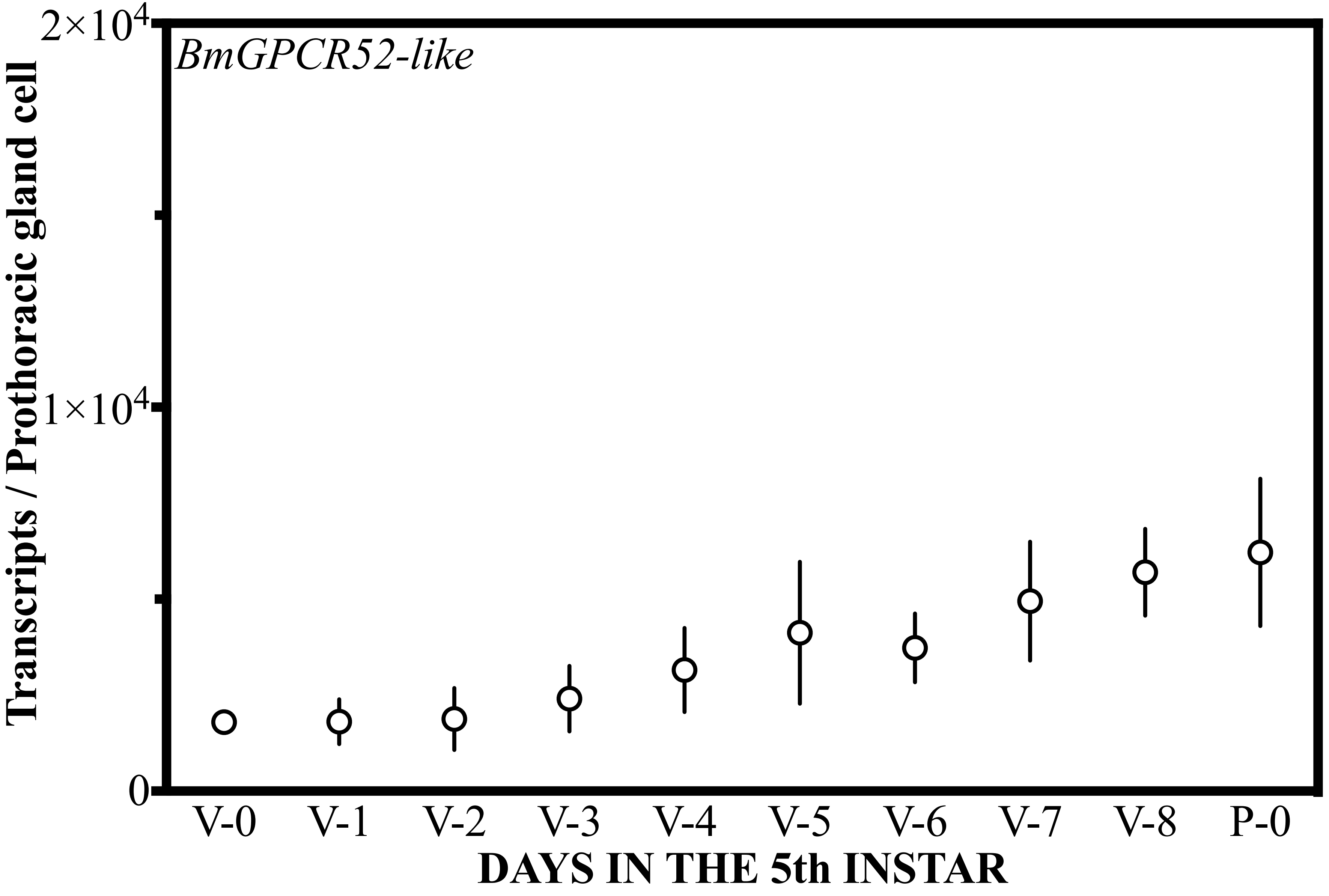

BmGPCR52-like
Transcripts / Prothoracic gland cell
2×10^4
1×10^4
0
V-0
V-1
V-2
V-3
V-4
V-5
V-6
V-7
V-8
P-0
DAYS IN THE 5th INSTAR

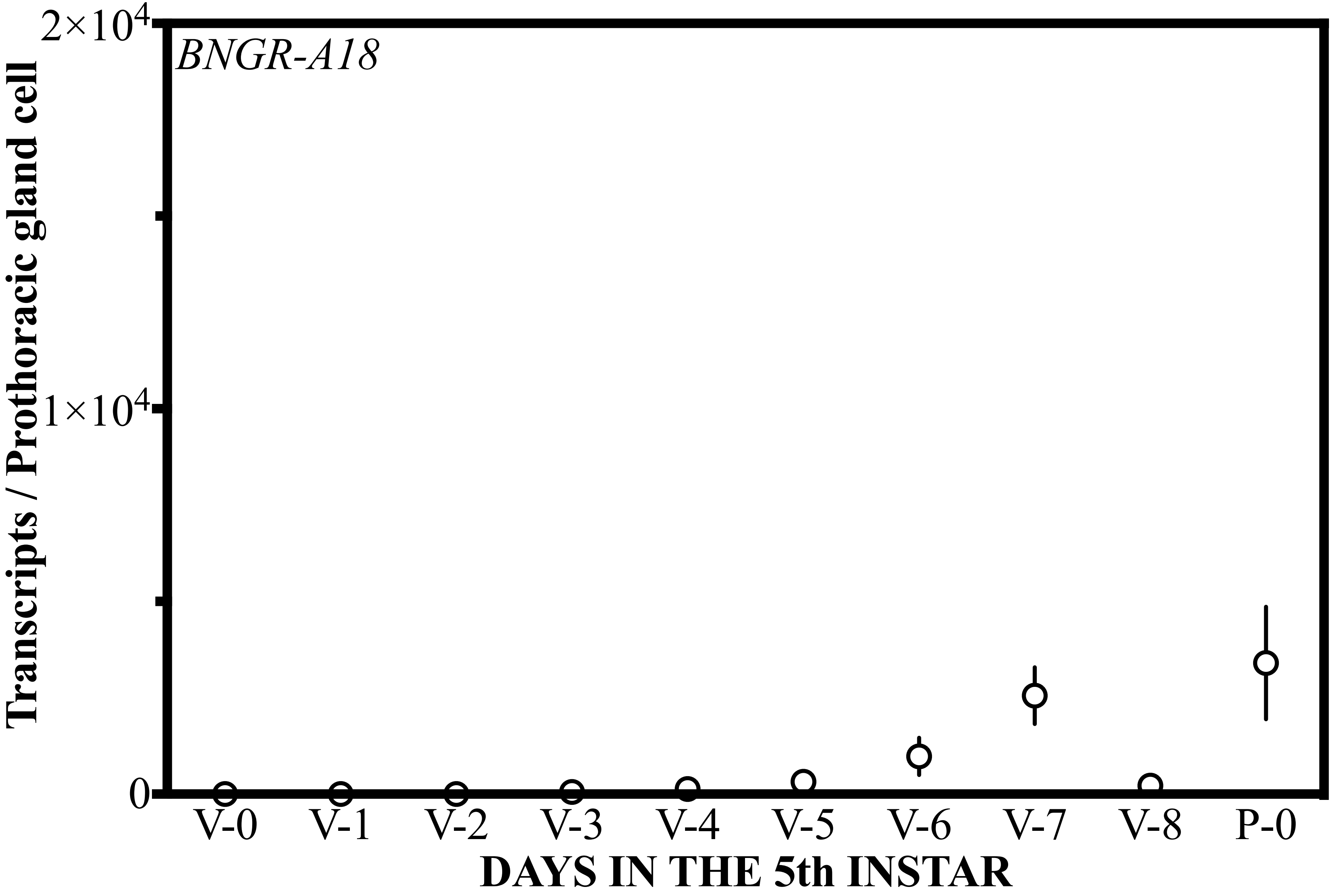
BNGR-A18
2×10^4
1×10^4
0
Transcripts / Prothoracic gland cell
V-0
V-1
V-2
V-3
V-4
V-5
V-6
V-7
V-8
P-0
DAYS IN THE 5th INSTAR

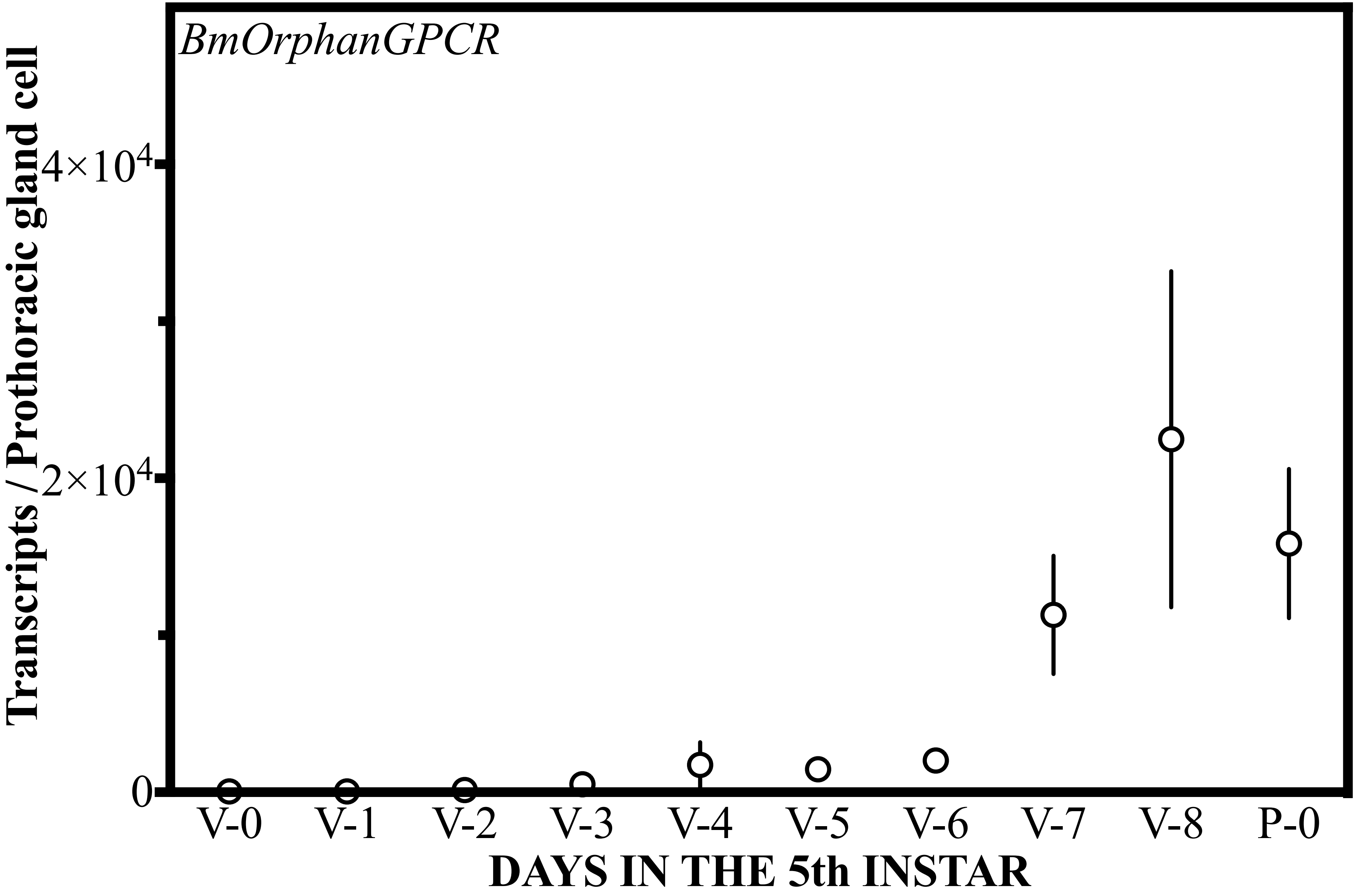
BmOrphanGPCR
Transcripts / Prothoracic gland cell
4×10^4
2×10^4
0
V-0
V-1
V-2
V-3
V-4
V-5
V-6
V-7
V-8
P-0
DAYS IN THE 5th INSTAR

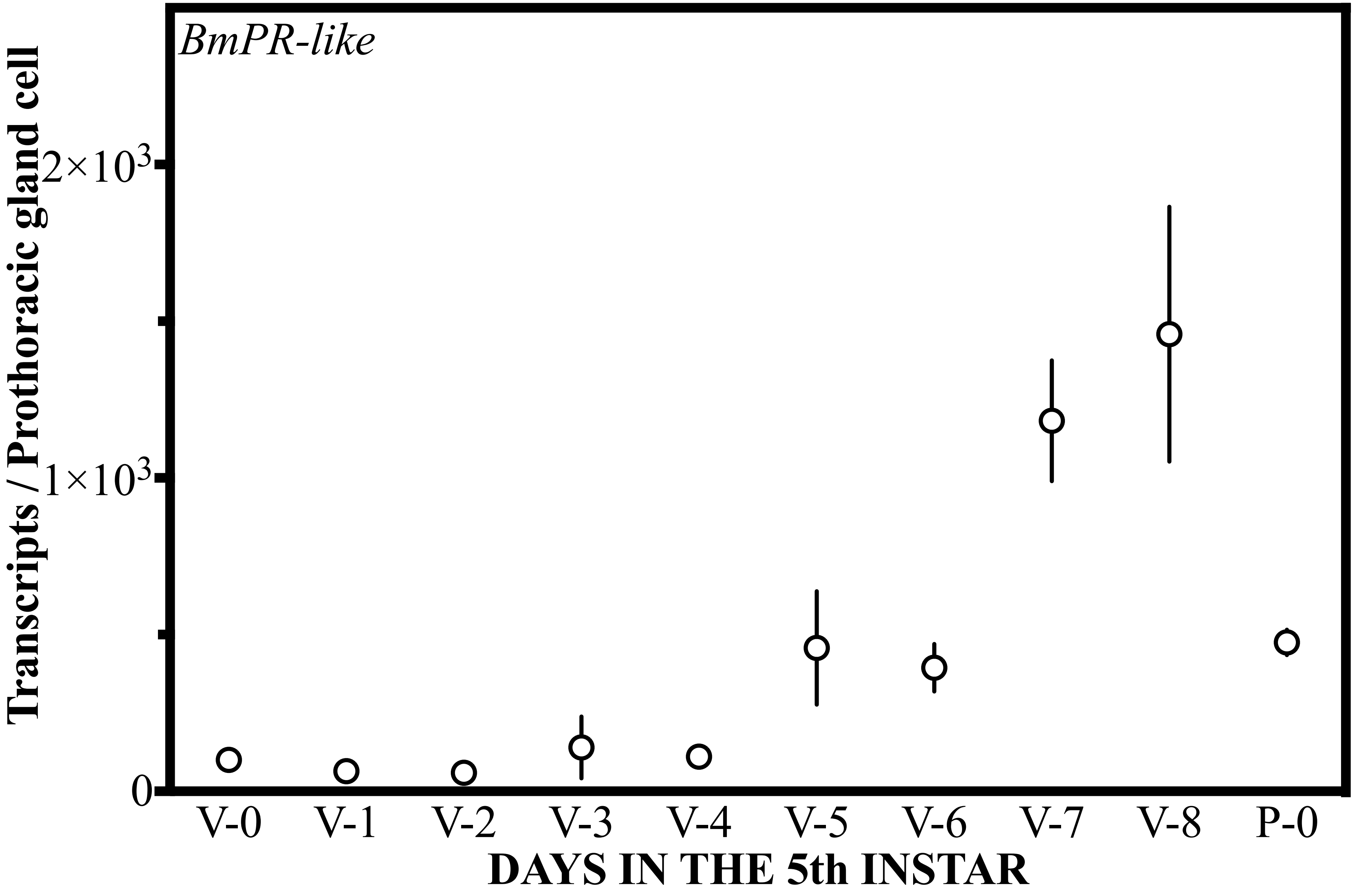

BmPR-like
Transcripts / Prothoracic gland cell
2×10^3
1×10^3
0
V-0
V-1
V-2
V-3
V-4
V-5
V-6
V-7
V-8
P-0
DAYS IN THE 5th INSTAR

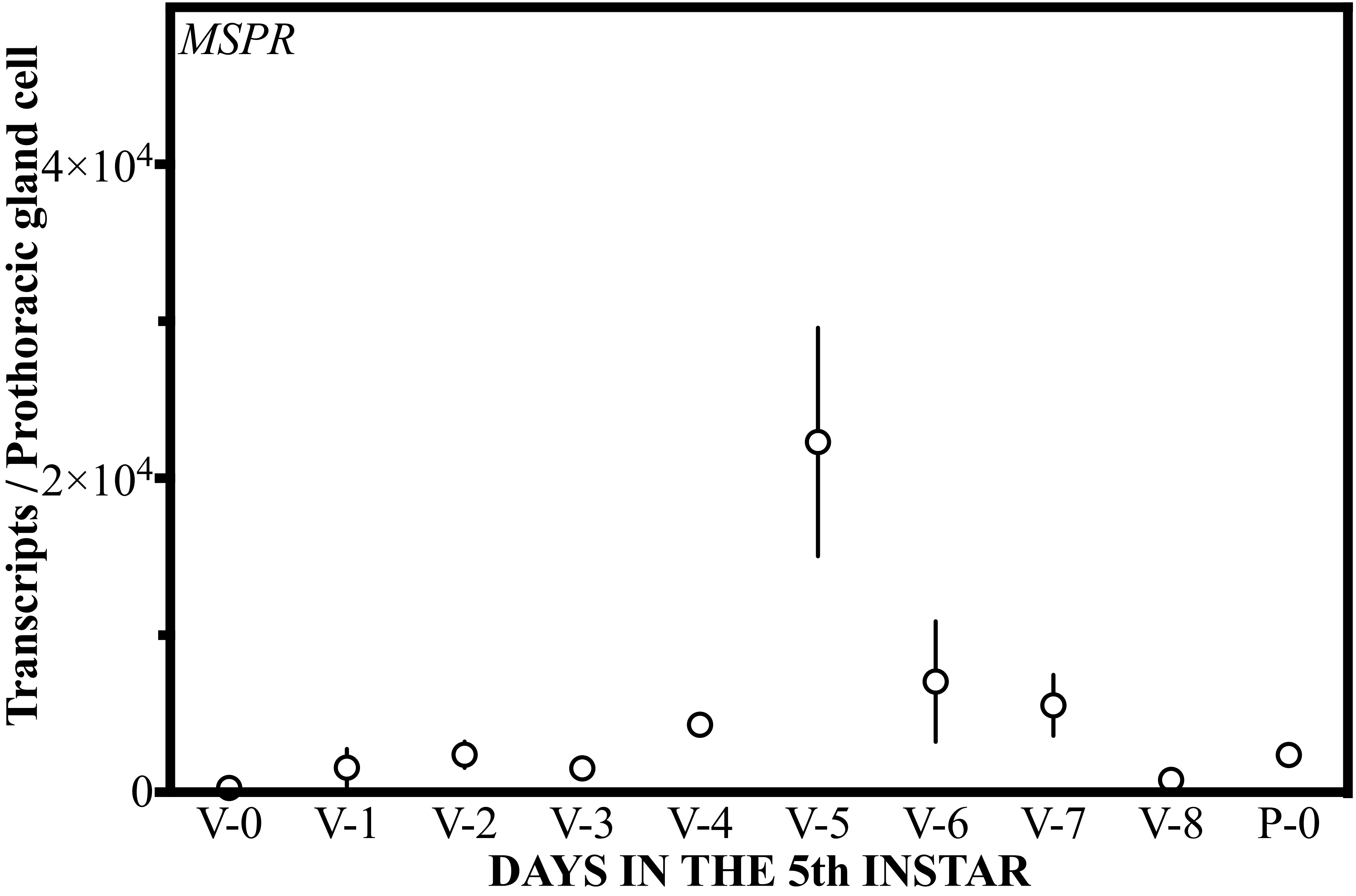

MSPR
Transcripts / Prothoracic gland cell
4×10^4
2×10^4
0
V-0
V-1
V-2
V-3
V-4
V-5
V-6
V-7
V-8
P-0
DAYS IN THE 5th INSTAR

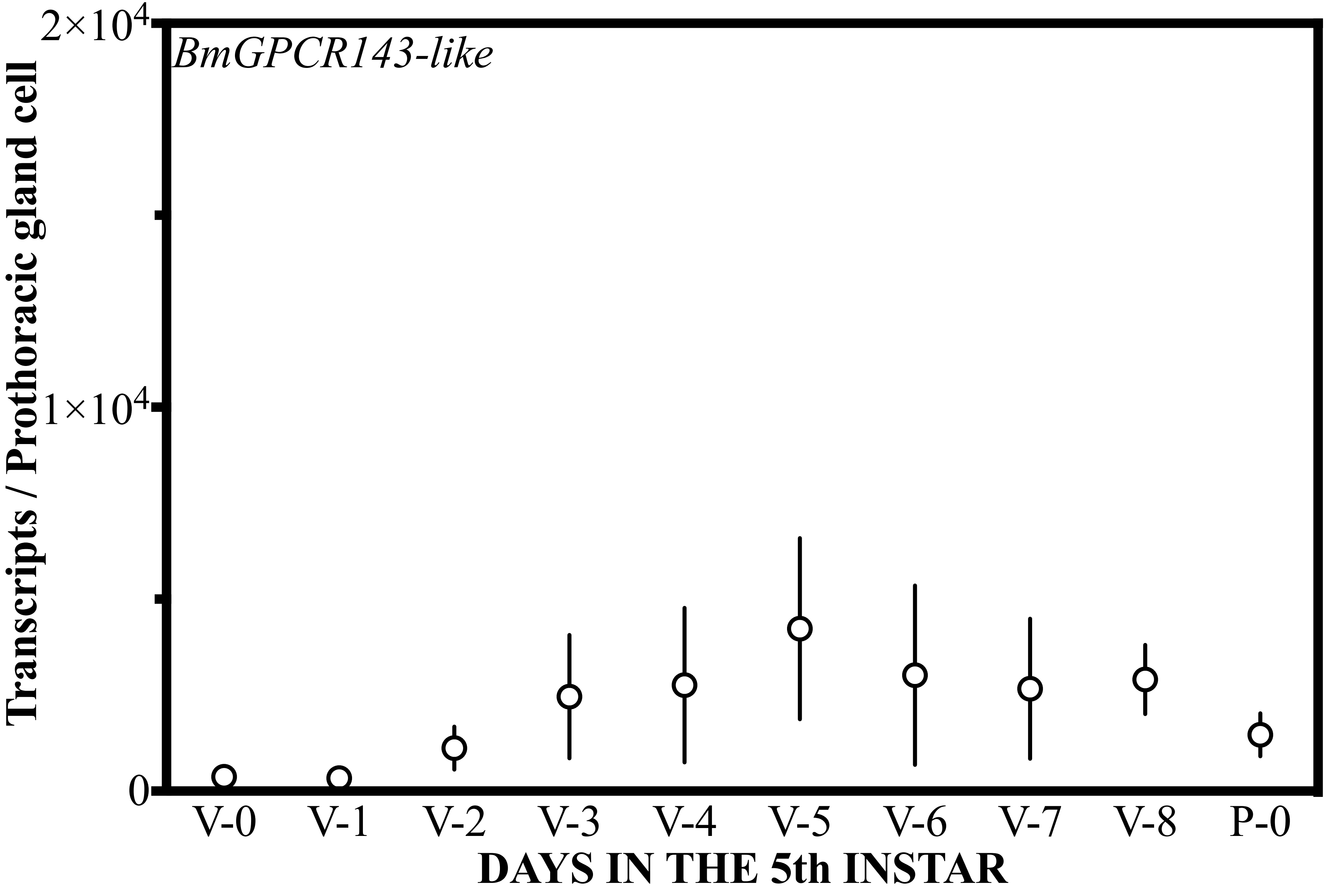

BmGPCR143-like
Transcripts / Prothoracic gland cell
2×10^4
1×10^4
0
V-0
V-1
V-2
V-3
V-4
V-5
V-6
V-7
V-8
P-0
DAYS IN THE 5th INSTAR

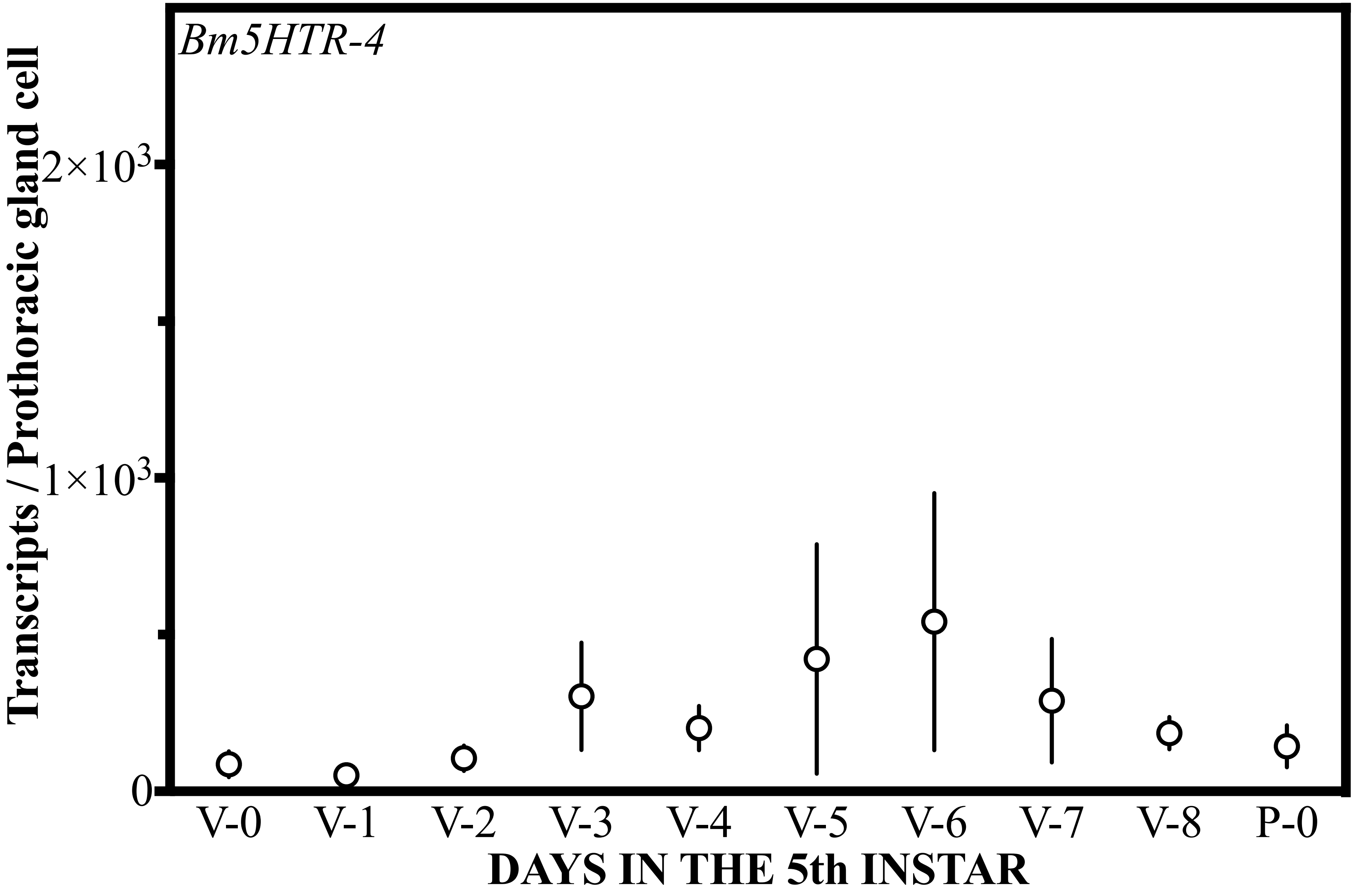
Bm5HTR-4
Transcripts / Prothoracic gland cell
2×10³
1×10³
0
V-0
V-1
V-2
V-3
V-4
V-5
V-6
V-7
V-8
P-0
DAYS IN THE 5th INSTAR

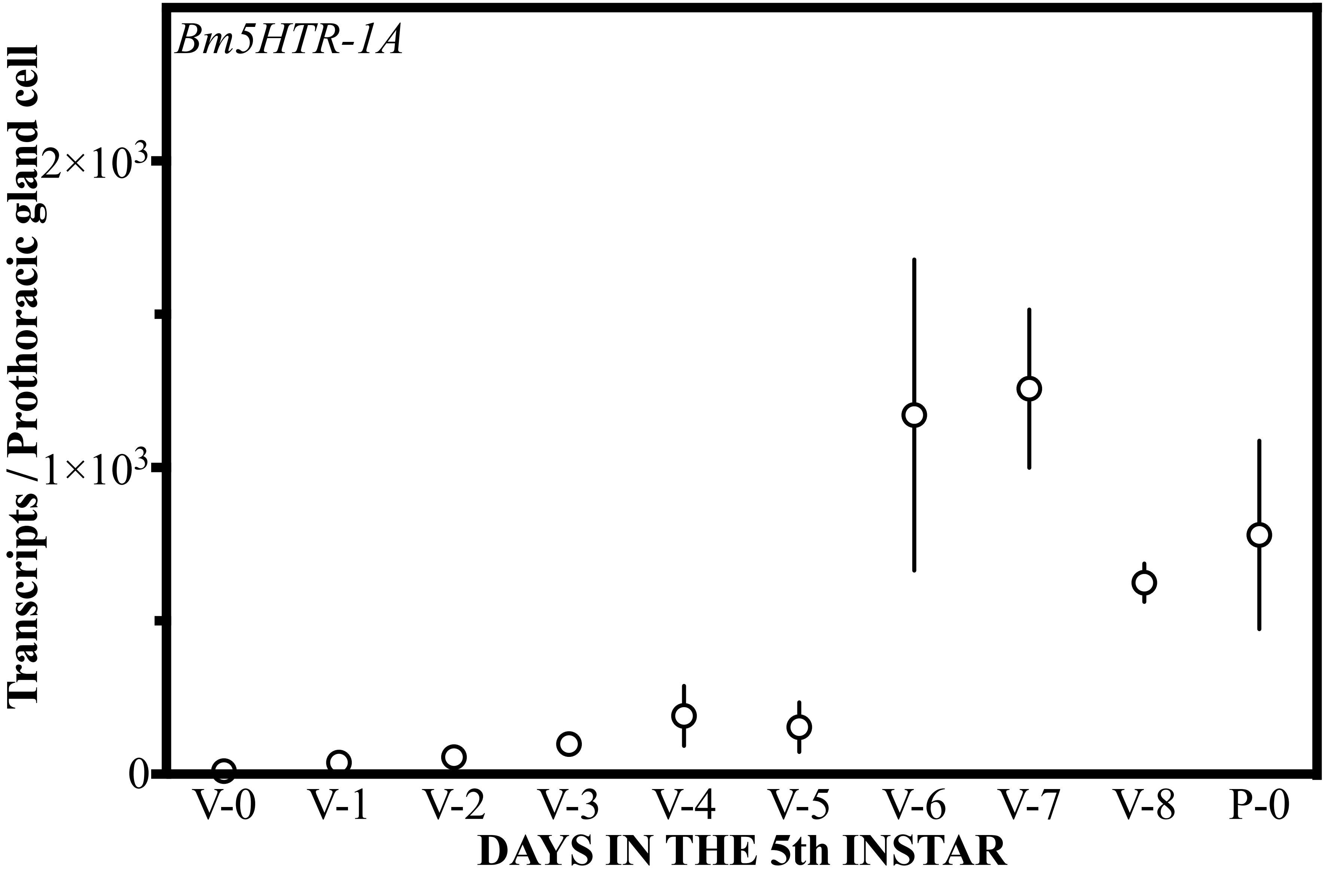

Bm5HTR-1A
Transcripts / Prothoracic gland cell
2×10³
1×10³
0
V-0
V-1
V-2
V-3
V-4
V-5
V-6
V-7
V-8
P-0
DAYS IN THE 5th INSTAR

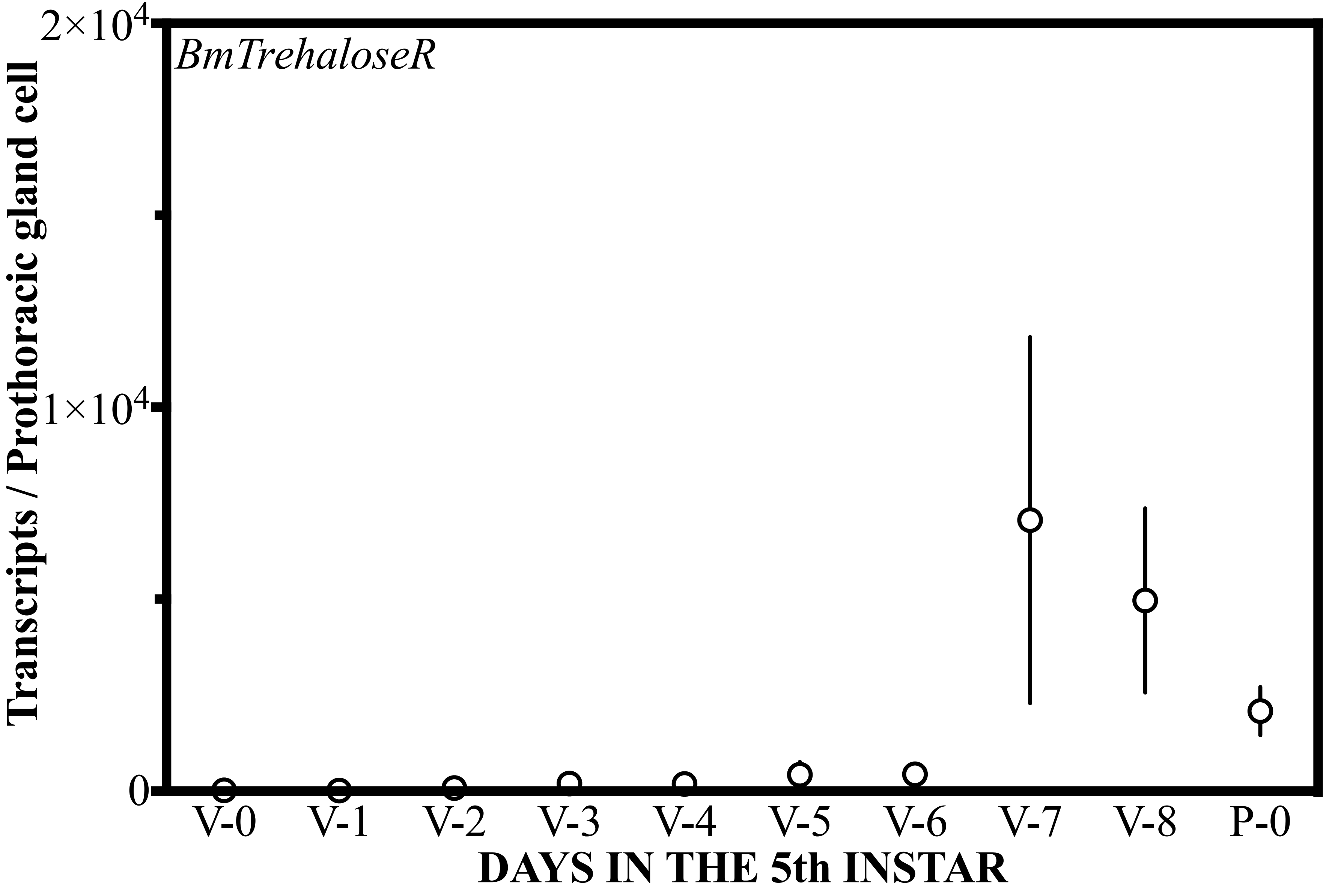
BmTrehaloseR
Transcripts / Prothoracic gland cell
2×10^4
1×10^4
0
V-0
V-1
V-2
V-3
V-4
V-5
V-6
V-7
V-8
P-0
DAYS IN THE 5th INSTAR

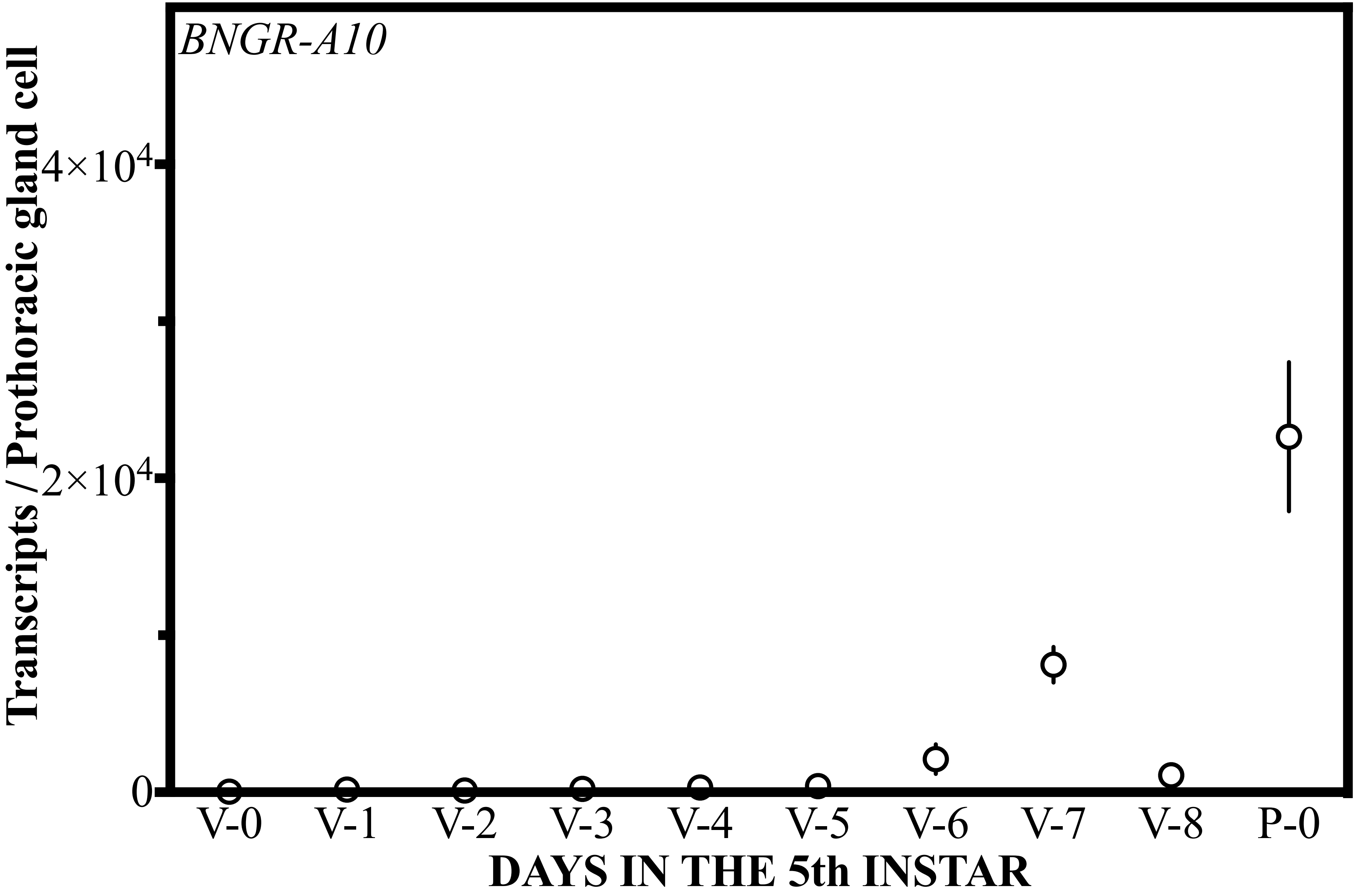
BNGR-A10
Transcripts / Prothoracic gland cell
4×10^4
2×10^4
0
V-0
V-1
V-2
V-3
V-4
V-5
V-6
V-7
V-8
P-0
DAYS IN THE 5th INSTAR

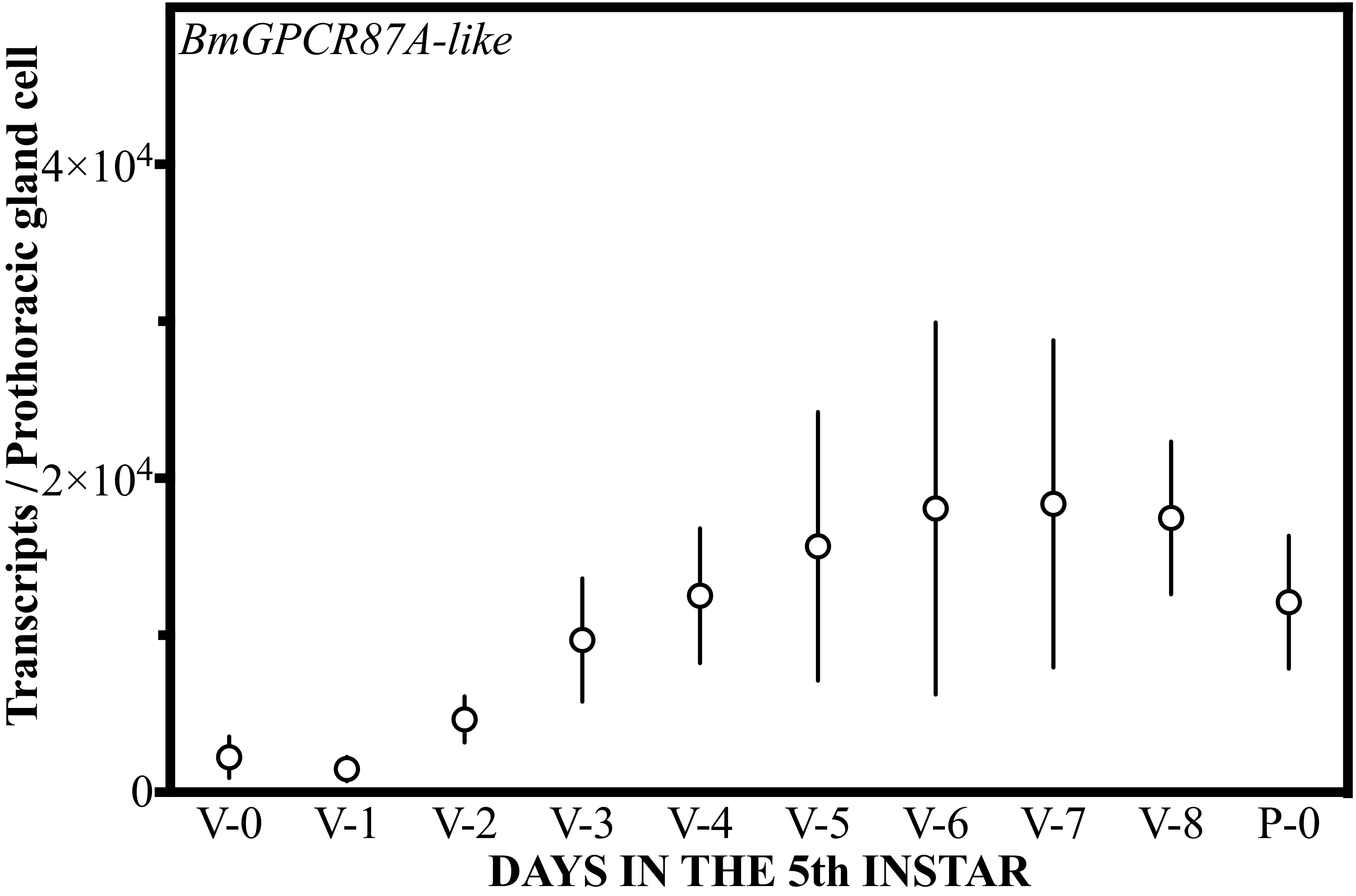

BmGPCR87A-like
Transcripts / Prothoracic gland cell
4×10^4
2×10^4
0
V-0
V-1
V-2
V-3
V-4
V-5
V-6
V-7
V-8
P-0
DAYS IN THE 5th INSTAR

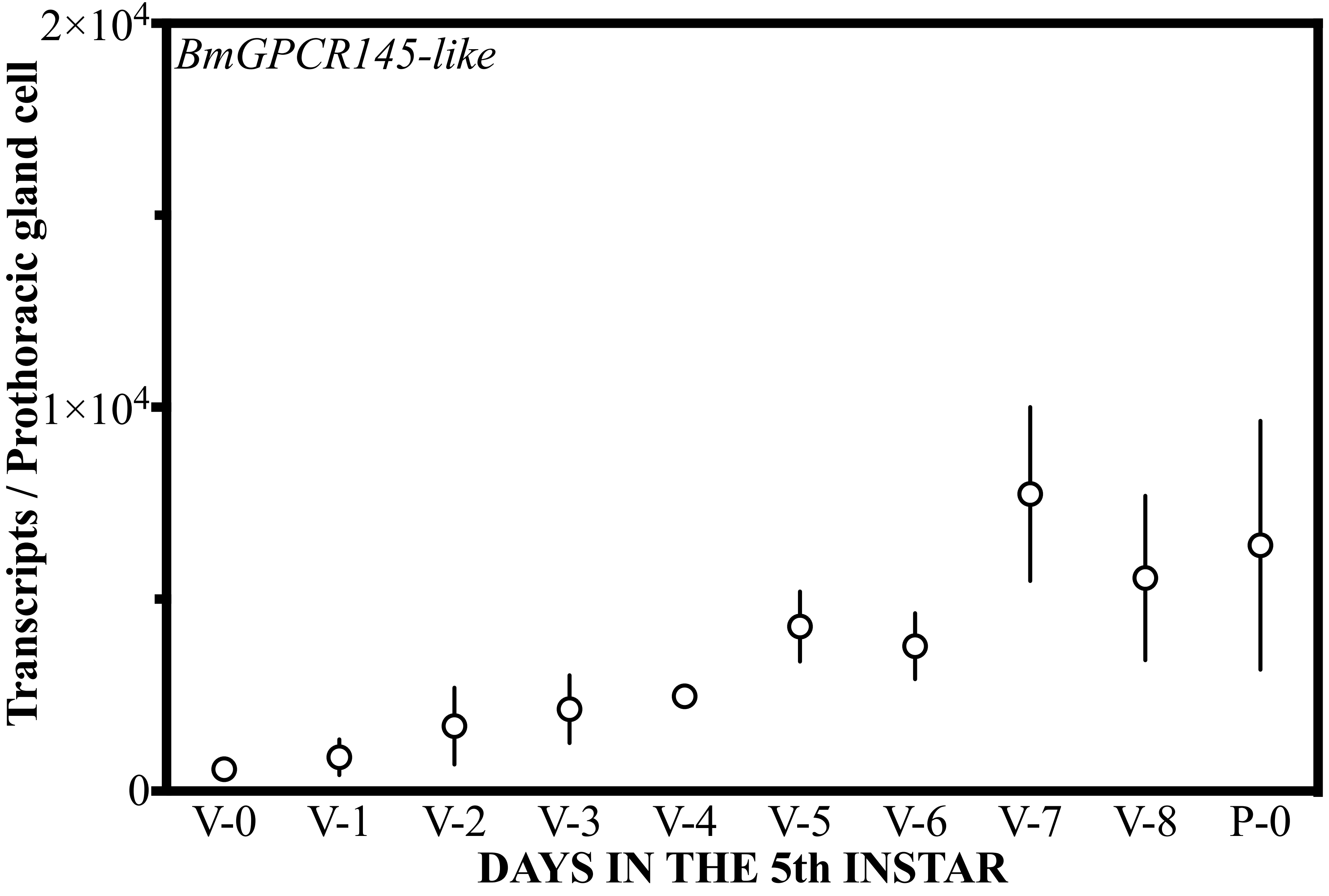
BmGPCR145-like
Transcripts / Prothoracic gland cell
2×10^4
1×10^4
0
V-0
V-1
V-2
V-3
V-4
V-5
V-6
V-7
V-8
P-0
DAYS IN THE 5th INSTAR

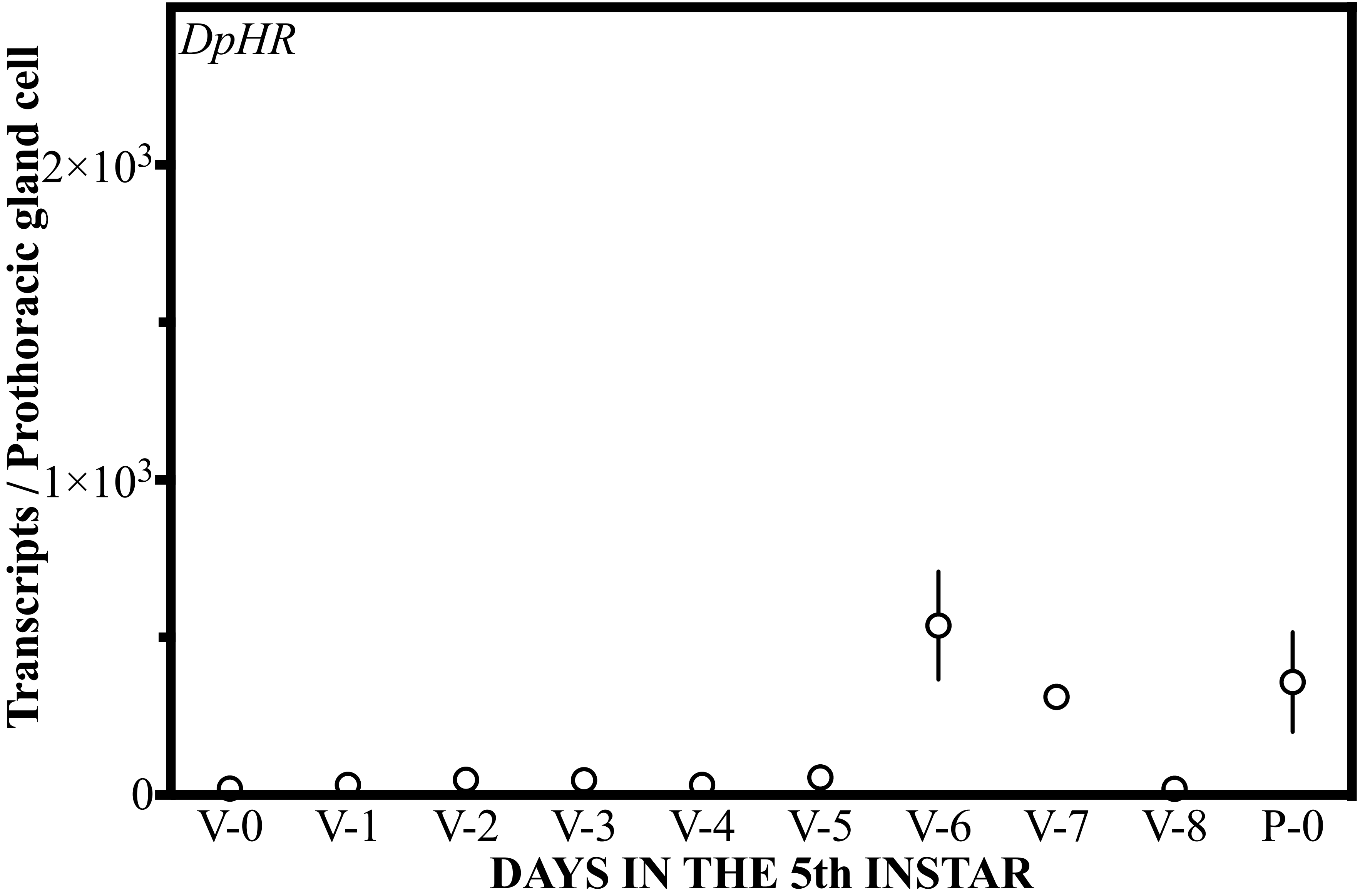

DpHR
Transcripts / Prothoracic gland cell
2×10³
1×10³
0
V-0
V-1
V-2
V-3
V-4
V-5
V-6
V-7
V-8
P-0
DAYS IN THE 5th INSTAR

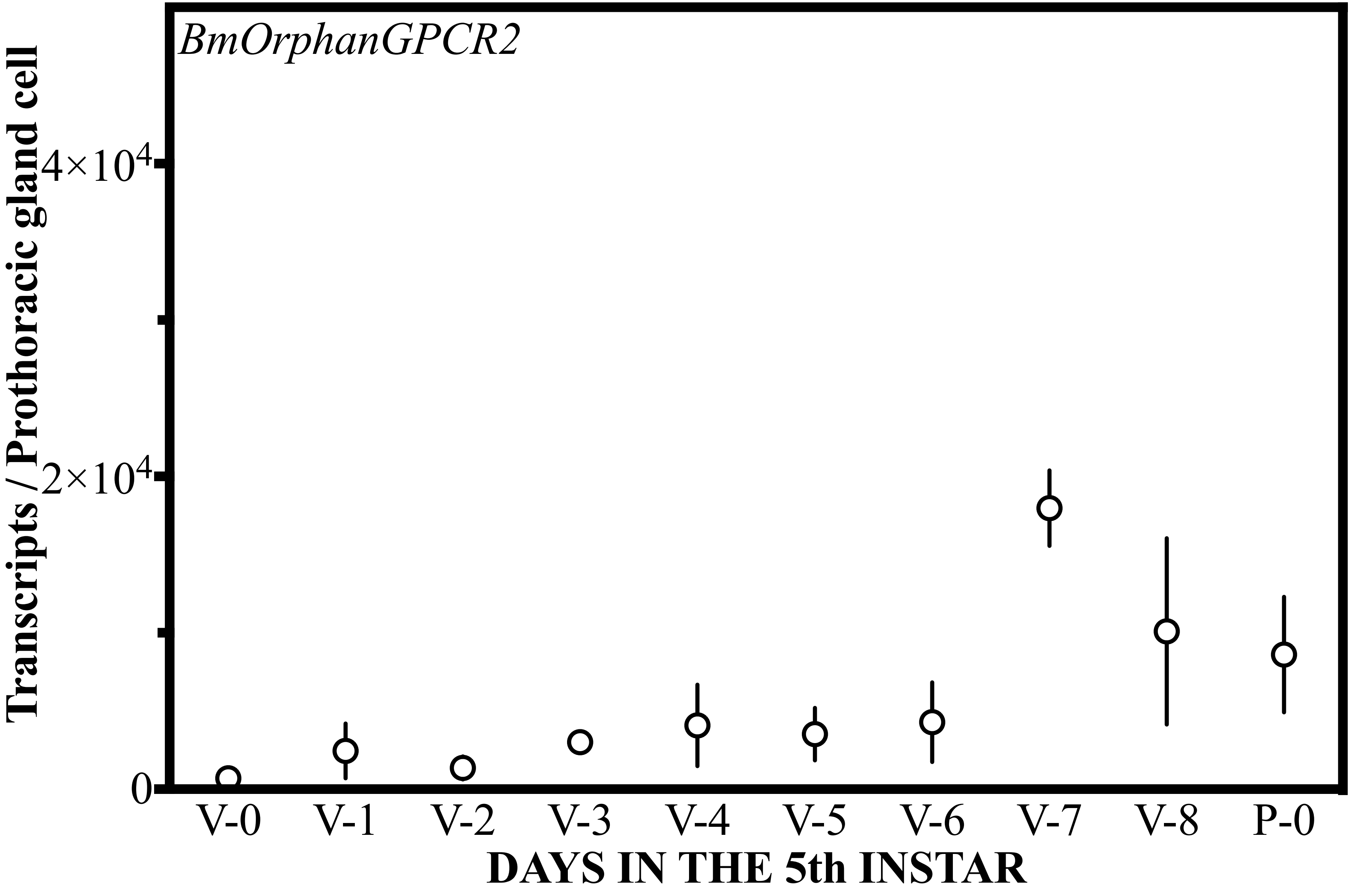

BmOrphanGPCR2
Transcripts / Prothoracic gland cell
4×10⁴
2×10⁴
0
V-0
V-1
V-2
V-3
V-4
V-5
V-6
V-7
V-8
P-0
DAYS IN THE 5th INSTAR

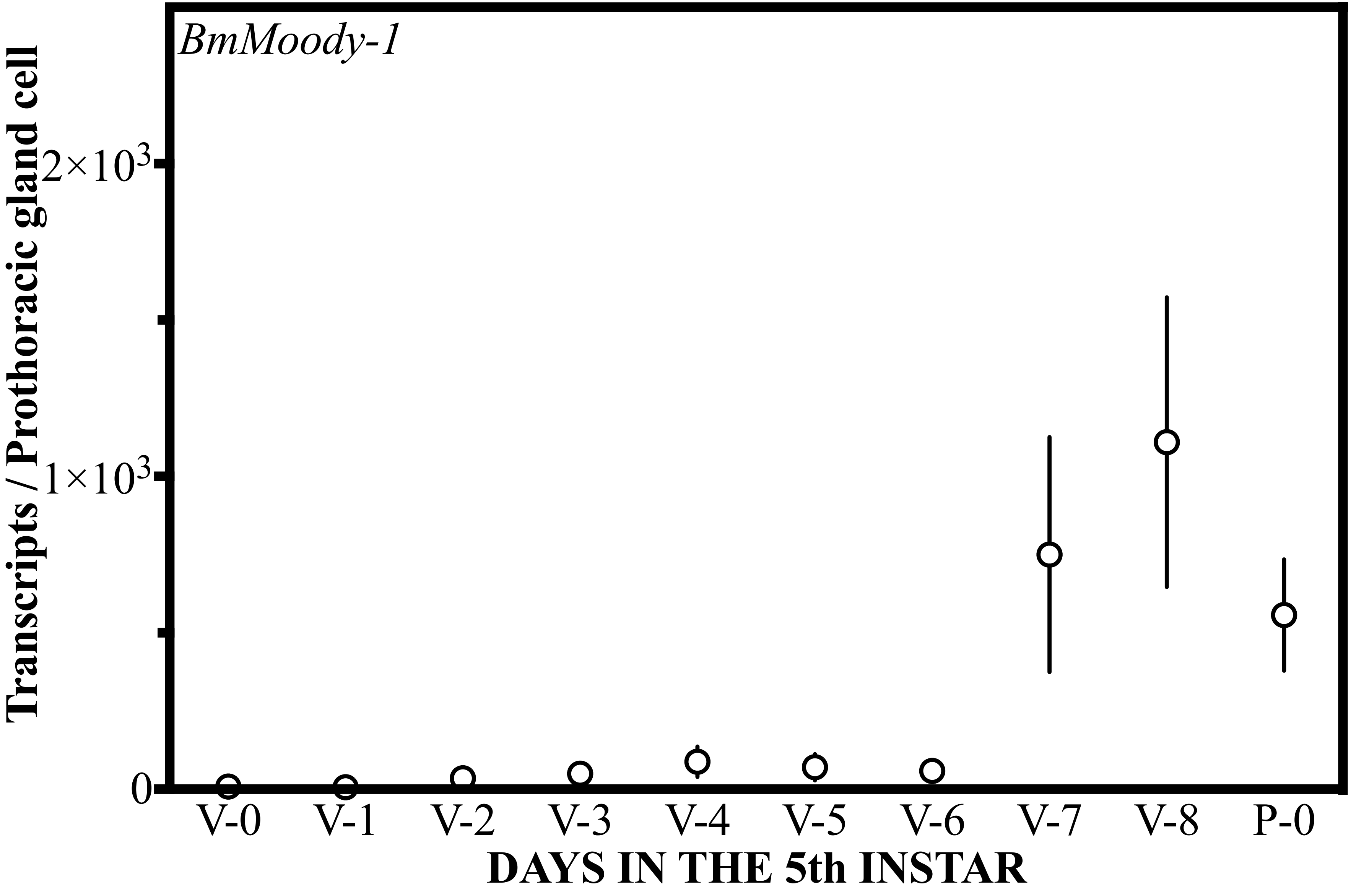

BmMoody-1
Transcripts / Prothoracic gland cell
2×10³
1×10³
0
V-0
V-1
V-2
V-3
V-4
V-5
V-6
V-7
V-8
P-0
DAYS IN THE 5th INSTAR

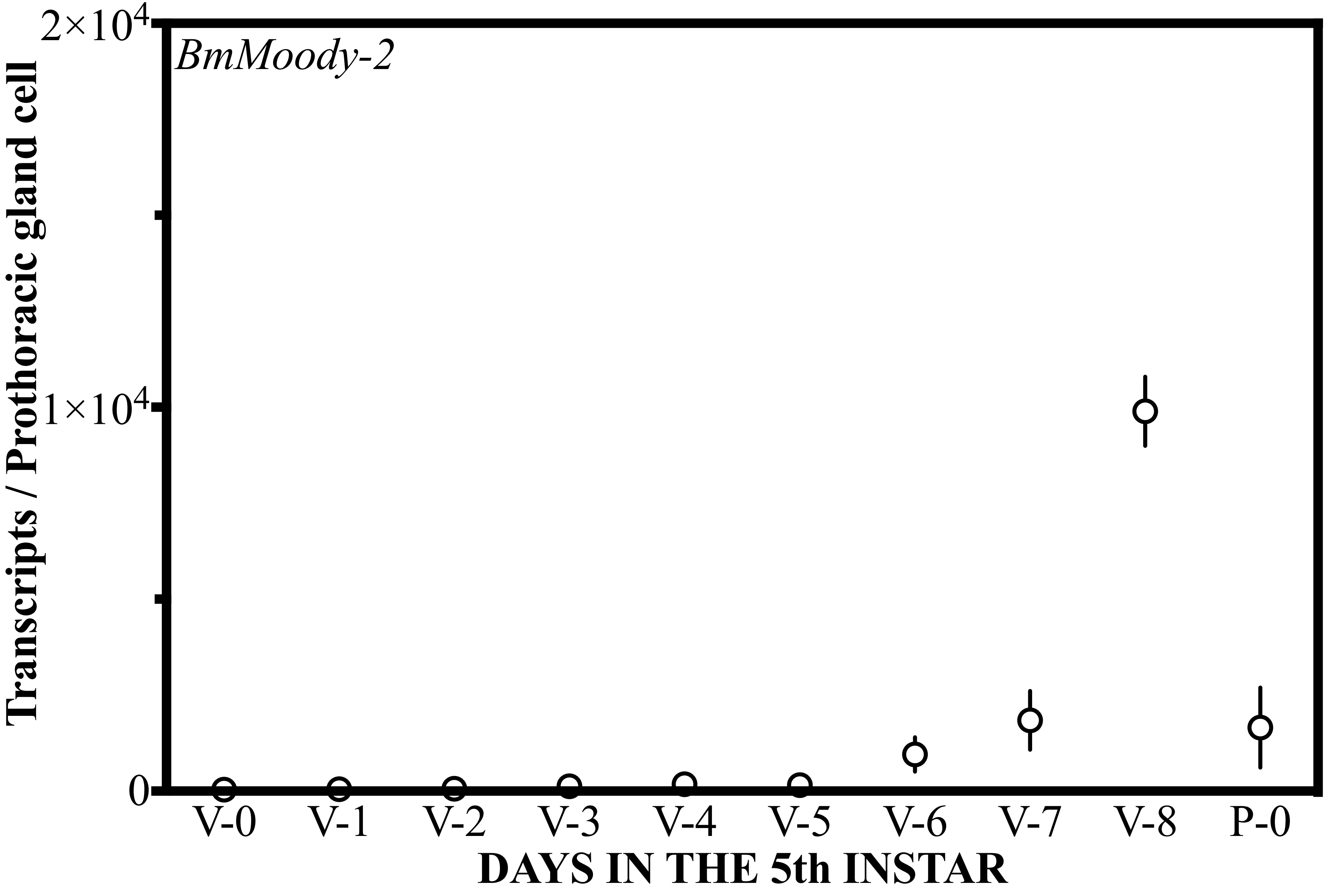
BmMoody-2
2×10^4
1×10^4
0
Transcripts / Prothoracic gland cell
V-0
V-1
V-2
V-3
V-4
V-5
V-6
V-7
V-8
P-0
DAYS IN THE 5th INSTAR

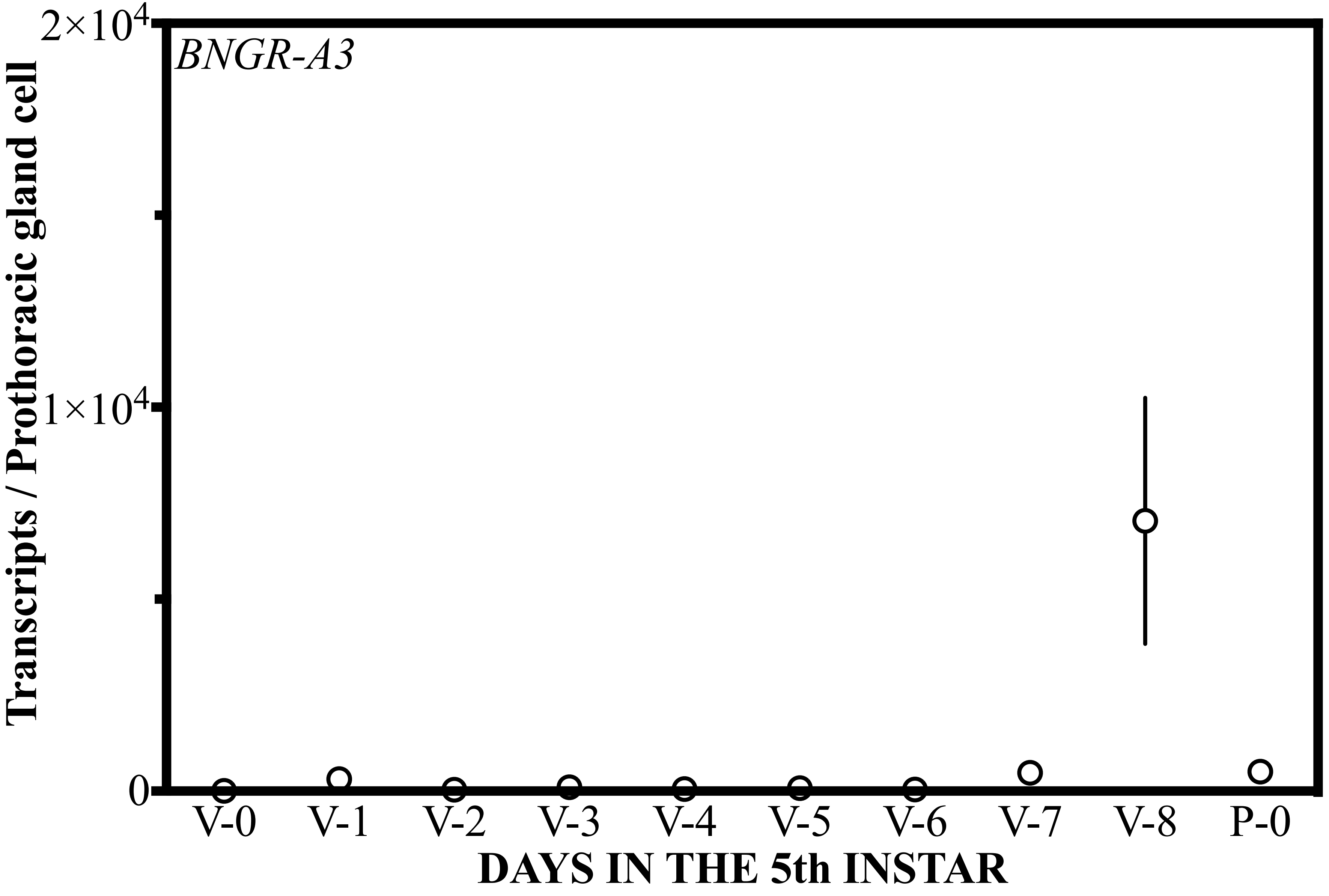
BNGR-A3
Transcripts / Prothoracic gland cell
2×10^4
1×10^4
0
V-0
V-1
V-2
V-3
V-4
V-5
V-6
V-7
V-8
P-0
DAYS IN THE 5th INSTAR

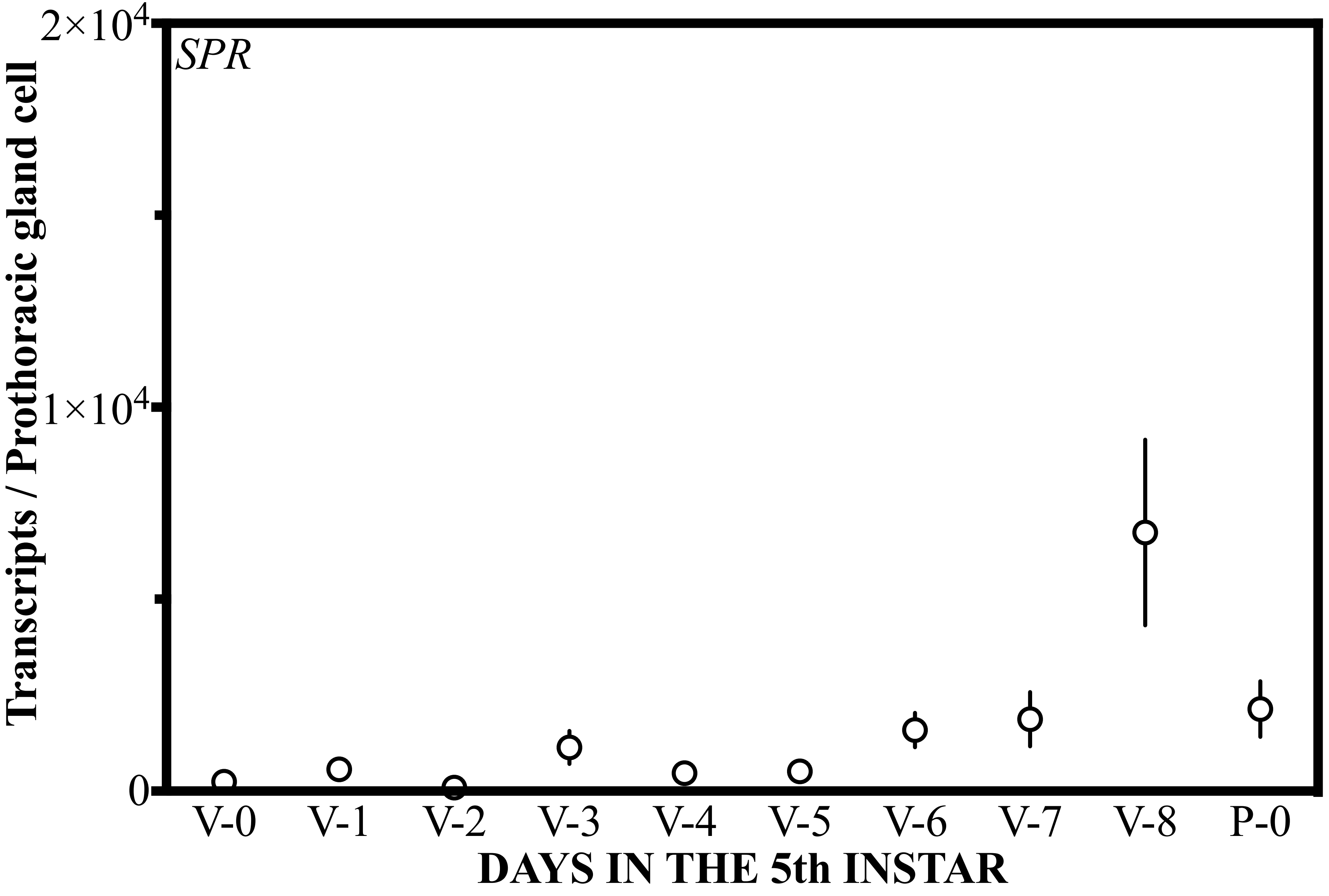
SPR
2×10^4
1×10^4
0
Transcripts / Prothoracic gland cell
V-0
V-1
V-2
V-3
V-4
V-5
V-6
V-7
V-8
P-0
DAYS IN THE 5th INSTAR

Class B

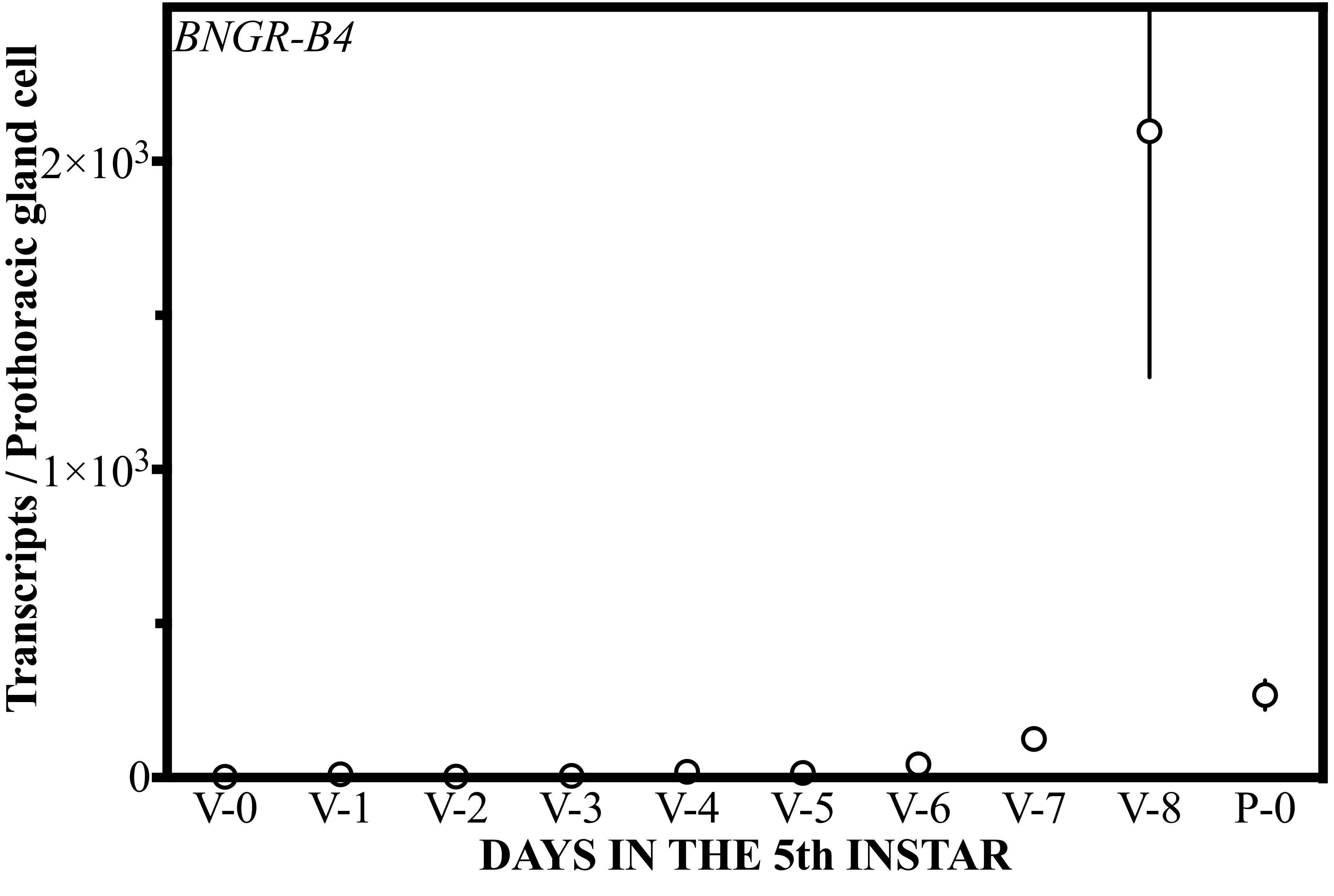

BNGR-B4
Transcripts / Prothoracic gland cell
2×10³
1×10³
0
V-0
V-1
V-2
V-3
V-4
V-5
V-6
V-7
V-8
P-0
DAYS IN THE 5th INSTAR

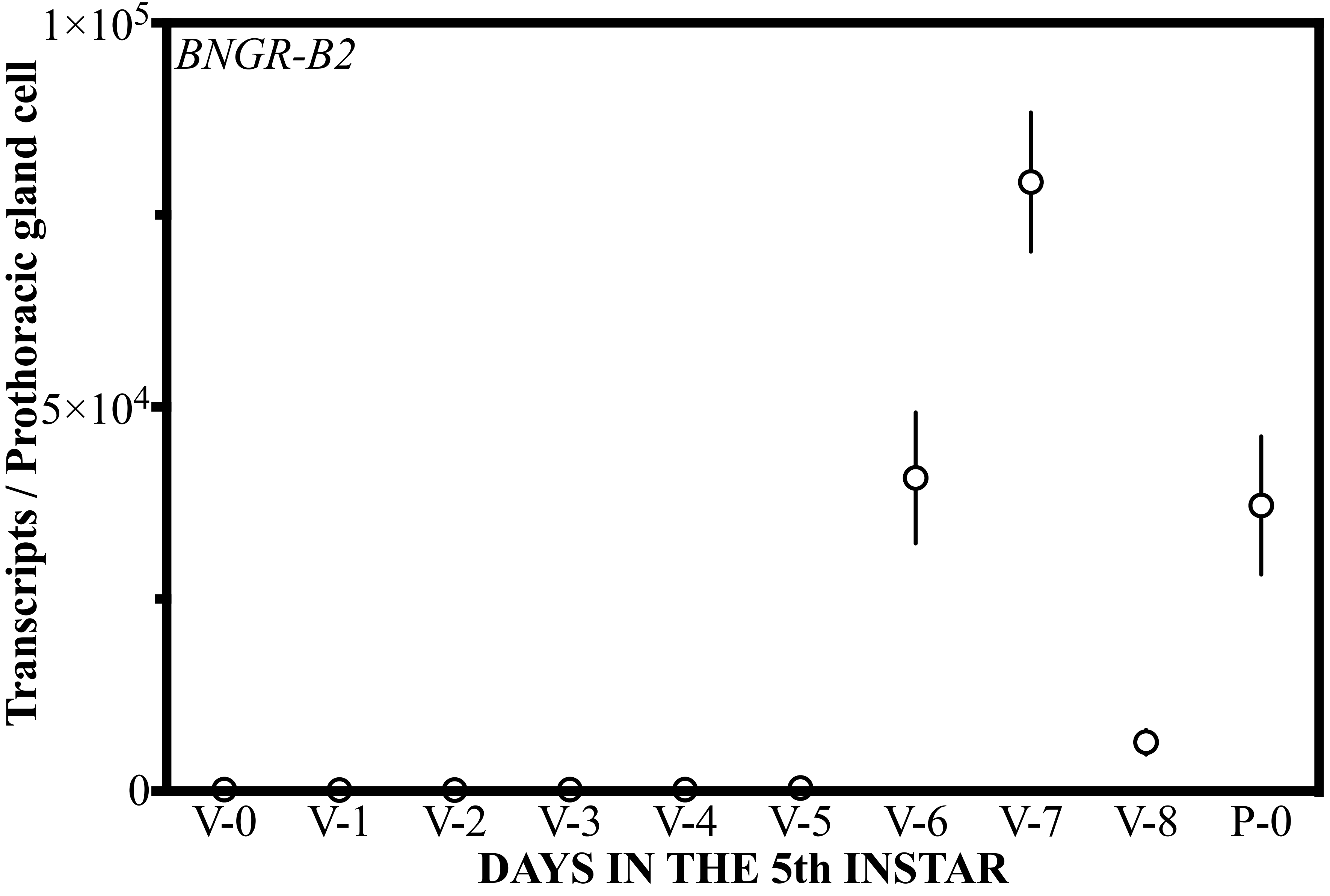

BNGR-B2
1×10^5
5×10^4
0
Transcripts / Prothoracic gland cell
V-0
V-1
V-2
V-3
V-4
V-5
V-6
V-7
V-8
P-0
DAYS IN THE 5th INSTAR

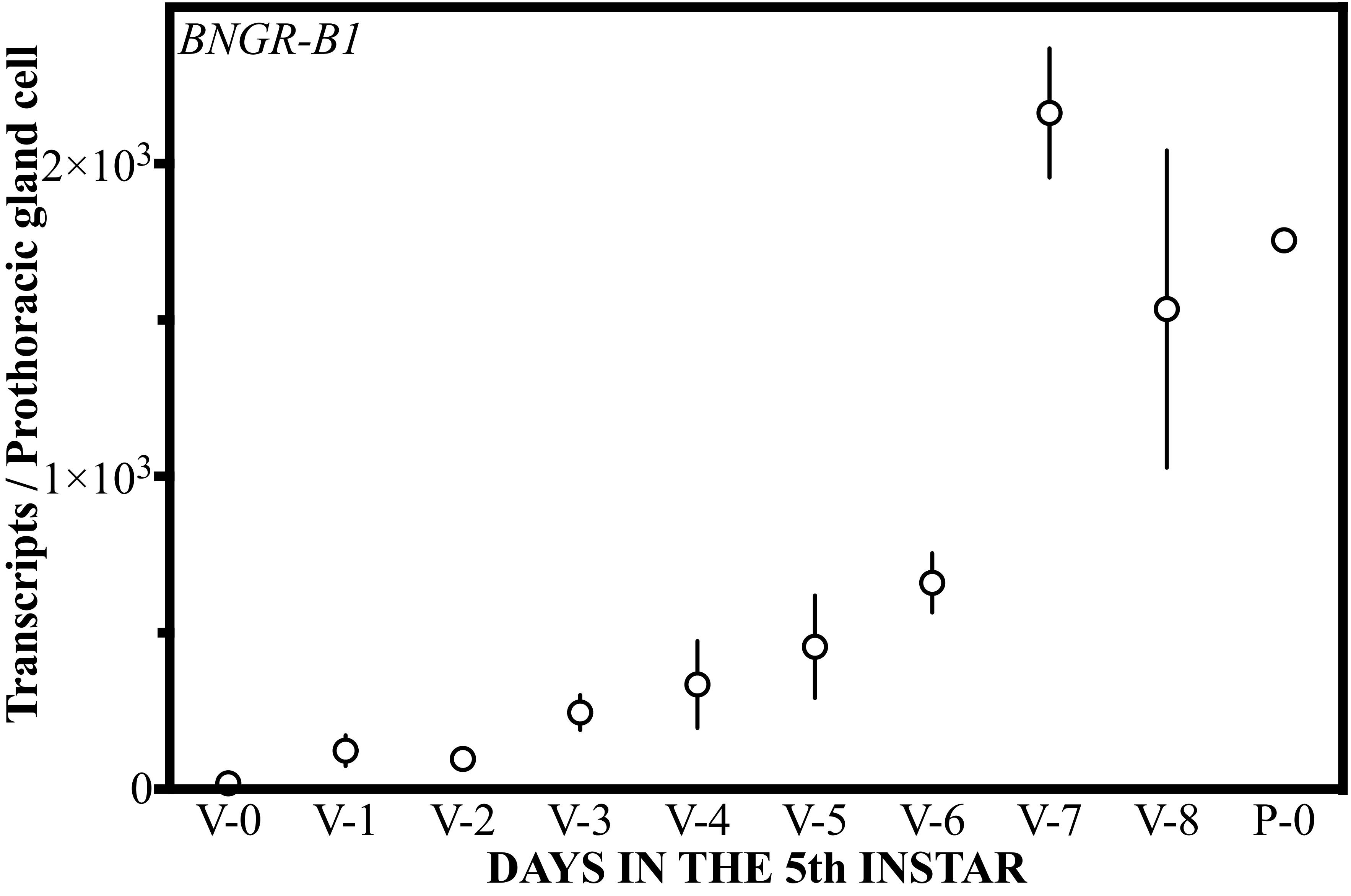

BNGR-B1
Transcripts / Prothoracic gland cell
2×10³
1×10³
0
V-0
V-1
V-2
V-3
V-4
V-5
V-6
V-7
V-8
P-0
DAYS IN THE 5th INSTAR

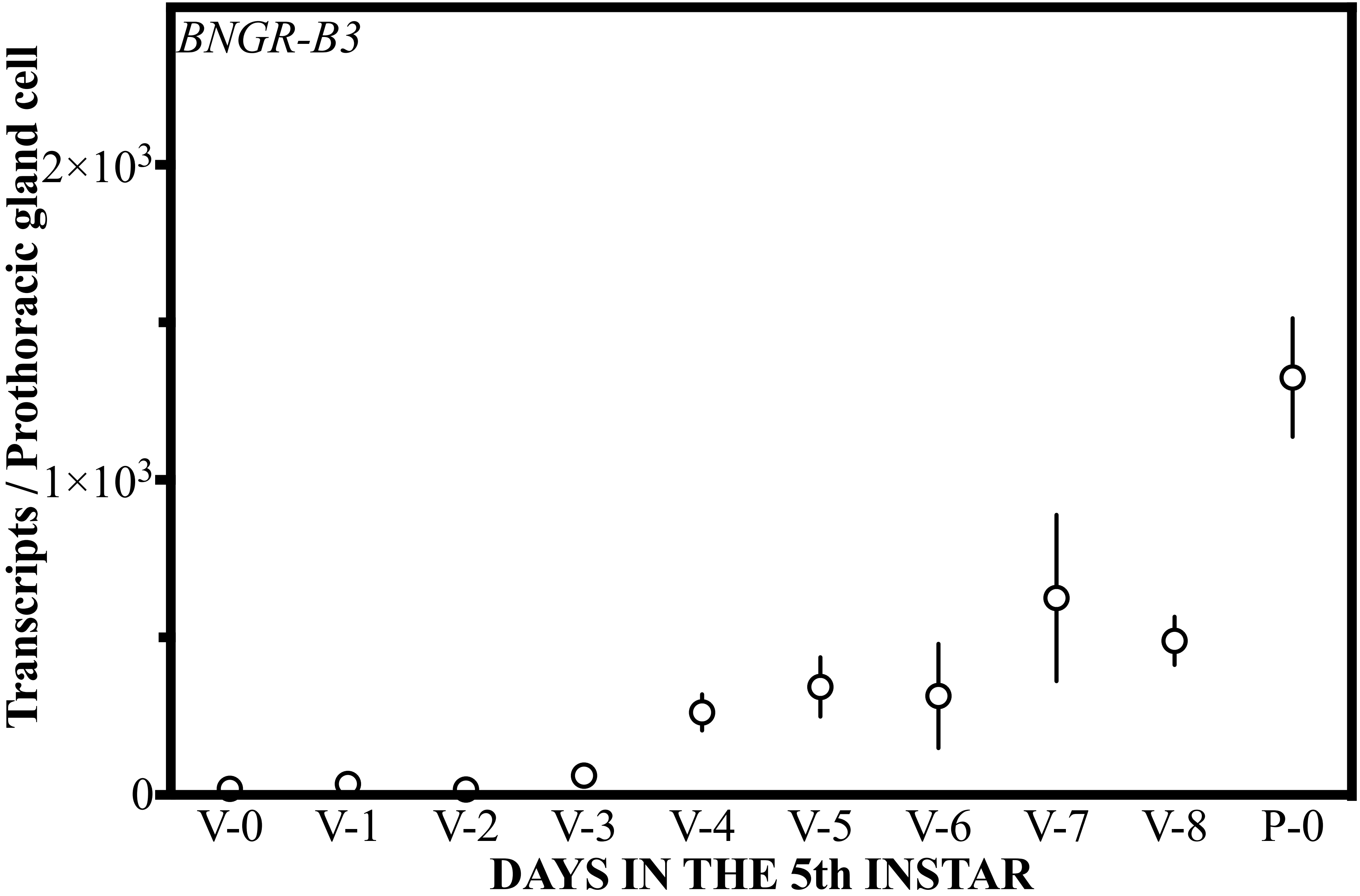
BNGR-B3
Transcripts / Prothoracic gland cell
2×10³
1×10³
0
V-0
V-1
V-2
V-3
V-4
V-5
V-6
V-7
V-8
P-0
DAYS IN THE 5th INSTAR

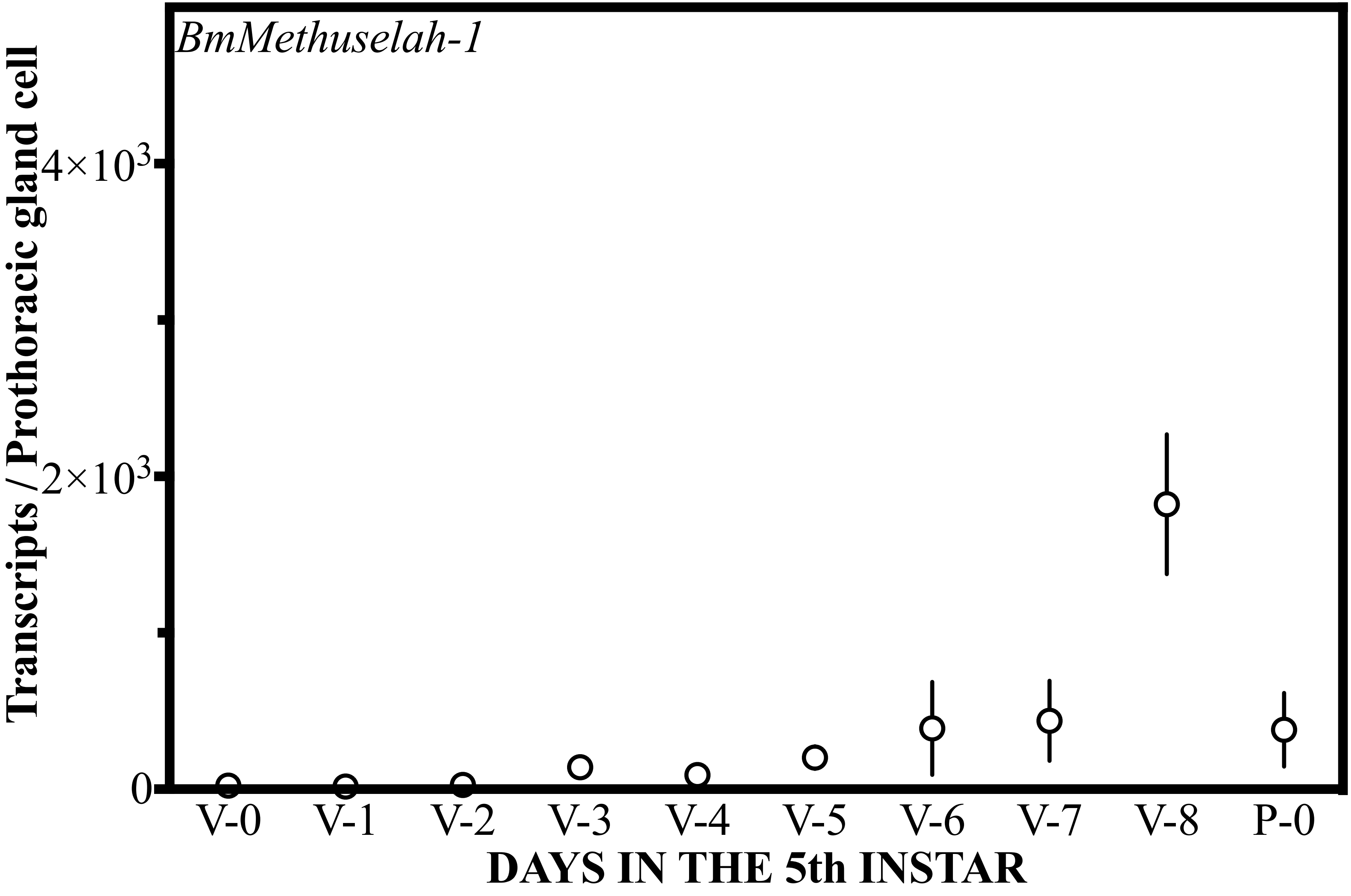
BmMethuselah-1
Transcripts / Prothoracic gland cell
4×10³
2×10³
0
V-0
V-1
V-2
V-3
V-4
V-5
V-6
V-7
V-8
P-0
DAYS IN THE 5th INSTAR

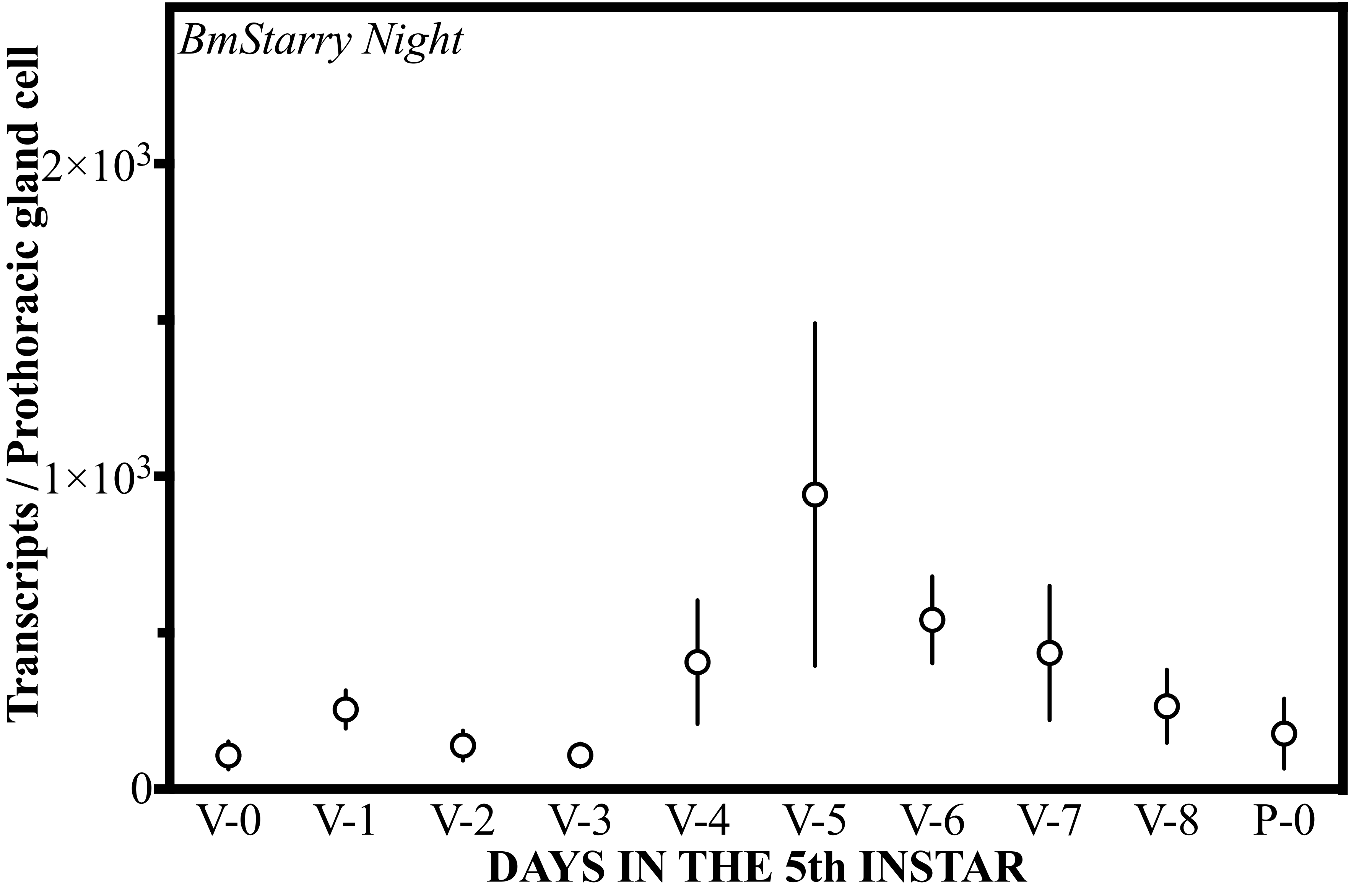
BmStarry Night
Transcripts / Prothoracic gland cell
2×10^3
1×10^3
0
V-0
V-1
V-2
V-3
V-4
V-5
V-6
V-7
V-8
P-0
DAYS IN THE 5th INSTAR

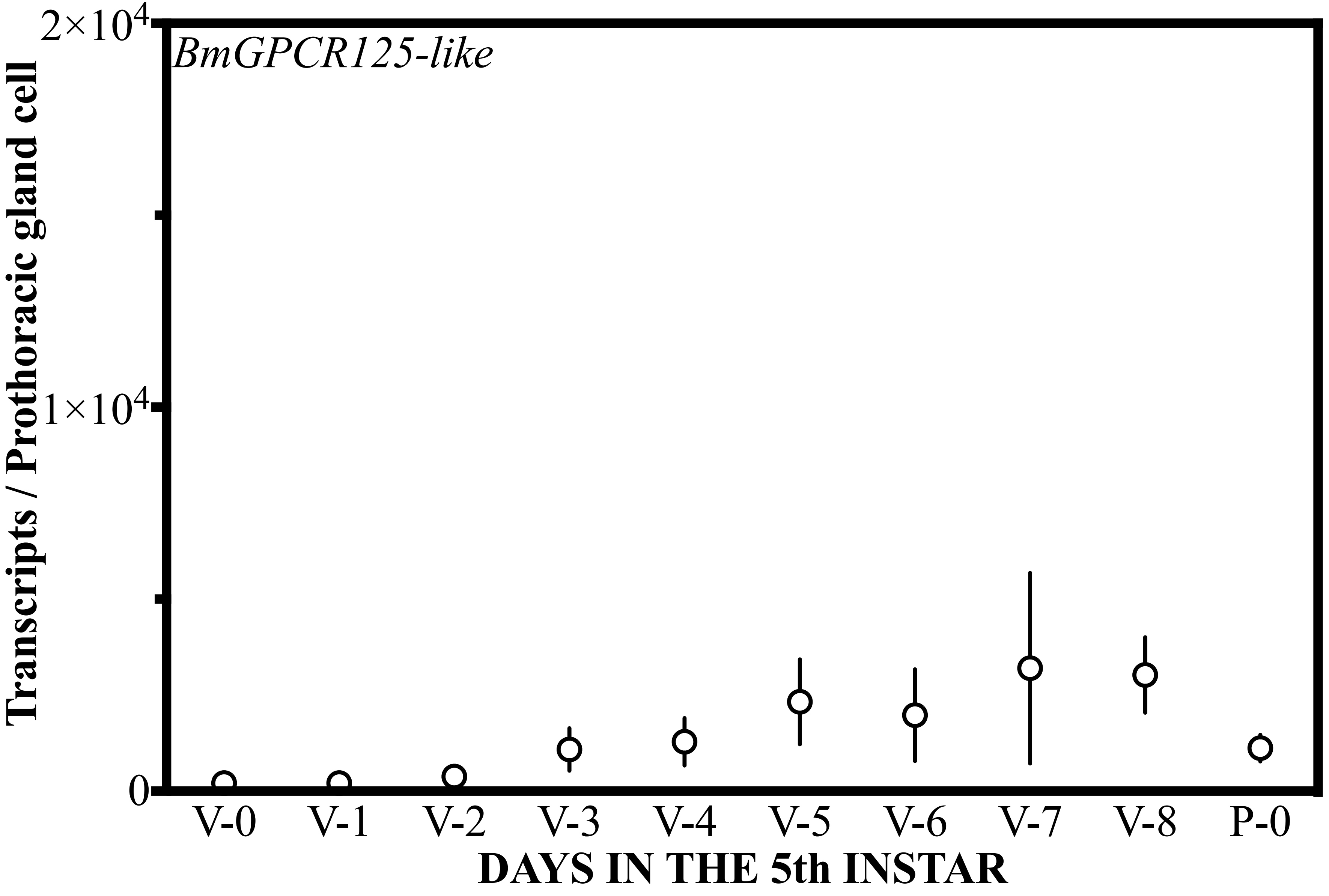
BmGPCR125-like
Transcripts / Prothoracic gland cell
2×10^4
1×10^4
0
V-0
V-1
V-2
V-3
V-4
V-5
V-6
V-7
V-8
P-0
DAYS IN THE 5th INSTAR

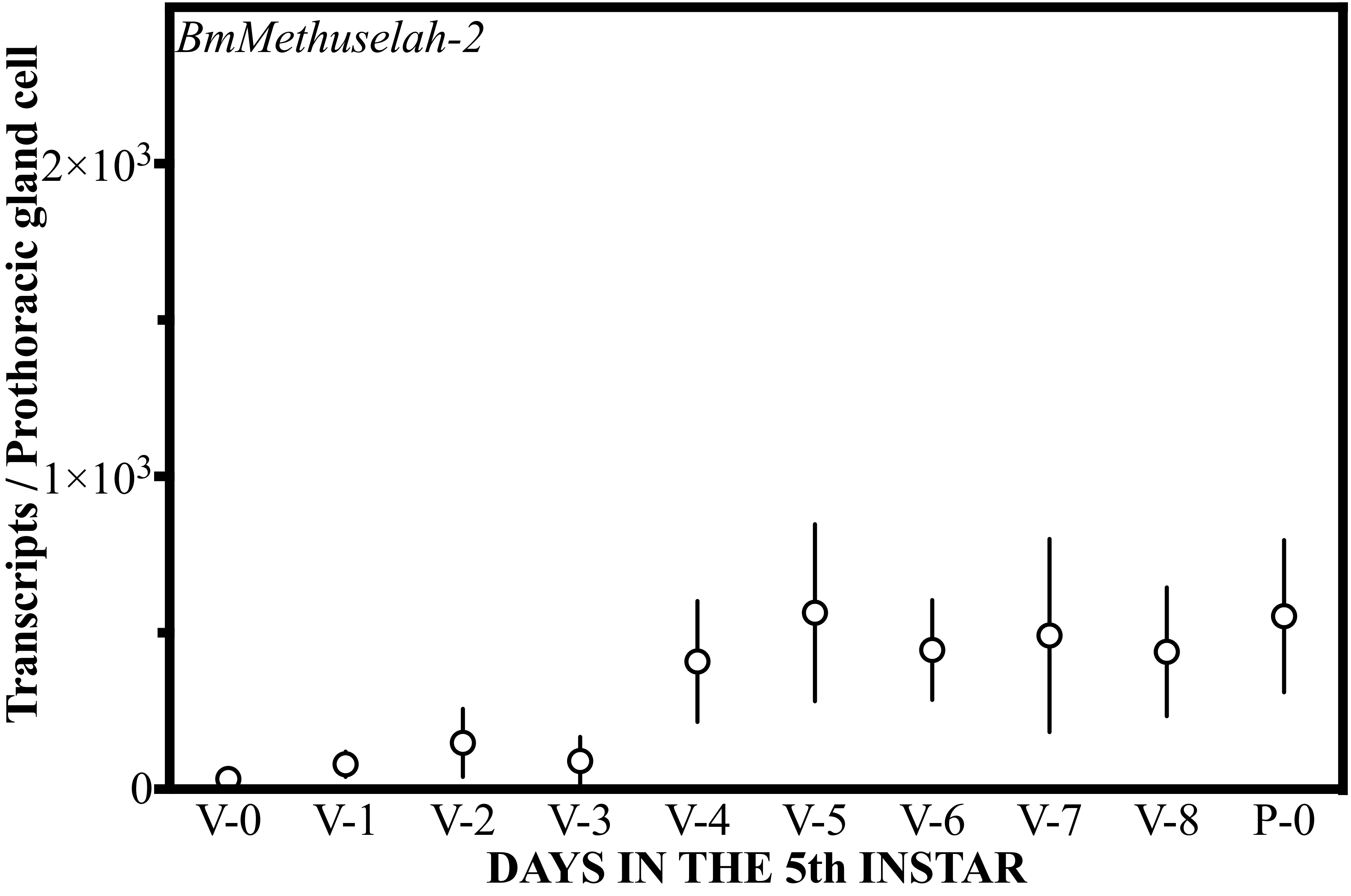

BmMethuselah-2
Transcripts / Prothoracic gland cell
2×10³
1×10³
0
V-0
V-1
V-2
V-3
V-4
V-5
V-6
V-7
V-8
P-0
DAYS IN THE 5th INSTAR

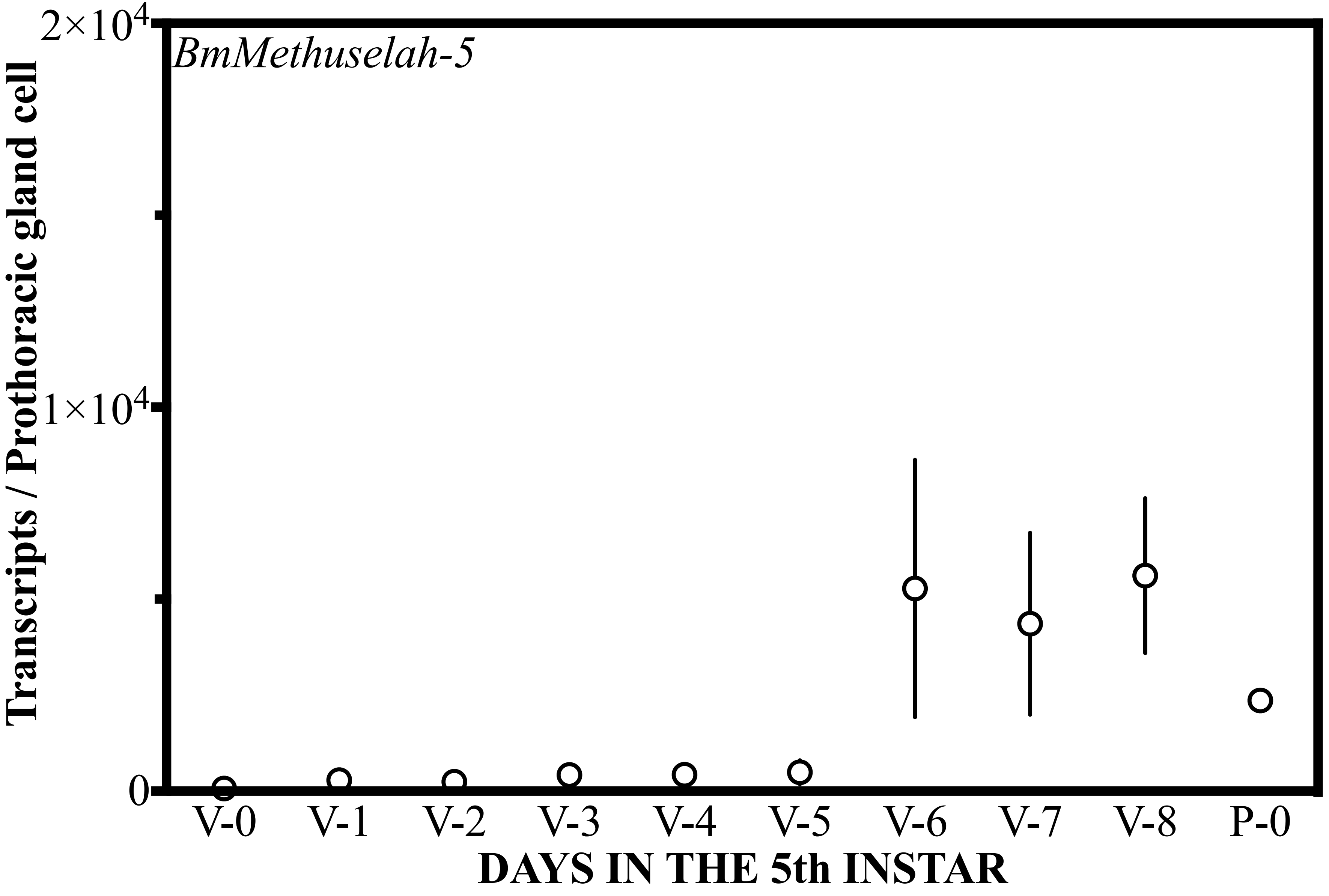

BmMethuselah-5
2×10^4
1×10^4
0
Transcripts / Prothoracic gland cell
V-0
V-1
V-2
V-3
V-4
V-5
V-6
V-7
V-8
P-0
DAYS IN THE 5th INSTAR

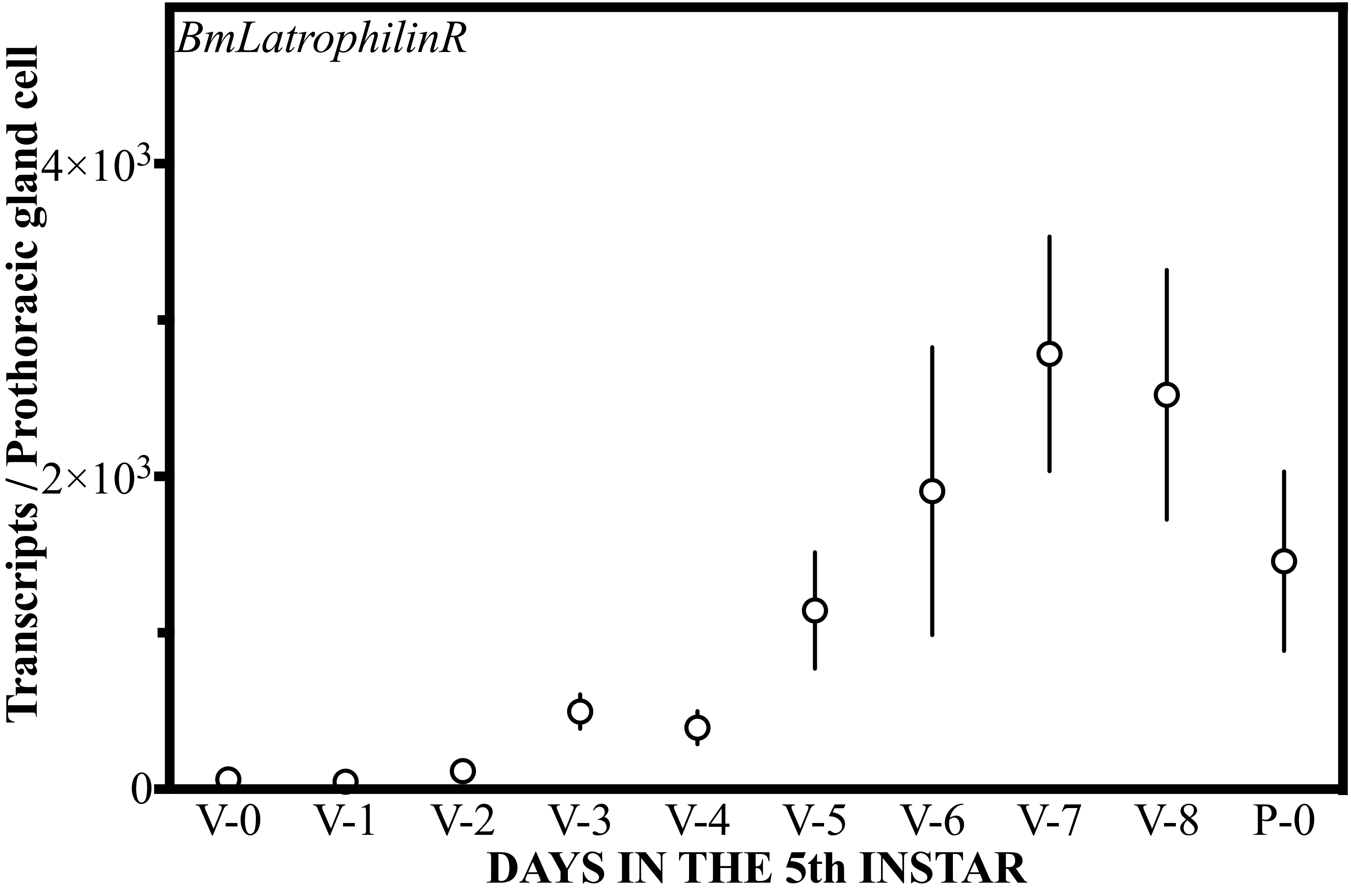

BmLatrophilinR
Transcripts / Prothoracic gland cell
4×10³
2×10³
0
V-0
V-1
V-2
V-3
V-4
V-5
V-6
V-7
V-8
P-0
DAYS IN THE 5th INSTAR

Class C

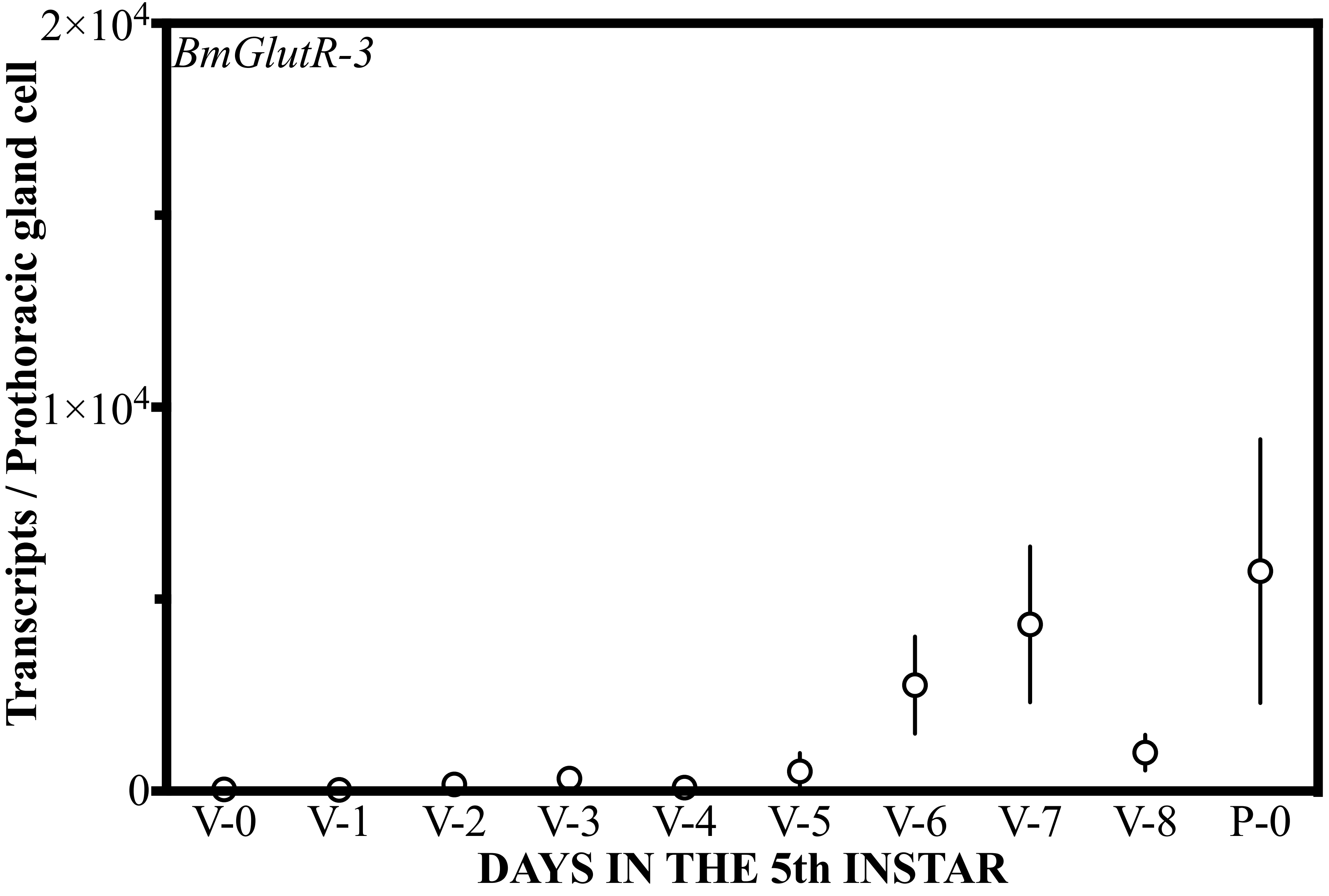

BmGlutR-3
2×10^4
1×10^4
0
Transcripts / Prothoracic gland cell
V-0
V-1
V-2
V-3
V-4
V-5
V-6
V-7
V-8
P-0
DAYS IN THE 5th INSTAR

Frizzled/Smoothened

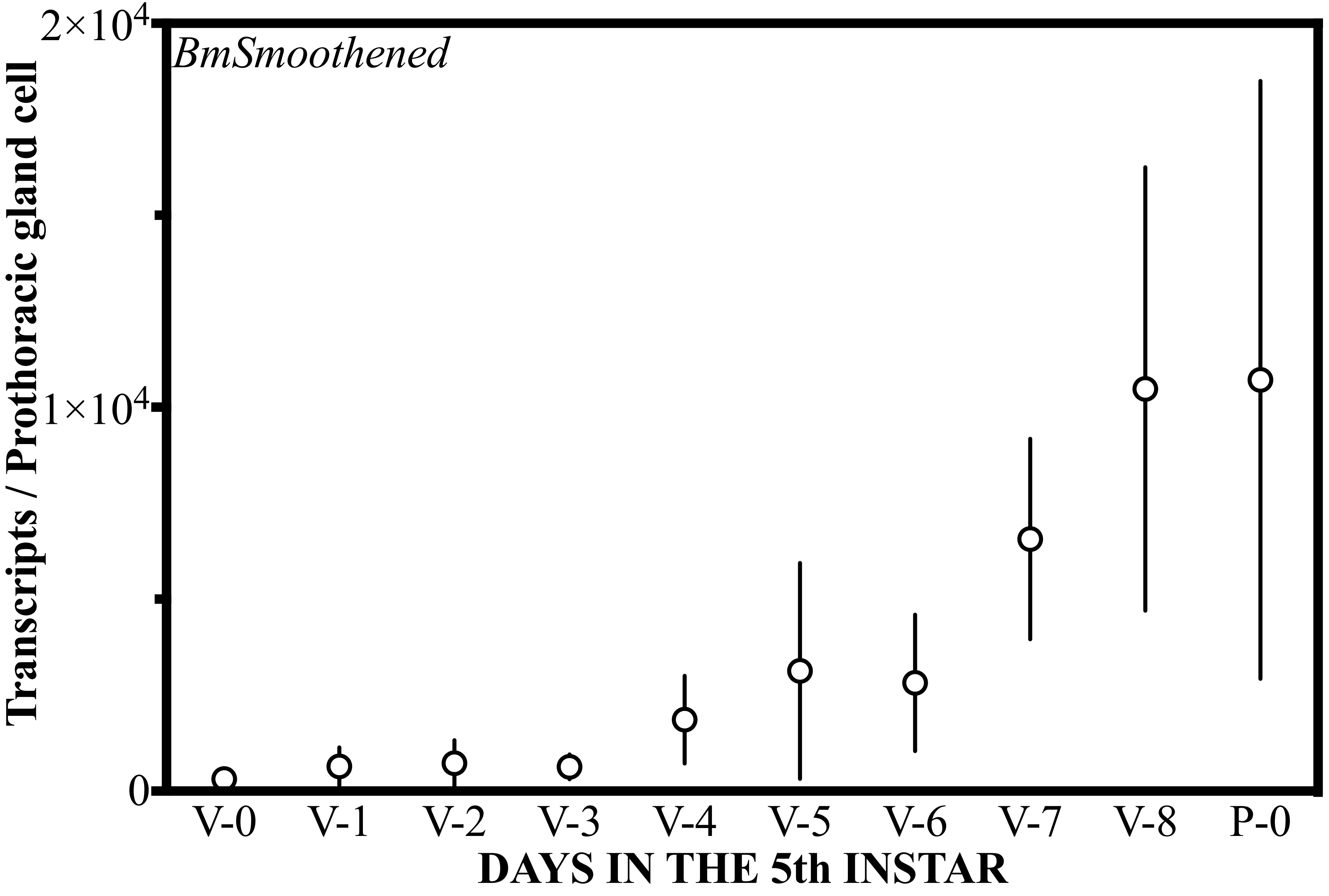

BmSmoothened
2×10^4
1×10^4
0
Transcripts / Prothoracic gland cell
V-0
V-1
V-2
V-3
V-4
V-5
V-6
V-7
V-8
P-0
DAYS IN THE 5th INSTAR

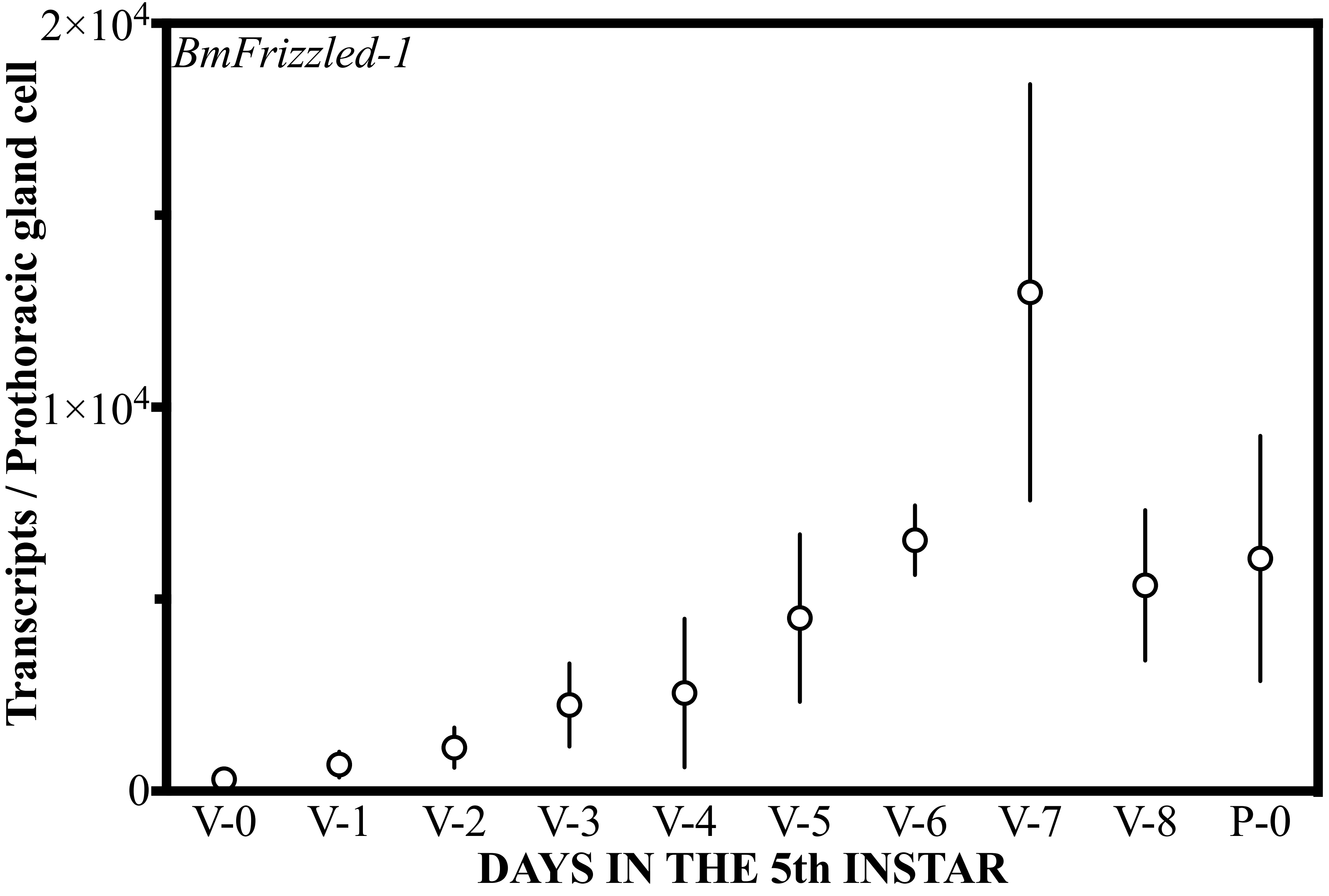

BmFrizzled-1
2×10^4
1×10^4
0
Transcripts / Prothoracic gland cell
V-0
V-1
V-2
V-3
V-4
V-5
V-6
V-7
V-8
P-0
DAYS IN THE 5th INSTAR

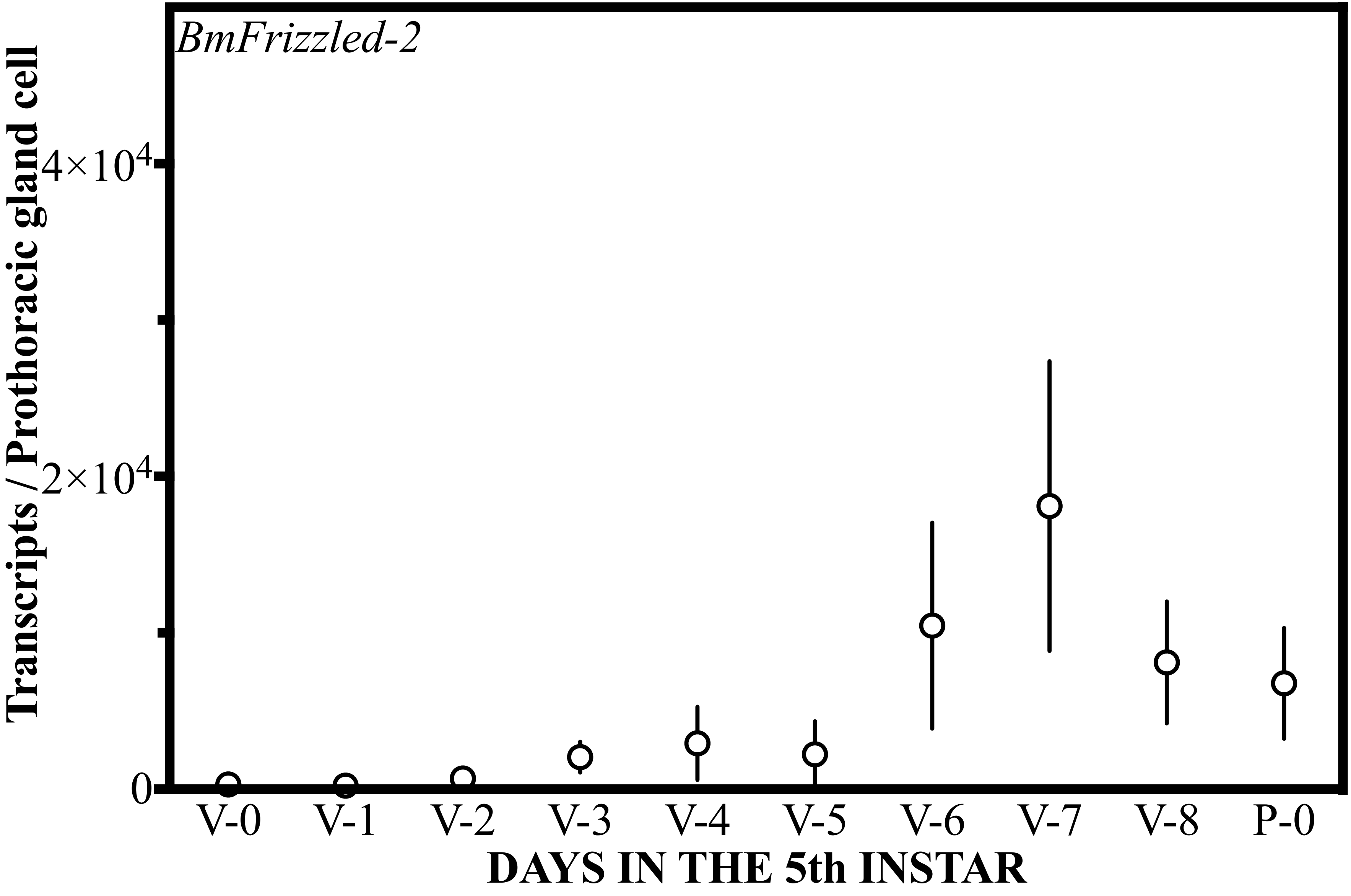

BmFrizzled-2
Transcripts / Prothoracic gland cell
4×10^4
2×10^4
0
V-0
V-1
V-2
V-3
V-4
V-5
V-6
V-7
V-8
P-0
DAYS IN THE 5th INSTAR

Unclassified 7 TM Receptors

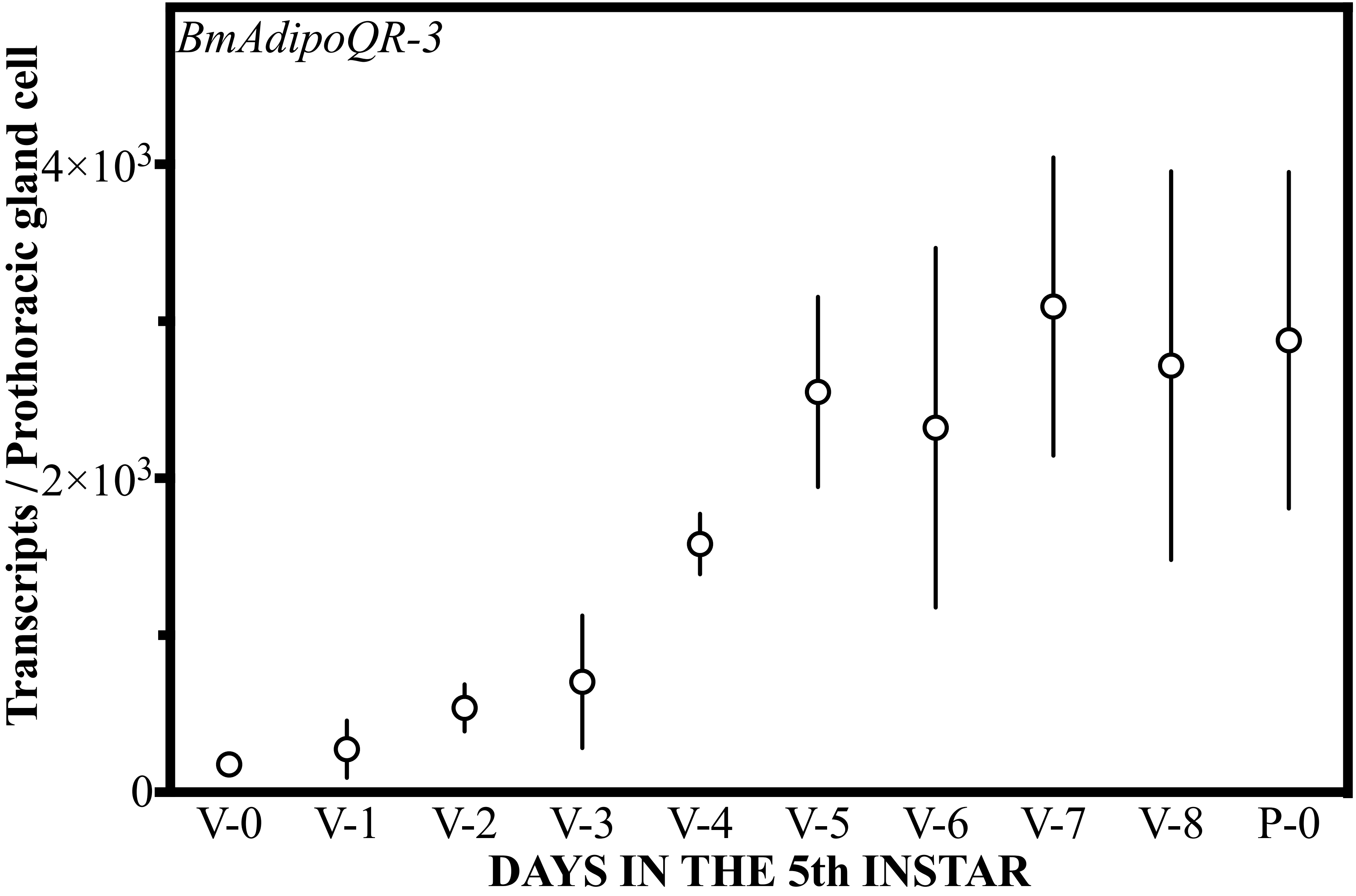

BmAdipoQR-3
Transcripts / Prothoracic gland cell
4×10³
2×10³
0
V-0
V-1
V-2
V-3
V-4
V-5
V-6
V-7
V-8
P-0
DAYS IN THE 5th INSTAR

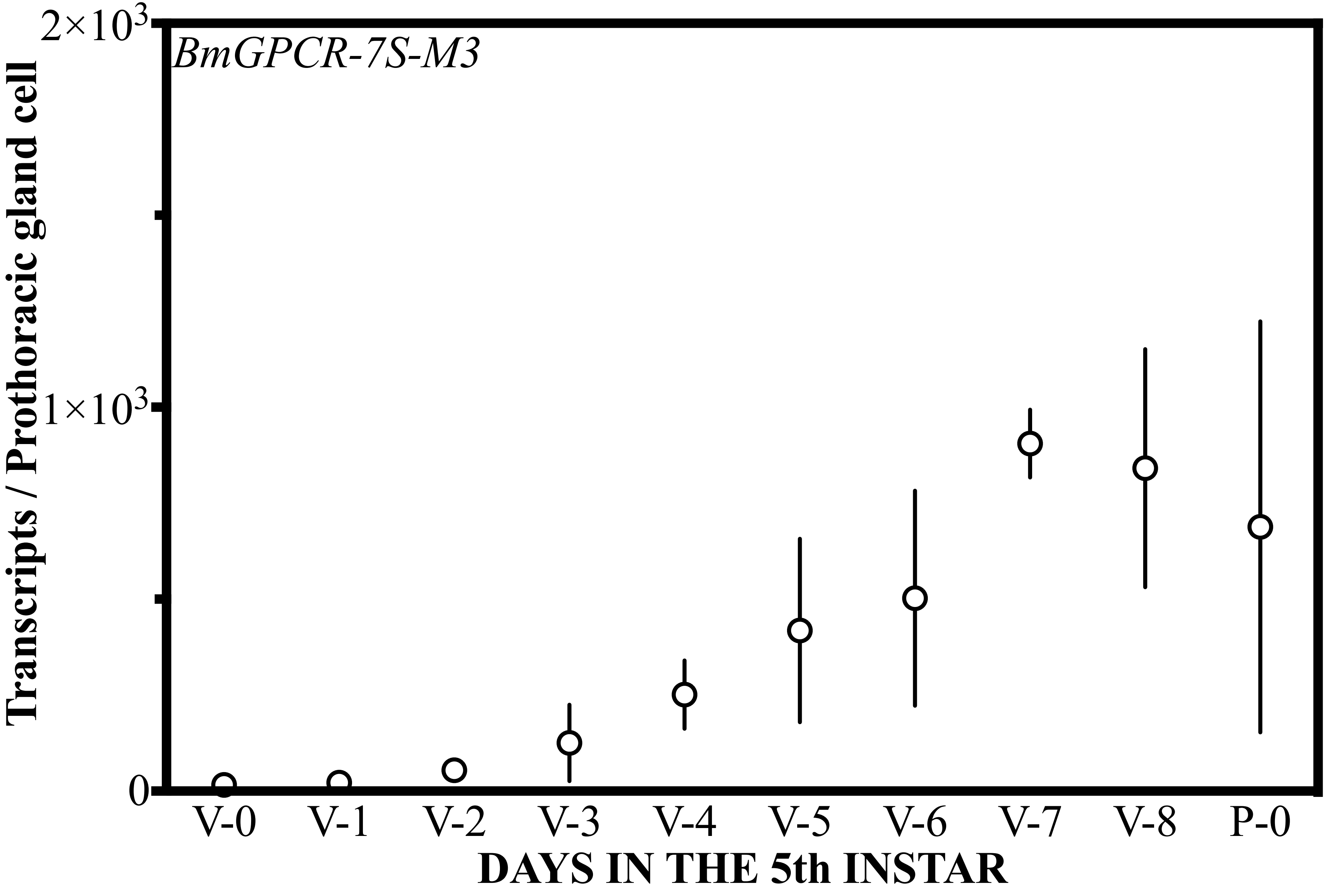
BmGPCR-7S-M3
2×10^3
1×10^3
0
Transcripts / Prothoracic gland cell
V-0
V-1
V-2
V-3
V-4
V-5
V-6
V-7
V-8
P-0
DAYS IN THE 5th INSTAR

RTKs

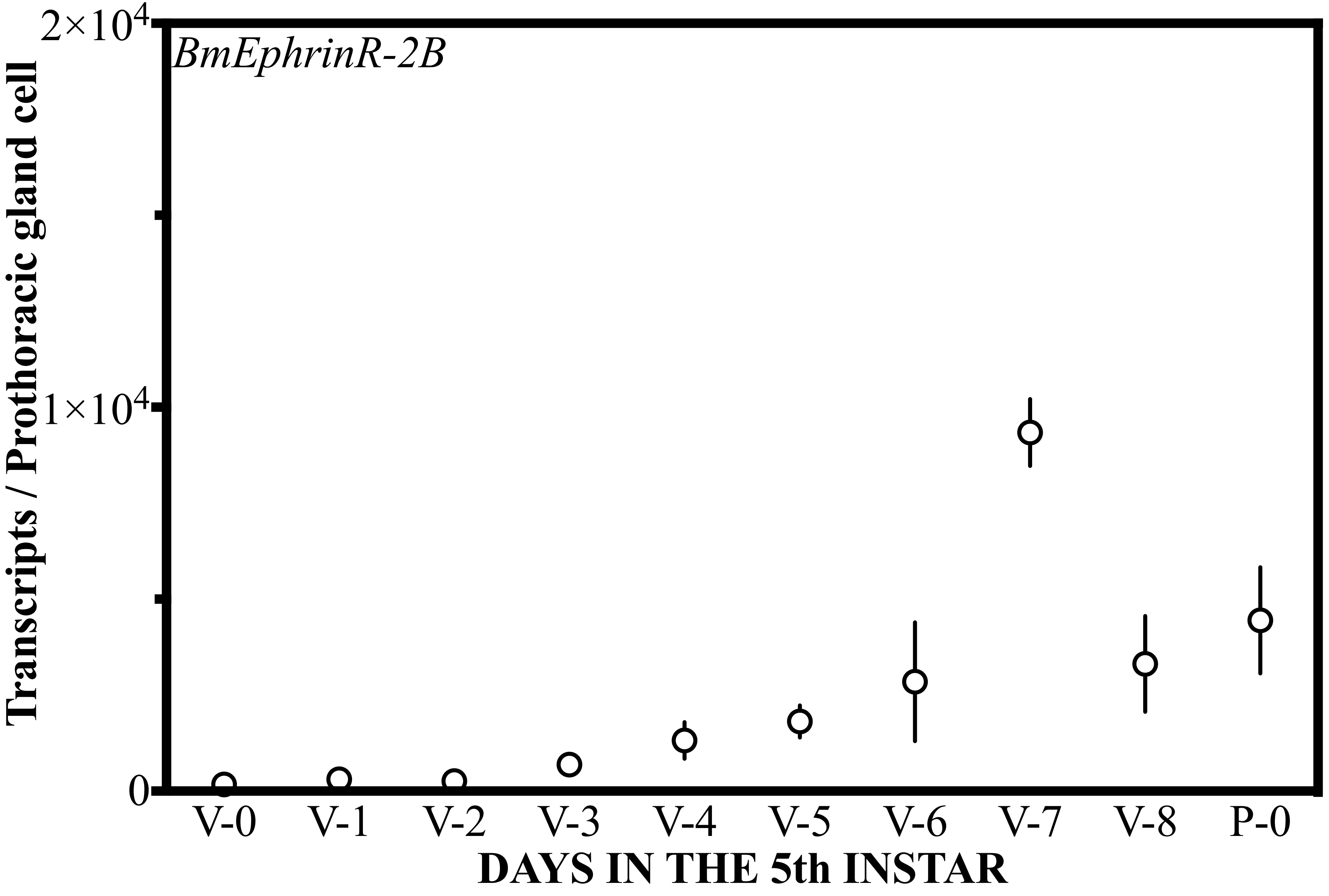
BmEphrinR-2B
2×10^4
1×10^4
0
Transcripts / Prothoracic gland cell
V-0
V-1
V-2
V-3
V-4
V-5
V-6
V-7
V-8
P-0
DAYS IN THE 5th INSTAR

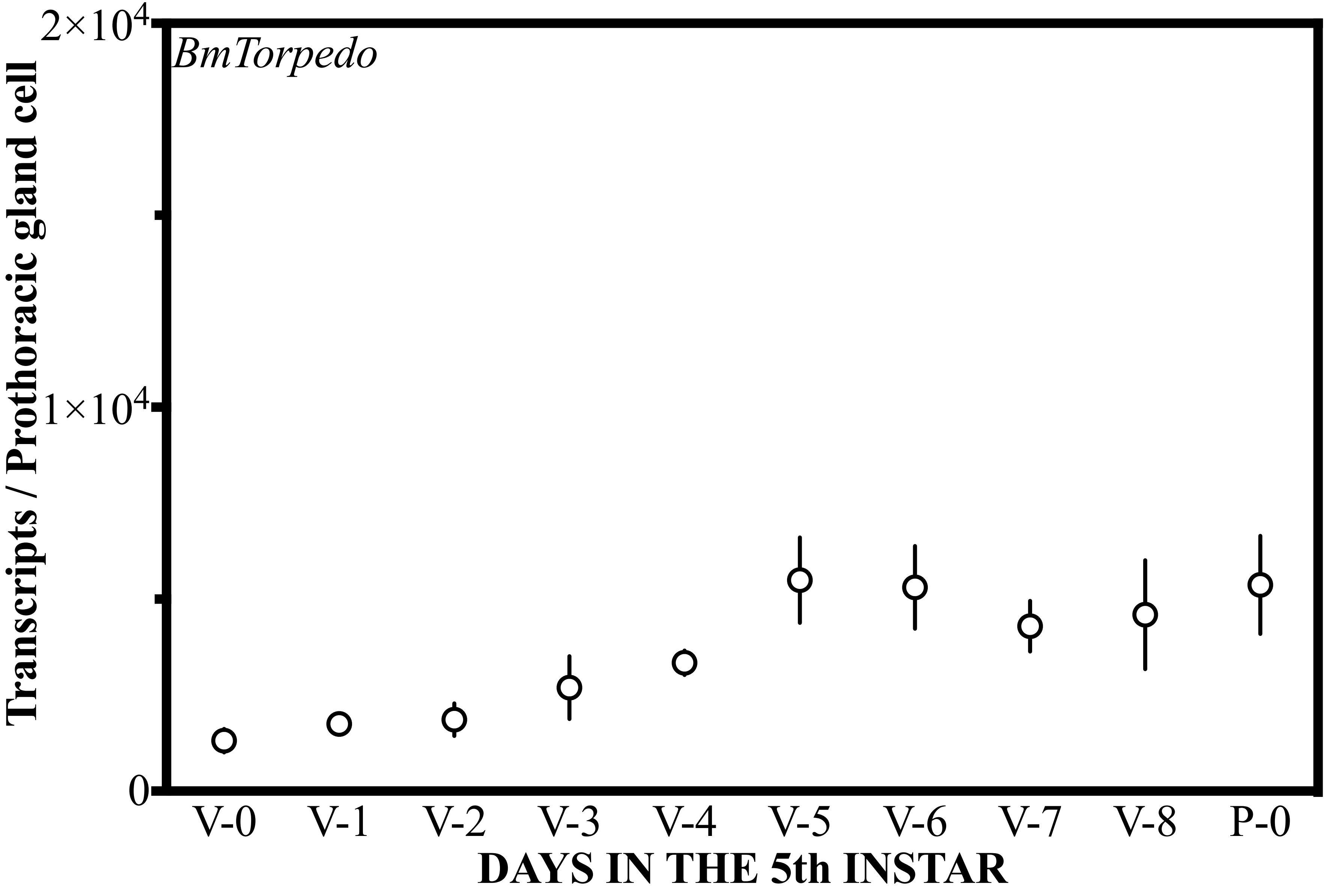

BmTorpedo
2×10^4
1×10^4
0
Transcripts / Prothoracic gland cell
V-0
V-1
V-2
V-3
V-4
V-5
V-6
V-7
V-8
P-0
DAYS IN THE 5th INSTAR

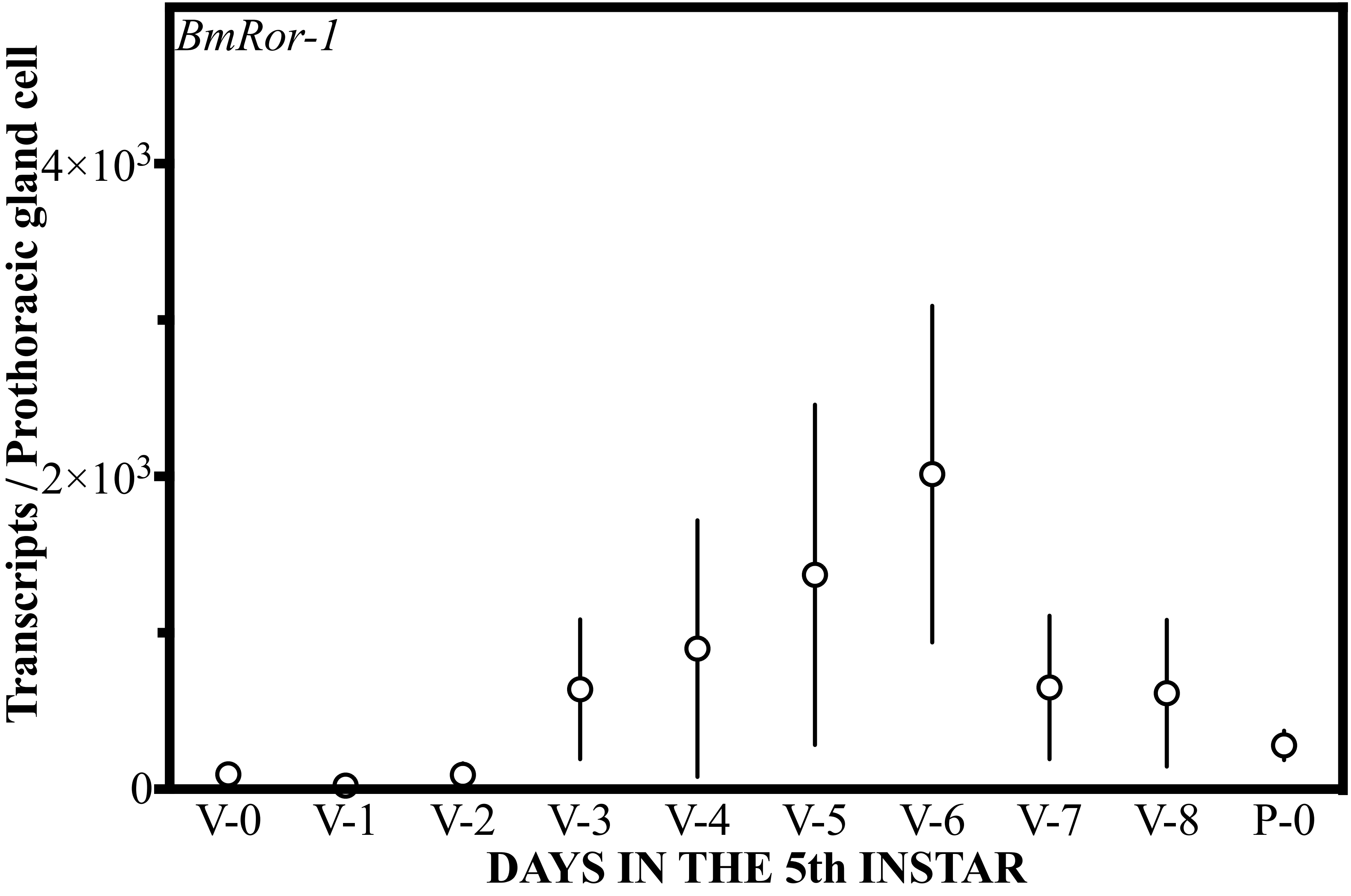

BmRor-1
Transcripts / Prothoracic gland cell
4×10³
2×10³
0
V-0
V-1
V-2
V-3
V-4
V-5
V-6
V-7
V-8
P-0
DAYS IN THE 5th INSTAR

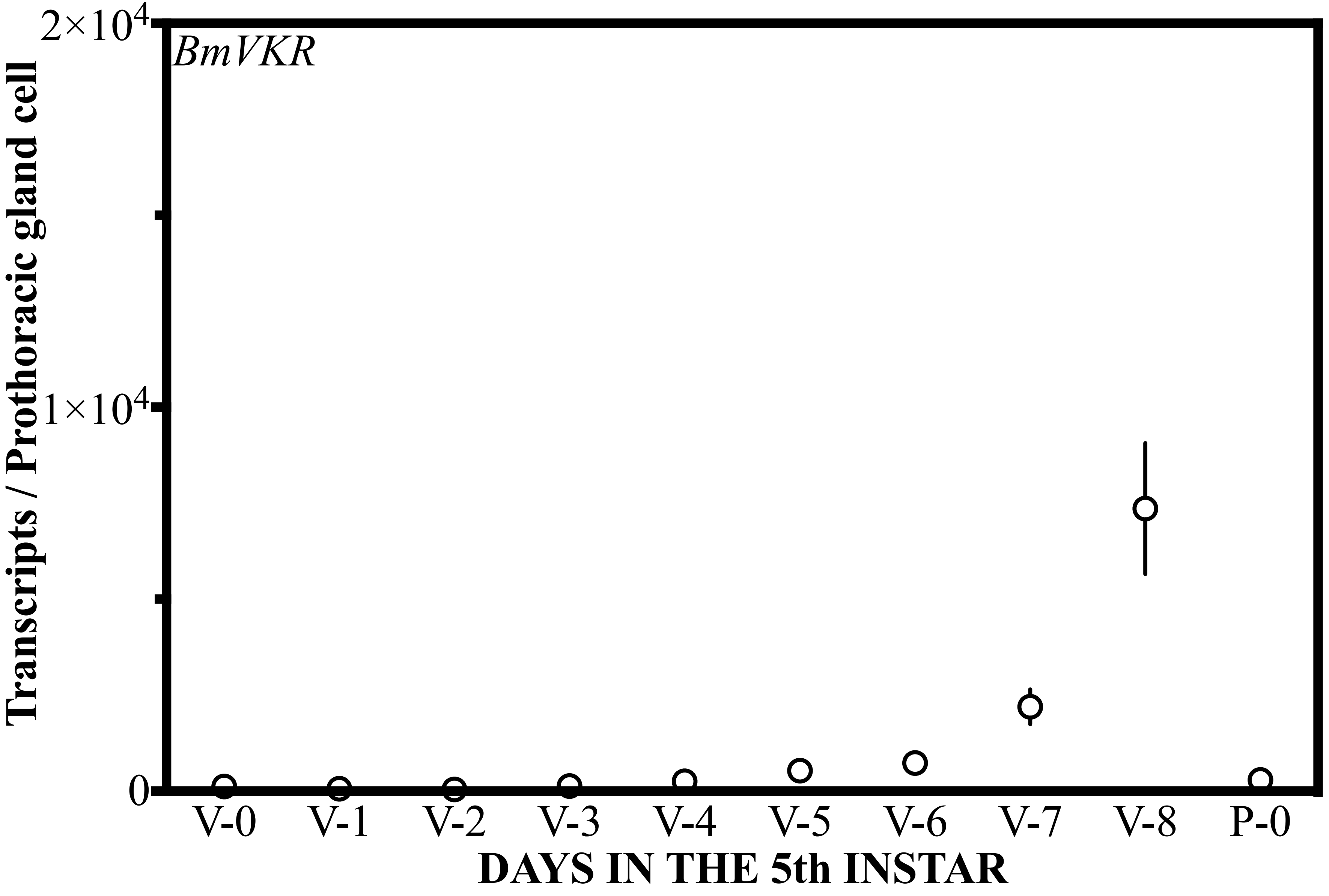

BmVKR
2×10^4
1×10^4
0
Transcripts / Prothoracic gland cell
V-0
V-1
V-2
V-3
V-4
V-5
V-6
V-7
V-8
P-0
DAYS IN THE 5th INSTAR

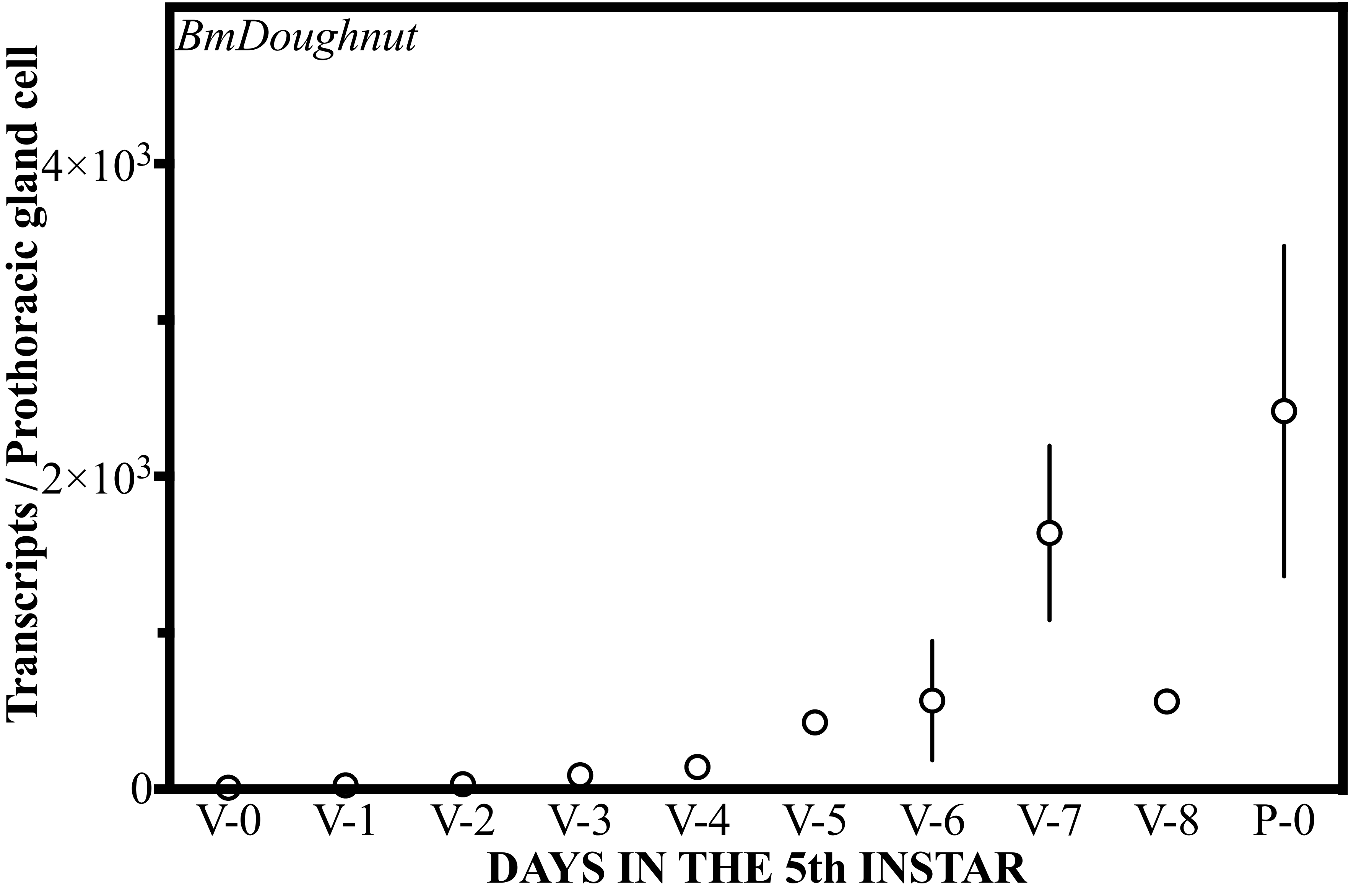
BmDoughnut
Transcripts / Prothoracic gland cell
4×10³
2×10³
0
V-0
V-1
V-2
V-3
V-4
V-5
V-6
V-7
V-8
P-0
DAYS IN THE 5th INSTAR

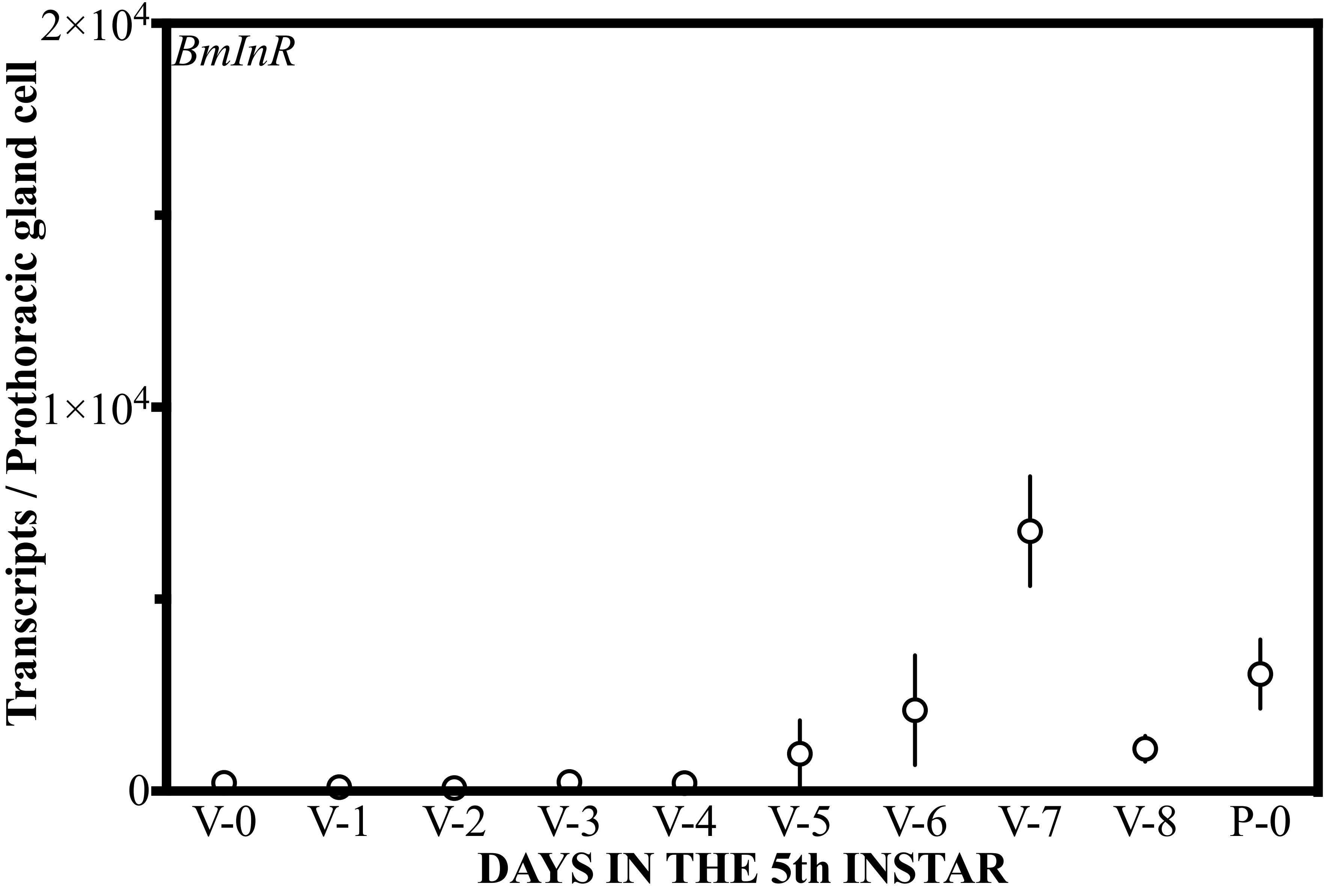

BmInR
Transcripts / Prothoracic gland cell
2×10^4
1×10^4
0
V-0
V-1
V-2
V-3
V-4
V-5
V-6
V-7
V-8
P-0
DAYS IN THE 5th INSTAR

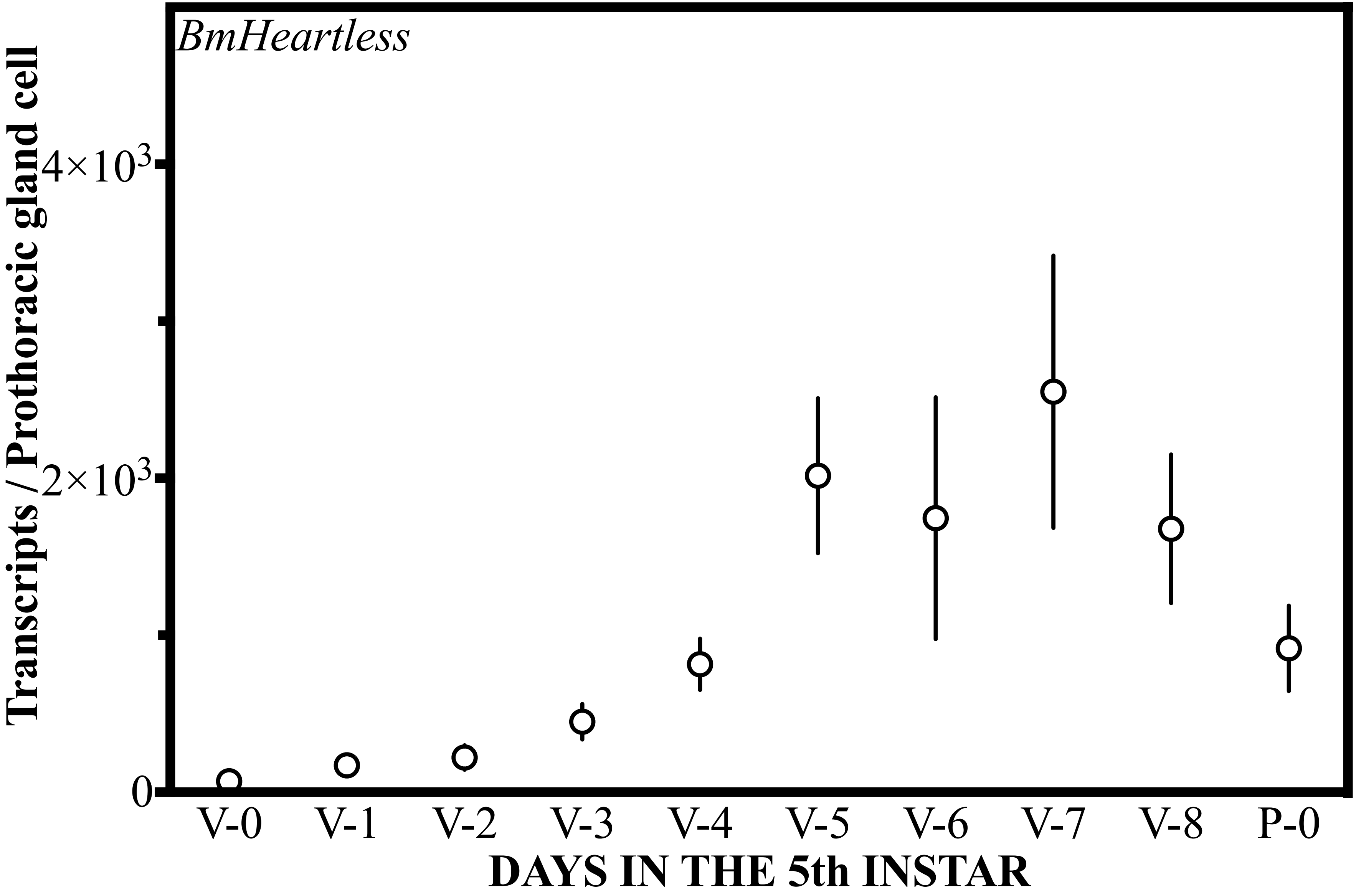
BmHeartless
Transcripts / Prothoracic gland cell
4×10³
2×10³
0
V-0
V-1
V-2
V-3
V-4
V-5
V-6
V-7
V-8
P-0
DAYS IN THE 5th INSTAR

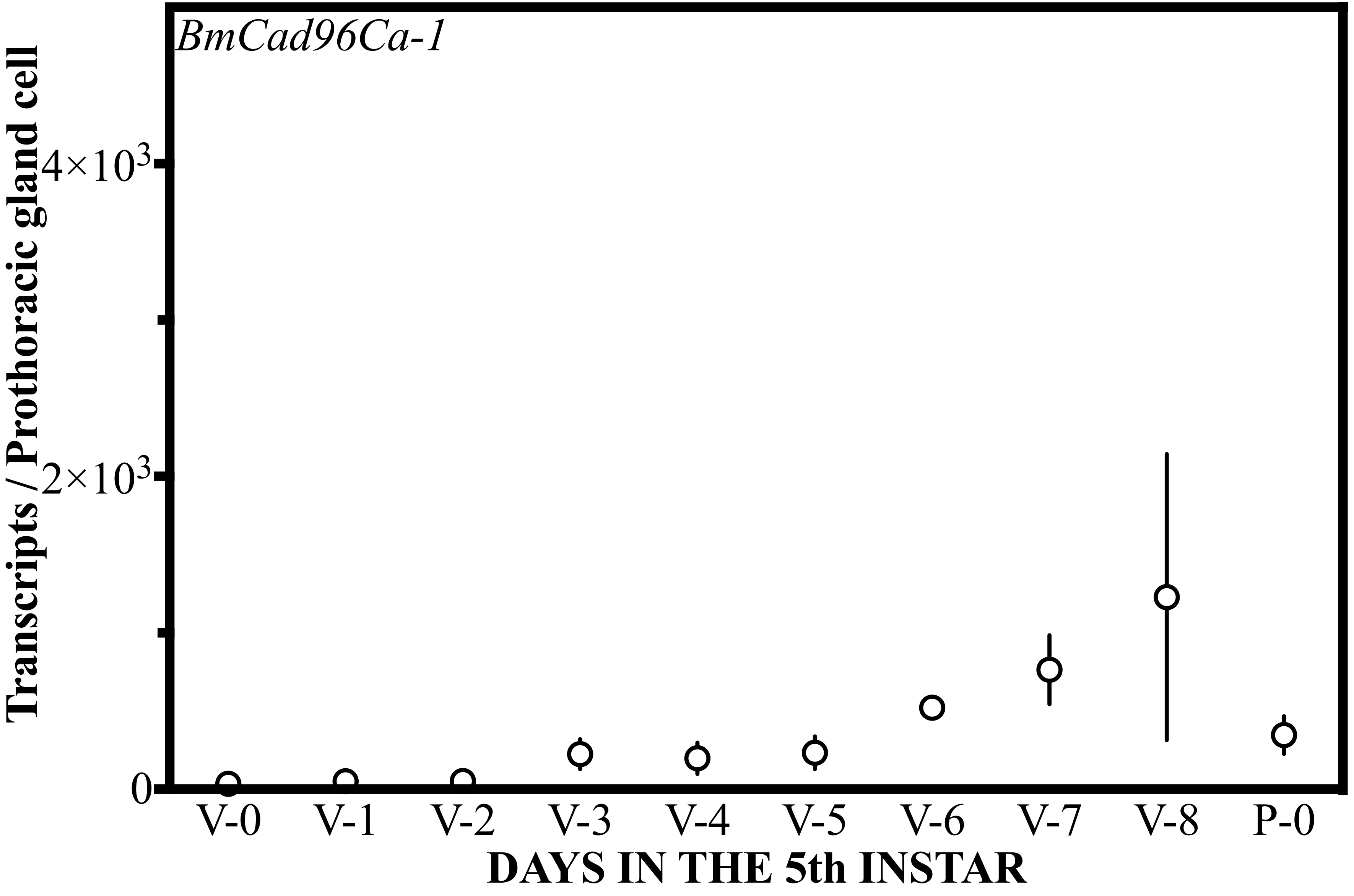

BmCad96Ca-1
Transcripts / Prothoracic gland cell
4×10³
2×10³
0
V-0
V-1
V-2
V-3
V-4
V-5
V-6
V-7
V-8
P-0
DAYS IN THE 5th INSTAR

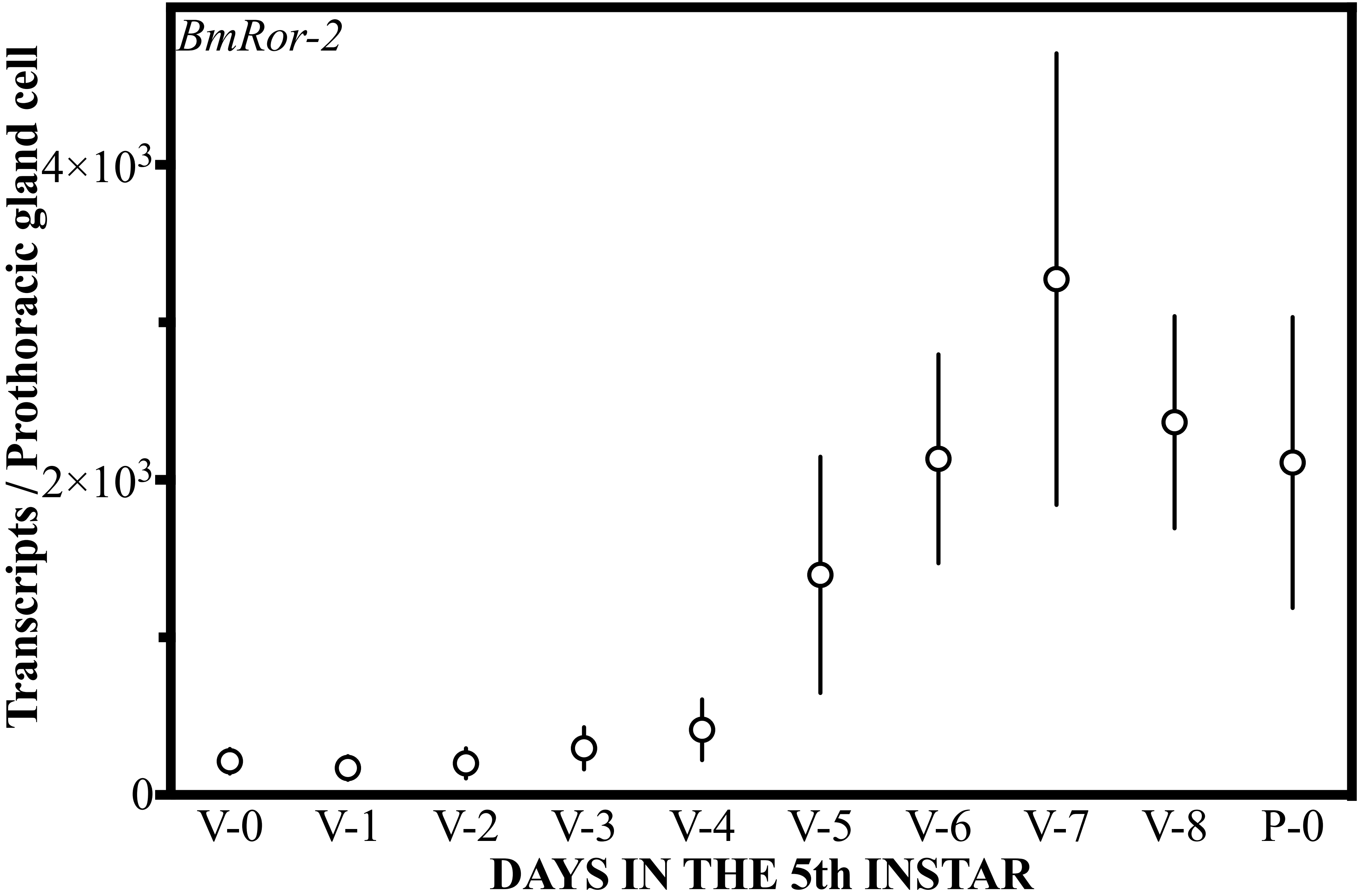

BmRor-2
Transcripts / Prothoracic gland cell
4×10³
2×10³
0
V-0
V-1
V-2
V-3
V-4
V-5
V-6
V-7
V-8
P-0
DAYS IN THE 5th INSTAR

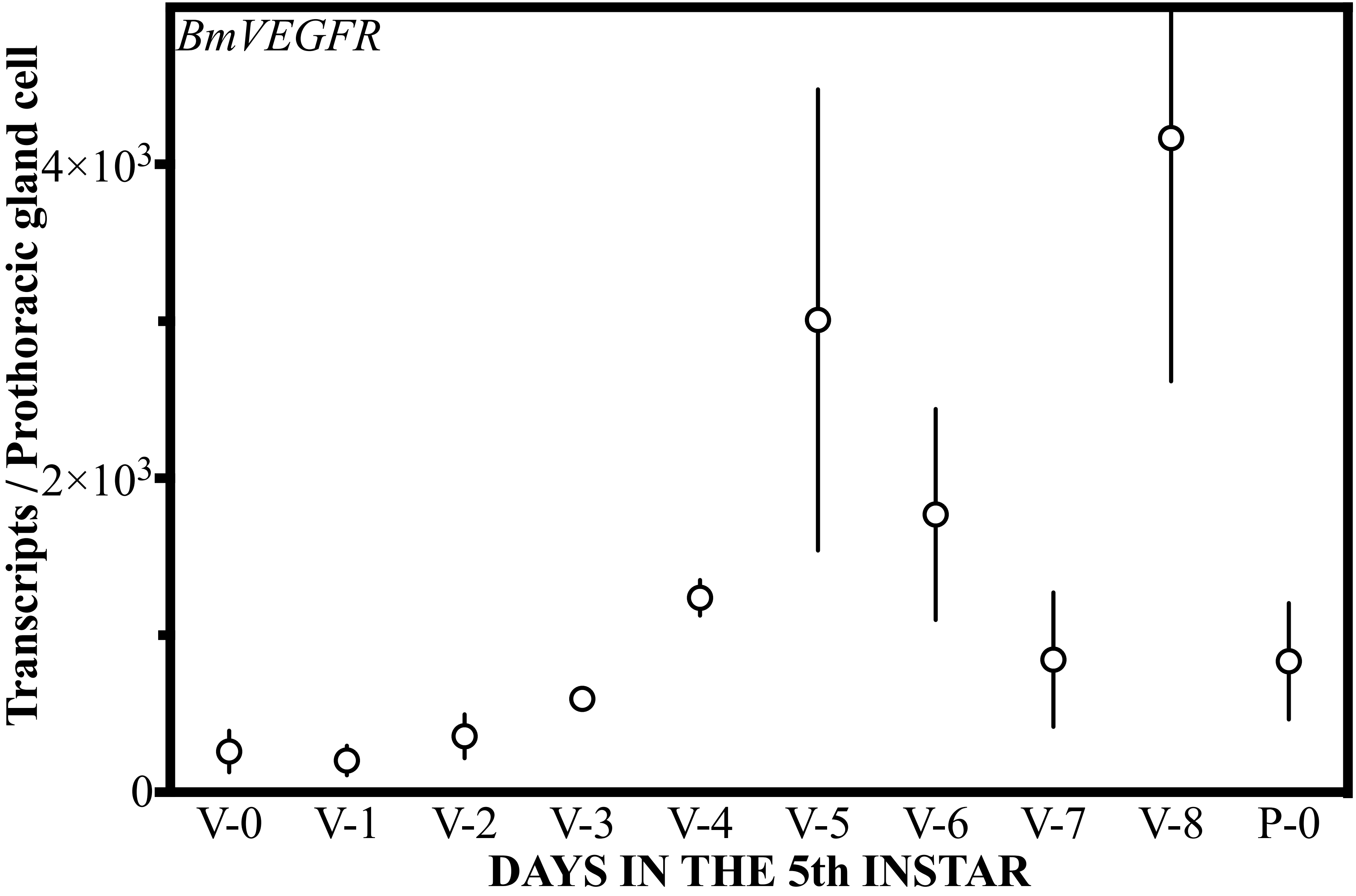

BmVEGFR
Transcripts / Prothoracic gland cell
4×10^3
2×10^3
0
V-0
V-1
V-2
V-3
V-4
V-5
V-6
V-7
V-8
P-0
DAYS IN THE 5th INSTAR

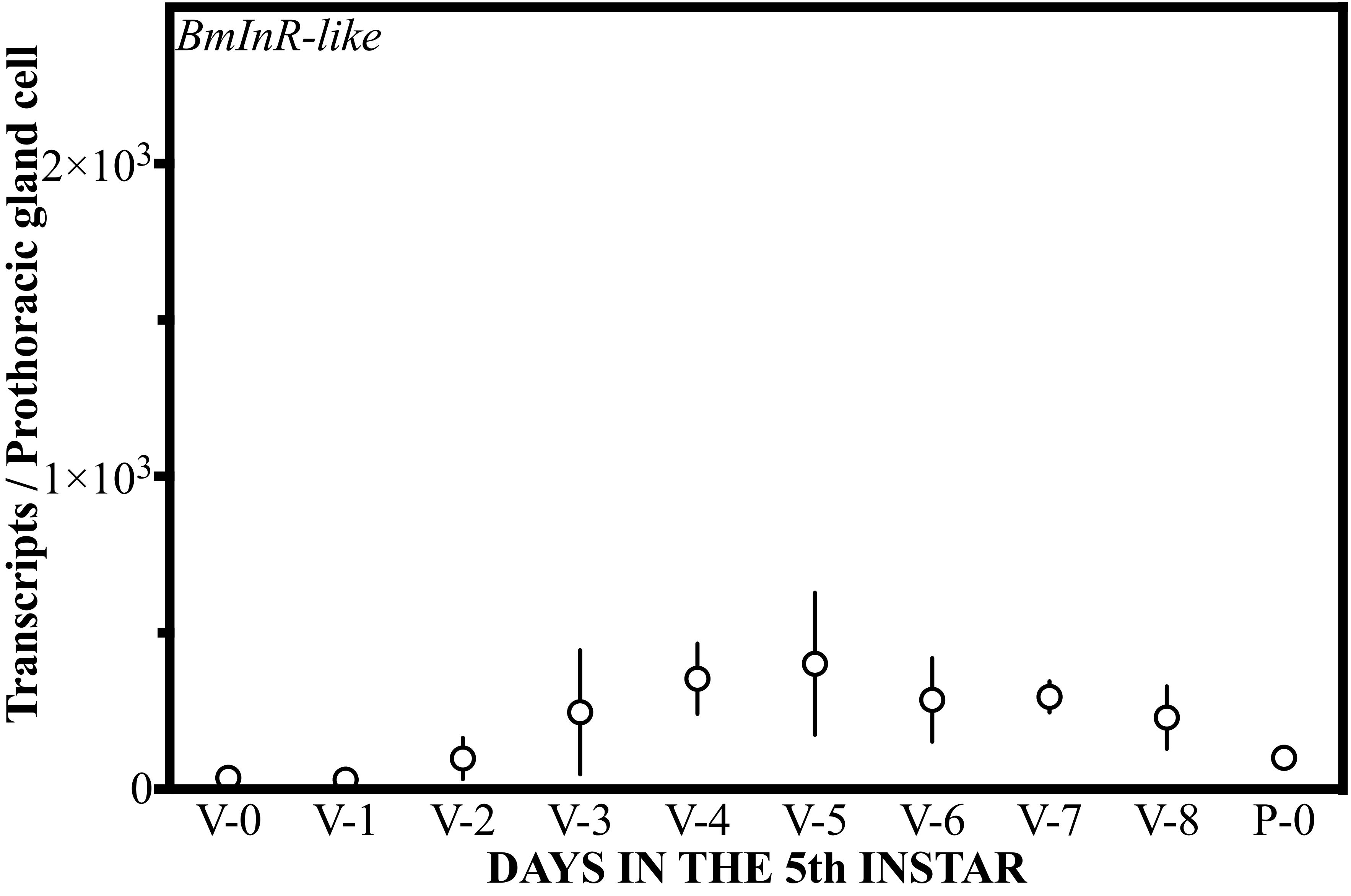

BmInR-like
Transcripts / Prothoracic gland cell
2×10³
1×10³
0
V-0
V-1
V-2
V-3
V-4
V-5
V-6
V-7
V-8
P-0
DAYS IN THE 5th INSTAR

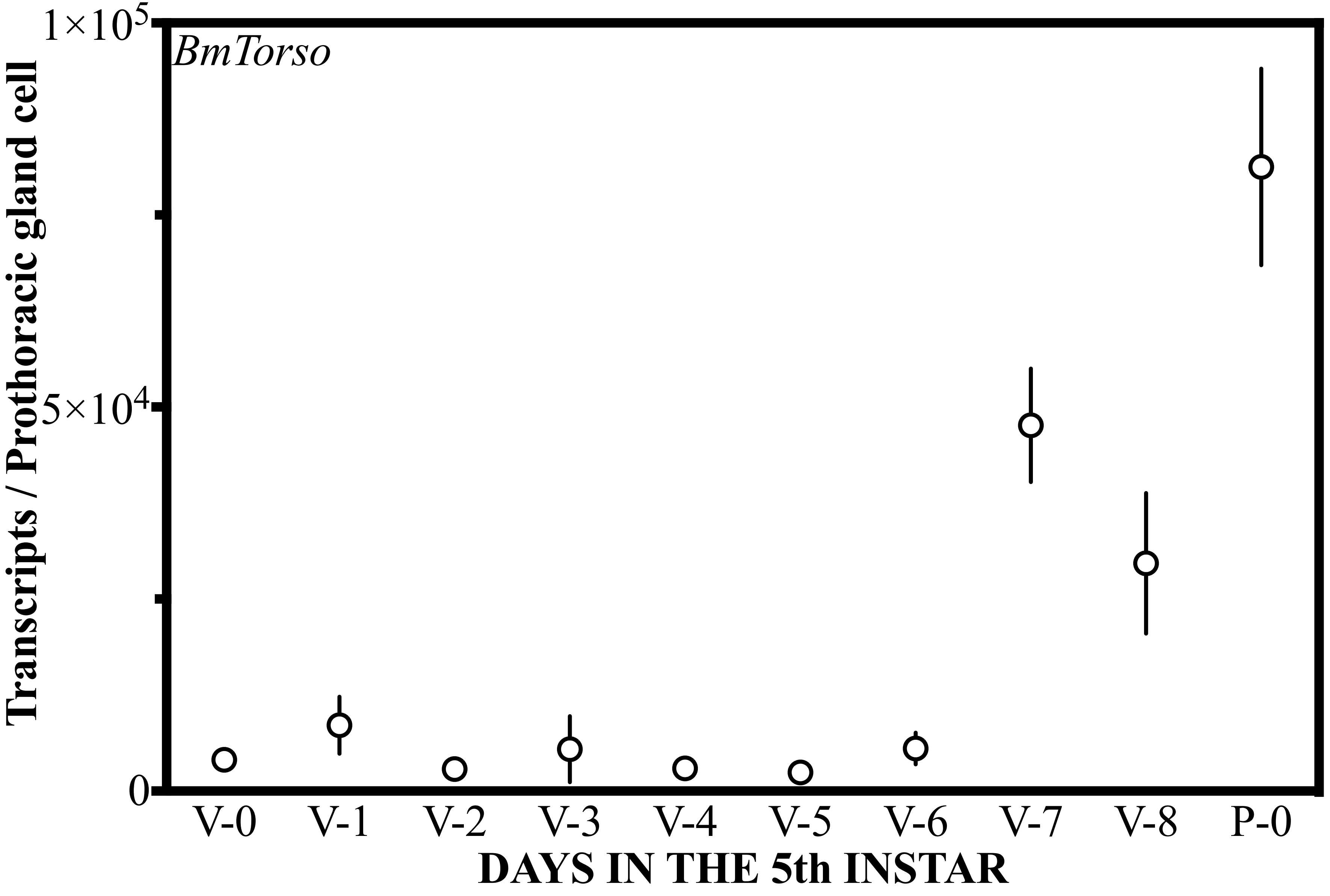
1×10^5
5×10^4
0
BmTorso
Transcripts / Prothoracic gland cell
V-0
V-1
V-2
V-3
V-4
V-5
V-6
V-7
V-8
P-0
DAYS IN THE 5th INSTAR

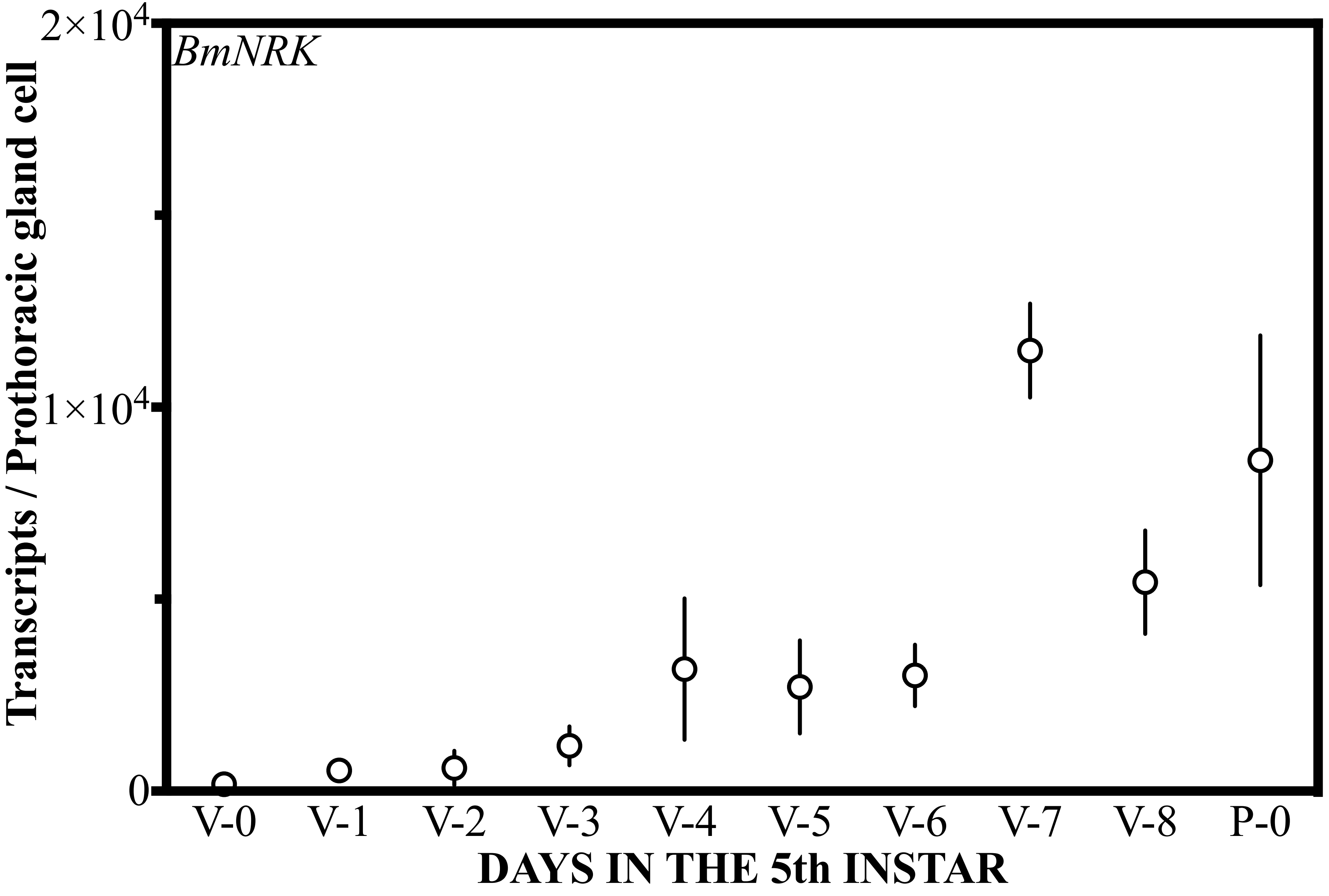

BmNRK
2×10^4
1×10^4
0
Transcripts / Prothoracic gland cell
V-0
V-1
V-2
V-3
V-4
V-5
V-6
V-7
V-8
P-0
DAYS IN THE 5th INSTAR

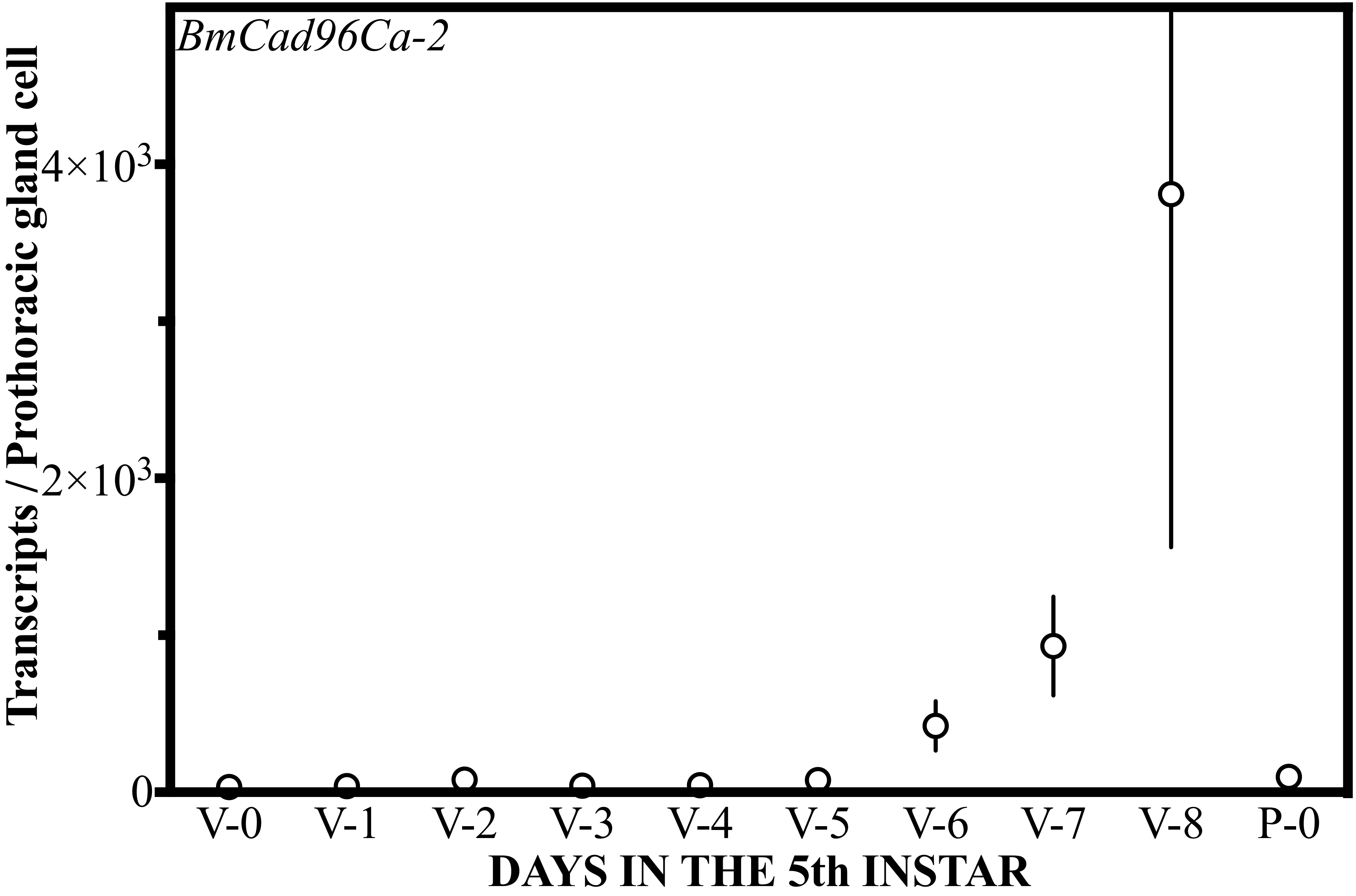

BmCad96Ca-2
Transcripts / Prothoracic gland cell
4×10³
2×10³
0
V-0
V-1
V-2
V-3
V-4
V-5
V-6
V-7
V-8
P-0
DAYS IN THE 5th INSTAR

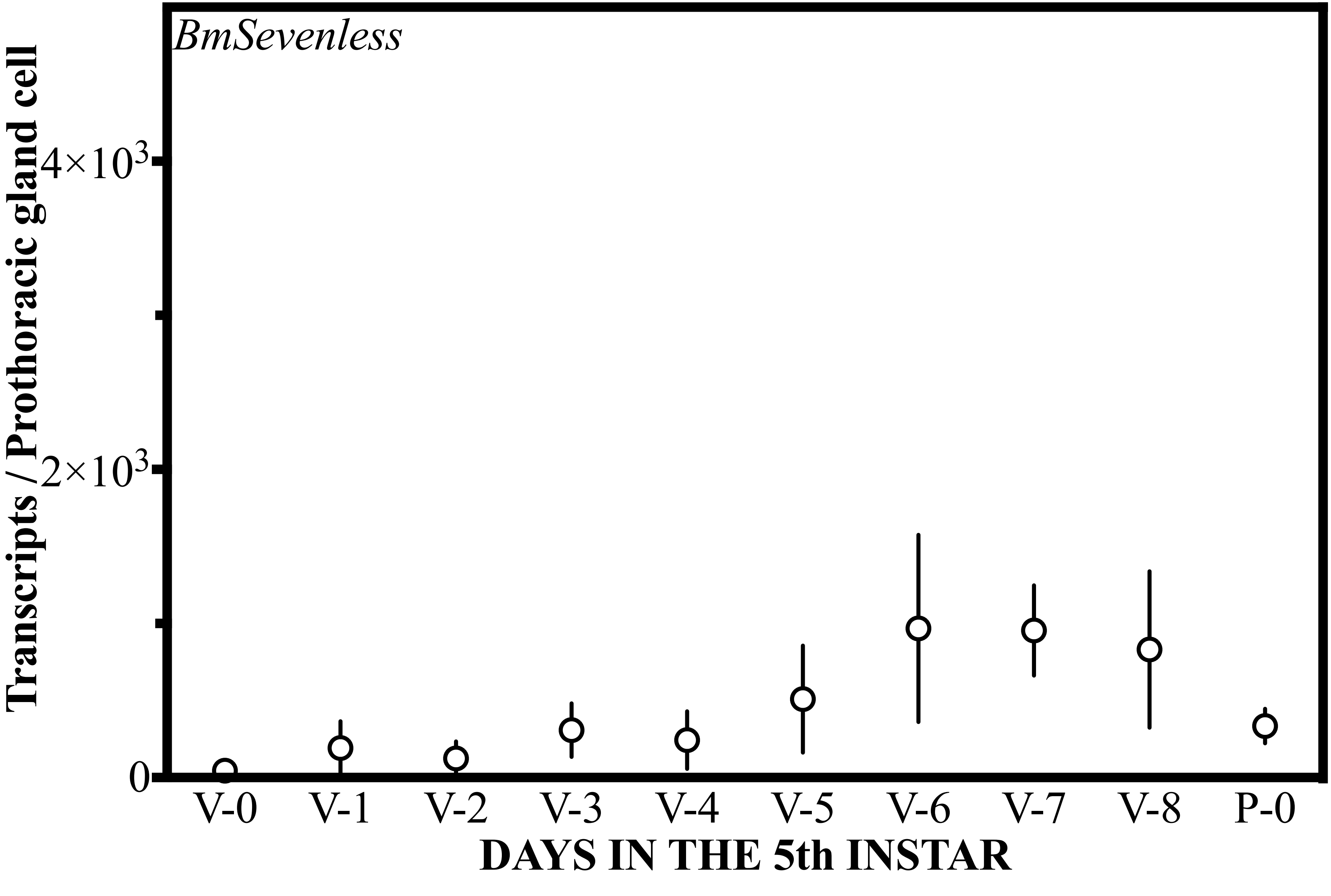
BmSevenless
Transcripts / Prothoracic gland cell
4×10³
2×10³
0
V-0
V-1
V-2
V-3
V-4
V-5
V-6
V-7
V-8
P-0
DAYS IN THE 5th INSTAR

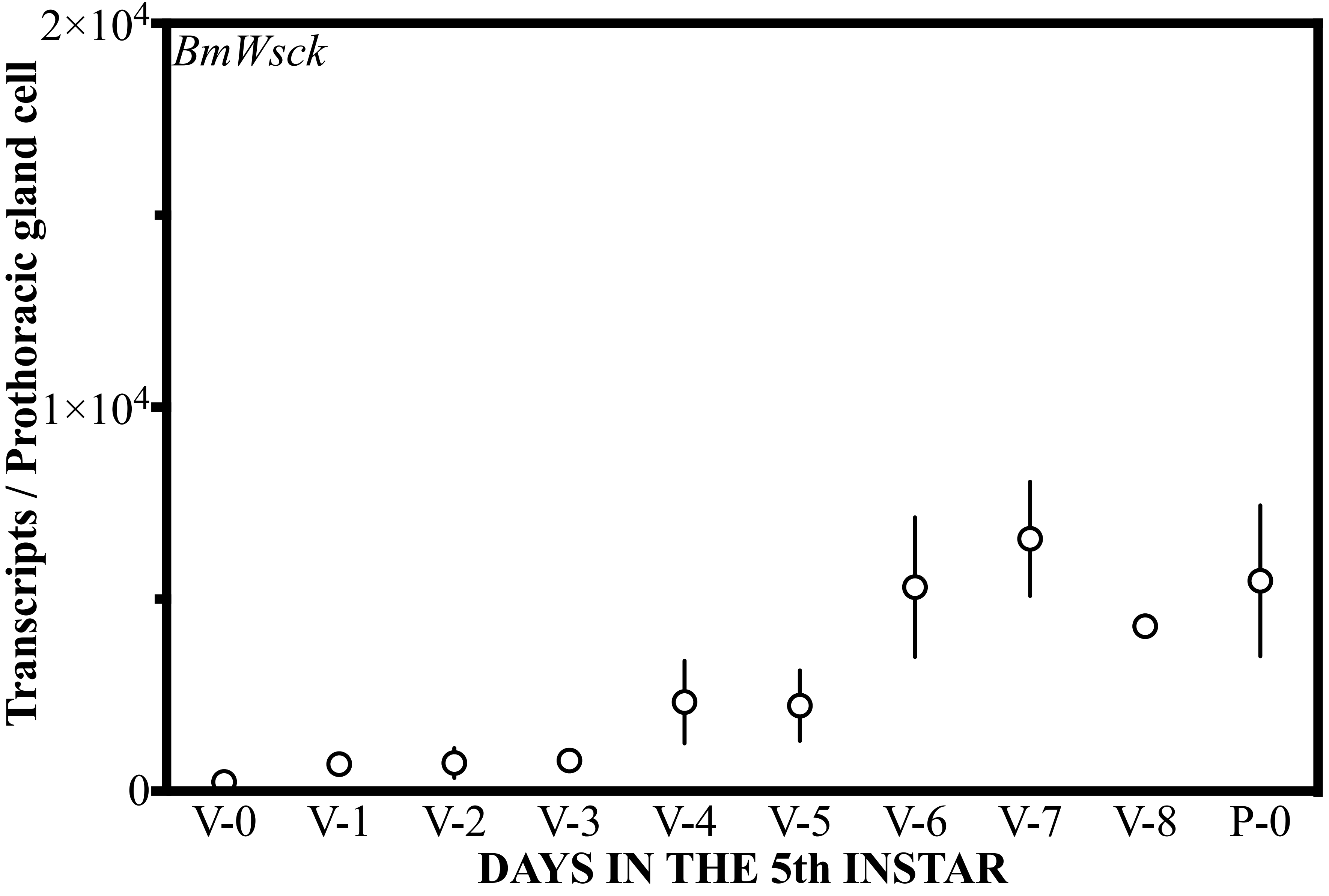
BmWsck
2×10^4
1×10^4
0
Transcripts / Prothoracic gland cell
V-0
V-1
V-2
V-3
V-4
V-5
V-6
V-7
V-8
P-0
DAYS IN THE 5th INSTAR

RTSKs

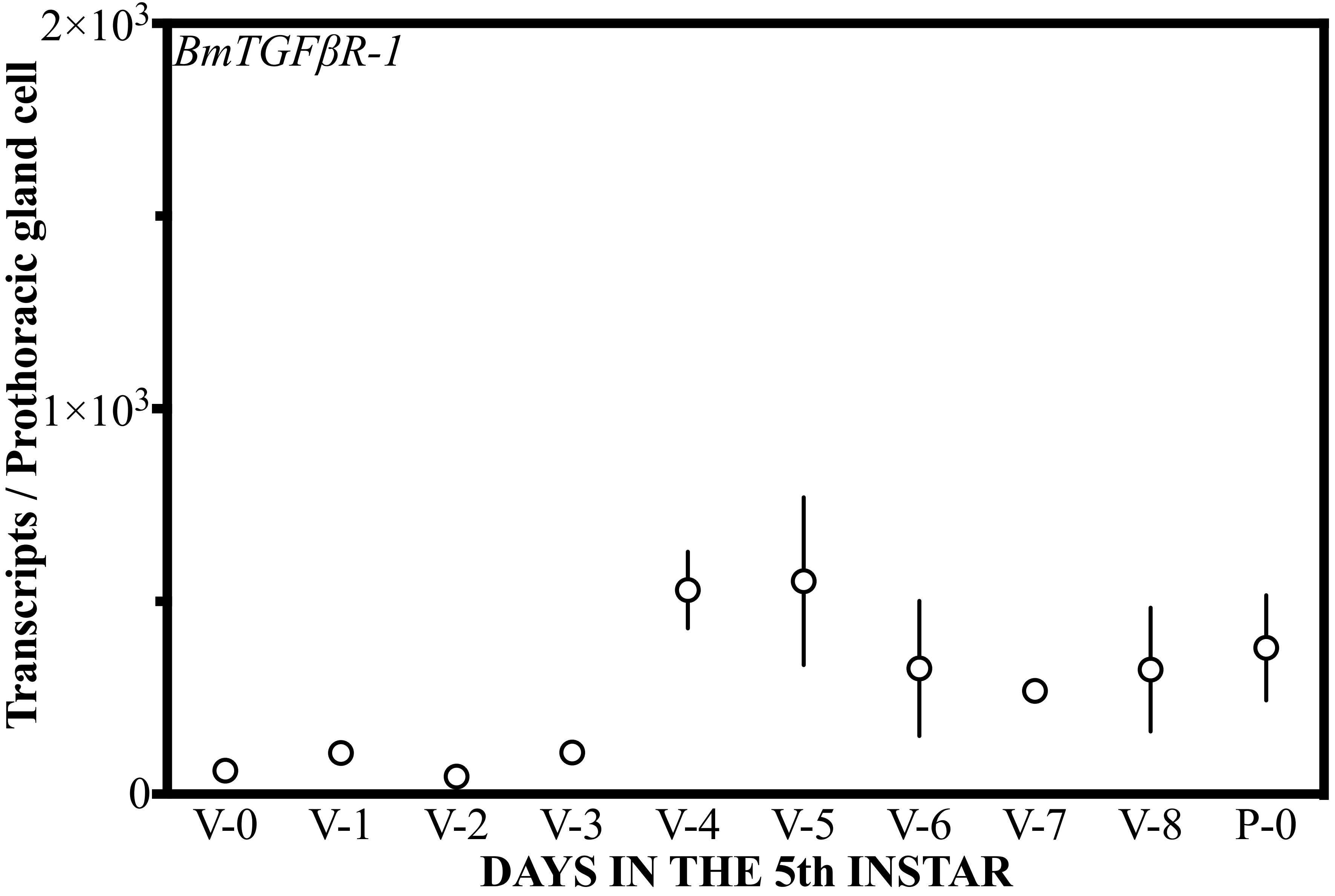

BmTGFβR-1
2×10^3
1×10^3
0
Transcripts / Prothoracic gland cell
V-0
V-1
V-2
V-3
V-4
V-5
V-6
V-7
V-8
P-0
DAYS IN THE 5th INSTAR

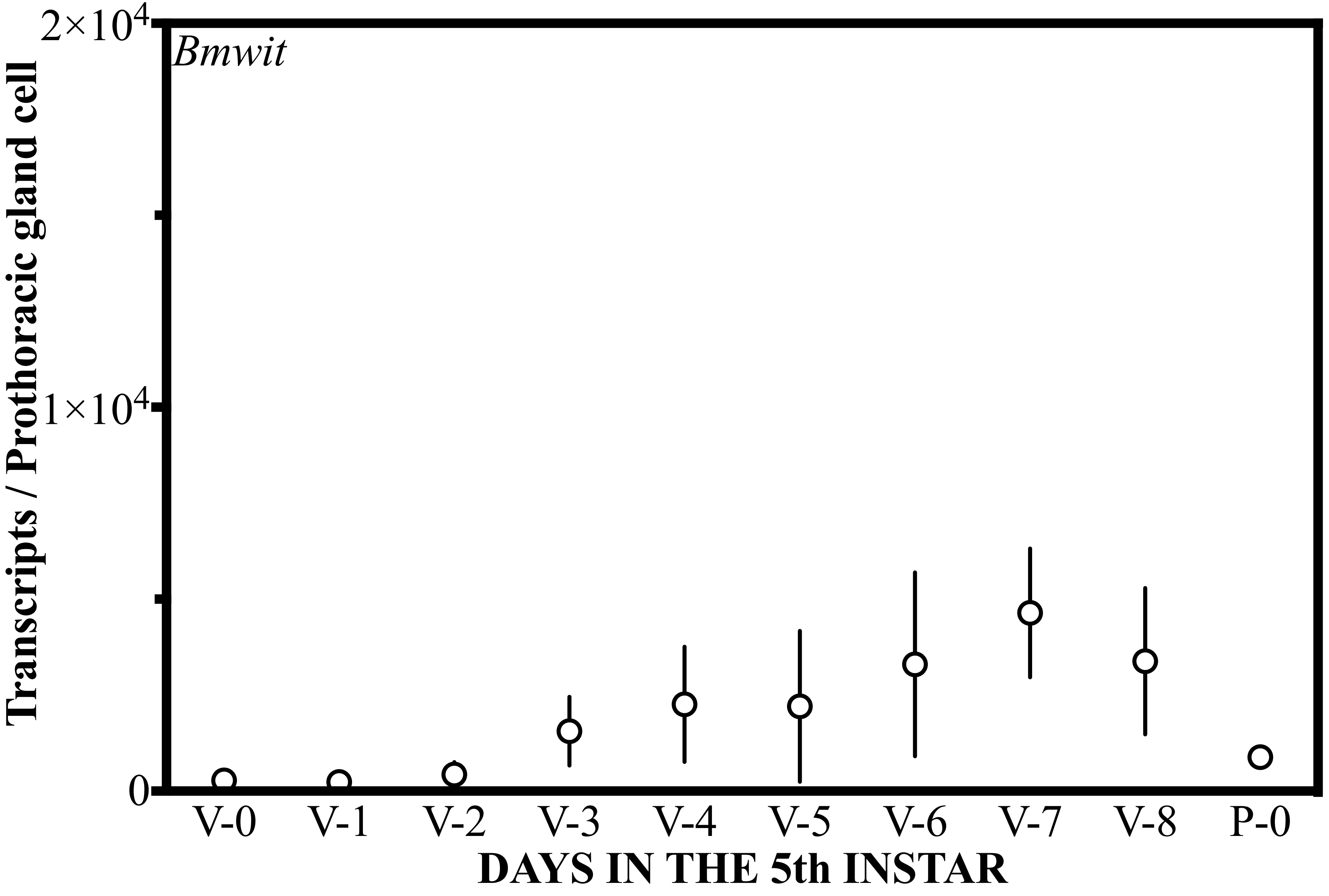
2×10^4
Bmwit
1×10^4
0
Transcripts / Prothoracic gland cell
V-0
V-1
V-2
V-3
V-4
V-5
V-6
V-7
V-8
P-0
DAYS IN THE 5th INSTAR

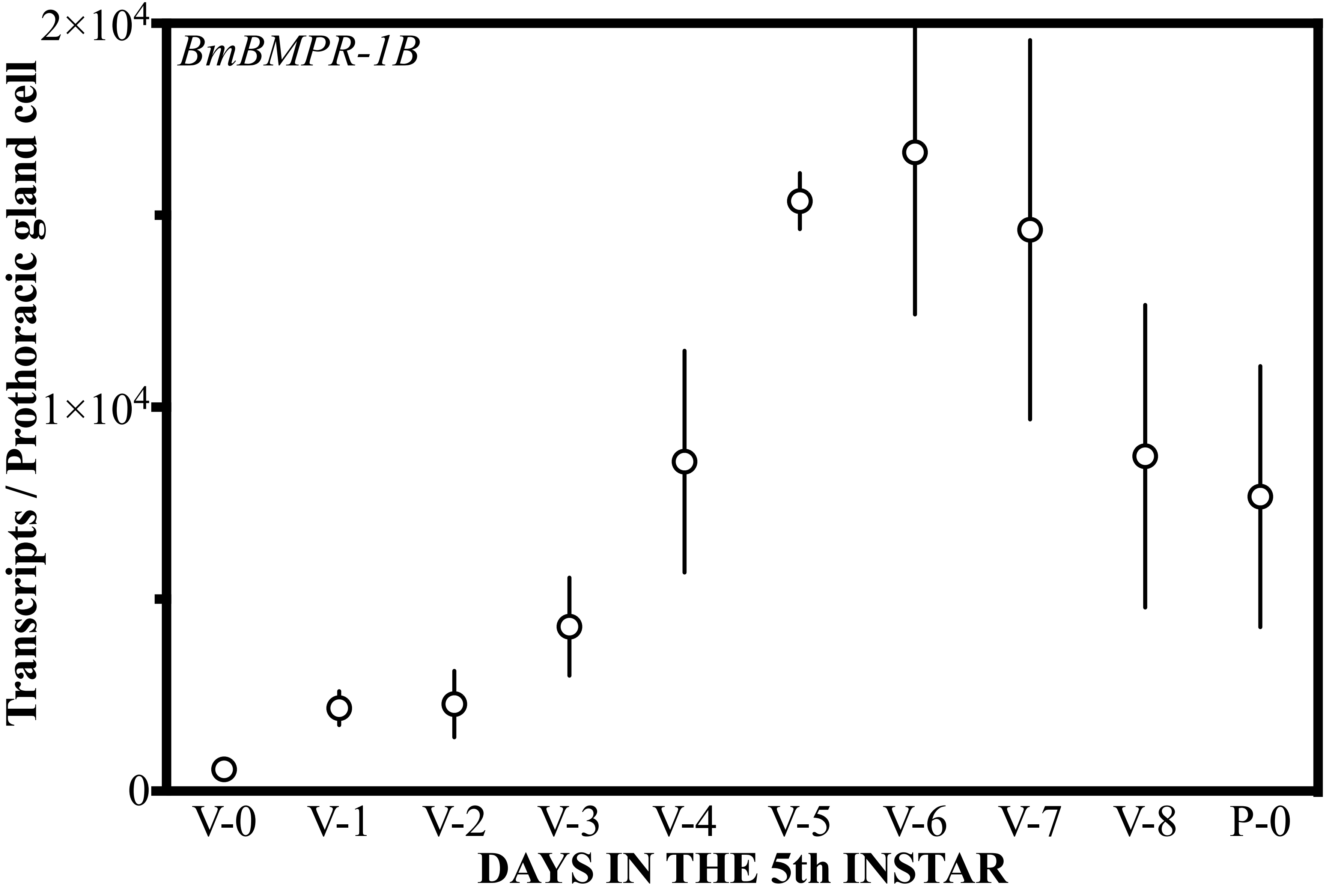

BmBMPR-1B
2×10⁴
1×10⁴
0
Transcripts / Prothoracic gland cell
V-0
V-1
V-2
V-3
V-4
V-5
V-6
V-7
V-8
P-0
DAYS IN THE 5th INSTAR

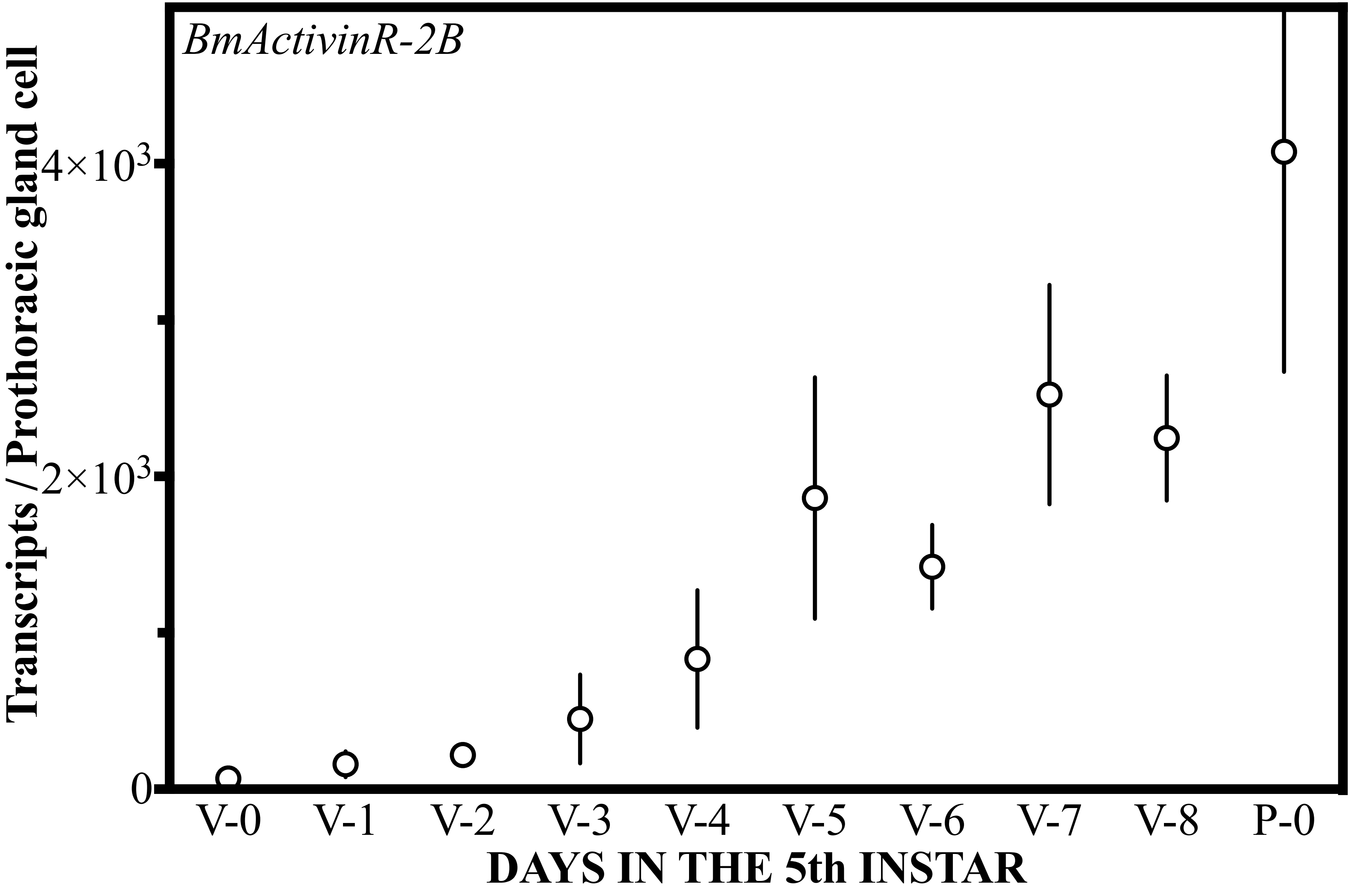

BmActivinR-2B
Transcripts / Prothoracic gland cell
4×10³
2×10³
0
V-0
V-1
V-2
V-3
V-4
V-5
V-6
V-7
V-8
P-0
DAYS IN THE 5th INSTAR

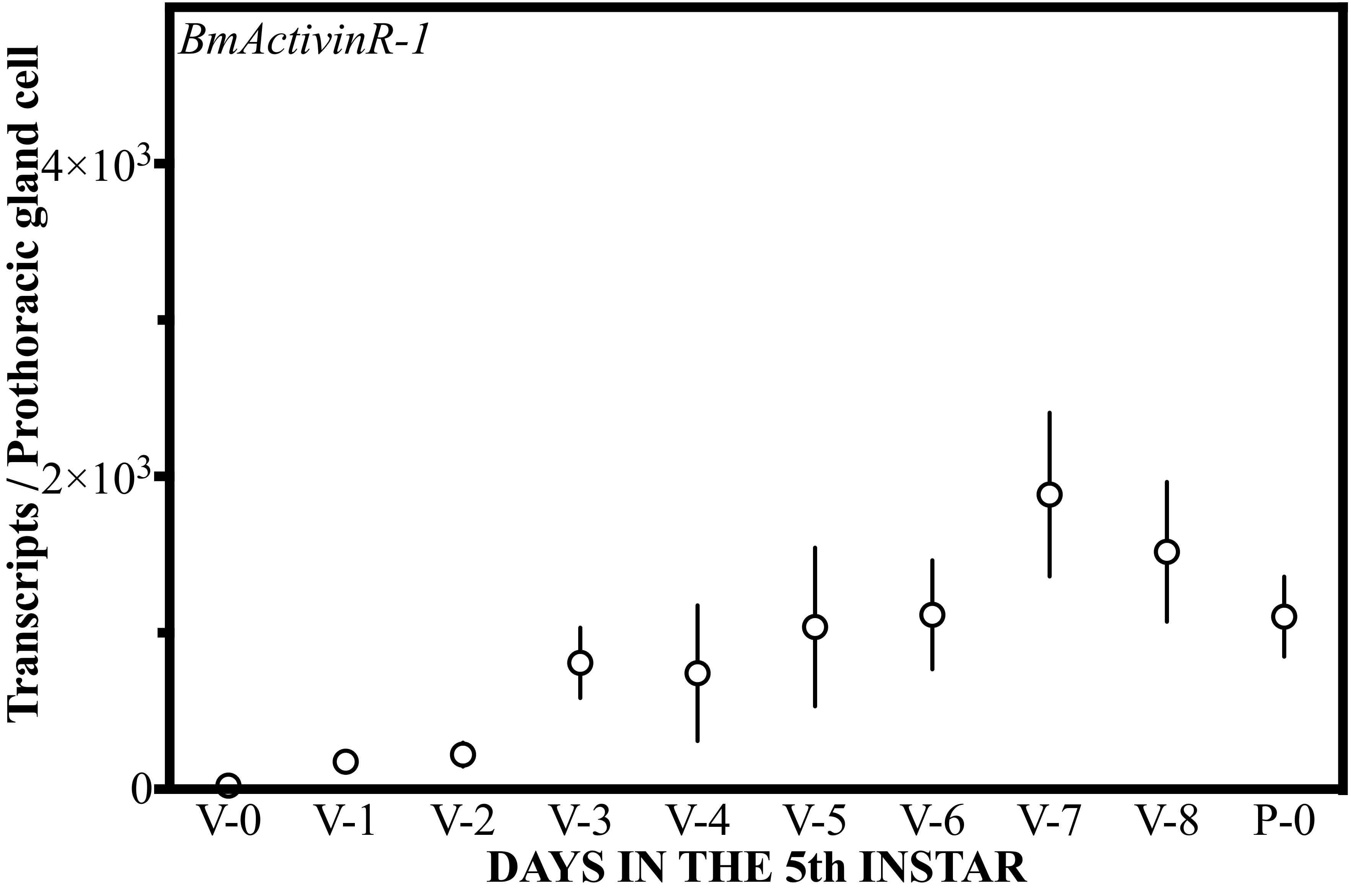

BmActivinR-1
Transcripts / Prothoracic gland cell
4×10³
2×10³
0
V-0
V-1
V-2
V-3
V-4
V-5
V-6
V-7
V-8
P-0
DAYS IN THE 5th INSTAR

RTPs

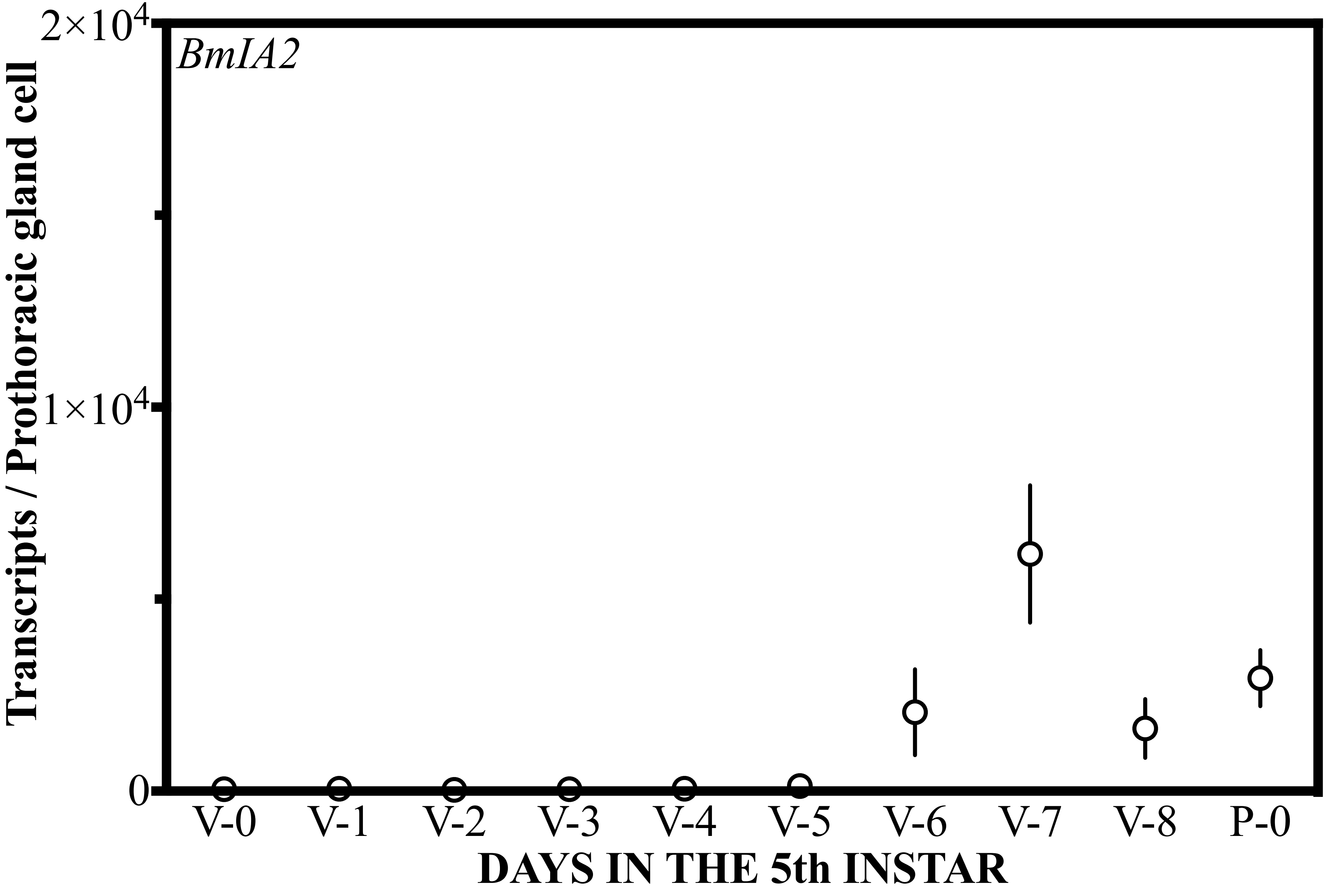

BmIA2
Transcripts / Prothoracic gland cell
2×10^4
1×10^4
0
V-0
V-1
V-2
V-3
V-4
V-5
V-6
V-7
V-8
P-0
DAYS IN THE 5th INSTAR

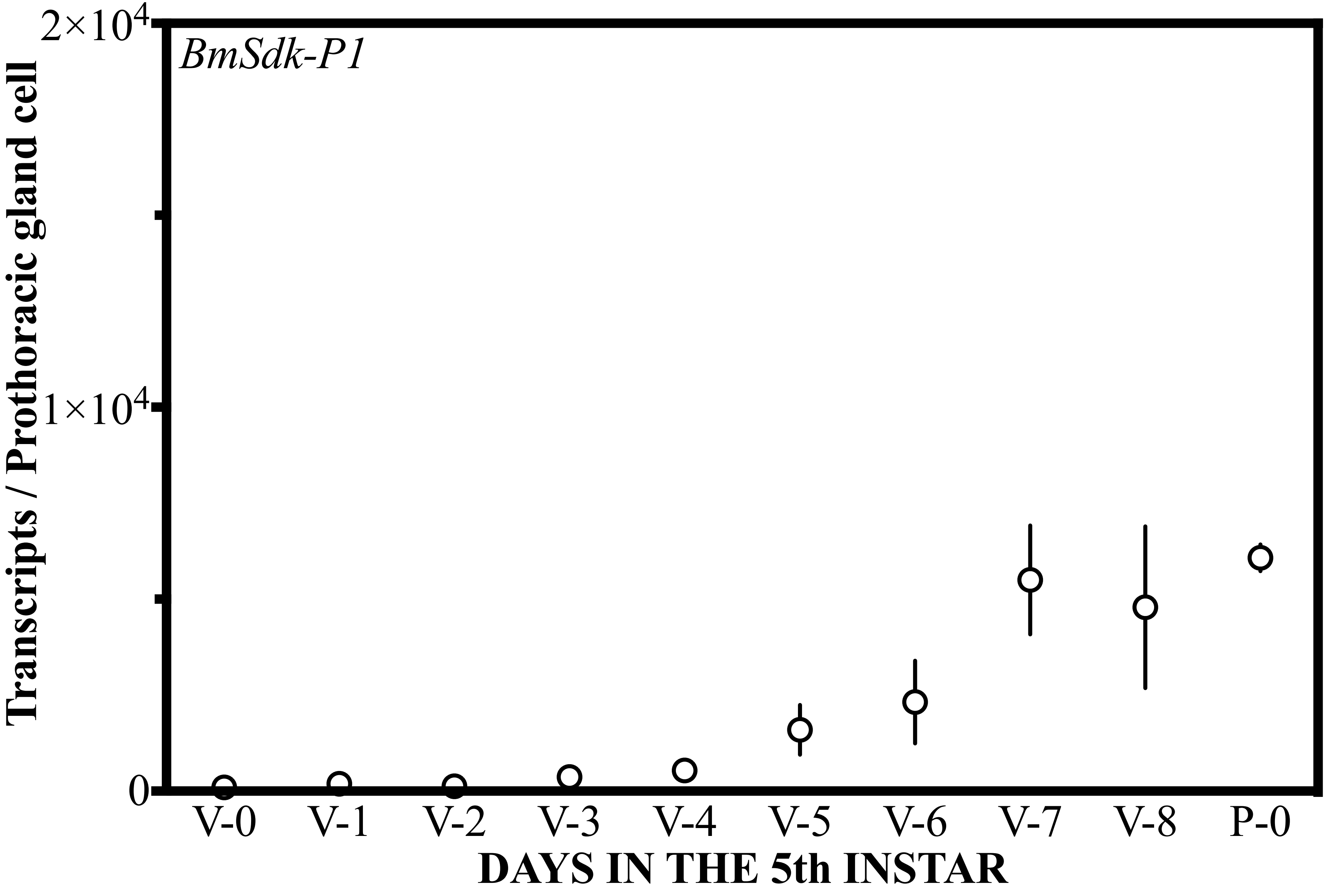
BmSdk-P1
2×10^4
1×10^4
0
Transcripts / Prothoracic gland cell
V-0
V-1
V-2
V-3
V-4
V-5
V-6
V-7
V-8
P-0
DAYS IN THE 5th INSTAR

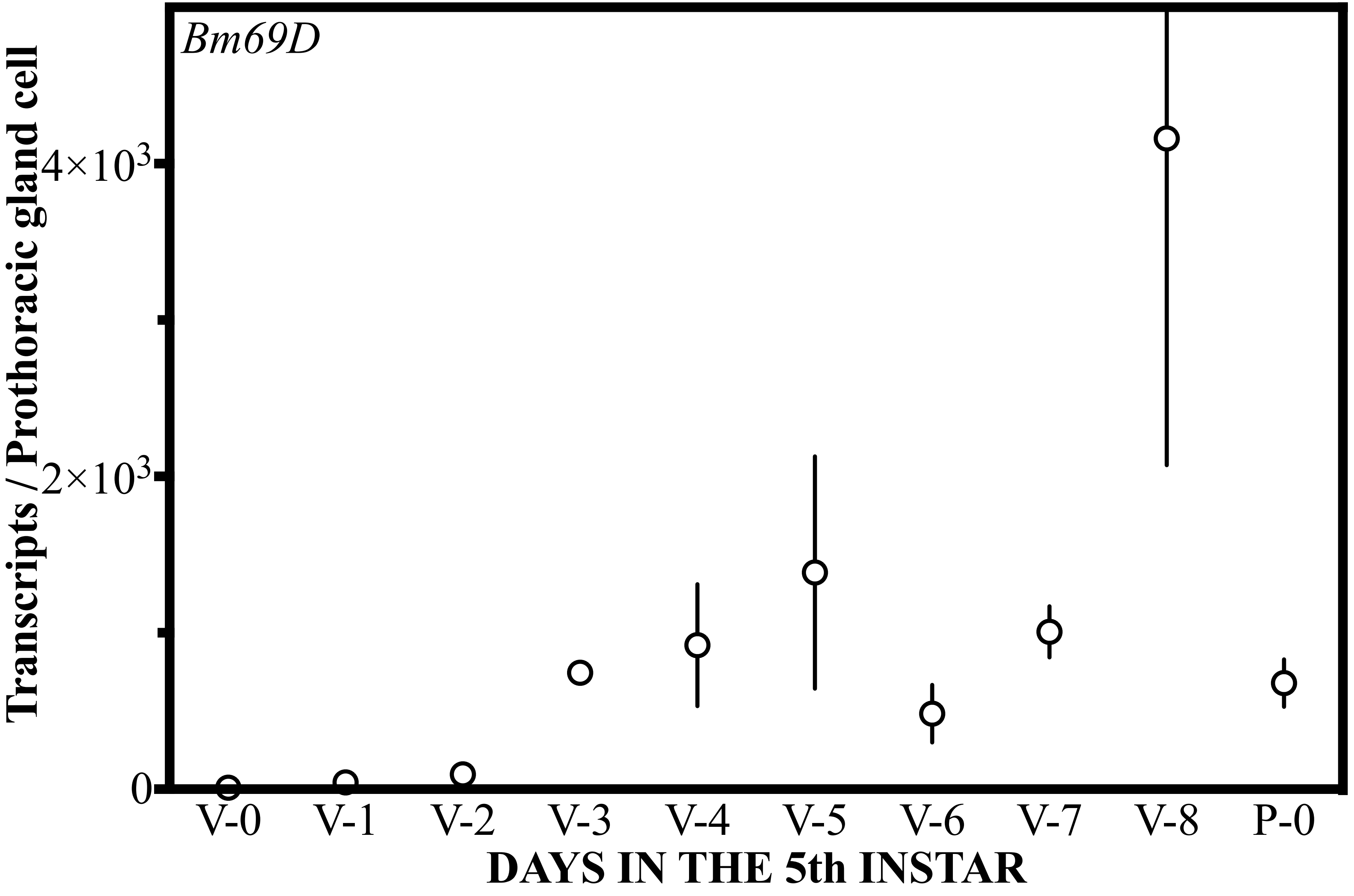

Bm69D
Transcripts / Prothoracic gland cell
4×10³
2×10³
0
V-0
V-1
V-2
V-3
V-4
V-5
V-6
V-7
V-8
P-0
DAYS IN THE 5th INSTAR

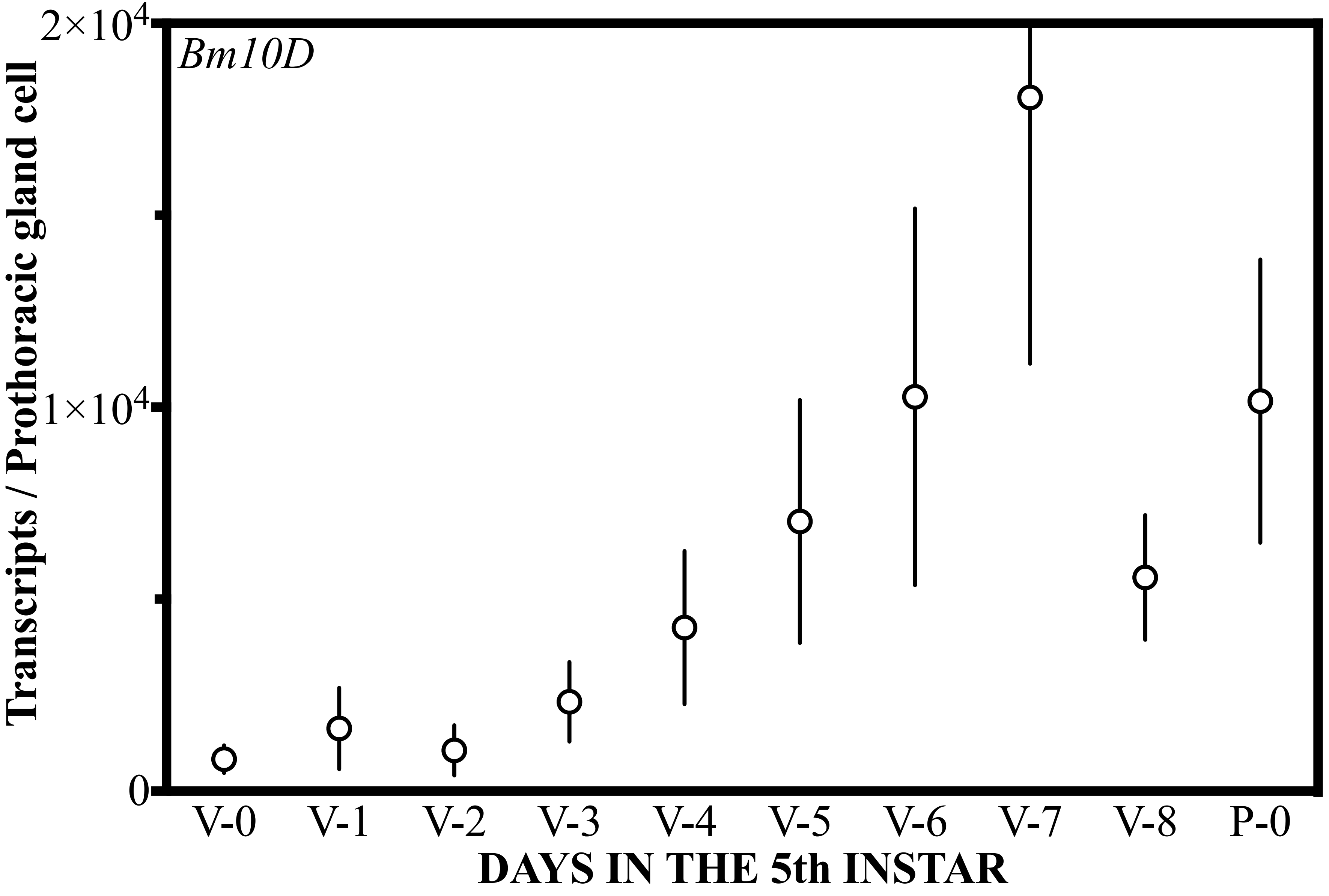

Bm10D
2×10^4
1×10^4
0
Transcripts / Prothoracic gland cell
V-0
V-1
V-2
V-3
V-4
V-5
V-6
V-7
V-8
P-0
DAYS IN THE 5th INSTAR

RTGCs

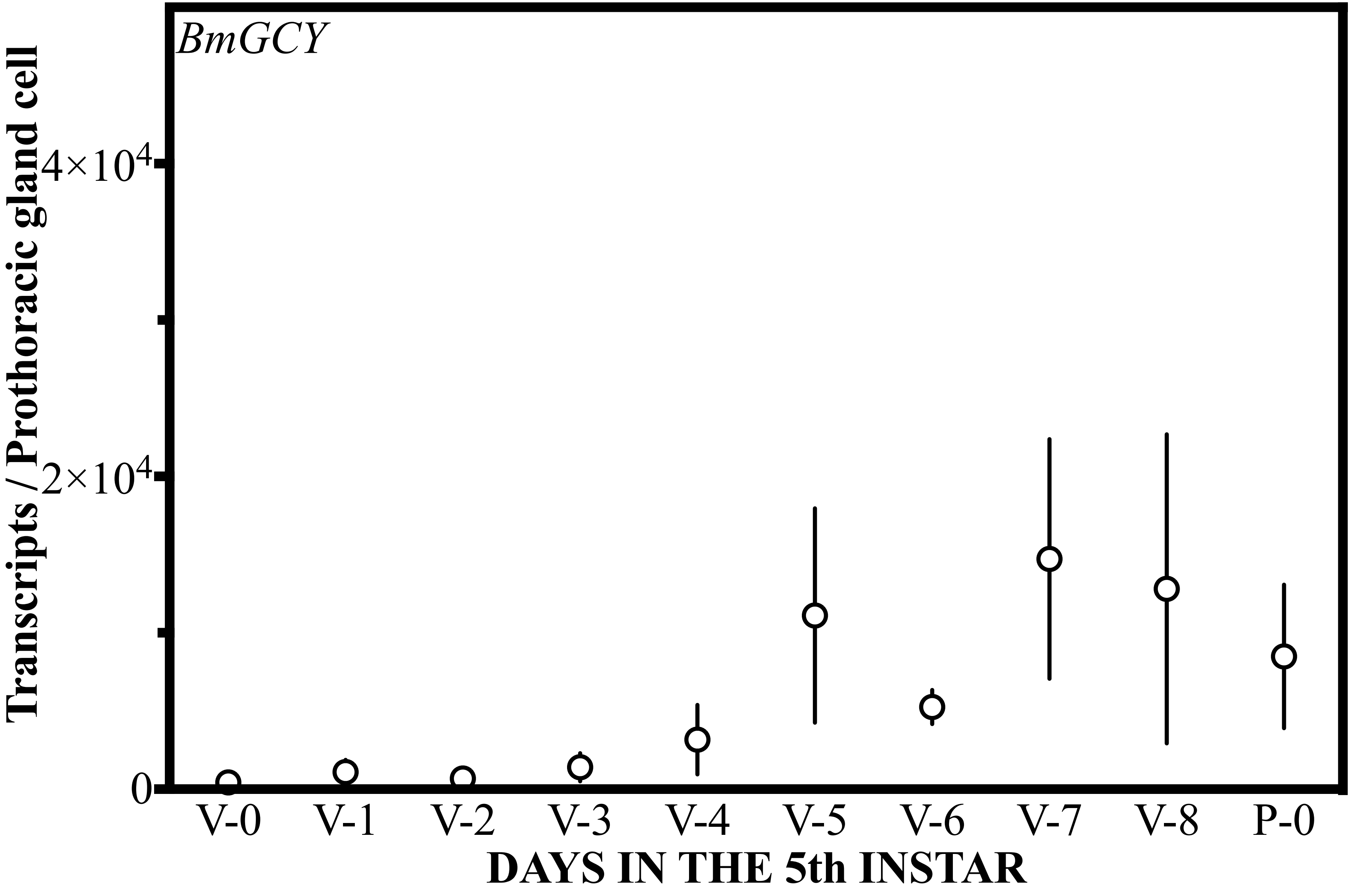
BmGCY
Transcripts / Prothoracic gland cell
4×10^4
2×10^4
0
V-0
V-1
V-2
V-3
V-4
V-5
V-6
V-7
V-8
P-0
DAYS IN THE 5th INSTAR

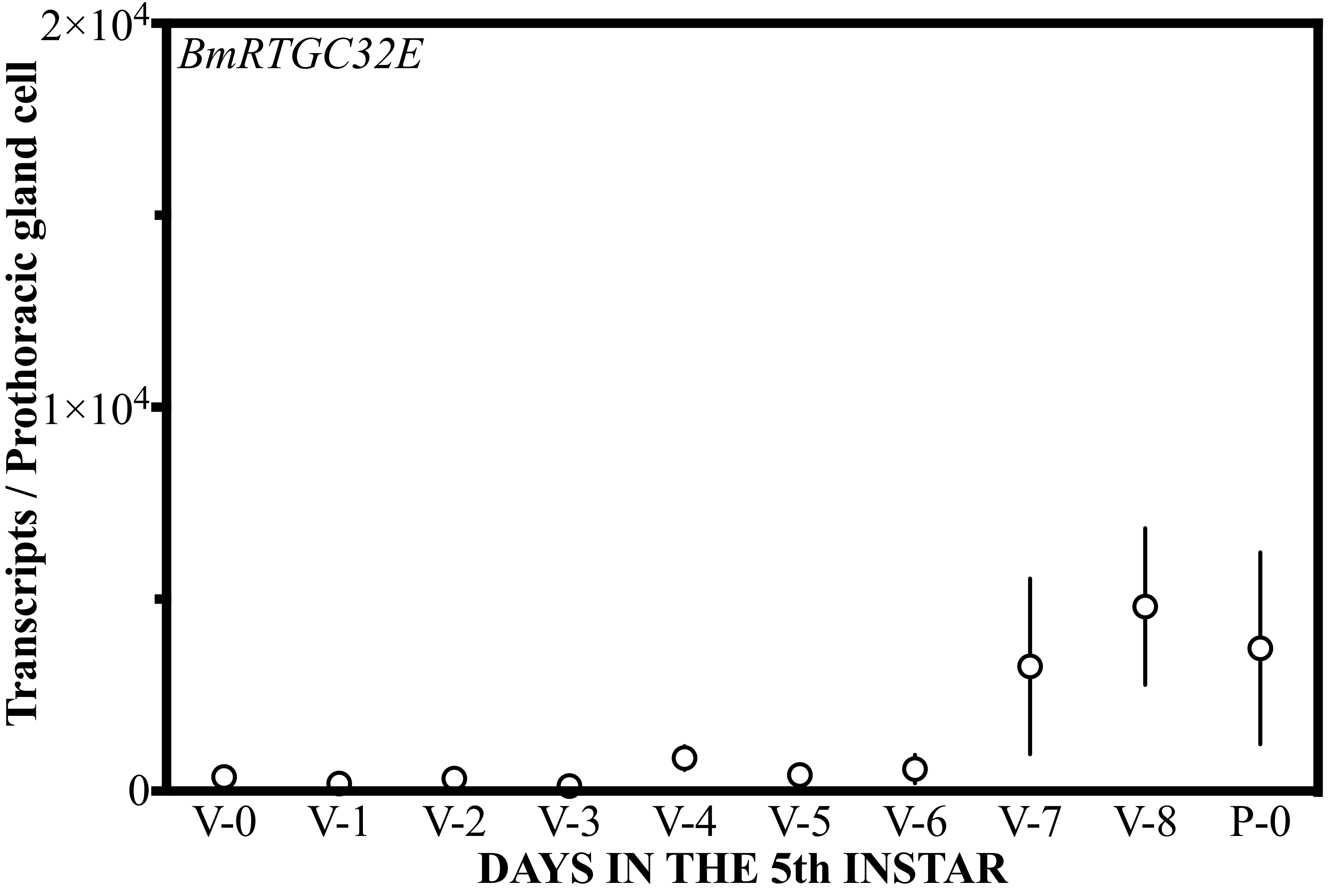
BmRTGC32E
Transcripts / Prothoracic gland cell
2×10^4
1×10^4
0
V-0
V-1
V-2
V-3
V-4
V-5
V-6
V-7
V-8
P-0
DAYS IN THE 5th INSTAR

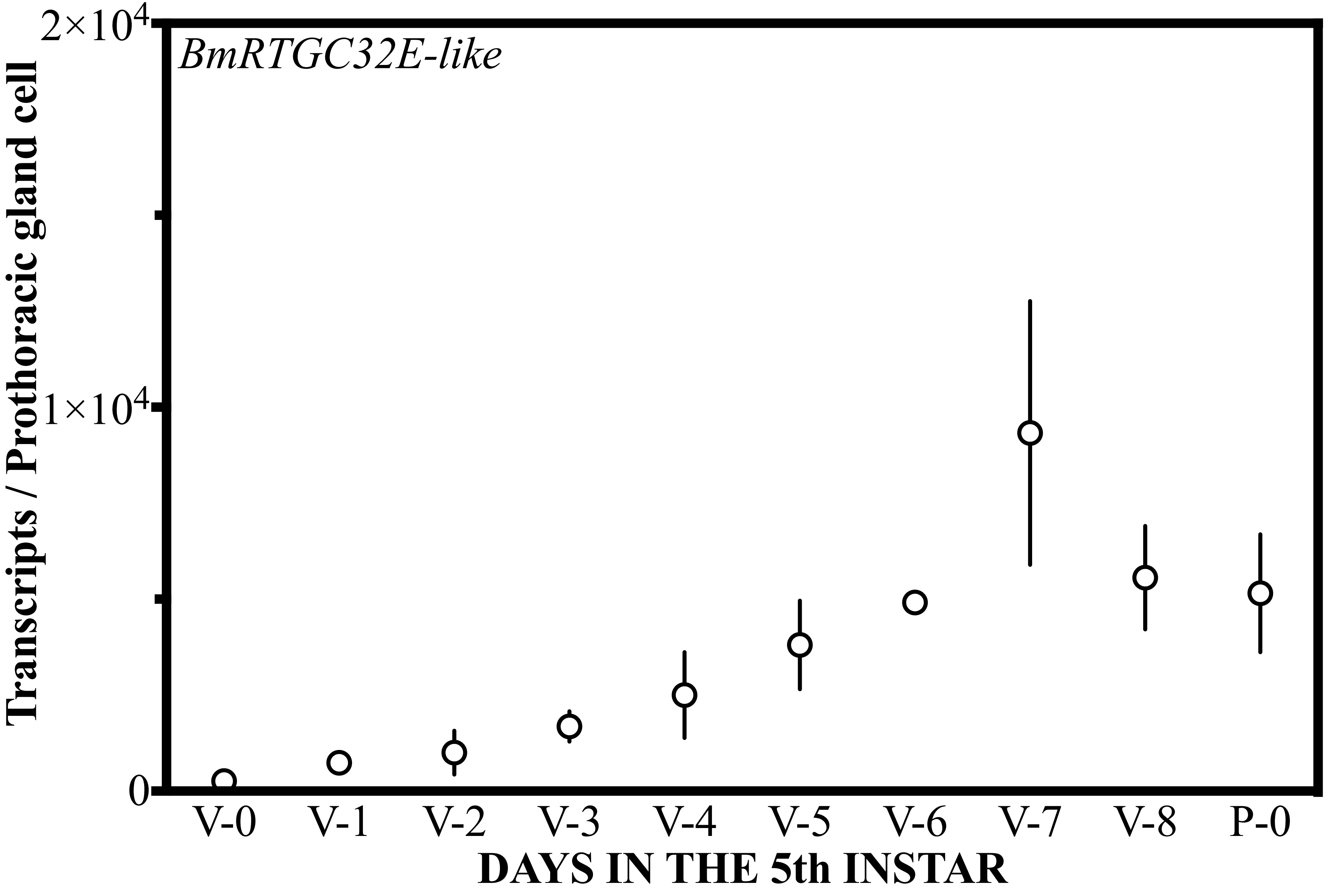

BmRTGC32E-like
2×10^4
1×10^4
0
Transcripts / Prothoracic gland cell
V-0
V-1
V-2
V-3
V-4
V-5
V-6
V-7
V-8
P-0
DAYS IN THE 5th INSTAR

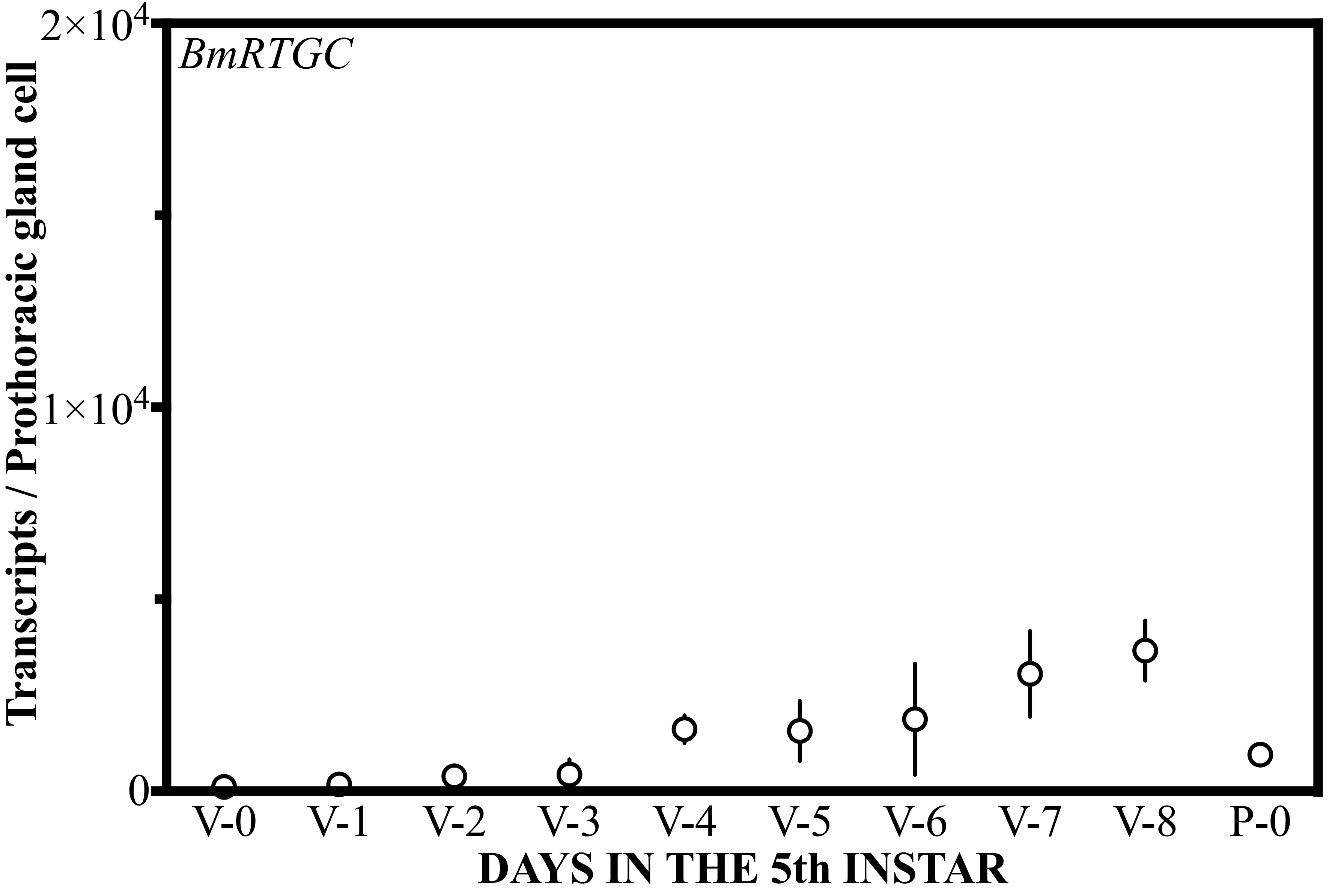

BmRTGC
Transcripts / Prothoracic gland cell
2×10^4
1×10^4
0
V-0
V-1
V-2
V-3
V-4
V-5
V-6
V-7
V-8
P-0
DAYS IN THE 5th INSTAR

Integrins

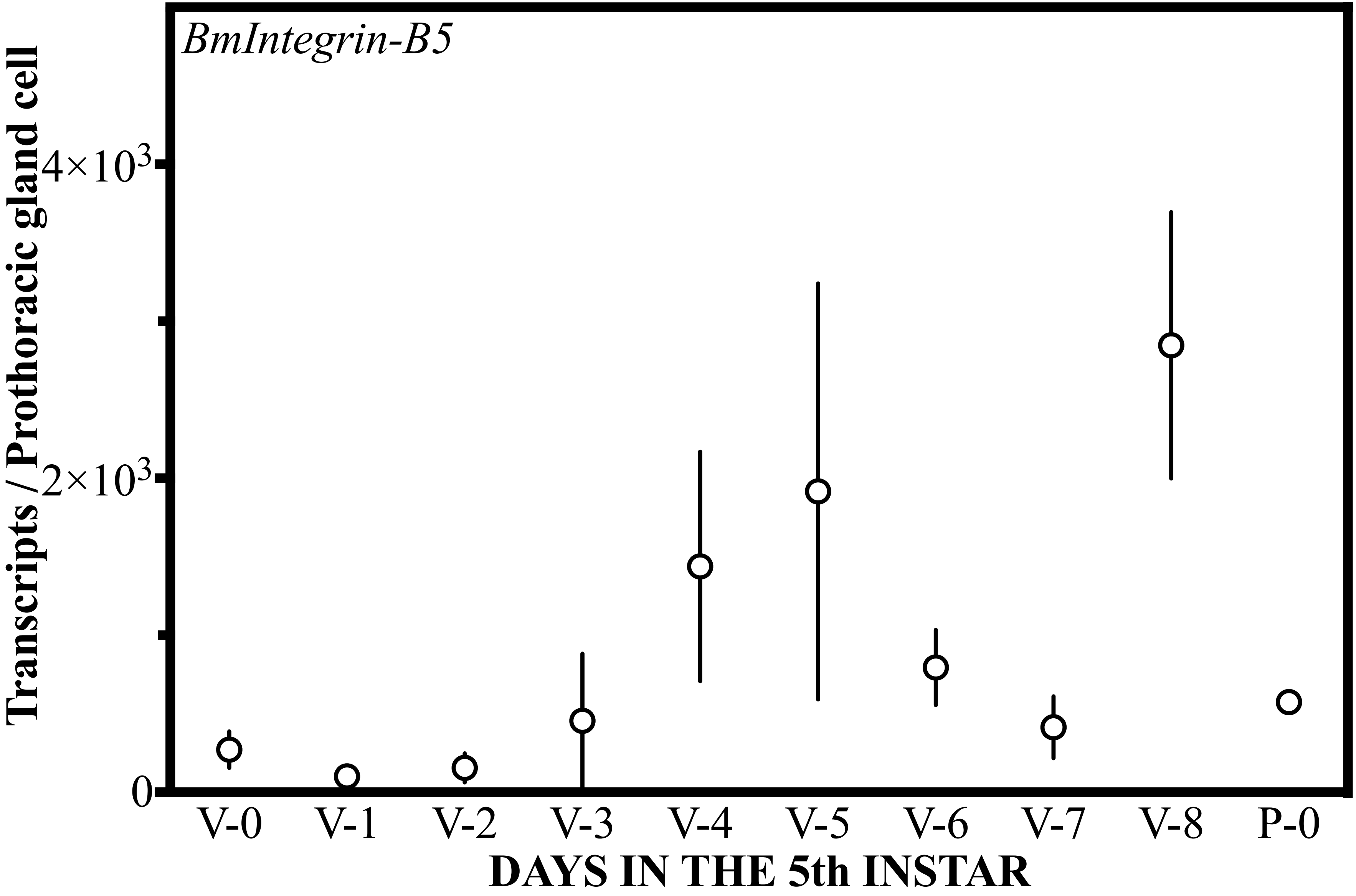

BmIntegrin-B5
Transcripts / Prothoracic gland cell
4×10³
2×10³
0
V-0
V-1
V-2
V-3
V-4
V-5
V-6
V-7
V-8
P-0
DAYS IN THE 5th INSTAR

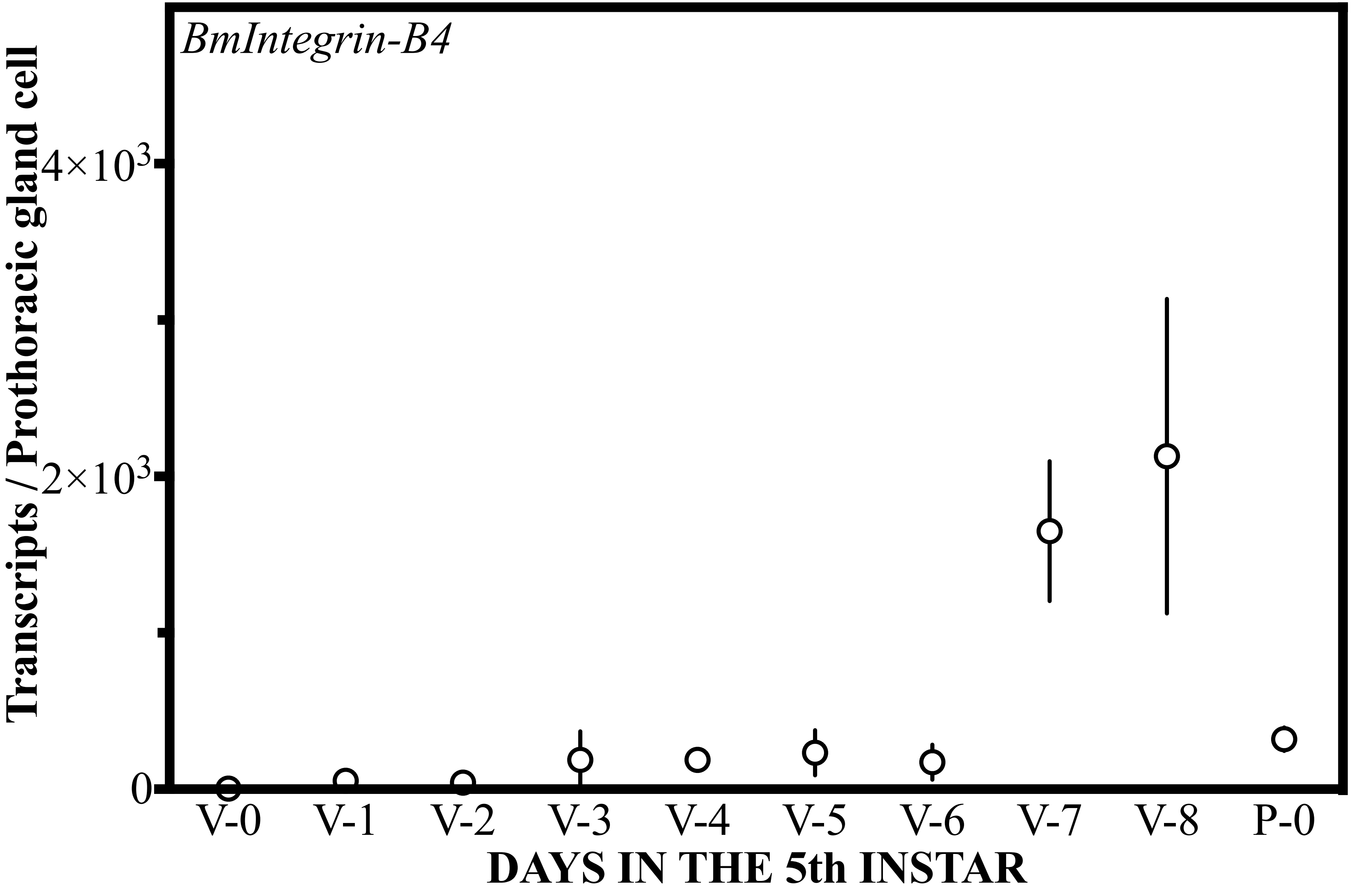
BmIntegrin-B4
Transcripts / Prothoracic gland cell
4×10³
2×10³
0
V-0
V-1
V-2
V-3
V-4
V-5
V-6
V-7
V-8
P-0
DAYS IN THE 5th INSTAR

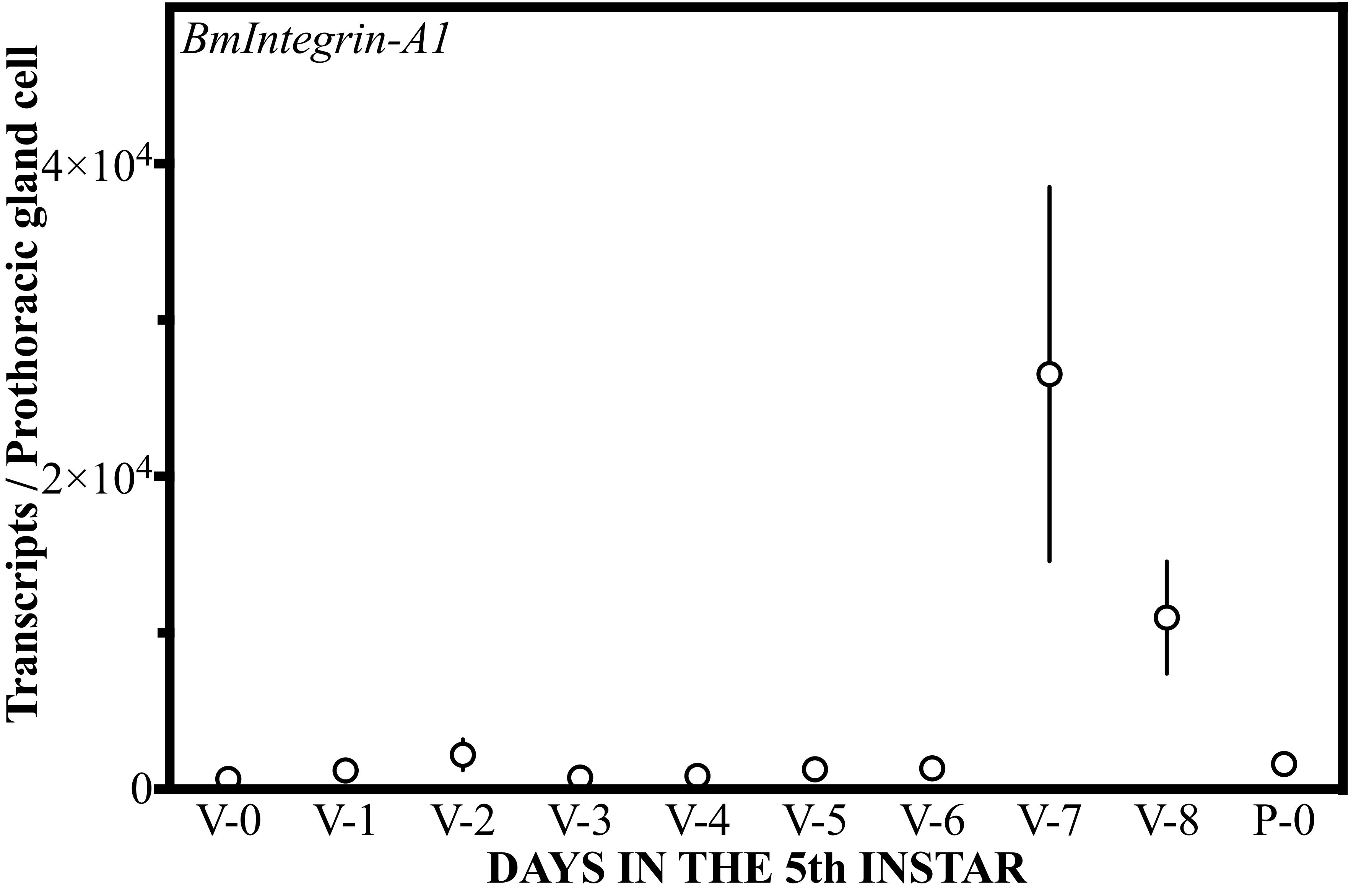
BmIntegrin-A1
Transcripts / Prothoracic gland cell
4×10^4
2×10^4
0
V-0
V-1
V-2
V-3
V-4
V-5
V-6
V-7
V-8
P-0
DAYS IN THE 5th INSTAR

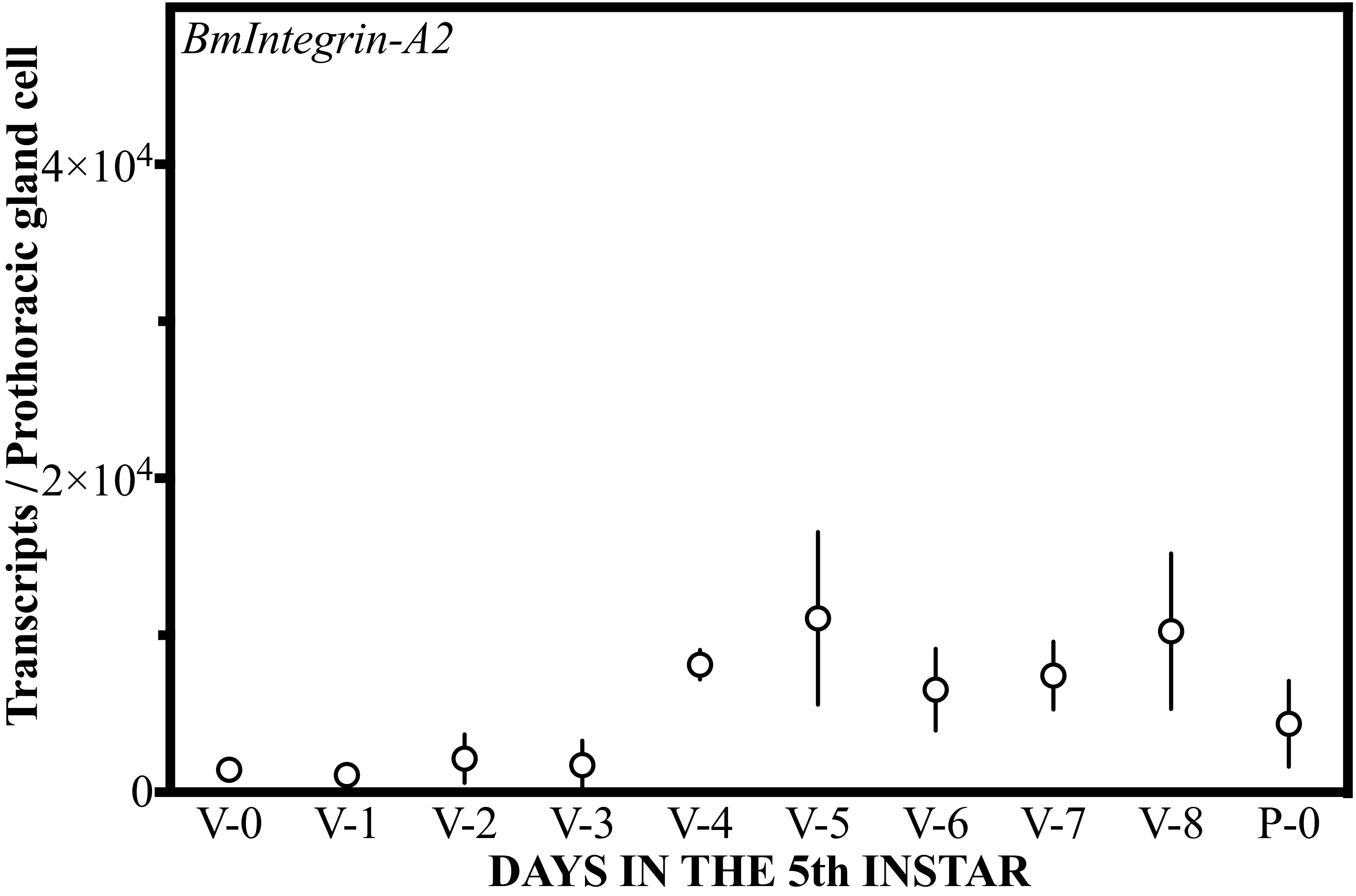

BmIntegrin-A2
Transcripts / Prothoracic gland cell
4×10^4
2×10^4
0
V-0
V-1
V-2
V-3
V-4
V-5
V-6
V-7
V-8
P-0
DAYS IN THE 5th INSTAR

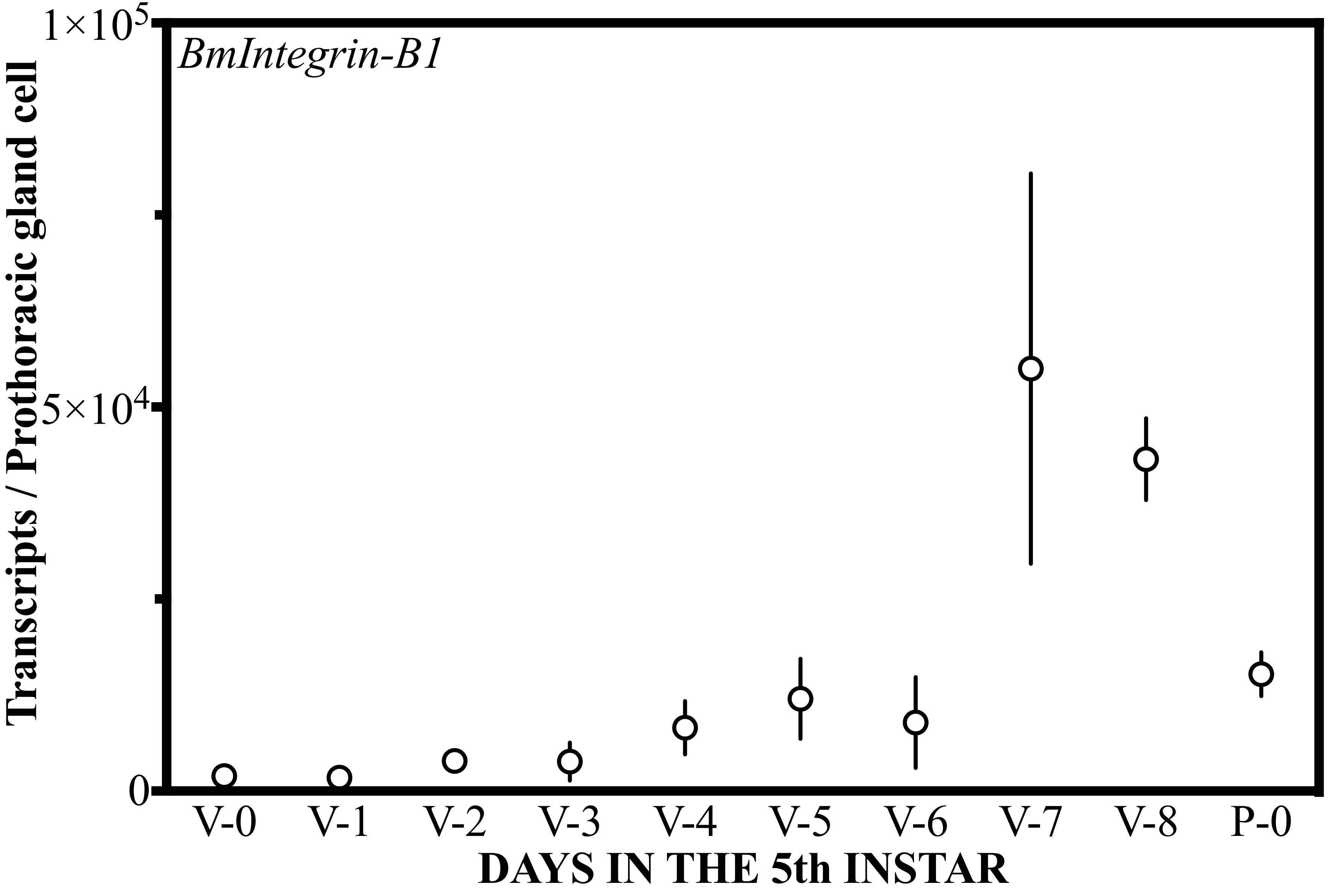

BmIntegrin-B1
1×10^5
5×10^4
0
Transcripts / Prothoracic gland cell
V-0
V-1
V-2
V-3
V-4
V-5
V-6
V-7
V-8
P-0
DAYS IN THE 5th INSTAR

Immunity-related receptors

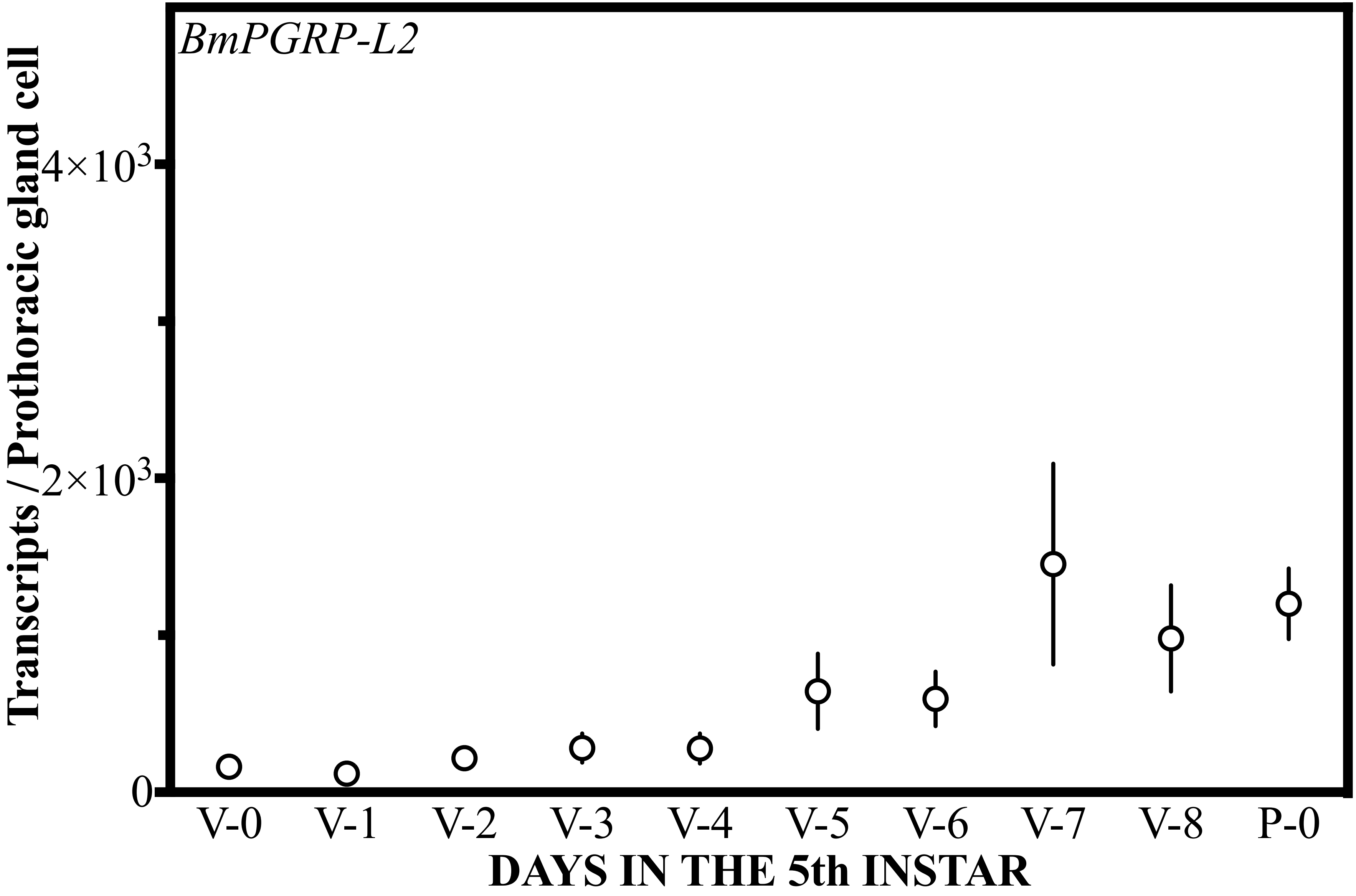

BmPGRP-L2
Transcripts / Prothoracic gland cell
4×10³
2×10³
0
V-0
V-1
V-2
V-3
V-4
V-5
V-6
V-7
V-8
P-0
DAYS IN THE 5th INSTAR

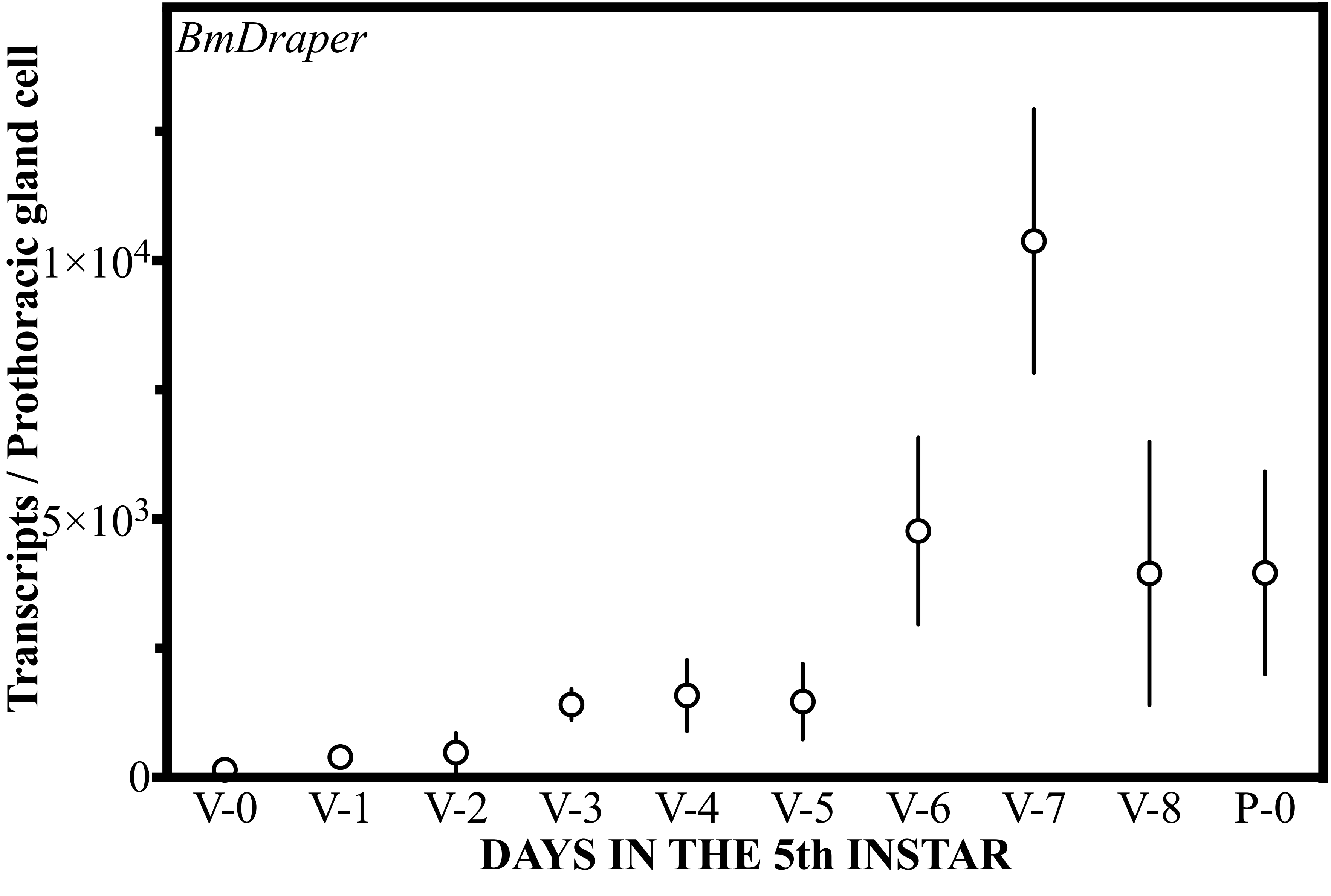
BmDraper
Transcripts / Prothoracic gland cell
1×10⁴
5×10³
0
V-0
V-1
V-2
V-3
V-4
V-5
V-6
V-7
V-8
P-0
DAYS IN THE 5th INSTAR

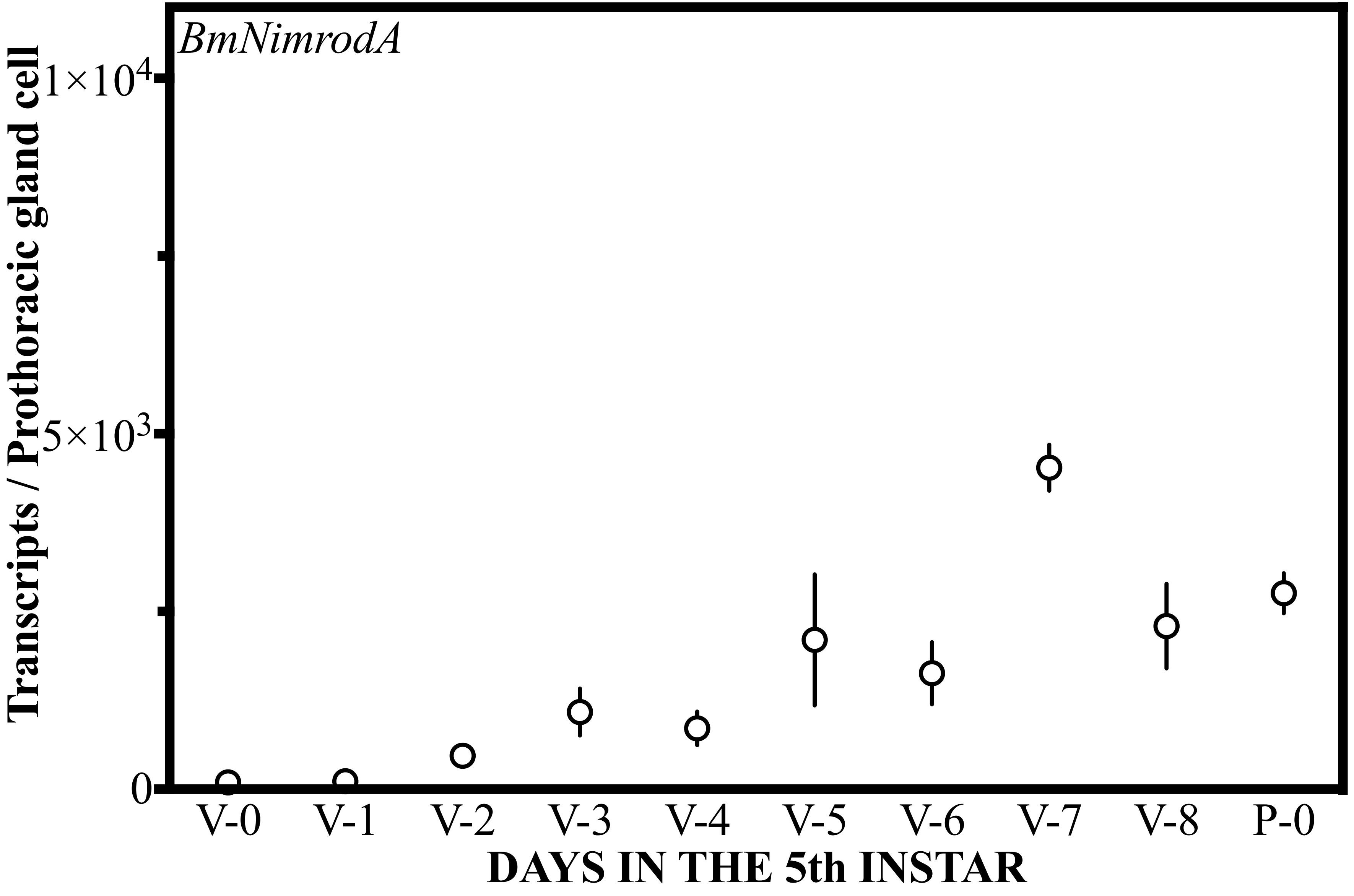
BmNimrodA
Transcripts / Prothoracic gland cell
1×10^4
5×10^3
0
V-0
V-1
V-2
V-3
V-4
V-5
V-6
V-7
V-8
P-0
DAYS IN THE 5th INSTAR

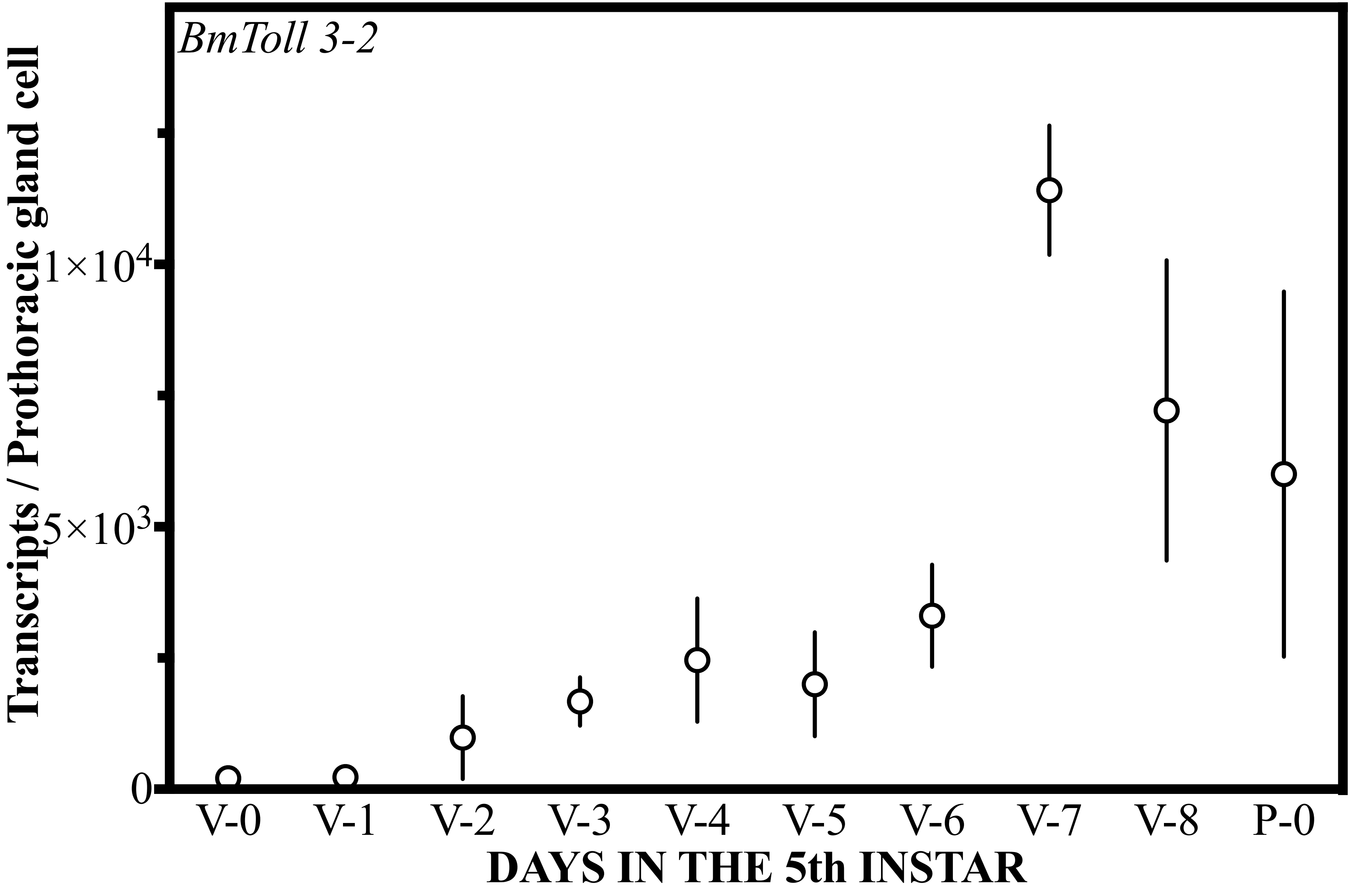
BmToll 3-2
Transcripts / Prothoracic gland cell
1×10^4
5×10^3
0
V-0
V-1
V-2
V-3
V-4
V-5
V-6
V-7
V-8
P-0
DAYS IN THE 5th INSTAR

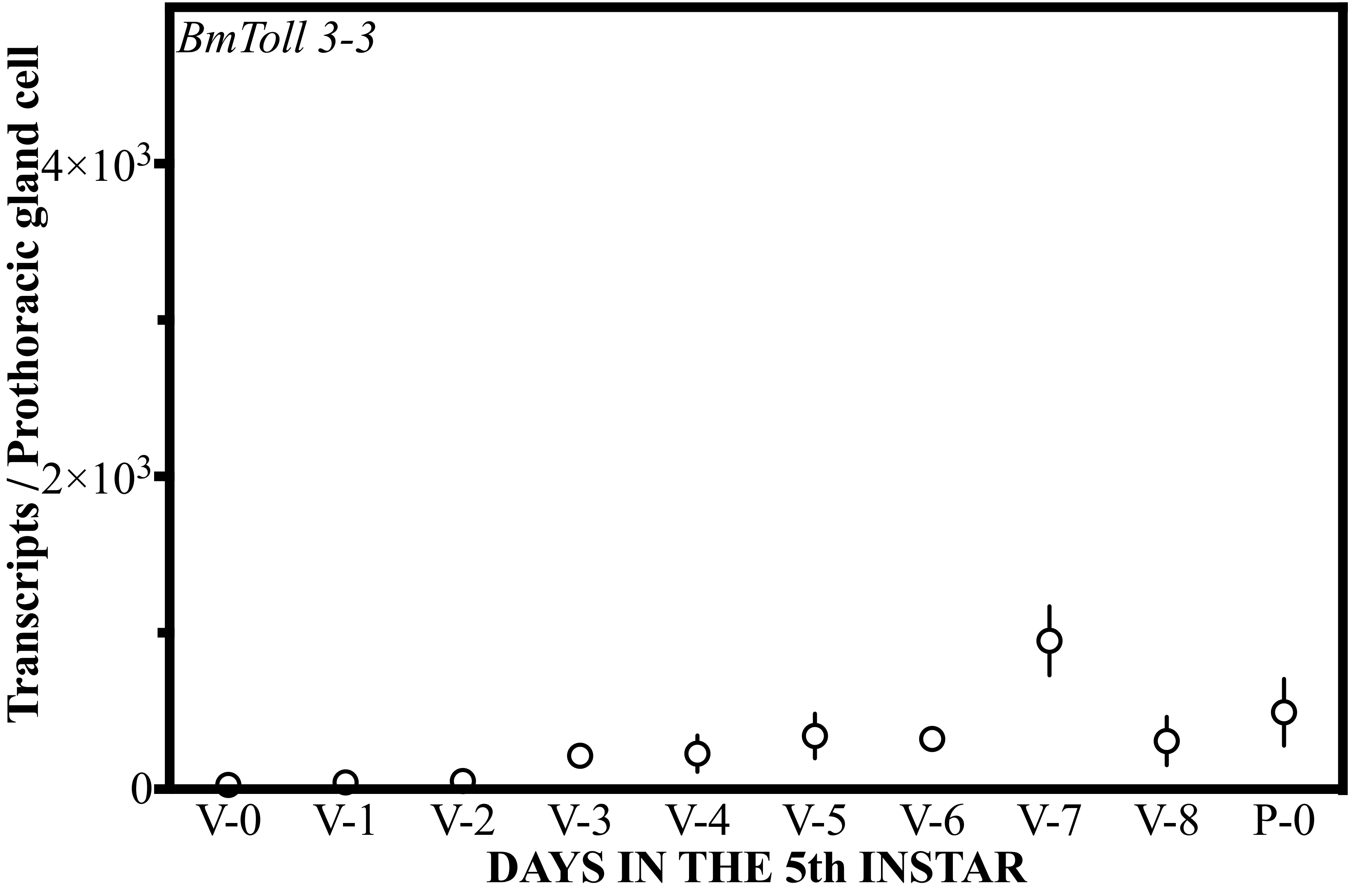
BmToll 3-3
Transcripts / Prothoracic gland cell
4×10³
2×10³
0
V-0
V-1
V-2
V-3
V-4
V-5
V-6
V-7
V-8
P-0
DAYS IN THE 5th INSTAR

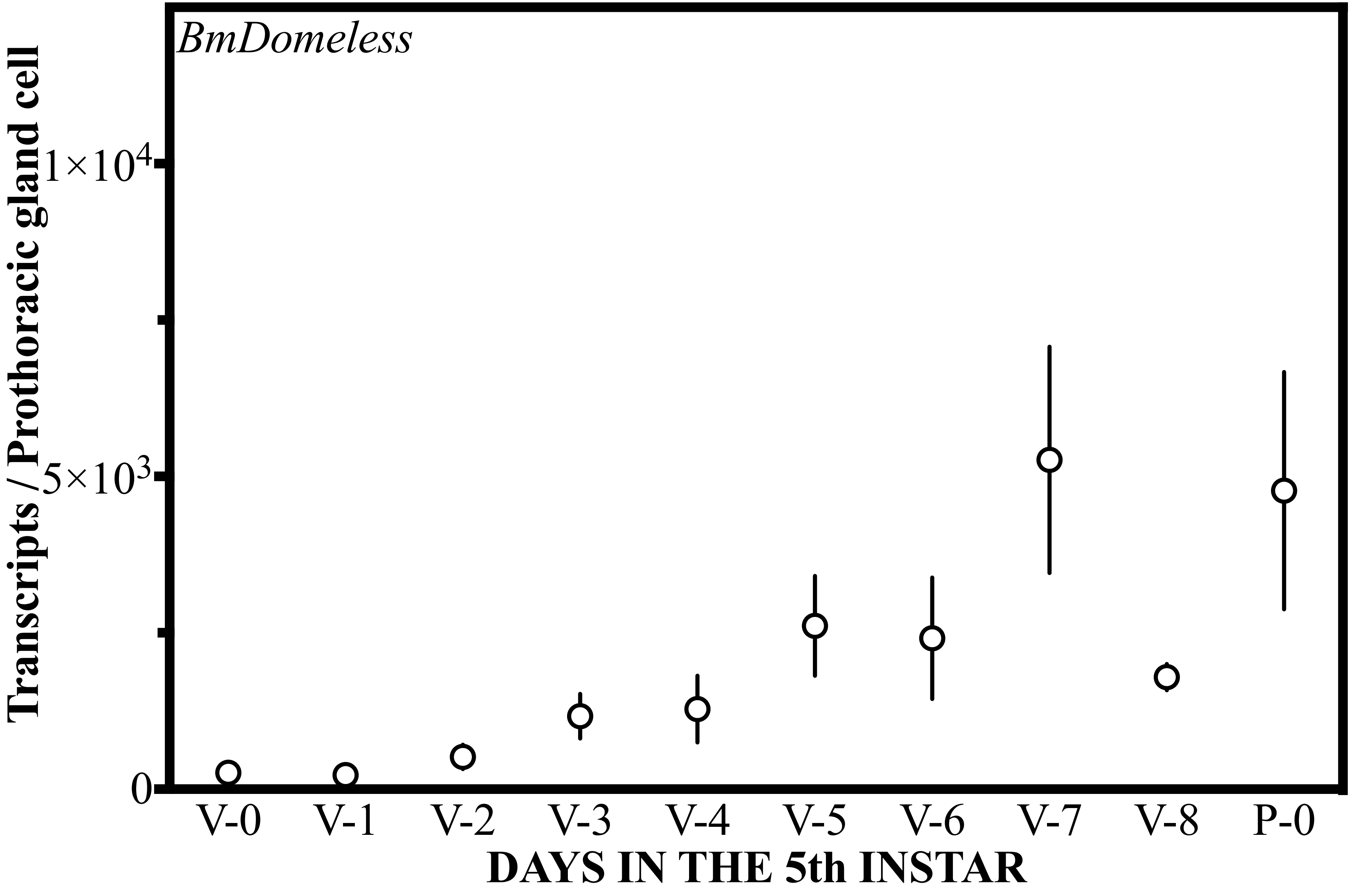
BmDomeless
Transcripts / Prothoracic gland cell
1×10^4
5×10^3
0
V-0
V-1
V-2
V-3
V-4
V-5
V-6
V-7
V-8
P-0
DAYS IN THE 5th INSTAR

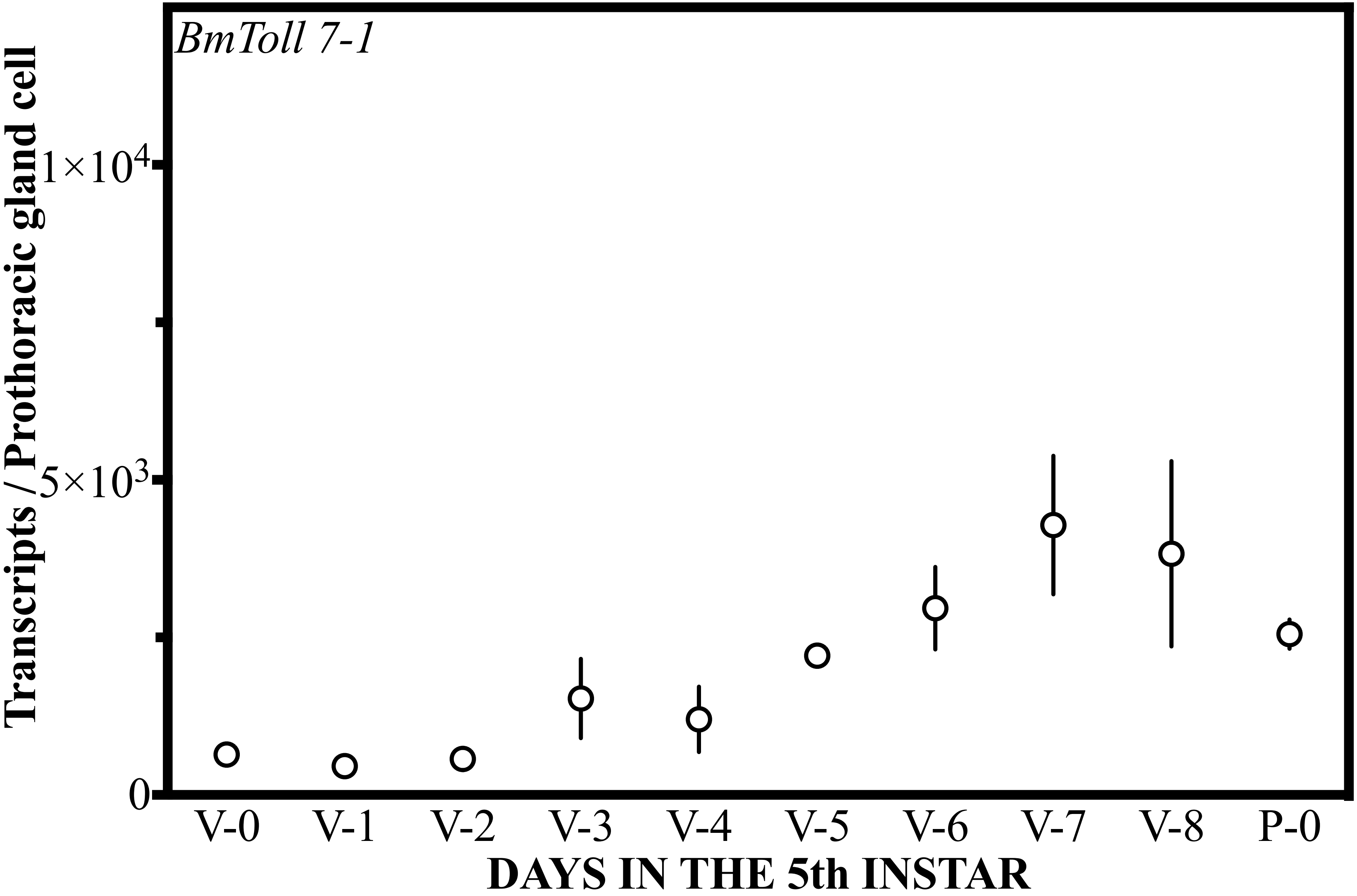

BmToll 7-1
Transcripts / Prothoracic gland cell
1×10^4
5×10^3
0
V-0
V-1
V-2
V-3
V-4
V-5
V-6
V-7
V-8
P-0
DAYS IN THE 5th INSTAR

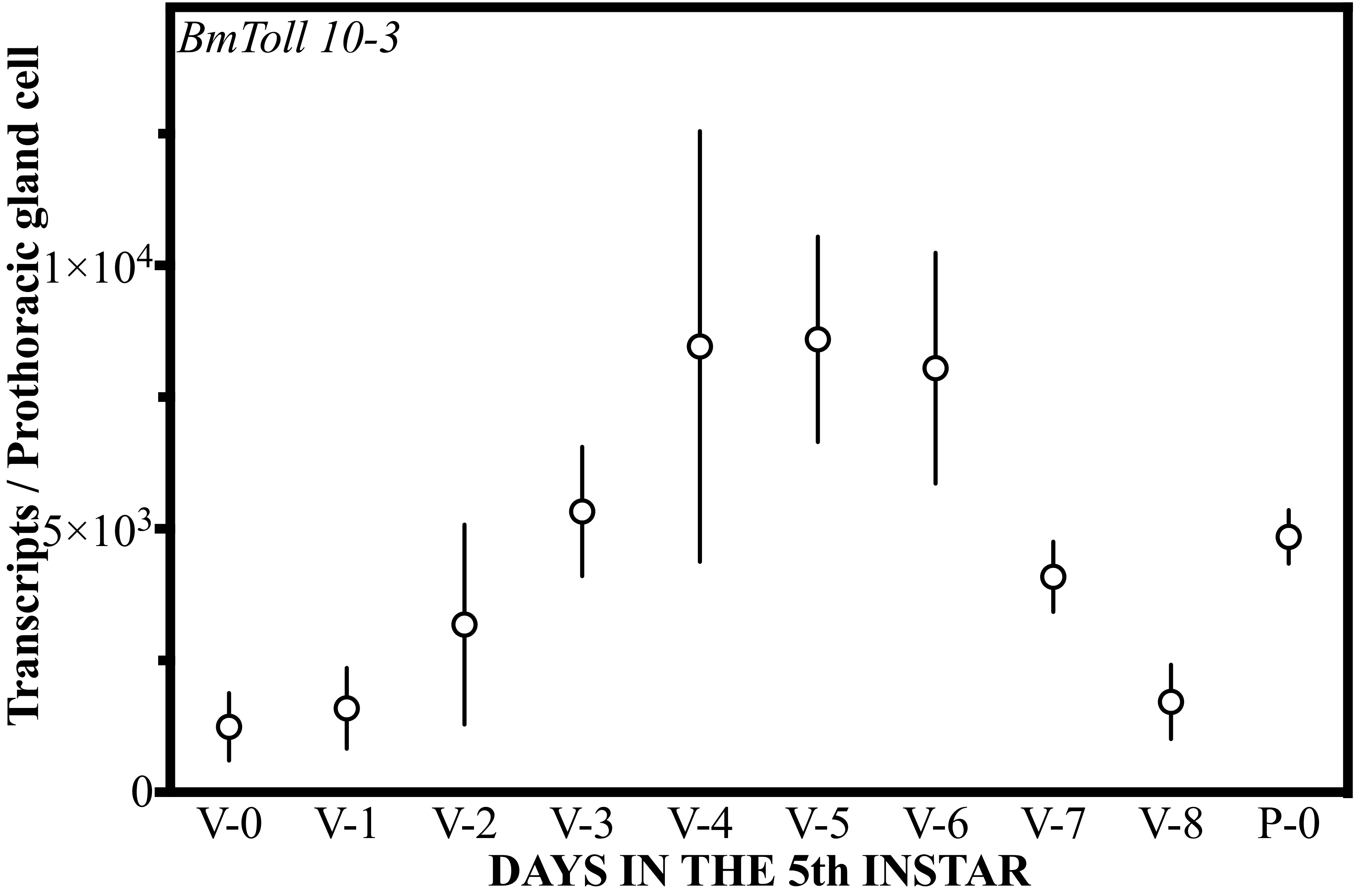
BmToll 10-3
Transcripts / Prothoracic gland cell
1×10^4
5×10^3
0
V-0
V-1
V-2
V-3
V-4
V-5
V-6
V-7
V-8
P-0
DAYS IN THE 5th INSTAR

Other cell membrane receptors

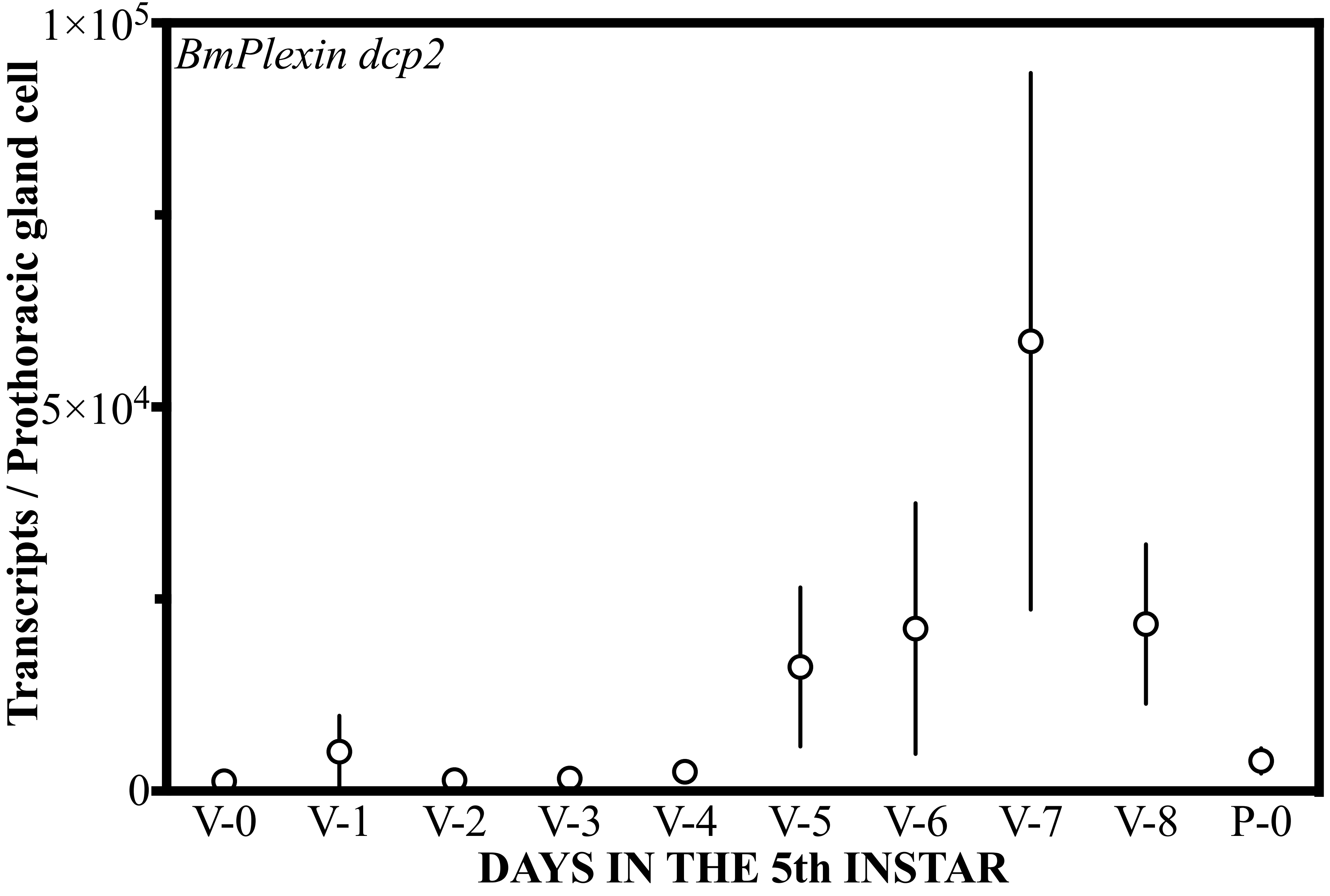

BmPlexin dcp2
1×10^5
5×10^4
0
Transcripts / Prothoracic gland cell
V-0
V-1
V-2
V-3
V-4
V-5
V-6
V-7
V-8
P-0
DAYS IN THE 5th INSTAR

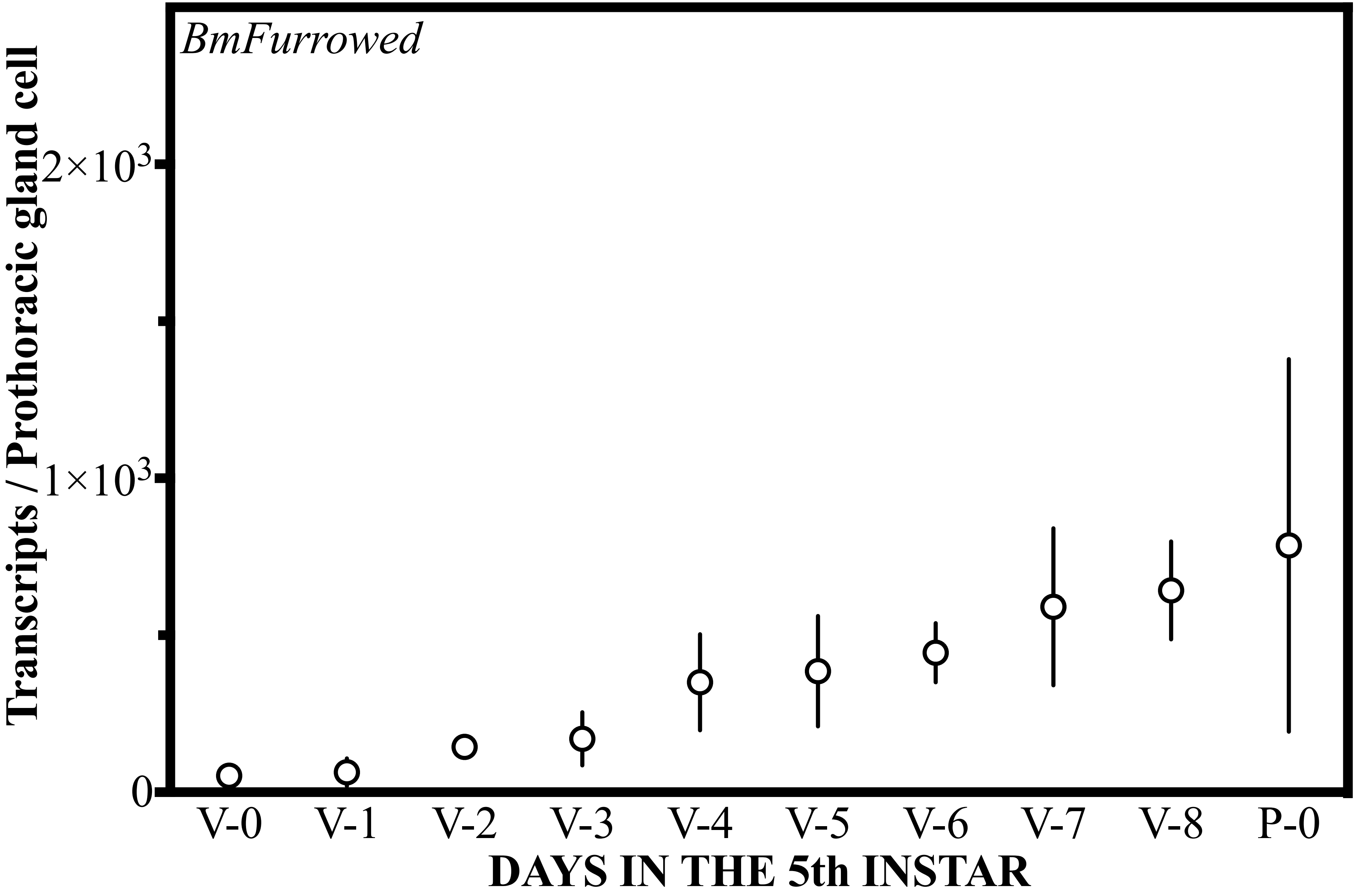

BmFurrowed
Transcripts / Prothoracic gland cell
2×10³
1×10³
0
V-0
V-1
V-2
V-3
V-4
V-5
V-6
V-7
V-8
P-0
DAYS IN THE 5th INSTAR

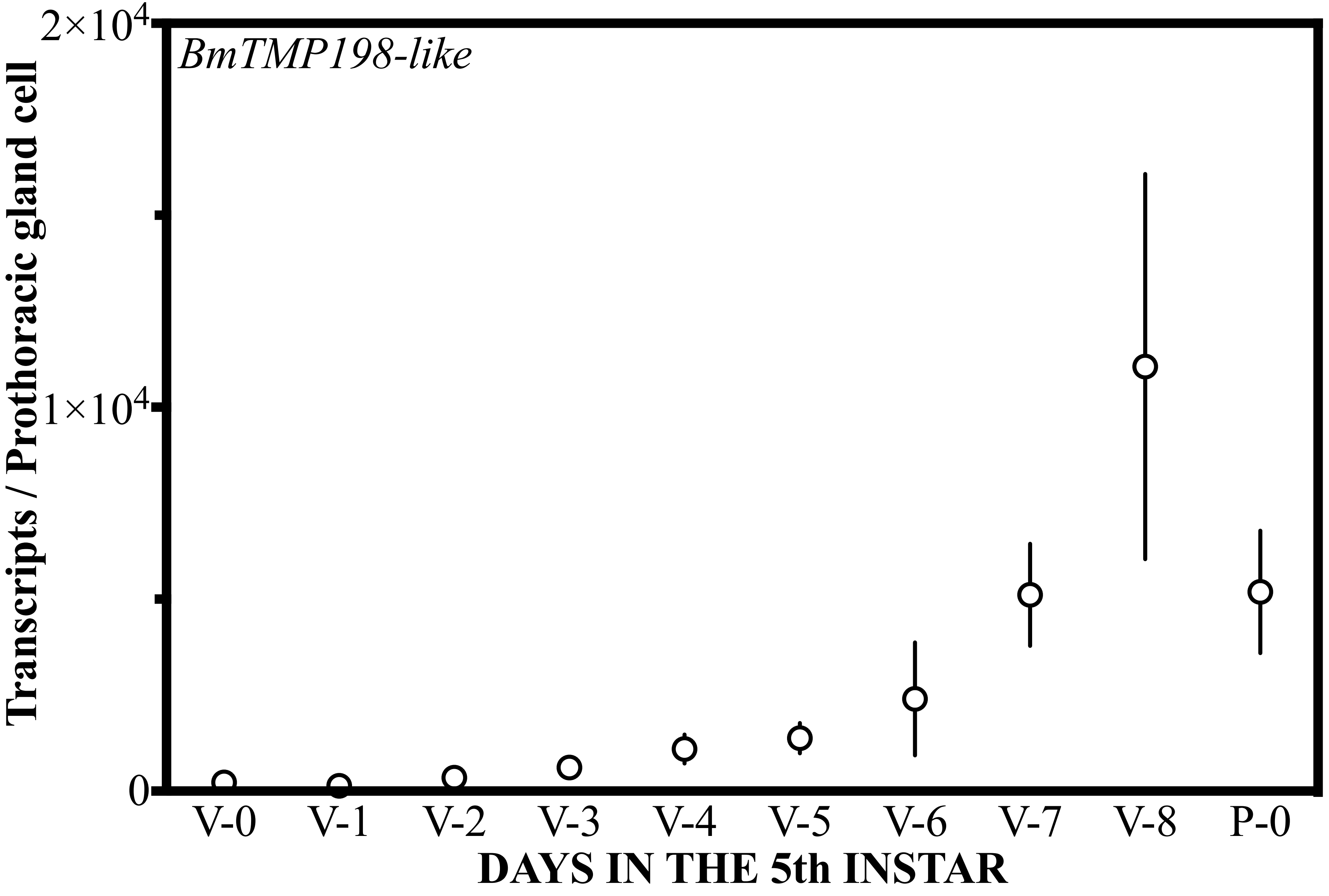

BmTMP198-like
2×10^4
1×10^4
0
Transcripts / Prothoracic gland cell
V-0
V-1
V-2
V-3
V-4
V-5
V-6
V-7
V-8
P-0
DAYS IN THE 5th INSTAR

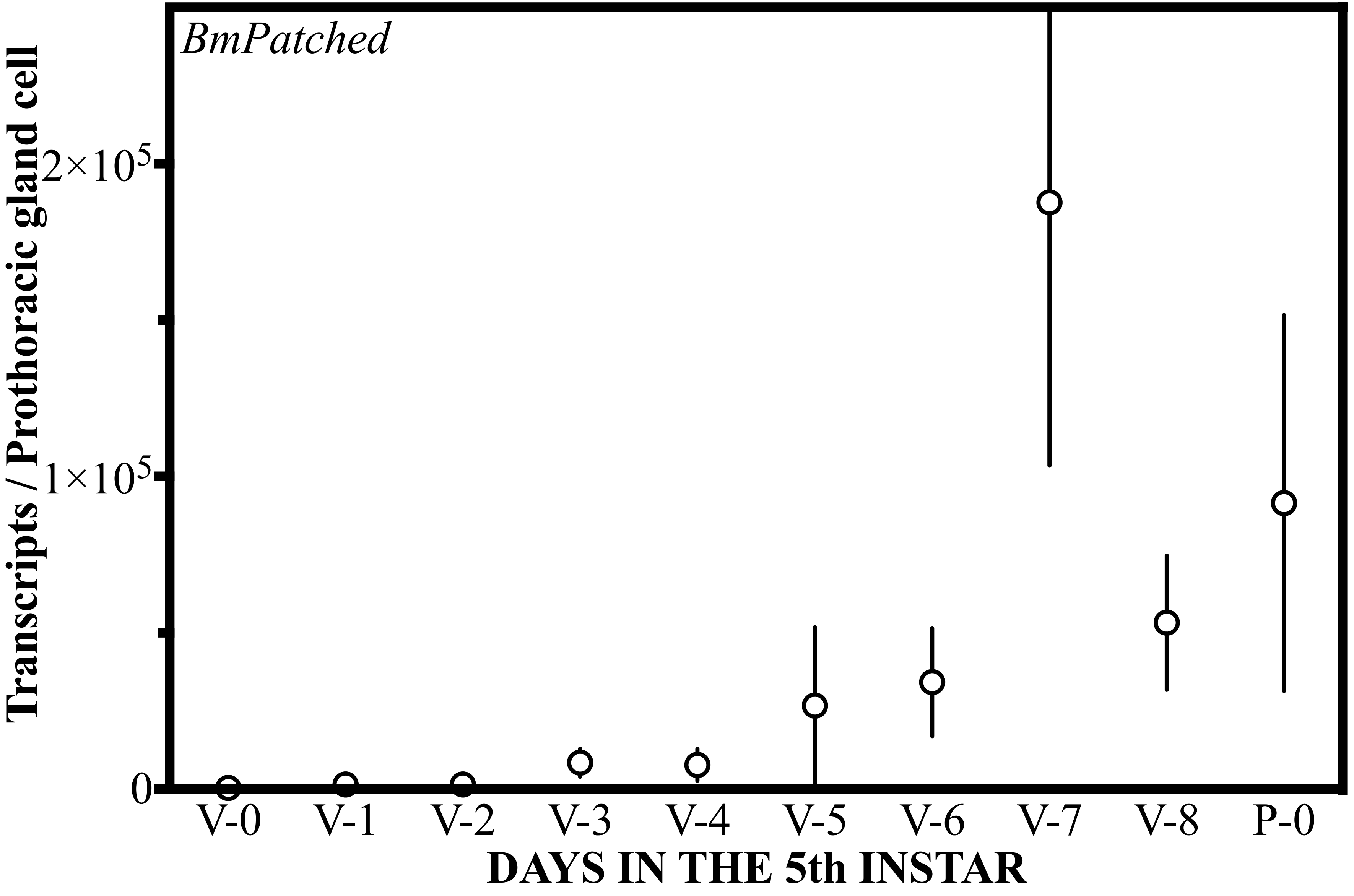

BmPatched
Transcripts / Prothoracic gland cell
2×10^5
1×10^5
0
V-0
V-1
V-2
V-3
V-4
V-5
V-6
V-7
V-8
P-0
DAYS IN THE 5th INSTAR

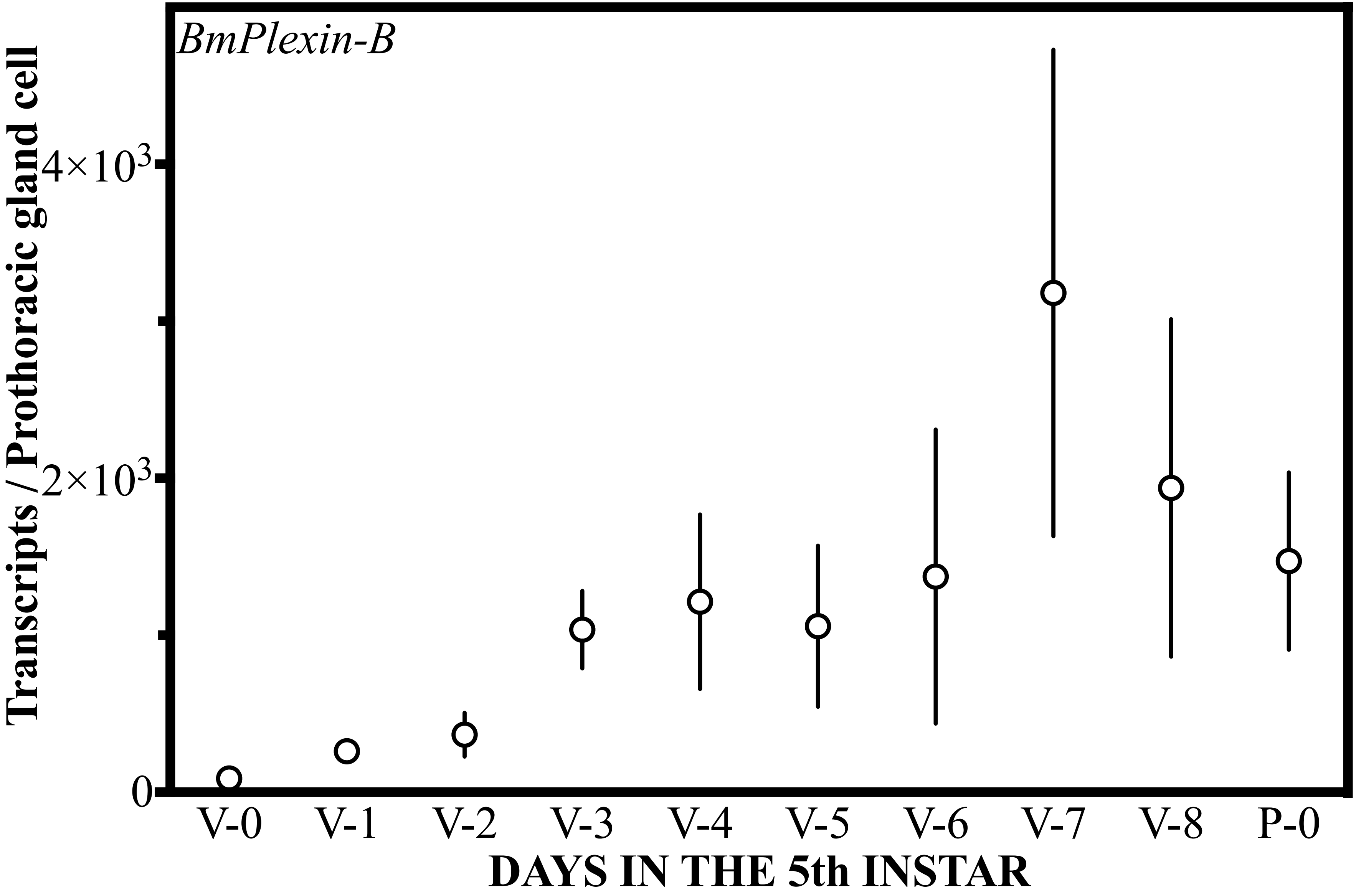

BmPlexin-B
Transcripts / Prothoracic gland cell
4×10³
2×10³
0
V-0
V-1
V-2
V-3
V-4
V-5
V-6
V-7
V-8
P-0
DAYS IN THE 5th INSTAR

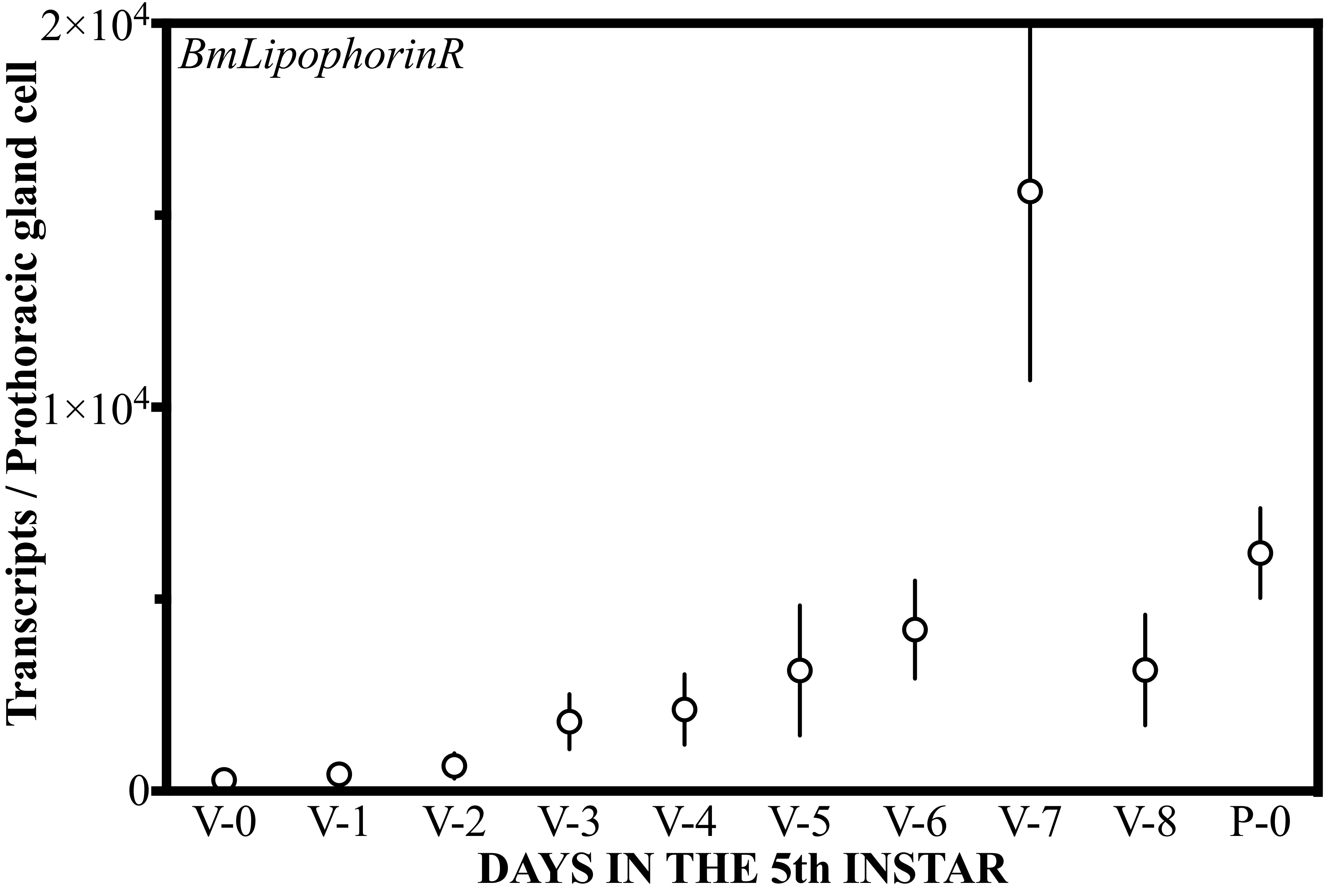
BmLipophorinR
Transcripts / Prothoracic gland cell
2×10^4
1×10^4
0
V-0
V-1
V-2
V-3
V-4
V-5
V-6
V-7
V-8
P-0
DAYS IN THE 5th INSTAR

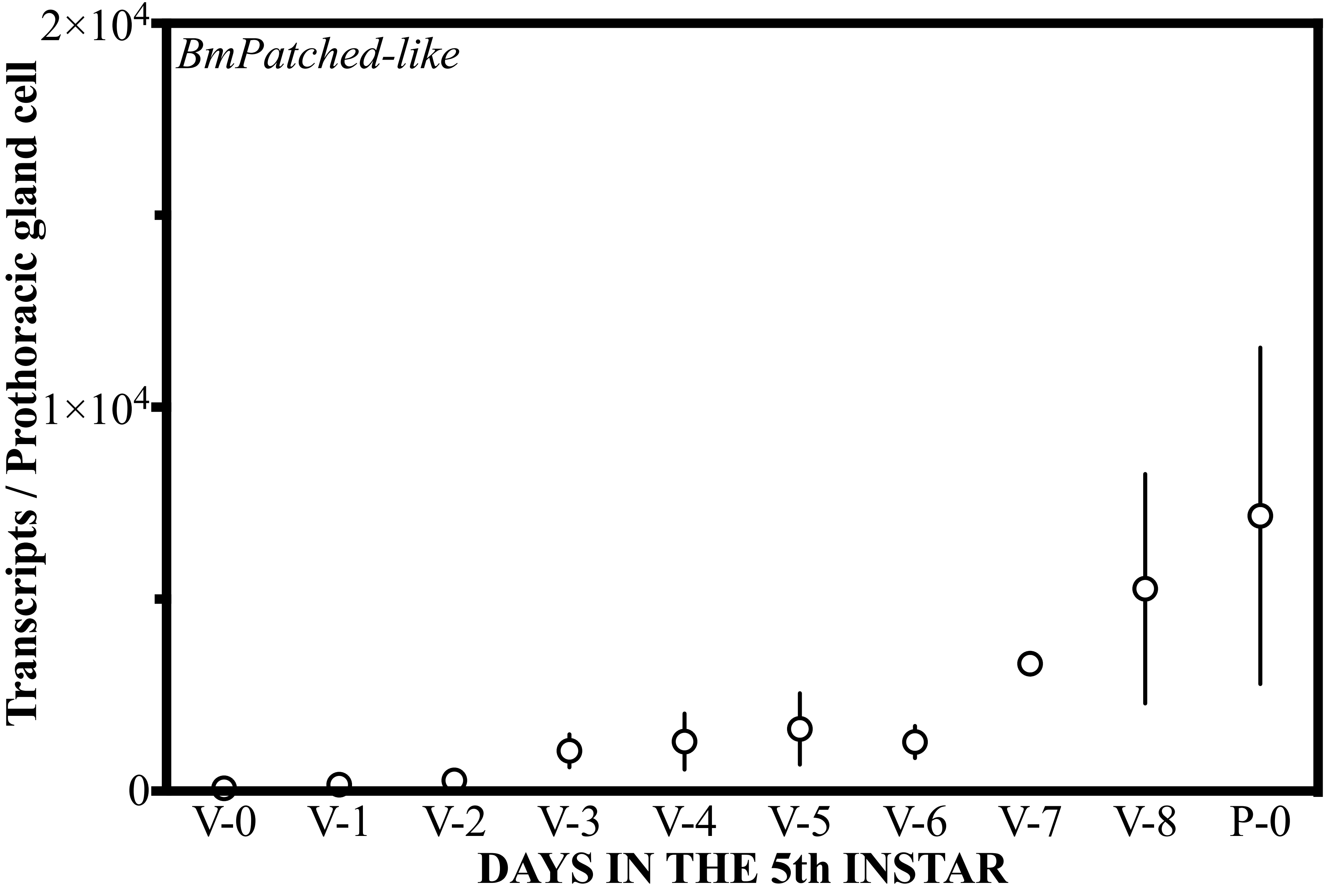

BmPatched-like
2×10^4
1×10^4
0
Transcripts / Prothoracic gland cell
V-0
V-1
V-2
V-3
V-4
V-5
V-6
V-7
V-8
P-0
DAYS IN THE 5th INSTAR

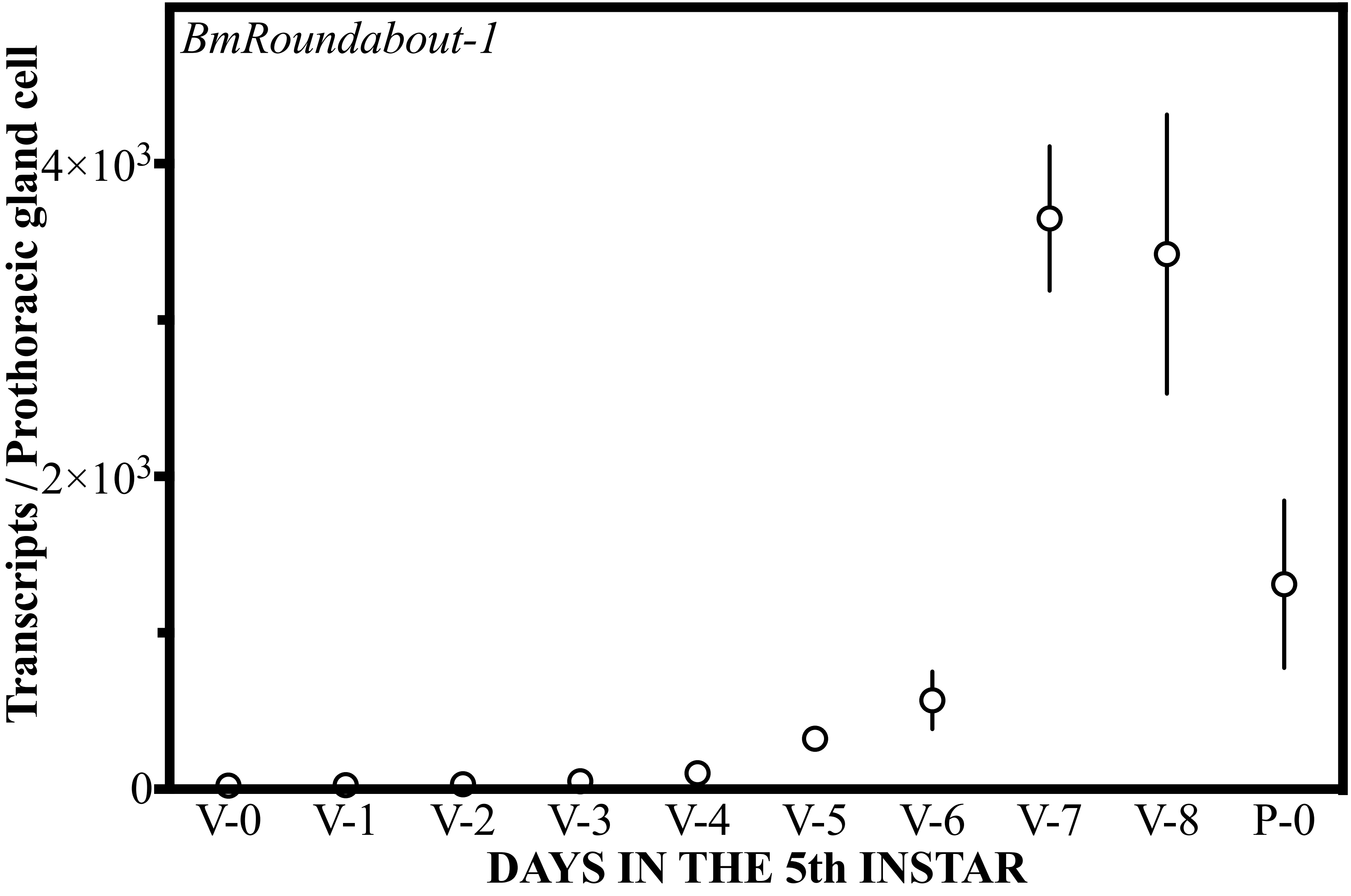

BmRoundabout-1
Transcripts / Prothoracic gland cell
4×10³
2×10³
0
V-0
V-1
V-2
V-3
V-4
V-5
V-6
V-7
V-8
P-0
DAYS IN THE 5th INSTAR

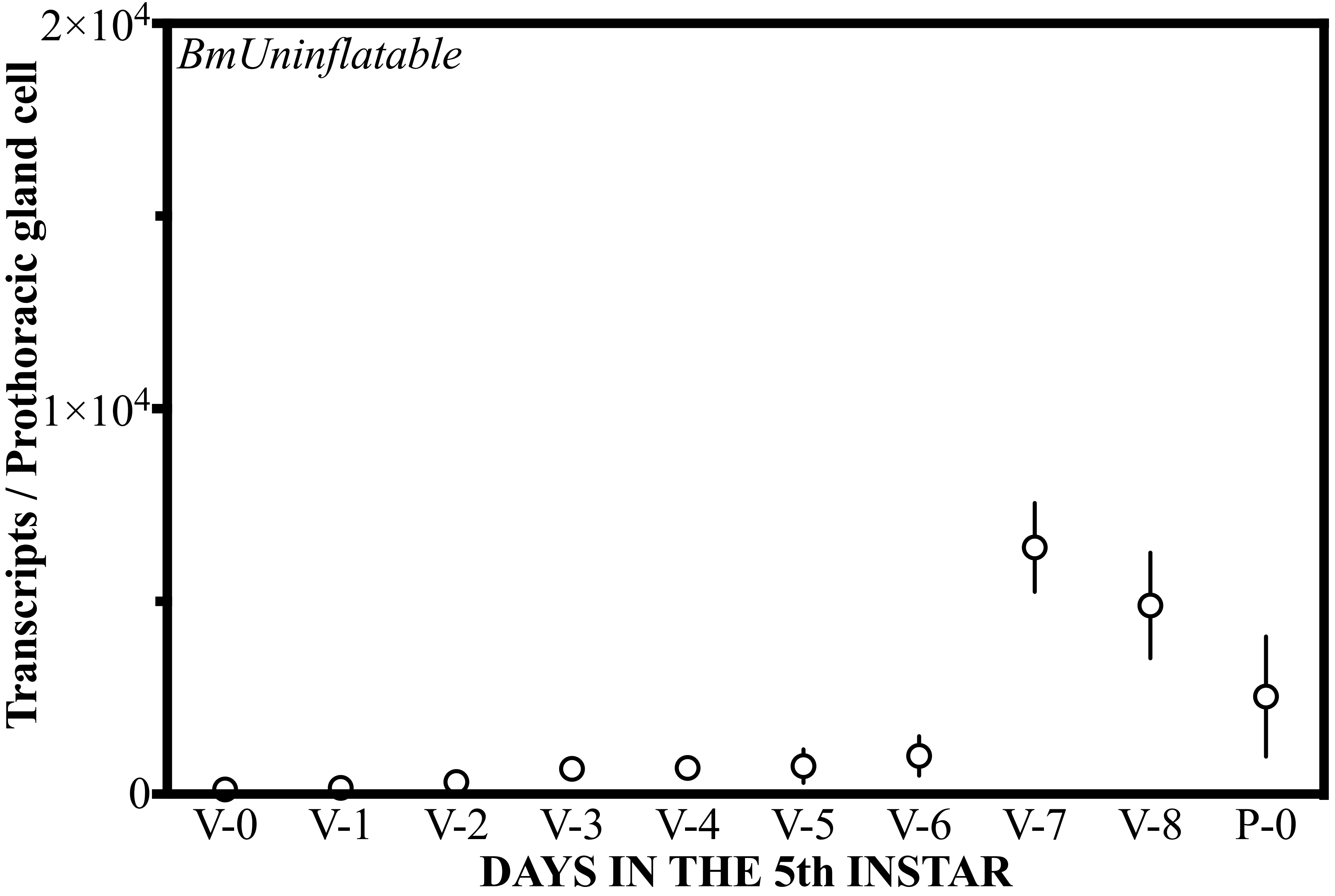

BmUninflatable
Transcripts / Prothoracic gland cell
2×10^4
1×10^4
0
V-0
V-1
V-2
V-3
V-4
V-5
V-6
V-7
V-8
P-0
DAYS IN THE 5th INSTAR

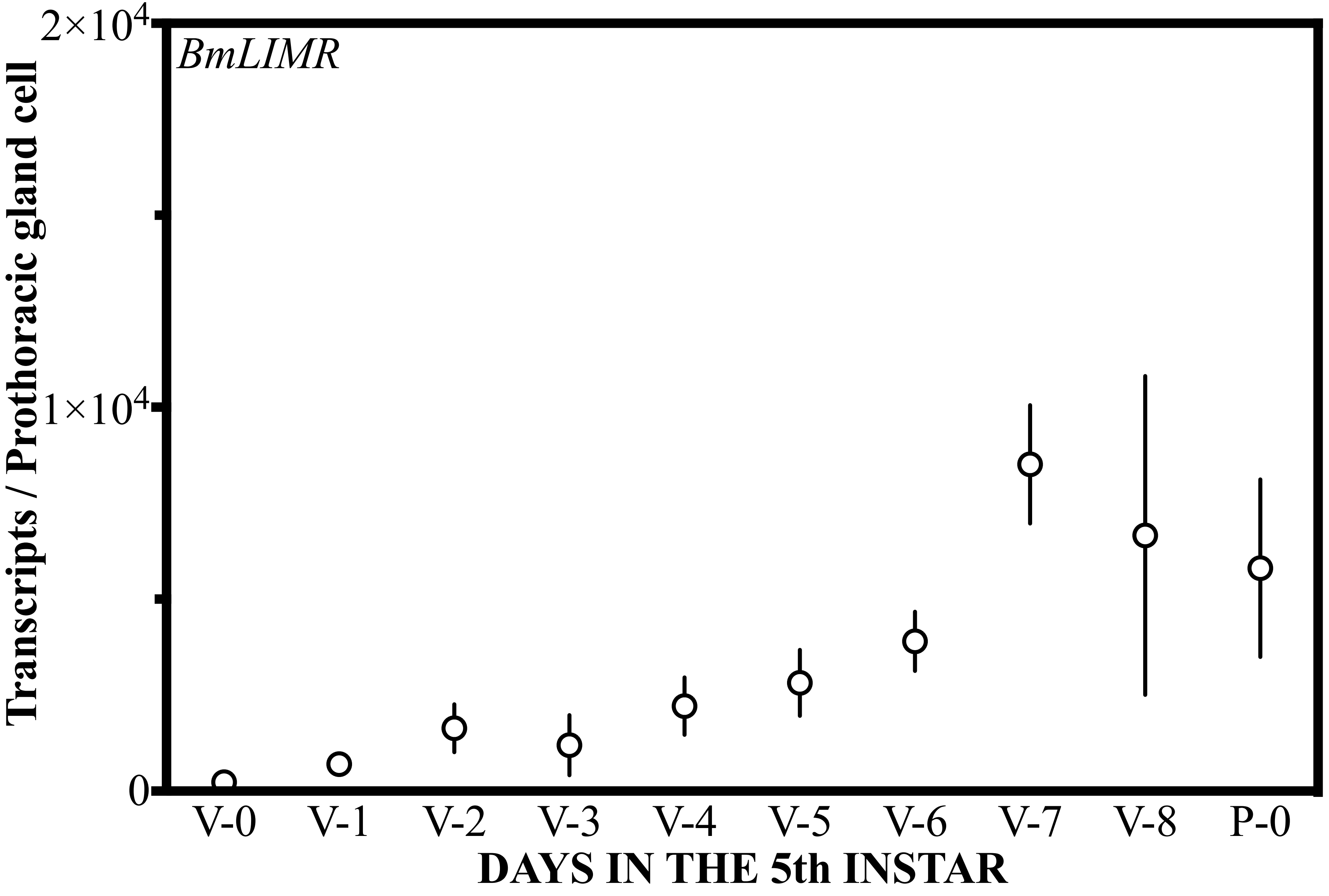

BmLIMR
2×10^4
1×10^4
0
Transcripts / Prothoracic gland cell
V-0
V-1
V-2
V-3
V-4
V-5
V-6
V-7
V-8
P-0
DAYS IN THE 5th INSTAR

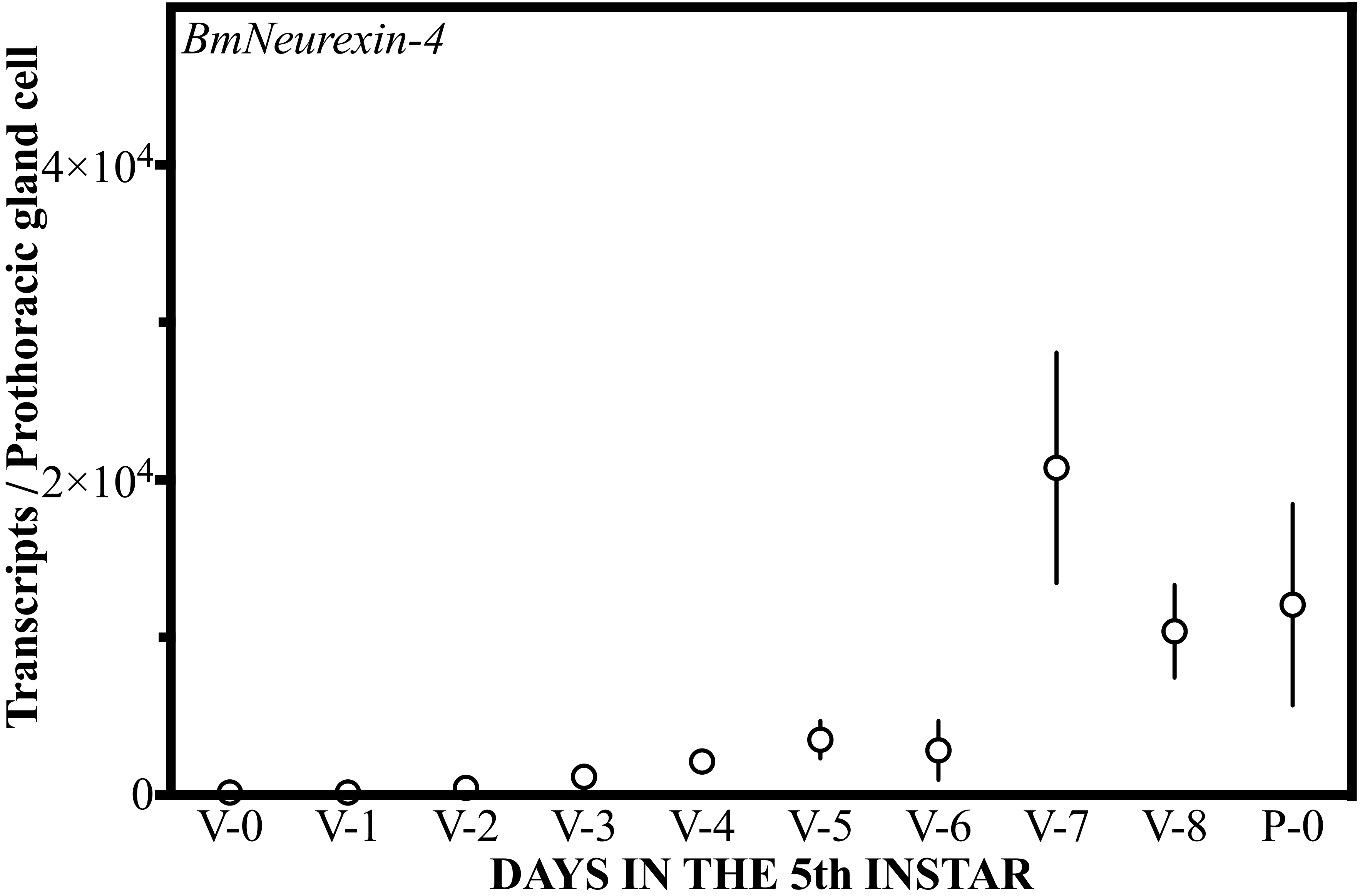
BmNeurexin-4
Transcripts / Prothoracic gland cell
4×10^4
2×10^4
0
V-0
V-1
V-2
V-3
V-4
V-5
V-6
V-7
V-8
P-0
DAYS IN THE 5th INSTAR

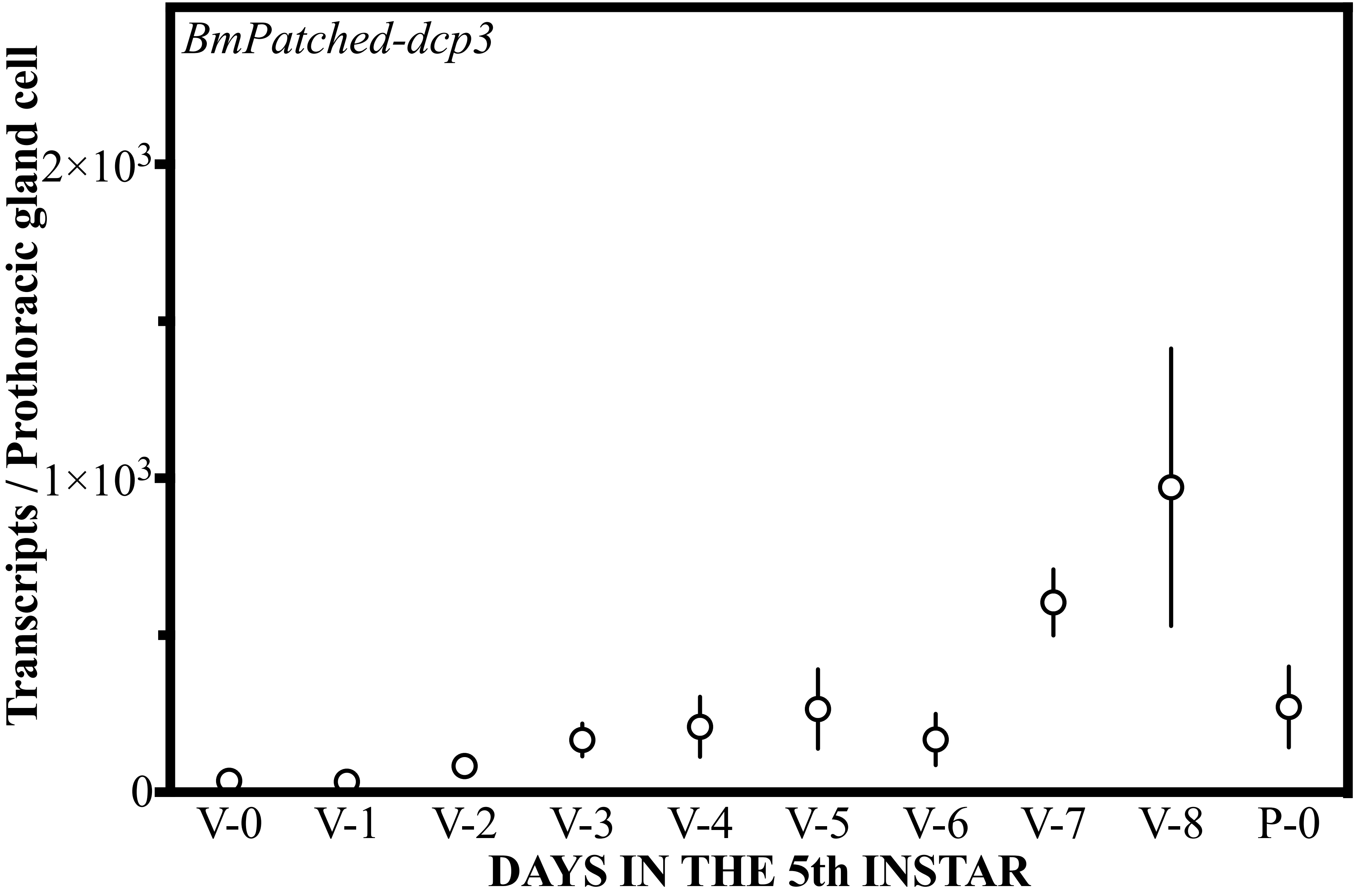
BmPatched-dcp3
Transcripts / Prothoracic gland cell
2×10³
1×10³
0
V-0
V-1
V-2
V-3
V-4
V-5
V-6
V-7
V-8
P-0
DAYS IN THE 5th INSTAR

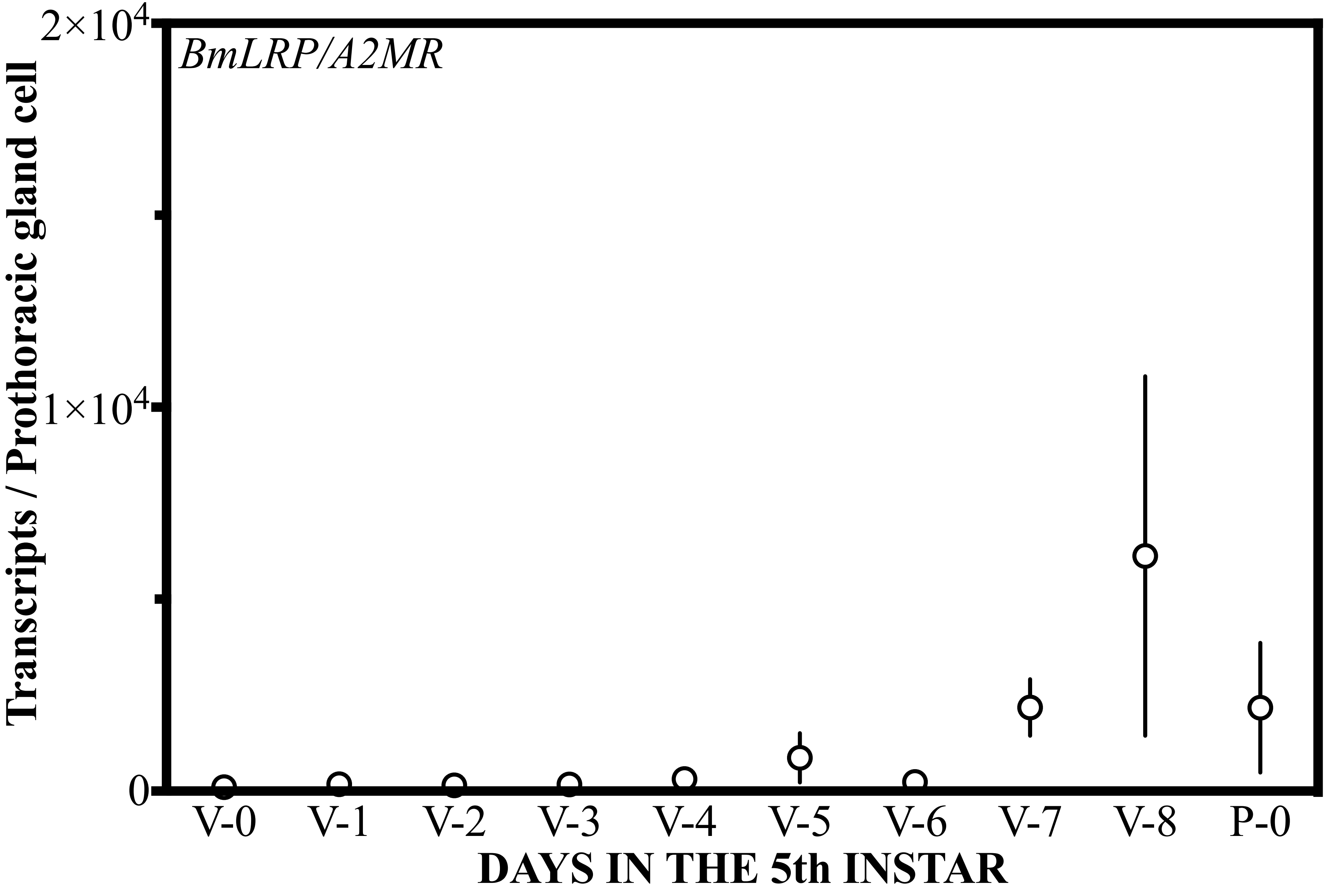
BmLRP/A2MR
2×10^4
1×10^4
0
Transcripts / Prothoracic gland cell
V-0
V-1
V-2
V-3
V-4
V-5
V-6
V-7
V-8
P-0
DAYS IN THE 5th INSTAR

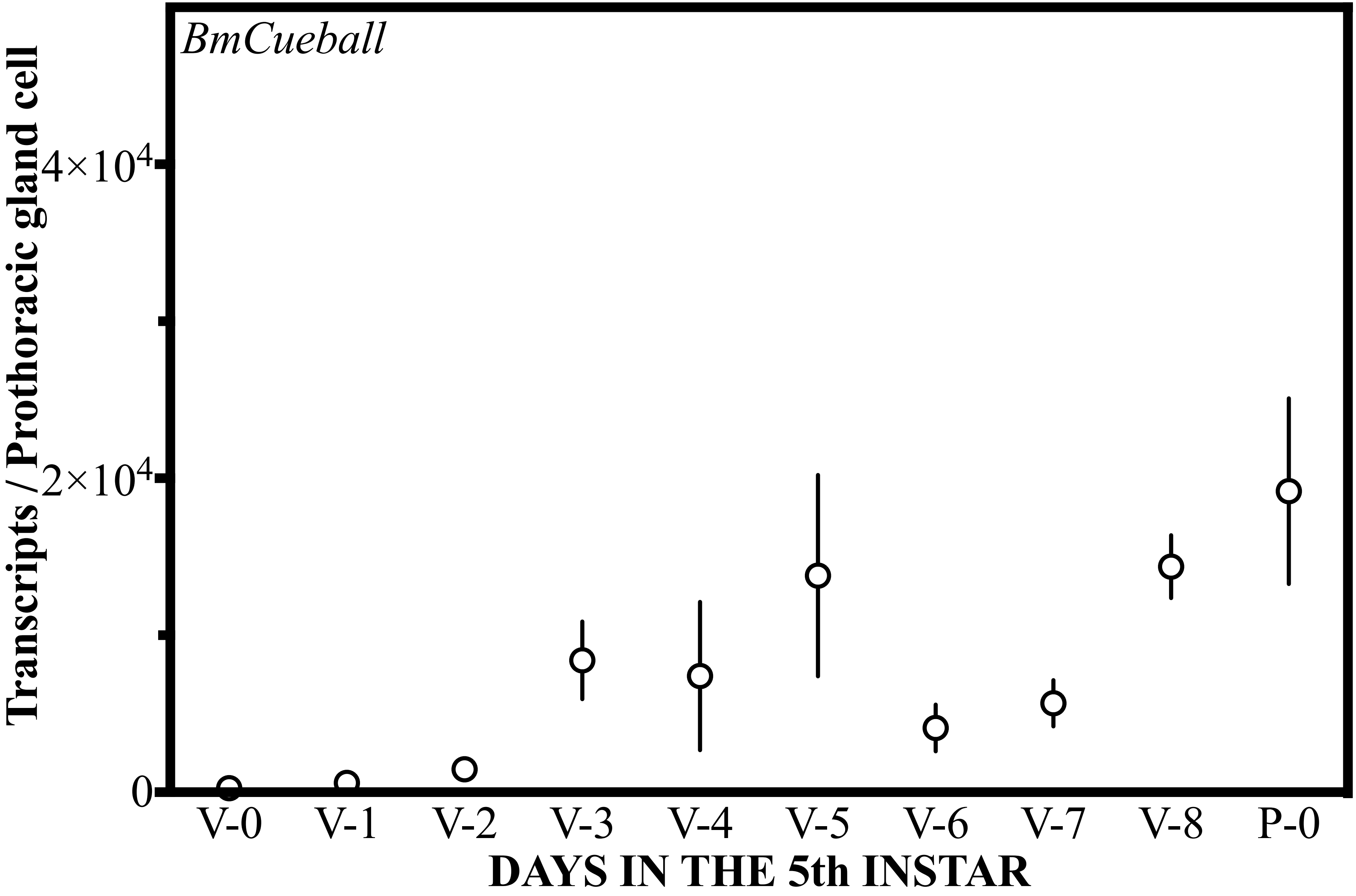

BmCueball
Transcripts / Prothoracic gland cell
4×10^4
2×10^4
0
V-0
V-1
V-2
V-3
V-4
V-5
V-6
V-7
V-8
P-0
DAYS IN THE 5th INSTAR

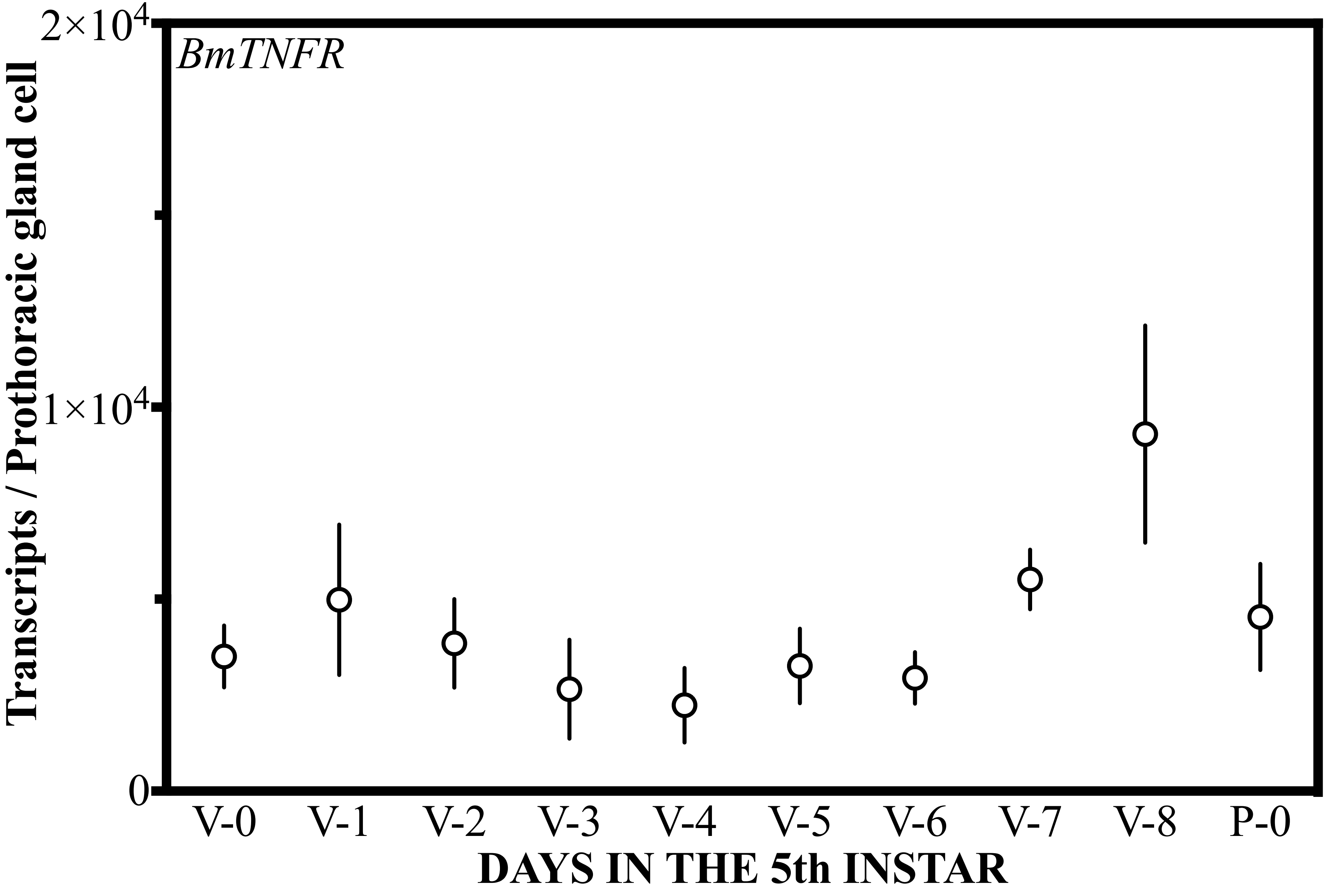
BmTNFR
Transcripts / Prothoracic gland cell
2×10^4
1×10^4
0
V-0
V-1
V-2
V-3
V-4
V-5
V-6
V-7
V-8
P-0
DAYS IN THE 5th INSTAR

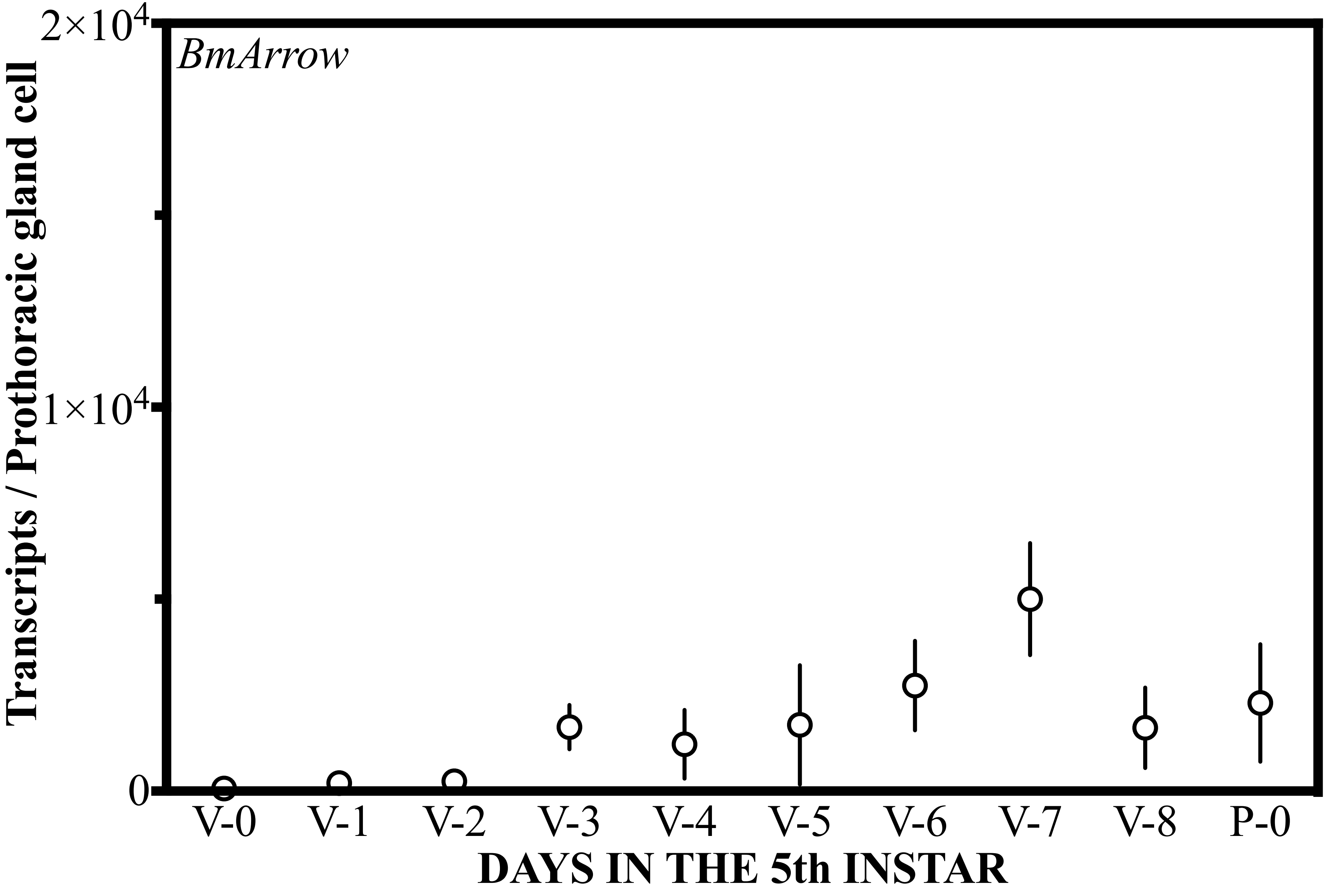

BmArrow
2×10⁴
1×10⁴
0
Transcripts / Prothoracic gland cell
V-0
V-1
V-2
V-3
V-4
V-5
V-6
V-7
V-8
P-0
DAYS IN THE 5th INSTAR

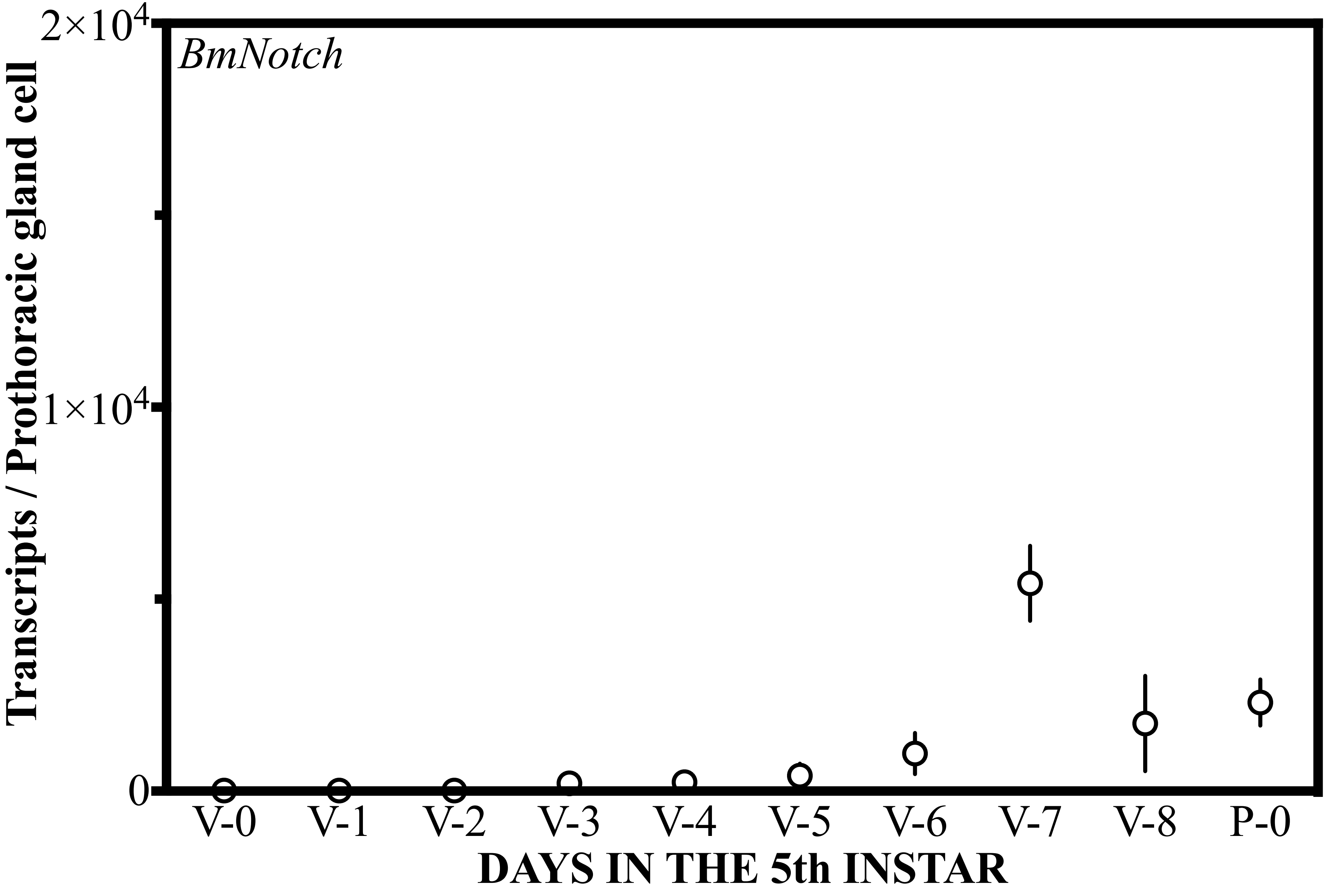
BmNotch
Transcripts / Prothoracic gland cell
2×10^4
1×10^4
0
V-0
V-1
V-2
V-3
V-4
V-5
V-6
V-7
V-8
P-0
DAYS IN THE 5th INSTAR

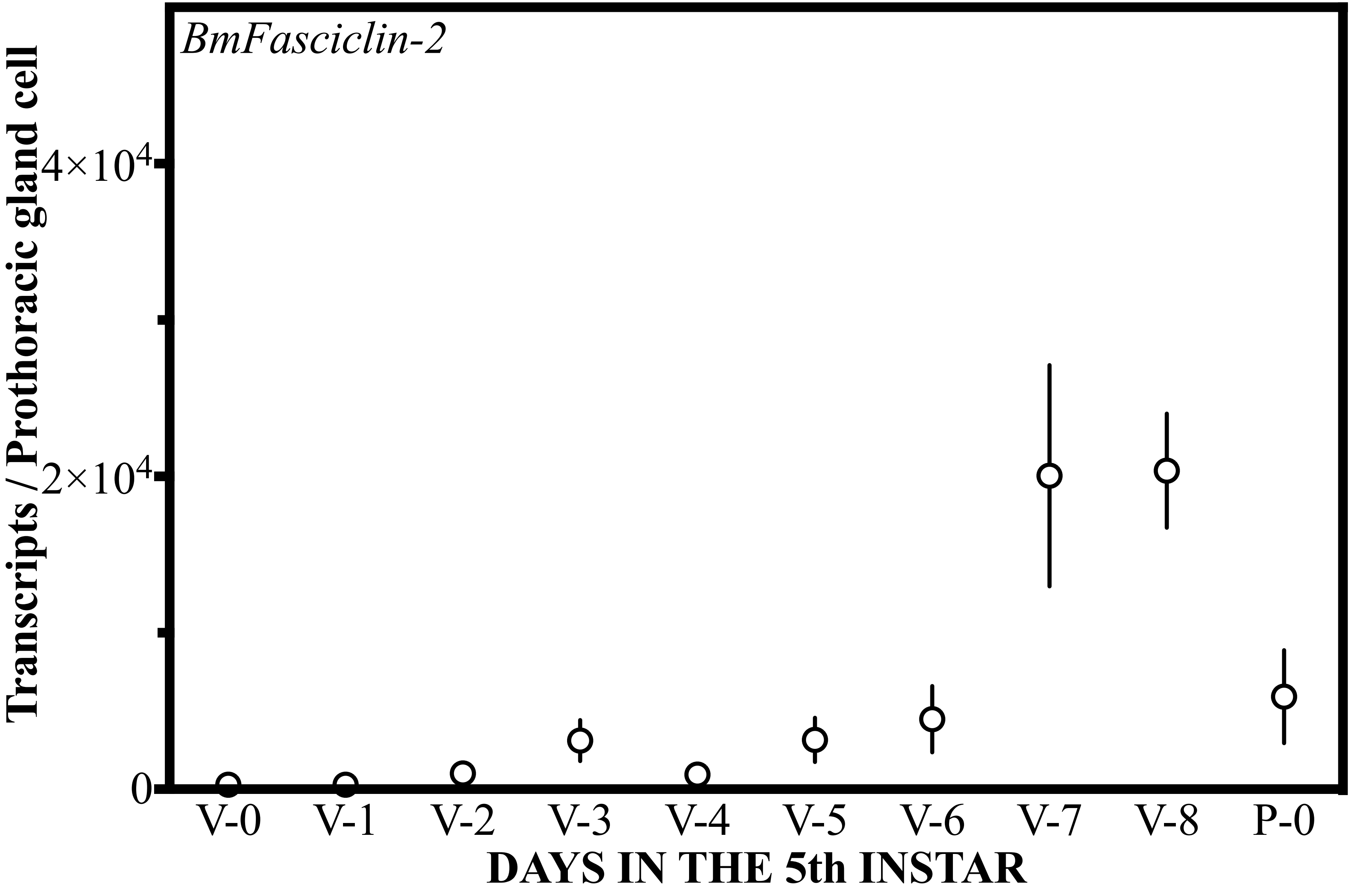

BmFasciclin-2
Transcripts / Prothoracic gland cell
4×10⁴
2×10⁴
0
V-0
V-1
V-2
V-3
V-4
V-5
V-6
V-7
V-8
P-0
DAYS IN THE 5th INSTAR

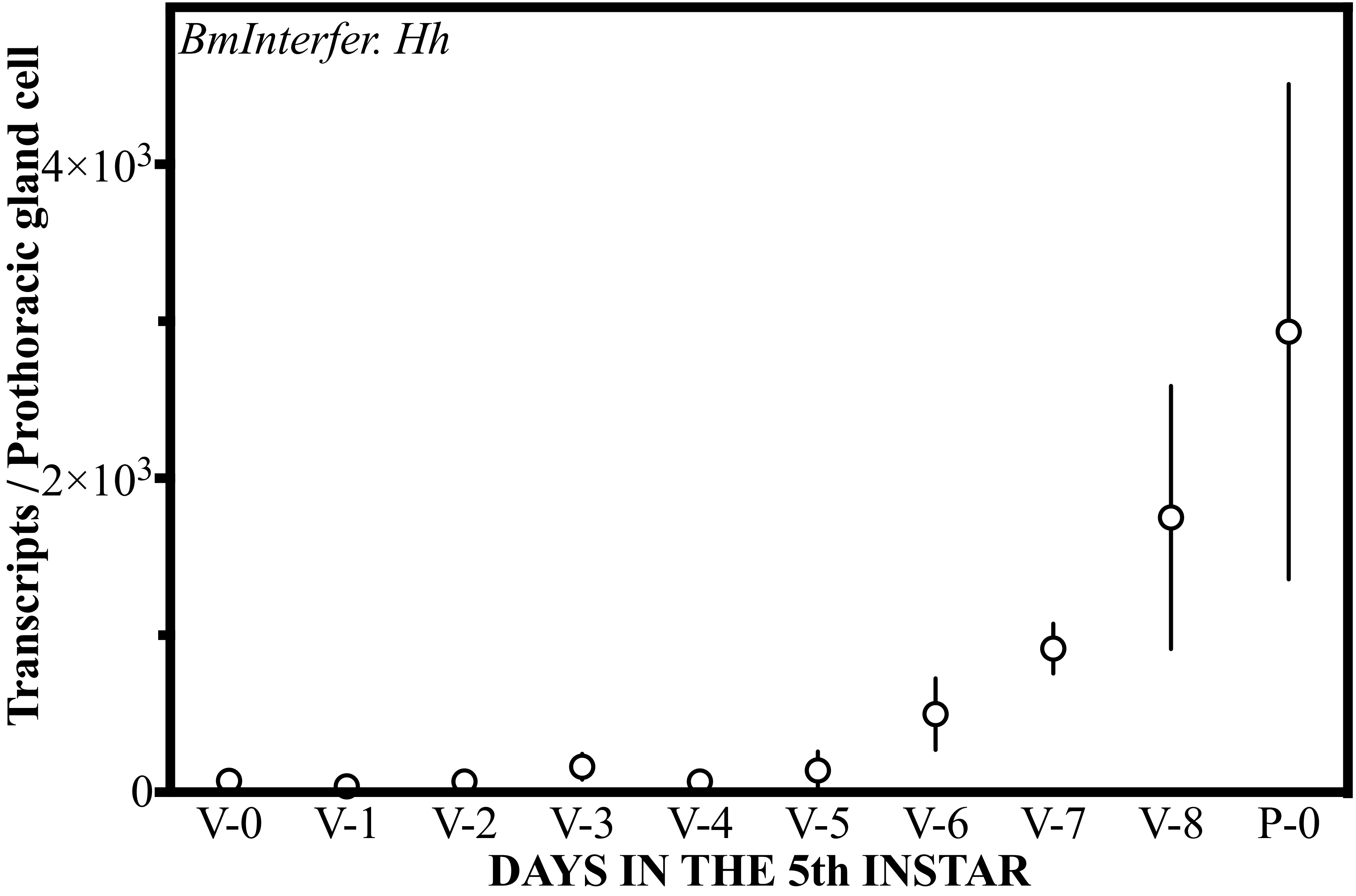
BmInterfer. Hh
Transcripts / Prothoracic gland cell
4×10³
2×10³
0
V-0
V-1
V-2
V-3
V-4
V-5
V-6
V-7
V-8
P-0
DAYS IN THE 5th INSTAR

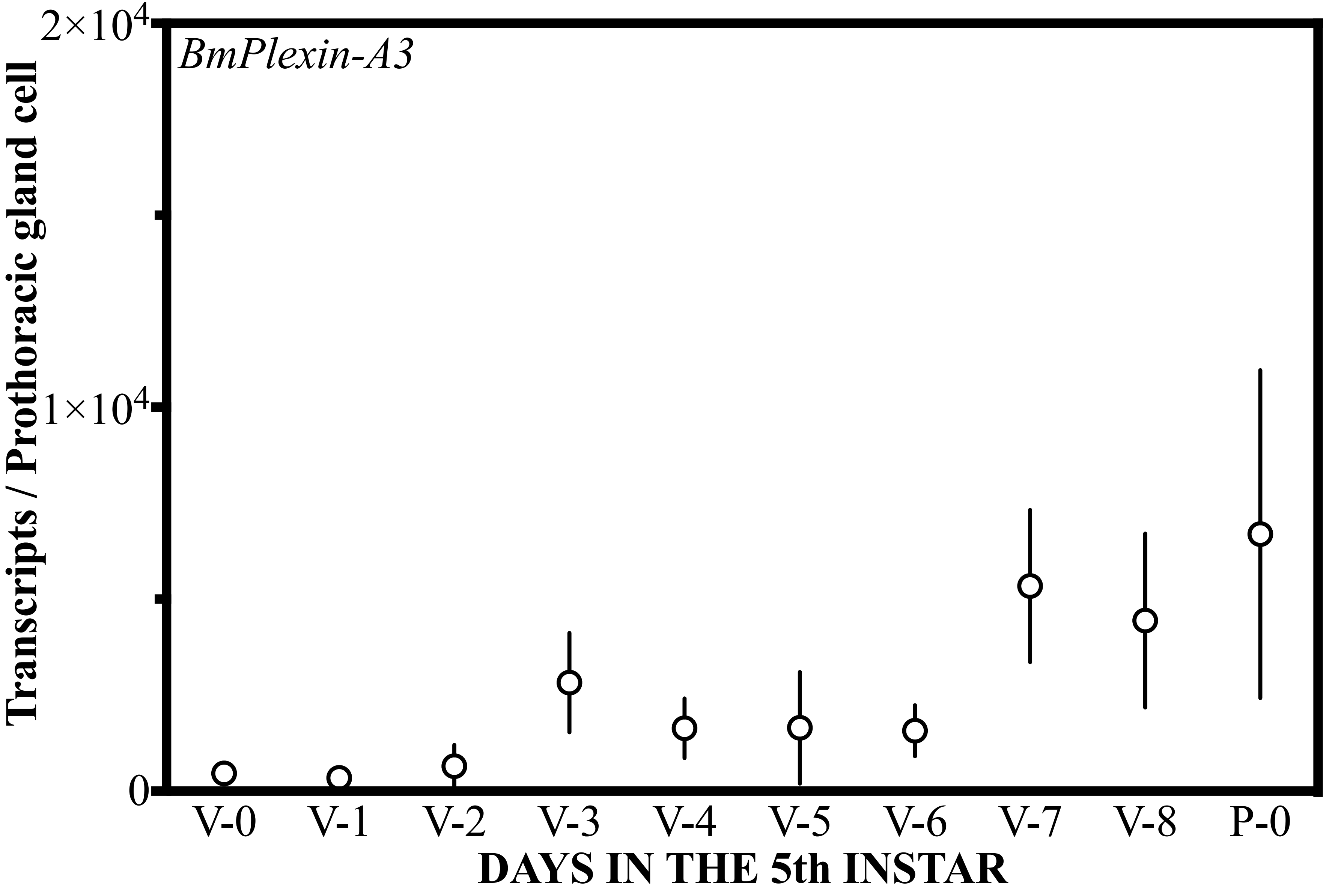

BmPlexin-A3
2×10^4
1×10^4
0
Transcripts / Prothoracic gland cell
V-0
V-1
V-2
V-3
V-4
V-5
V-6
V-7
V-8
P-0
DAYS IN THE 5th INSTAR

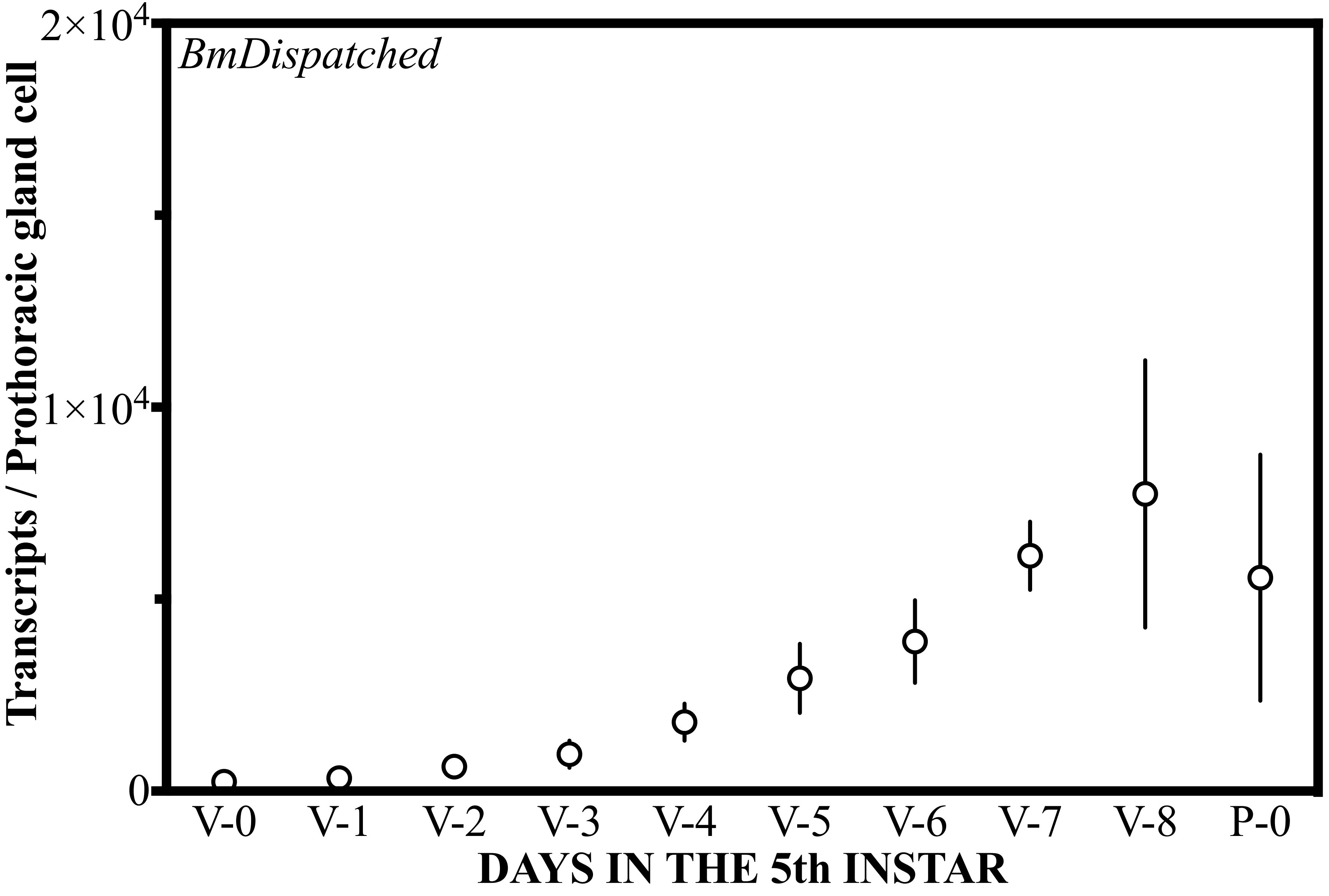

BmDispatched
2×10^4
1×10^4
0
Transcripts / Prothoracic gland cell
V-0
V-1
V-2
V-3
V-4
V-5
V-6
V-7
V-8
P-0
DAYS IN THE 5th INSTAR

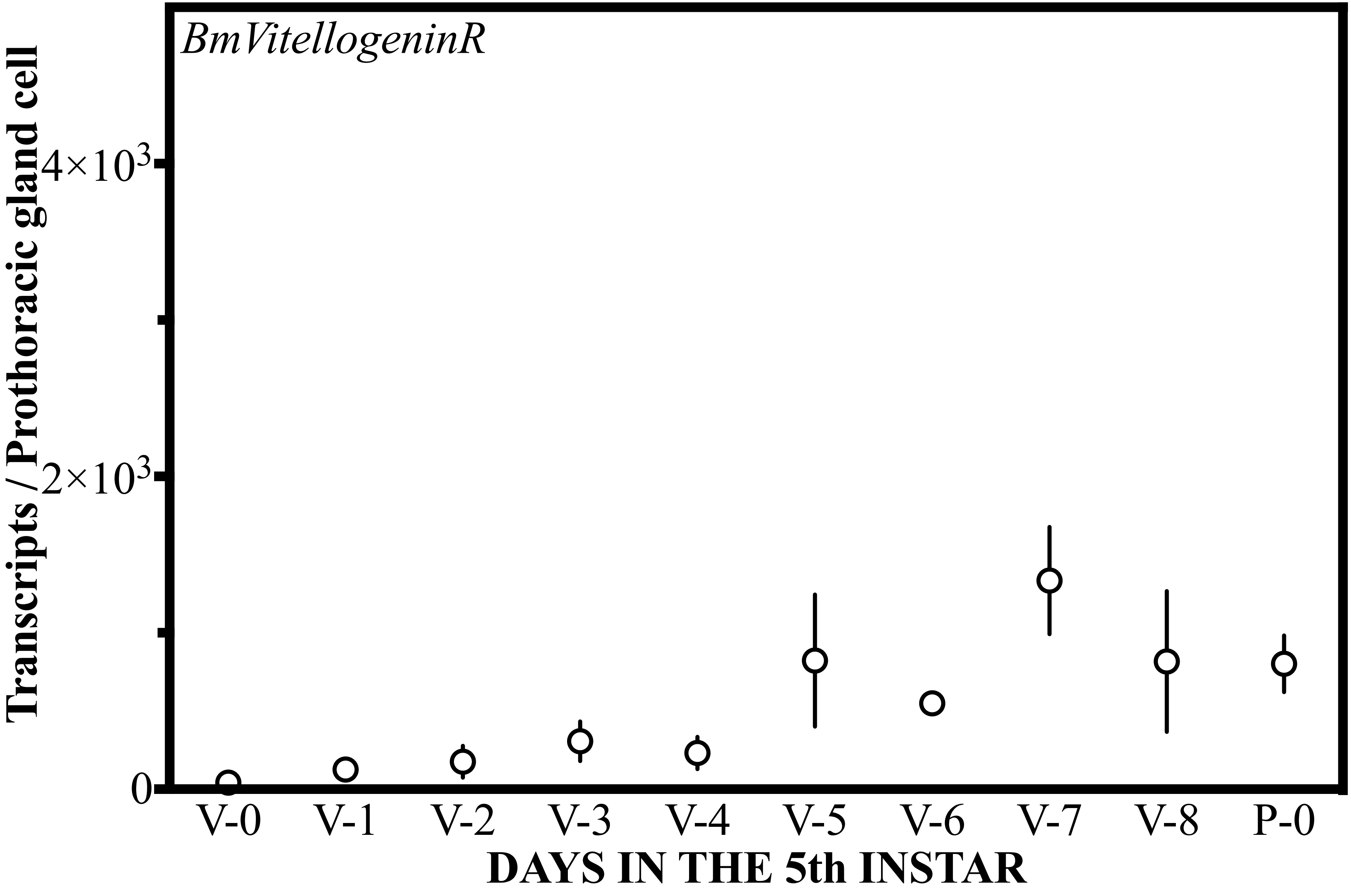
BmVitellogeninR
Transcripts / Prothoracic gland cell
4×10³
2×10³
0
V-0
V-1
V-2
V-3
V-4
V-5
V-6
V-7
V-8
P-0
DAYS IN THE 5th INSTAR

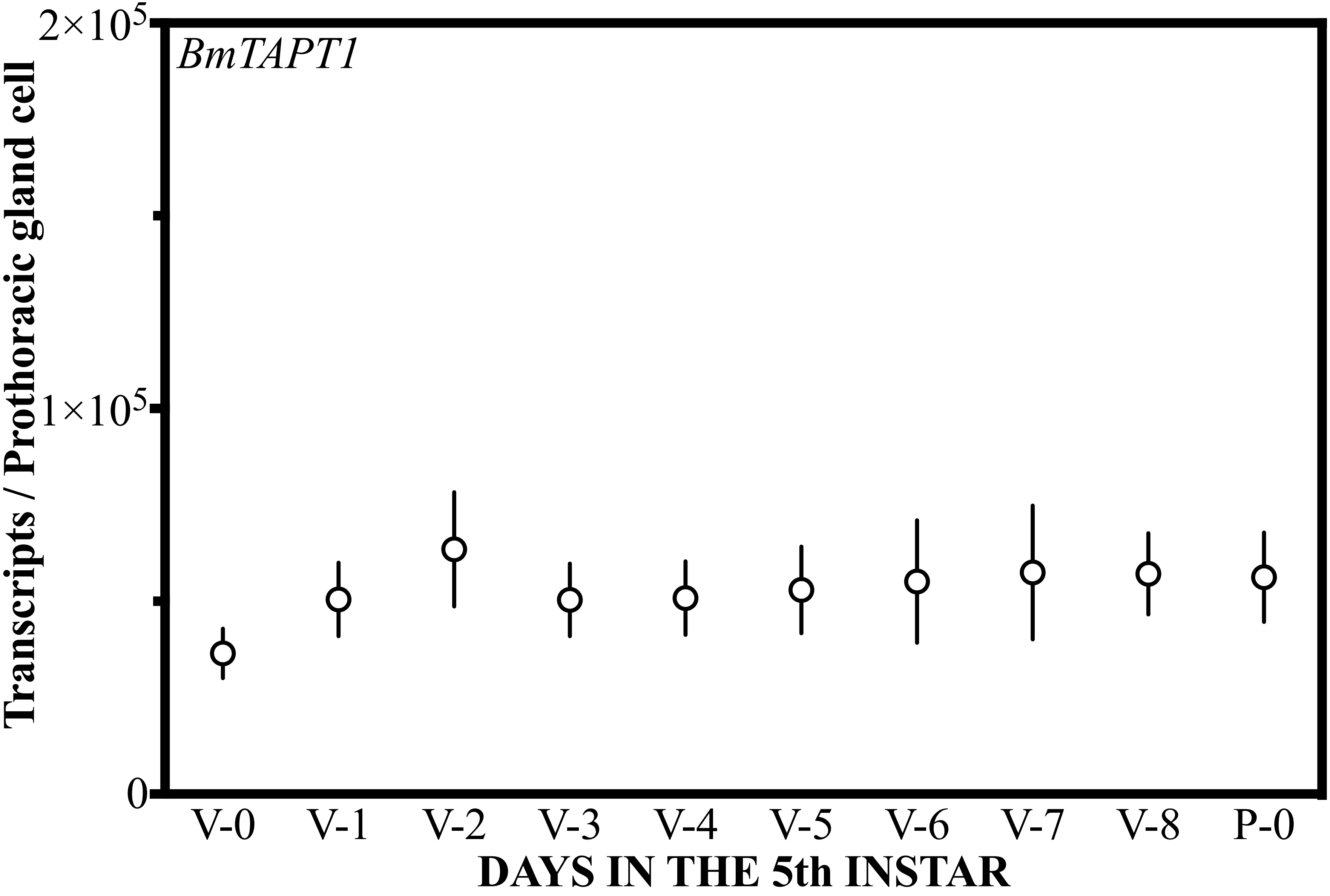

BmTAPT1
2×10^5
1×10^5
0
Transcripts / Prothoracic gland cell
V-0
V-1
V-2
V-3
V-4
V-5
V-6
V-7
V-8
P-0
DAYS IN THE 5th INSTAR

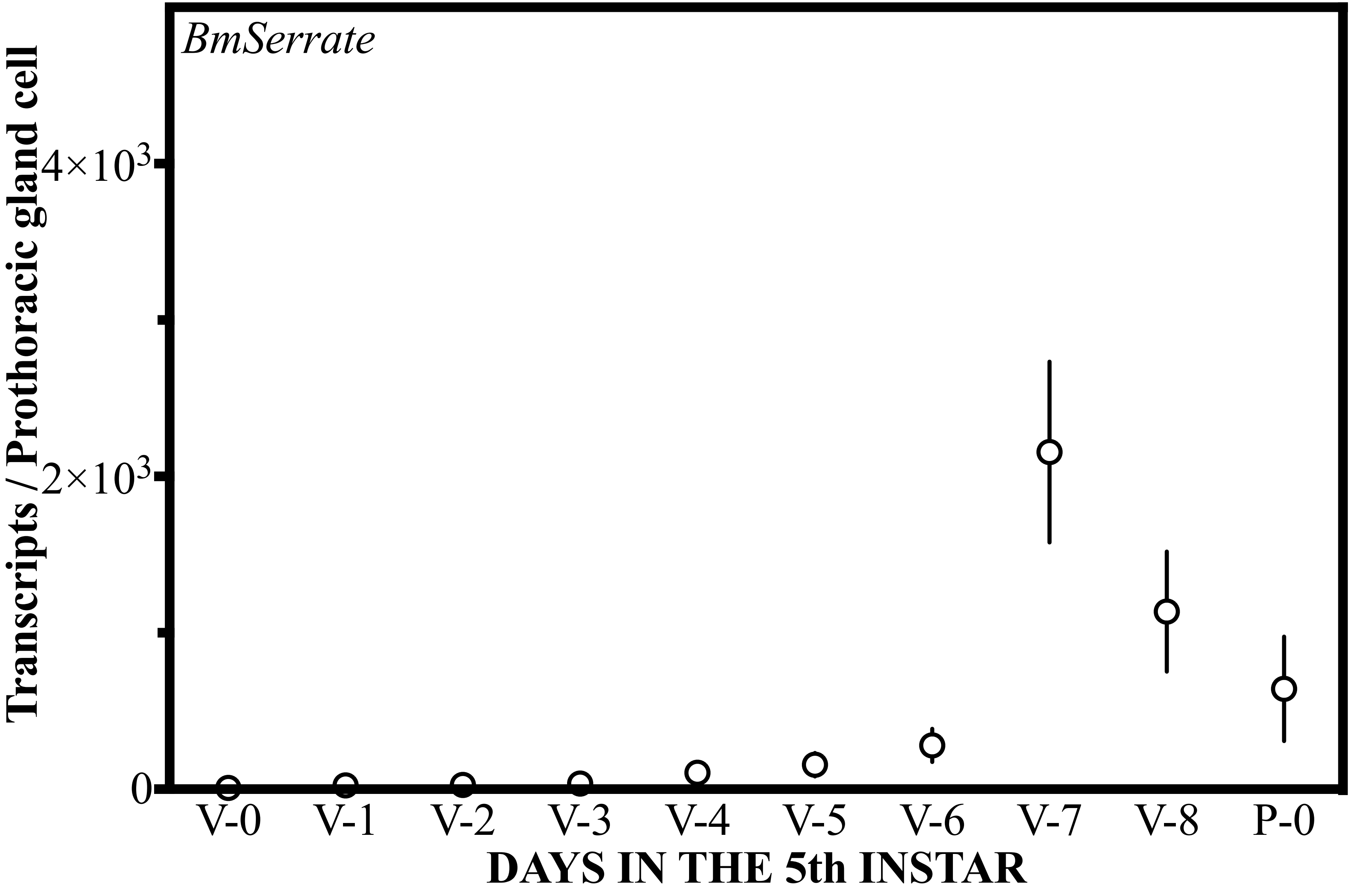
BmSerrate
Transcripts / Prothoracic gland cell
4×10^3
2×10^3
0
V-0
V-1
V-2
V-3
V-4
V-5
V-6
V-7
V-8
P-0
DAYS IN THE 5th INSTAR

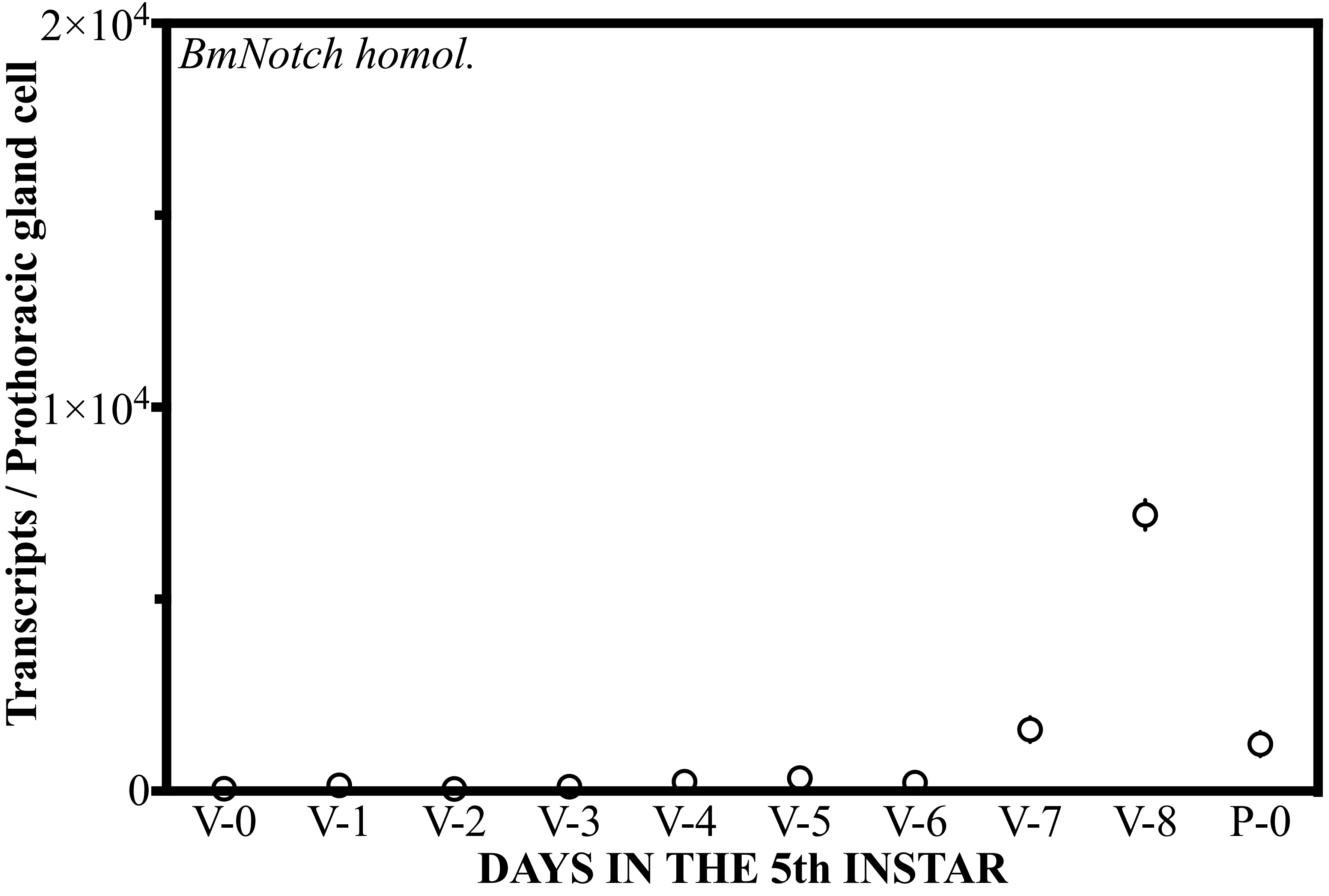

BmNotch homol.
2×10^4
1×10^4
0
Transcripts / Prothoracic gland cell
V-0
V-1
V-2
V-3
V-4
V-5
V-6
V-7
V-8
P-0
DAYS IN THE 5th INSTAR

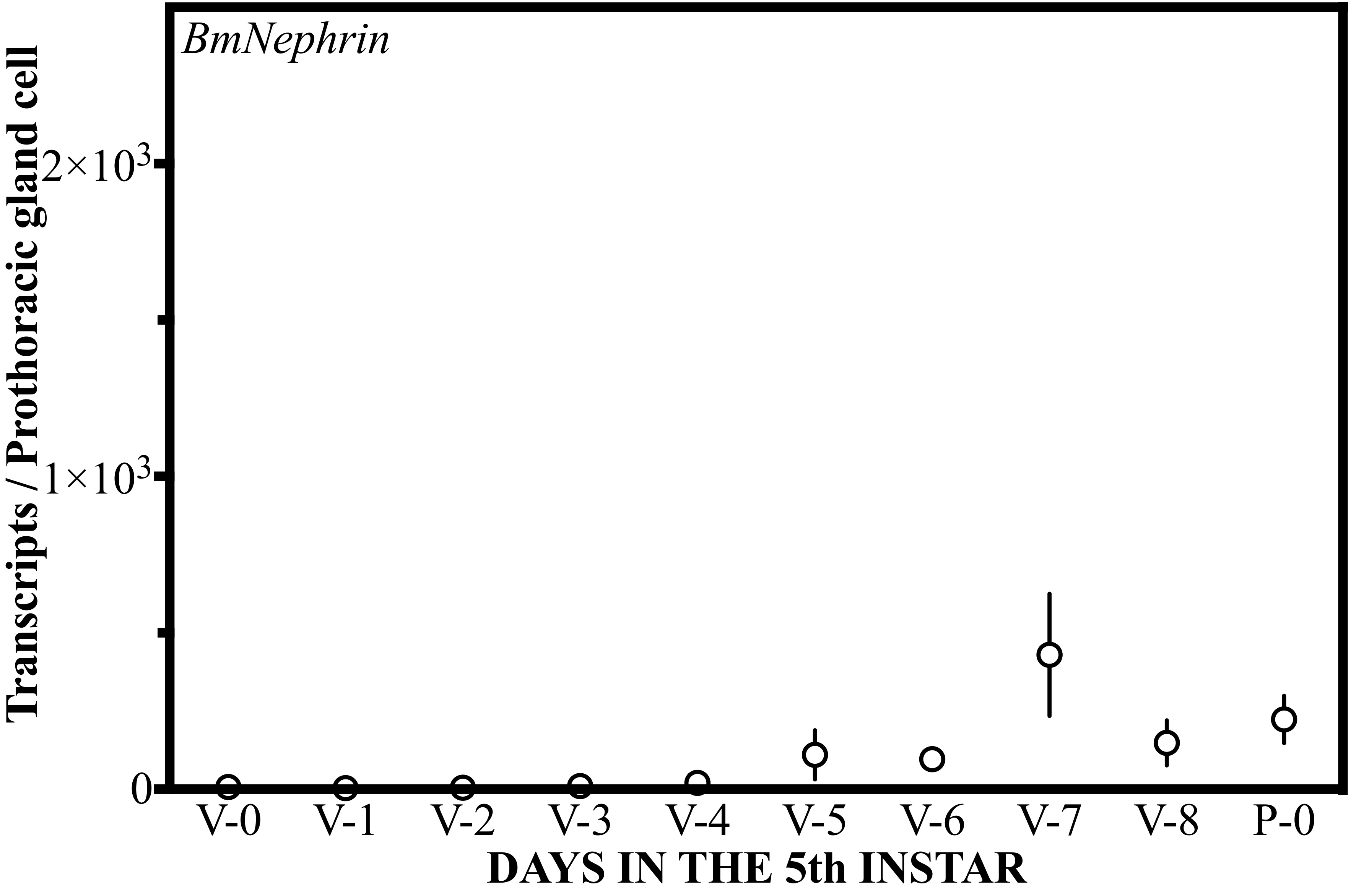

BmNephrin
Transcripts / Prothoracic gland cell
2×10³
1×10³
0
V-0
V-1
V-2
V-3
V-4
V-5
V-6
V-7
V-8
P-0
DAYS IN THE 5th INSTAR

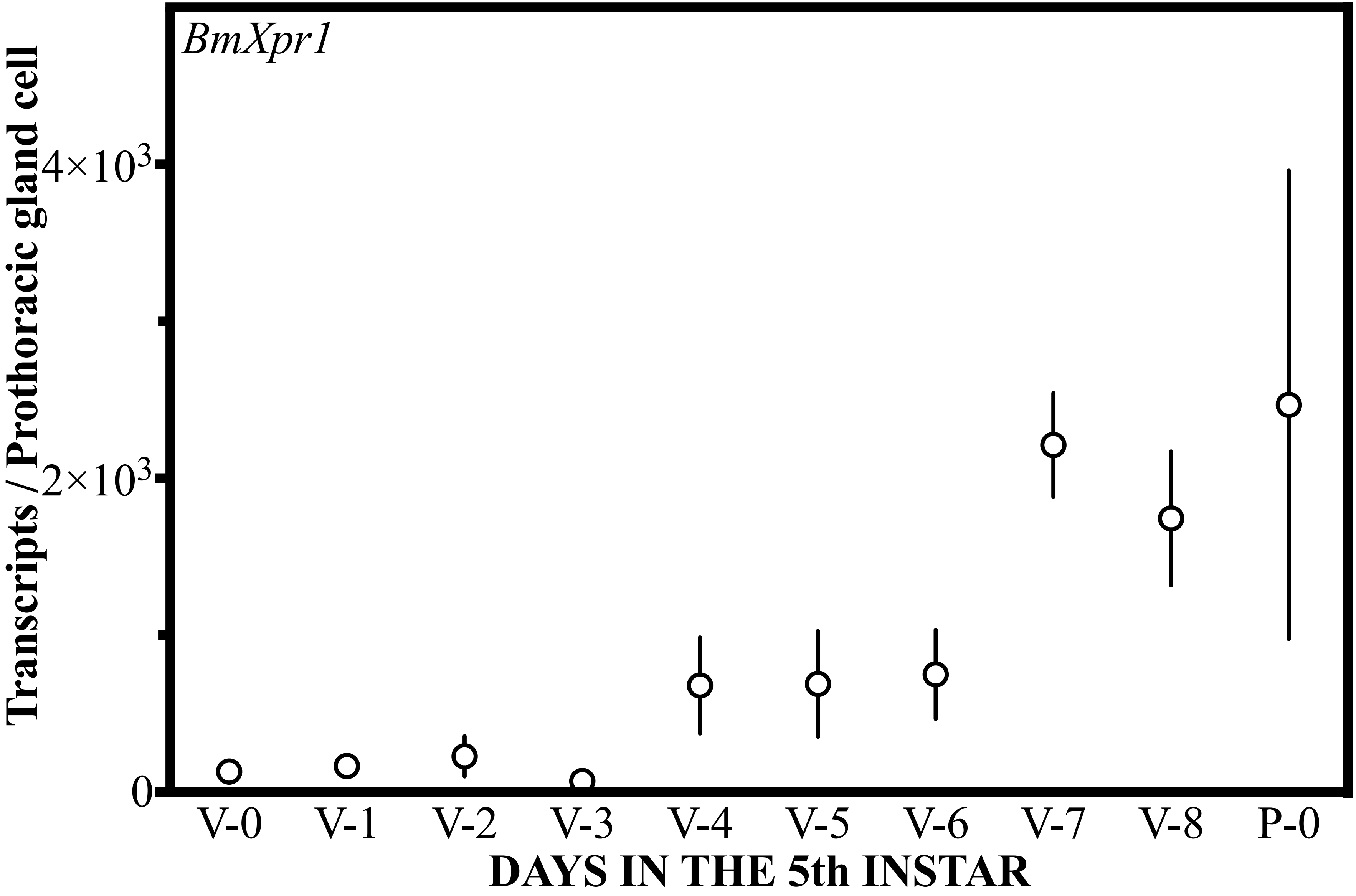
BmXpr1
Transcripts / Prothoracic gland cell
4×10³
2×10³
0
V-0
V-1
V-2
V-3
V-4
V-5
V-6
V-7
V-8
P-0
DAYS IN THE 5th INSTAR

Reference genes

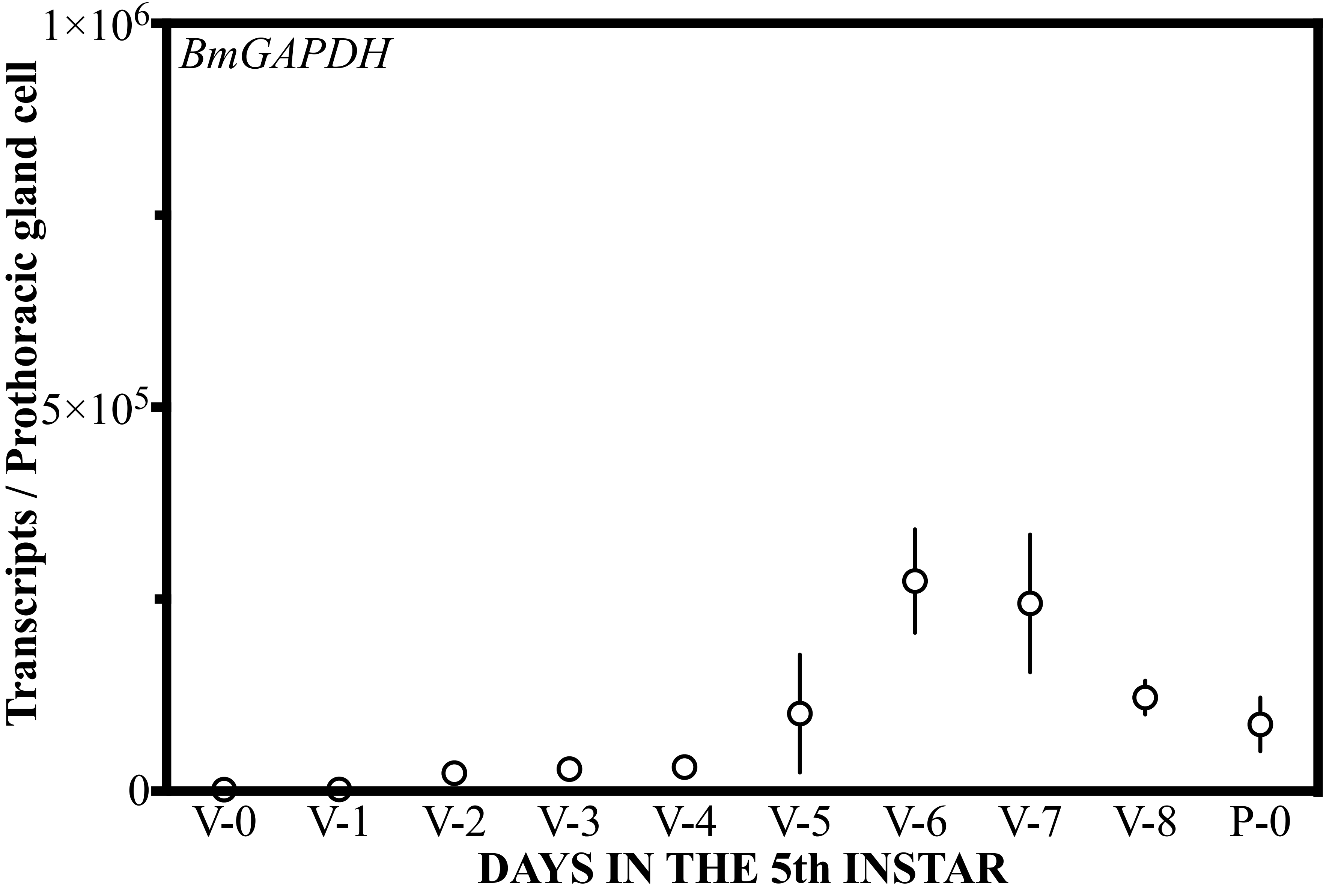
BmGAPDH
Transcripts / Prothoracic gland cell
1×10^6
5×10^5
0
V-0
V-1
V-2
V-3
V-4
V-5
V-6
V-7
V-8
P-0
DAYS IN THE 5th INSTAR

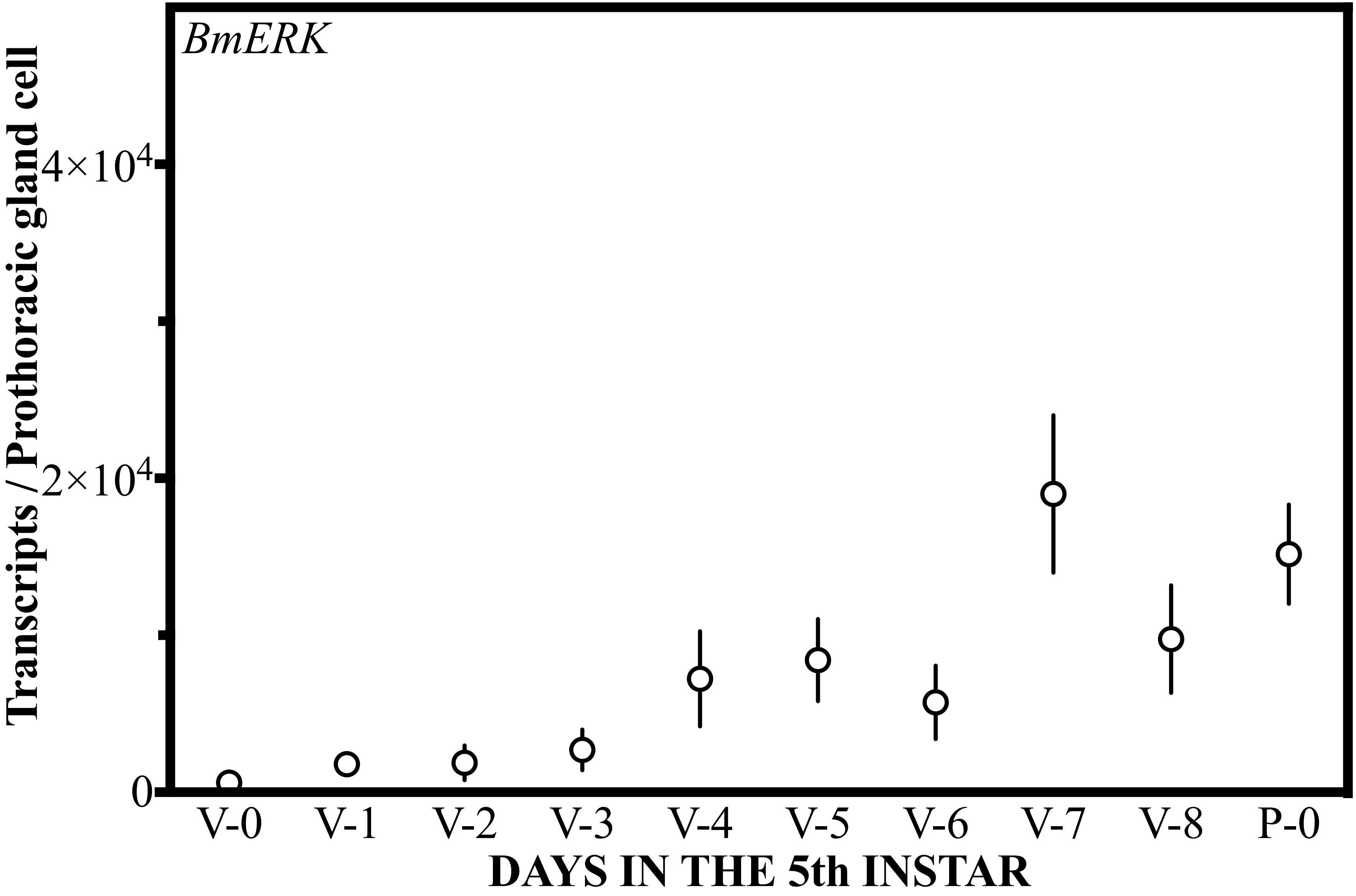
BmERK
Transcripts / Prothoracic gland cell
4×10⁴
2×10⁴
0
V-0
V-1
V-2
V-3
V-4
V-5
V-6
V-7
V-8
P-0
DAYS IN THE 5th INSTAR

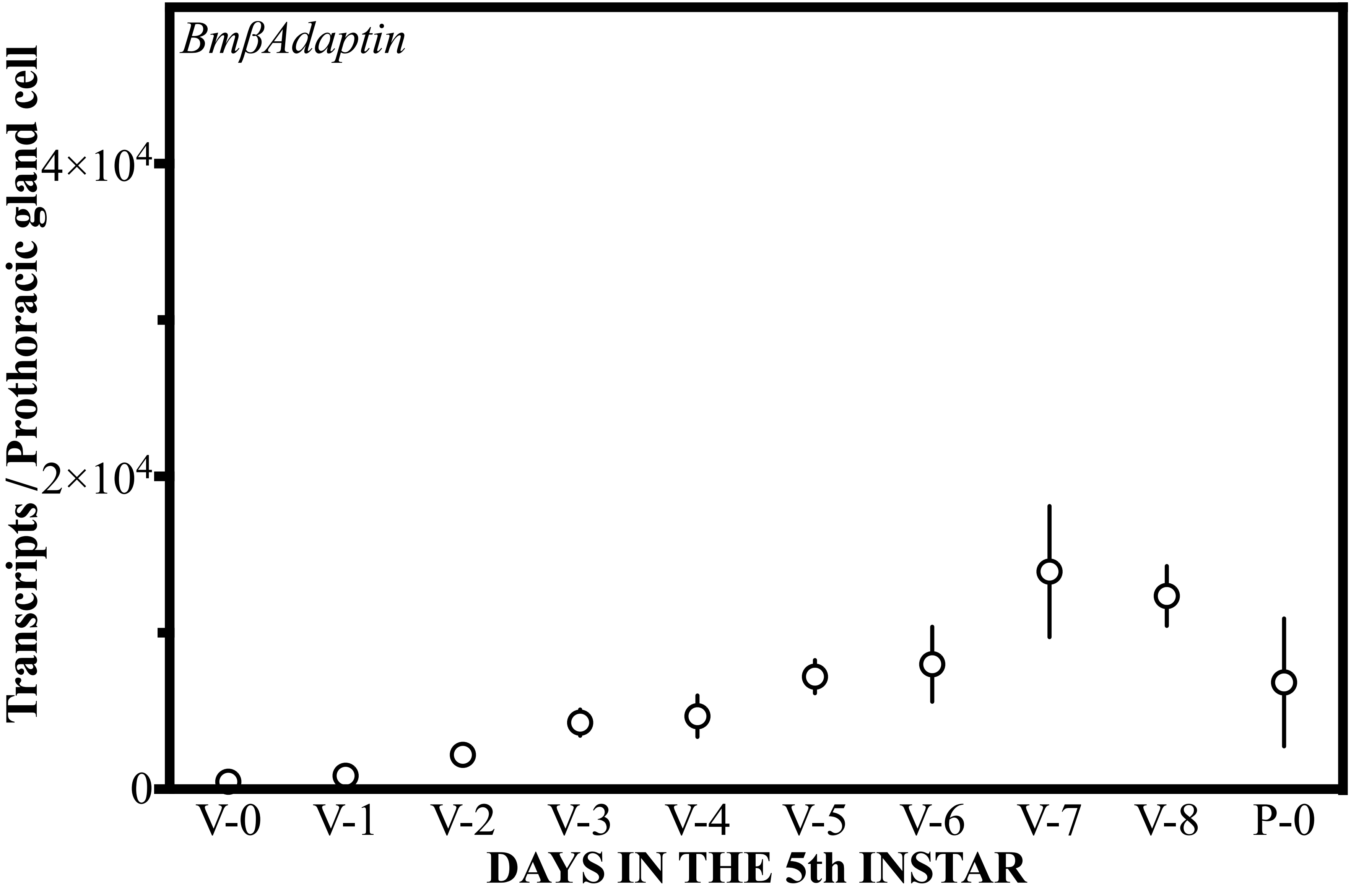

BmβAdaptin
Transcripts / Prothoracic gland cell
4×10^4
2×10^4
0
V-0
V-1
V-2
V-3
V-4
V-5
V-6
V-7
V-8
P-0
DAYS IN THE 5th INSTAR

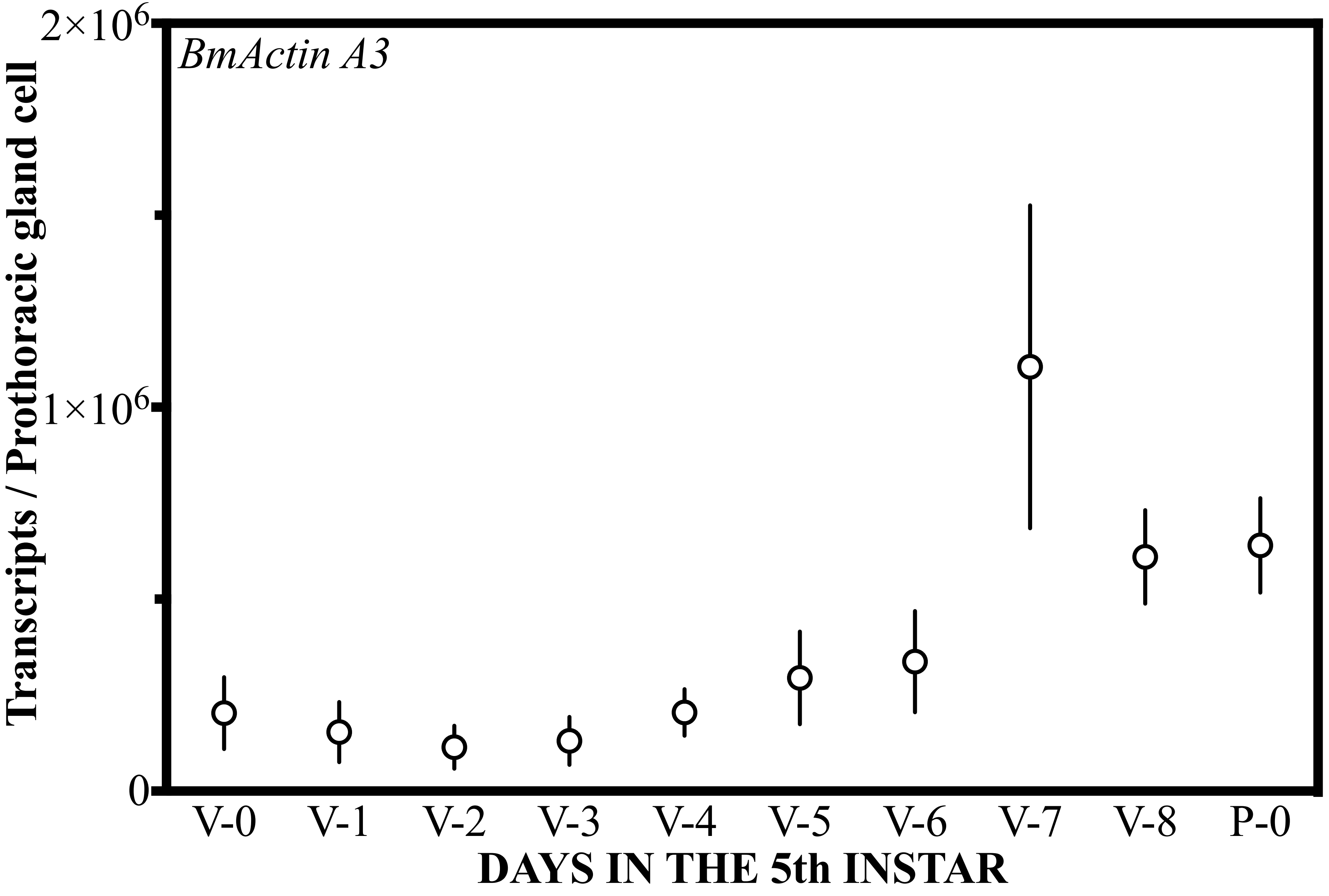

BmActin A3
2×10^6
1×10^6
0
Transcripts / Prothoracic gland cell
V-0
V-1
V-2
V-3
V-4
V-5
V-6
V-7
V-8
P-0
DAYS IN THE 5th INSTAR

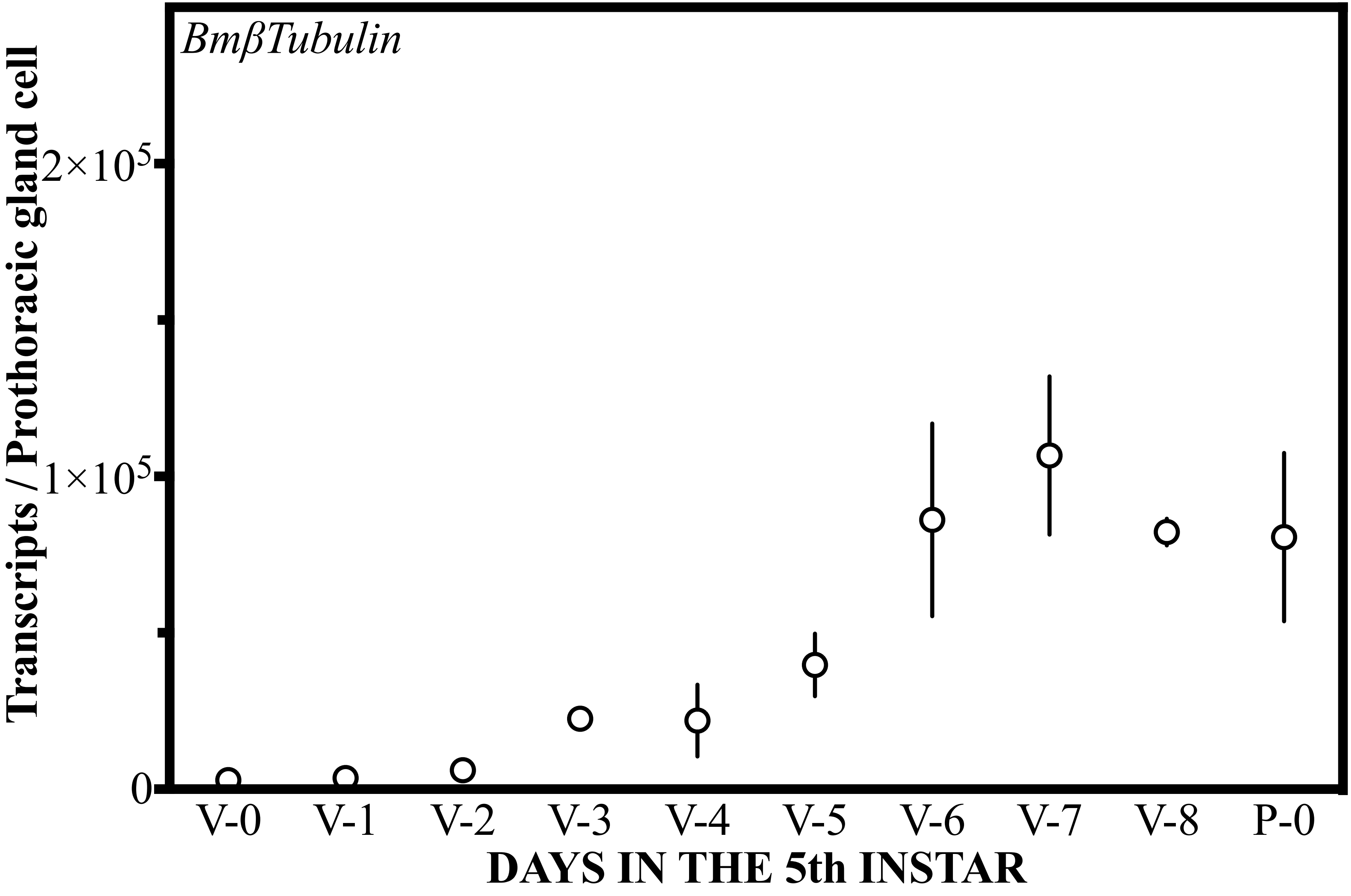

BmβTubulin
Transcripts / Prothoracic gland cell
2×10^5
1×10^5
0
V-0
V-1
V-2
V-3
V-4
V-5
V-6
V-7
V-8
P-0
DAYS IN THE 5th INSTAR

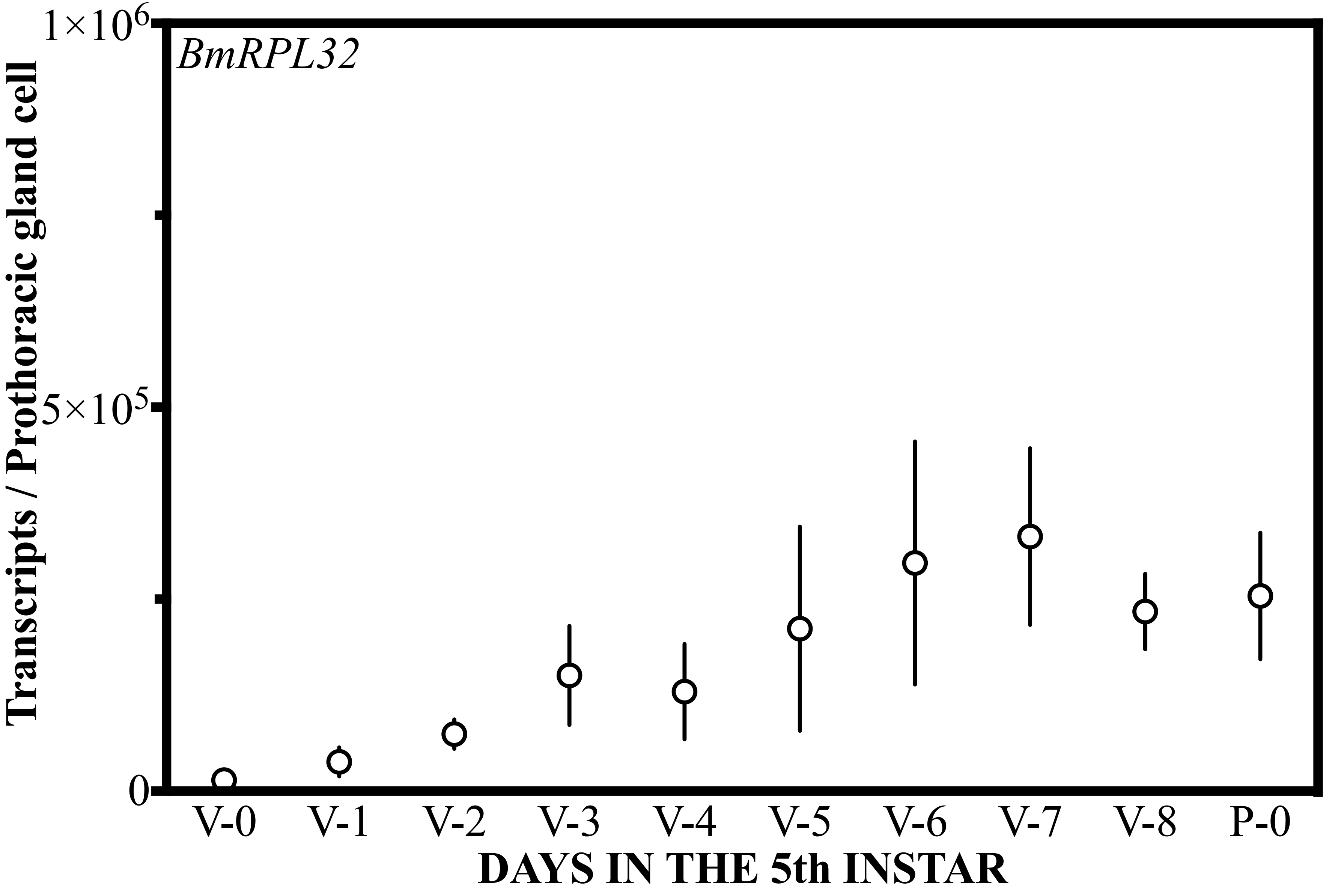

BmRPL32
1×10^6
5×10^5
0
Transcripts / Prothoracic gland cell
V-0
V-1
V-2
V-3
V-4
V-5
V-6
V-7
V-8
P-0
DAYS IN THE 5th INSTAR
